# Global proteomic analysis of extracellular matrix in mouse and human brain highlights relevance to cerebrovascular disease

**DOI:** 10.1177/0271678X211004307

**Published:** 2021-03-17

**Authors:** Alexandra Pokhilko, Gaia Brezzo, Lahiru Handunnetthi, Raphael Heilig, Rachel Lennon, Colin Smith, Stuart M Allan, Alessandra Granata, Sanjay Sinha, Tao Wang, Hugh S Markus, Alexandra Naba, Roman Fischer, Tom Van Agtmael, Karen Horsburgh, M Zameel Cader

**Affiliations:** 1 Translational Molecular Neuroscience Group, Weatherall Institute of Molecular Medicine, Nuffield Department of Clinical Neurosciences, University of Oxford, Oxford, UK; 2 Centre for Discovery Brain Sciences, University of Edinburgh, Edinburgh, UK; 3 Institute of Cardiovascular and Medical Sciences, University of Glasgow, Glasgow, UK; 4 Wellcome Centre for Human Genetics, University of Oxford, Oxford, UK; 5 Discovery Proteomics Facility, Target Discovery Institute, Nuffield Department of Medicine, University of Oxford, Oxford, UK; 6 Division of Cell-Matrix Biology and Regenerative Medicine, Wellcome Centre for Cell-Matrix Research, School of Biological Sciences, Faculty of Biology, Medicine and Health, Manchester Academic Health Science Centre, The University of Manchester, Manchester, UK; 7 Department of Paediatric Nephrology, Royal Manchester Children's Hospital, Manchester University Hospitals National Health Service Foundation Trust, Manchester Academic Health Science Centre, Manchester, UK; 8 Centre for Clinical Brain Sciences, University of Edinburgh, Edinburgh, UK; 9 Lydia Becker Institute of Immunology and Inflammation, Division of Neuroscience and Experimental Psychology, School of Biological Sciences, Faculty of Biology, Medicine and Health, The University of Manchester, Manchester Academic Health Science Centre, Manchester, UK; 10 Clinical Neurosciences Department, University of Cambridge, Cambridge, UK; 11 Anne McLaren Lab, Forvie Site, Cambridge, UK; 12 Faculty of Biology, Medicine and Health, The University of Manchester, Manchester, UK; 13 Department of Neurology, Cambridge University Hospitals NHS Foundation Trust, Cambridge, UK; 14 Department of Physiology and Biophysics, University of Illinois at Chicago, Chicago, IL, USA

**Keywords:** Cerebrovascular, proteome, extracellular matrix, basement membrane, matrisome

## Abstract

The extracellular matrix (ECM) is a key interface between the cerebrovasculature and adjacent brain tissues. Deregulation of the ECM contributes to a broad range of neurological disorders. However, despite this importance, our understanding of the ECM composition remains very limited mainly due to difficulties in its isolation. To address this, we developed an approach to extract the cerebrovascular ECM from mouse and human post-mortem normal brain tissues. We then used mass spectrometry with off-line high-pH reversed-phase fractionation to increase the protein detection. This identified more than 1000 proteins in the ECM-enriched fraction, with > 66% of the proteins being common between the species. We report 147 core ECM proteins of the human brain vascular matrisome, including collagens, laminins, fibronectin and nidogens. We next used network analysis to identify the connection between the brain ECM proteins and cerebrovascular diseases. We found that genes related to cerebrovascular diseases, such as *COL4A1*, *COL4A2*, *VCAN* and *APOE* were significantly enriched in the cerebrovascular ECM network. This provides unique mechanistic insight into cerebrovascular disease and potential drug targets. Overall, we provide a powerful resource to study the functions of brain ECM and highlight a specific role for brain vascular ECM in cerebral vascular disease.

## Introduction

Brain function depends on the finely tuned interplay and communication between cells within the neuro-glial-vascular unit (NGVU), a dynamic structure comprised of endothelial cells, astrocytes and their end-feet contacts, contractile cells (smooth muscle cells, pericytes), neurons and microglia.^1^ The NGVU serves a number of critical roles including cerebral blood flow regulation, formation and maintenance of the blood-brain barrier, controlling the exchange of substances between blood and brain, immune surveillance and regulation of drainage mechanisms. The extracellular matrix (ECM) proteins act as a complex meshwork providing essential structural support and functional stability within the NGVU. The protein components within the ECM, collectively called the matrisome, covers both core ECM proteins including glycoproteins, proteoglycans, glycosaminoglycans like collagens, and ECM associated proteins, such as ECM-modifying enzymes and ECM receptors.

The molecular composition of the ECM varies among tissues and continuously undergoes remodelling in both physiological and pathological states. Indeed, deregulation of the ECM proteins and breakdown of the NGVU underpin pathomolecular mechanisms in ageing, cerebrovascular disease and dementia.^2,3^ Notably, mutations in ECM genes cause monogenic forms of cerebrovascular disease.^4–6^ This includes mutations in collagen IV, a key component of the specialised basement membrane in the ECM.^7,8^ Furthermore, common variants in genes that code for ECM proteins are genetically associated with cerebral small vessel disease and stroke.^9–13^ Thus, changes in the composition of the ECM are likely to exert profound effects on all NGVU components. Despite its critical role in neurological disease, including cerebrovascular disease, very little is known about the components of the cerebrovascular ECM.

Biochemical analysis of the constituents of the ECM is challenging due to their cross-linked structure and insolubility. Advances in proteomics and mass-spectrometry (MS) combined with bioinformatics are unravelling the constituents of the ECM across many healthy and disease tissues.^14,15^ However, there is limited information on the brain matrisome and particularly that of the cerebrovasculature. To date most information has been derived indirectly from proteomic evaluation of the cerebrovasculature, including our previous work which described the mouse cerebrovascular proteome.^16^ This represents a significant knowledge-gap that needs to be bridged to uncover the mechanisms by which the ECM can affect cerebrovascular health.

In this study we characterise the proteome of the cerebrovascular ECM in mouse and human brains. We generate a new pipeline by combining our published methodology to define the cerebrovascular proteome^16^ with an experimental technique used to enrich insoluble ECM proteins from the glomerulus.^17^ We subsequently evaluate the human cerebrovascular ECM proteome in relation to genetic susceptibility to cerebrovascular disease including small vessel disease and ischemic stroke.

## Materials and methods

### Animals

All animal experiments were conducted in accordance with the Animal (Scientific Procedures) Act 1986, local ethical approval at the University of Edinburgh, and performed under appropriate personal and project licences authority granted by the Home Office, and in compliance with the ARRIVE guidelines for animal research. C57BL/6J (wild-type, WT) mice were obtained from Charles River (UK) and group housed on a 12-hour light/dark cycle with *ad libitum* access to food and water. A total of *n = 3* adult male mice aged 4 months were used in this study. Young adult brains were used to avoid any confounding effects of age on the vascular proteome. All tissues were collected at the same time of day to avoid any differences due to circadian rhythm.

### Human subjects

Brain tissue samples were taken from the basal ganglia of n = 3 control subjects (male, aged 33, 34 and 39) in the Edinburgh Sudden Death Brain Bank. The subjects had no clinical or neuropathological evidence of a chronic illness or neurological condition including cerebral vascular disease. The Edinburgh Sudden Death Brain Bank has ethics approval from East of Scotland Research Ethics Service (16/ES/0084) in line with the Human Tissue (Scotland) Act.

### Cerebral vessel enrichment

Mice were sacrificed under deep isoflurane anaesthesia by transcardiac perfusion with heparinized phosphate buffered saline. Brains were removed, sectioned into left and right hemispheres and frozen in liquid nitrogen. Hemibrains were stored at −80°C until processing. Left hemispheres (hemibrains) were used for vessel and subsequent ECM enrichment. Vessels were enriched using modifications of our previously published protocols.^16,18^ All steps were performed on ice or at 4 °C to avoid proteolysis. Briefly, human basal ganglia (approx. 0.3-0.4g) and mouse hemibrains (approx. 0.2 g) were homogenised with 1 mL ice-cold PBS using a glass hand-held loose fit dounce homogeniser with 20 strokes. Brain homogenates were then centrifuged at 250 g for 10 min to remove cellular debris and myelin. The pellets were re-suspended in 17.5% Ficoll solution (Sigma) and centrifuged for 25 min at 3,200 g to separate brain vessels. This pellet was retained on ice, whilst the supernatant was centrifuged again for 25 min at 3,200 g. Pellets from both spins were combined and re-suspended in 1 mL 1% BSA-PBS. This was further centrifuged for 10 min at 2,000 g, the pellets were washed in 1 mL PBS (to remove BSA) and stored at −80°C, as the vessel enriched fraction, until further processing.

### Extracellular matrix enrichment

The ECM enrichment protocol was modified from.^17^ This method is used to remove intracellular proteins, based on their solubility in detergent, and enrich for ECM proteins, that are, in contrast, more insoluble, reducing the complexity of protein samples for MS analysis. In our modified protocol, pellets of vessel enriched fractions were re-suspended in ice-cold extraction buffer (5:1 vol/vol, 10 mM Tris, 150 mM NaCl, 1% Triton-X-100, 25 mM EDTA with added protease and phosphatase inhibitors) to solubilise cellular proteins. Samples were left on a rotator at 4 °C for 1 hour and then centrifuged at 14000 g for 10 min. The supernatant (Fraction A) was then removed and stored at 4 °C. The remaining pellet was re-suspended in an ice-cold alkaline detergent buffer (5:1 vol/vol, 20 mM NH_4_OH and triton X-100) to disrupt cell-to-cell interactions, left on a rotator at 4 °C for 1 hour and centrifuged at 14000 g for 10 minutes at 4 °C and the supernatant was collected as Fraction B. The remaining pellet is then re-suspended in sample buffer (5:1 vol/vol, 7% SDS, 30% glycerol, 200 mM Tris-HCl) to solubilise the ECM and to yield the final **ECM-enriched fraction**. Fractions A and B were combined to generate a **cellular fraction**. Proteins were then prepared to a concentration of 20 µg for proteomic analysis.

### SDS-PAGE and Western blotting

Protein levels for vessel enriched, ECM-enriched and cellular fractions from mouse and human were determined using a Pierce BCA protein assay kit (Thermo Scientific, UK). Samples were denatured at 70 °C for 20 min and resolved by SDS-PAGE. Proteins (8 µg) were transferred to a PVDF membrane, blocked in Odyssey Blocking Buffer in PBS (Licor, UK) and incubated with primary antibodies diluted in Odyssey Blocking Buffer and 0.1% PBS Tween overnight at 4 °C. Vessel enriched fractions were probed with antibodies against blood vessel-associated proteins (PECAM, occludin, SMA) to ascertain vessel enrichment. ECM-derived fractions were probed with antibodies against ECM (e.g. laminin, fibronectin) and non-ECM components (eg. synaptophysin, GAPDH) to ascertain ECM enrichment (antibodies details are provided in Supplementary Table 1). Membranes were washed in 0.1% PBS-Tween and incubated for 1 hour at RT with species-specific fluorescent dye-conjugated secondary antibodies diluted in Odyssey Blocking Buffer and 0.1% PBS Tween. Membranes were washed with 0.1% PBS-Tween and imaged with the Odyssey CLx infrared imaging system (LICOR Biosciences, Cambridge, UK). Individual protein bands were normalised to the loading control α-tubulin and subsequently fold differences expressed relative to either the total fraction or ECM enriched fraction.

### Proteomics sample preparation

Samples were prepared in a 96-well S-Trap plate (Protifi LCC) as per manufacturer’s protocol. Briefly, samples were reduced with 5 µl 200 mM DTT and alkylated with 20 µl 200 mM IAM for 30 min each, acidified with 12% phosphoric acid 10:1 (v/v) sample to acid, transferred into the S-Trap well and precipitated with 7 parts 90% methanol in 100 mM TEAB to 1 part sample (v/v). Samples were washed 3x by spinning the S-trap plate at 1500xg for 30 s and the last step for 1 min, each time with fresh 90% methanol in 100 mM TEAB. The sample was resuspended in 50 µL 50 mM TEAB and digested with 0.8 µg trypsin (Promega) overnight at 37 °C. Peptides were eluted from the S-Trap by spinning for 1 min at 1500 g with 80 µL 50 mM ammonium bicarbonate, 80 µL 0.1% FA and finally 80 µL 50% ACN, 0.1% FA. The eluates were dried down in a vacuum centrifuge and resuspended in loading buffer (2% ACN, 0.1% TFA) prior to off-line high-pH reversed-phase fractionation (HpH fractionation) and mass spectrometry (MS) acquisition.

### Off-line high-pH reversed-phase fractionation on agilent bravo assaymap

RP-S cartridges were primed with 100 µL ACN at 300 µL/min, equilibrated with 50 µL loading buffer (2% ACN, 0.1% TFA) at 10 µL/min followed by loading 120 µL sample (with a peptide content of about 18.5 µg) at 5 µL/min. the cartridge cup was washed with 50 µL and an internal cartridge wash was performed with 25 µL loading buffer at 5 µL/min.

Each sample was divided into 8 fractions, which were then run individually on the MS platform, to determine how much additional depth arises from the fractionated sample. The 8 fractions (elution steps) were done with buffer A (Water, pH 10) and buffer B (90% ACN, pH 10) at the following percentages of buffer B: 5%, 10%, 12.5%, 15%, 20%, 22.5%, 25% and 50%. The eluates of the following steps were directly concatenated as 1 + 5, 2 + 6, 3 + 7 and 4 + 8. Fractions were dried down in a vacuum centrifuge and resuspended in loading buffer.

### LC-MS/MS data acquisition

Peptides (50-80ng) were loaded onto Evotips as described by the manufacturer. Briefly, Evotips were activated by soaking them in isopropanol, primed with 20 µL buffer B (ACN, 0.1% FA) by centrifugation for 1 min at 700 g. Tips were soaked in isopropanol for 1 s and equilibrated with 20 µL buffer A (water, 0.1% FA) by centrifugation. Another 20 µL buffer A was loaded onto the tips and the samples were added on top of that. Tips were spun and then washed with 20 µl buffer A followed by overlaying the C18 material in the tips with 100 µL buffer A and a short 20 s spin.

Samples were run on a LC-MS system comprised of an Evosep One LC and a Bruker timsTOF Pro. Peptides were separated on an 8 cm analytical C18 column (Evosep, 3 µm beads, 100 µm ID) using the pre-set 60 samples per day gradient on the Evosep one. MS data acquisition was done in PASEF mode (oTOF control v6.0.0.12). The ion mobility window was set to 1/k0 start = 0.85 Vs/cm^2^ to 1/k0 end = 1.3 Vs/cm^2^, ramp time 100 ms with locked duty cycle, mass range 100 - 1700 m/z. MS/MS were acquired in 4 PASEF frames (3 cycles overlap). For proteomics analysis, the raw data files were searched against either the reviewed Uniprot homo sapiens databased (retrieved 2,01,80,131) or mus musculus (retrieved 2,01,90,304) using MaxQuant^19^ version 1.6.10.43 and its built-in contaminant database using tryptic specificity and allowing two missed cleavages.

### Downstream analysis of proteomics data

ECM proteins were identified and categorized in our datasets based on the recently updated publicly available matrisome list (http://matrisome.org/^20,21^). The Venn diagrams and bar plots were based on proteins identified in two replicates with at least two unique peptides. Most of the analysis (including Venn diagrams, differential expression, identification of potential ECM-interacting and disease-associated proteins) was performed on datasets from which we filtered out chromatin-associated, nuclear and mitochondrial proteins using the genes from the respective terms of BioPlanet and GO Biological Processes databases of the Enrichr web server^22^ (http://amp.pharm.mssm.edu/Enrichr/).

For comparison with published vascular proteome datasets, proteins identified in the ECM and cellular fractions in ≥ two replicates were compared to either the list of 5042 human proteins from retinal endothelial cells (ECs) (reported with FDR ≤ 0.01^23^) or with the list of 653 mouse vascular proteins identified in ≥ 2 replicates.^16^

For the construction of protein-protein interactions (PPI) network we used human proteins identified in at least 2 replicates of the ECM-enriched fraction of HpH fractionated samples. To identify proteins which might interact with the ECM we used a 3-step filtering approach. Firstly, we identified ECM-enriched proteins with potential ECM location or function, based on the available annotation, using databases of the Enrichr web server (GO cellular component, GO molecular function and Jensen compartment) and GeneCards online database. Secondly, to narrow down the list of potential ECM interactors, we compared our list of ECM-enriched proteins with a list of 5137 potentially secreted human proteins, which were identified using computational prediction algorithms.^24^ And lastly, we used the web servers SignalP 4^25^ and Phobius^26^ to further re-assess the list of potential ECM-interactors for the presence of secreted or transmembrane domains.

ECM PPI networks were constructed using StringApp in Cytoscape,^27^ by importing networks from STRING database.^28^ We used networks with high confidence interactions, omitting proteins disconnected from the main network.

To analyse the potential relevance of the ECM PPI network to cerebrovascular disease we next overlaid the ECM PPI network with stroke, vascular dementia and cerebral SVD-associated genes, which were reported in published GWAS studies with P value ≤ 1x10^−7^.^9,29–32^

### Gene enrichment analyses with the open target platform

As an additional approach, genes that are likely to be causal in small vessel disease, stroke, Parkinson’s disease and Alzheimer’s disease were identified using the ‘Variant-to-Gene’ pipeline in the Open Target Platform. Briefly, this pipeline takes into consideration all genetics associations with P < 5e^−8^ in the GWAS Catalogue as well as UK biobank summary statistics. To map genetic variants to genes, lead variants are first used to identify tag variants through (i) statistical fine mapping based on summary statistics or (ii) linkage disequilibrium expansion. Subsequently, these tag variants are integrated with higher order genomic information such as quantitative trait loci (expression QTLs,^33^ and protein QTLs,^34^), chromatin interactions (promoter capture Hi-C,^35^), in silico functional predictions^36^ and distance from the canonical transcript start site^37^ to generate variant-gene scores. Finally, aggregation of variant-genes scores is carried out to identify likely causal genes for the disease of interest.

Enrichment analyses for candidate causal genes in the human cerebrovascular proteome and interacting proteins, were carried out using a hypergeometric test sampling at random (without replacement) from the background containing annotated and non-annotated genes. We also identified pathways that were significantly enriched among disease causal genes, which were overrepresented in the brain cerebrovascular ECM, using the Reactome Knowledgebase.^38^ This was based on pathways whose associate genes were enriched among any overrepresented disease causal gene using the Fisher’s exact test. All matrisome genes were used as the background and the minimum number of overlaps was set to 3. The P values were adjusted for multiple testing by controlling the false discovery rate (FDR). All enrichment analyses were carried using the *xEnricher* functions in the R package ‘XGR’ (version 1.1.4).

### Other statistical analysis

Western blot data were analysed using a non-parametric Mann Whitney U test and a P value below 0.05 was considered to be significant. For proteomics analysis peptide and protein identifications are reported by MaxQuant using the protein parsimony principles^39^ and results were filtered to a 1% FDR. At least one unique peptide was required to identify a protein group. Protein quantification was performed with the MaxLFQ algorithm within MaxQuant.^40^ The intensity and LFQ intensity columns of the Maxquant output were used for protein identification and quantification, respectively. The data were analysed in three samples (biological replicates) derived from three individual mice or three patients.

Differential protein analysis was done using all proteins, which were quantified in both ECM-enriched and cellular fractions in at least two replicates, with at least two unique peptides. The analysis of the differences in protein levels between the ECM-enriched and cellular fractions was done using limma package in R. We used an empirical Bayes method for two group comparison with the moderated t-test of the eb.fit function output.^41^ The results were visualized on volcano plots by plotting the false discovery rate (FDR) adjusted P values for multiple-hypothesis testing with the Benjamini–Hochberg method, with significance level p ≤ 0.05 and absolute value of log2 fold changes, log2fc ≥ 1. Additionally, we plotted on the volcano plots the ECM markers quantified in the ECM-enriched fraction in ≥ two replicates, with ≥ two unique peptides, but present at zero levels in all 3 replicates of the cellular fraction. The significance of the enrichment of identified proteins in the vascular, adhesion and disease proteins were calculated using the hypergeometric test.

## Results

### Cerebral vessel and vascular ECM enrichment

**Figure 1.**
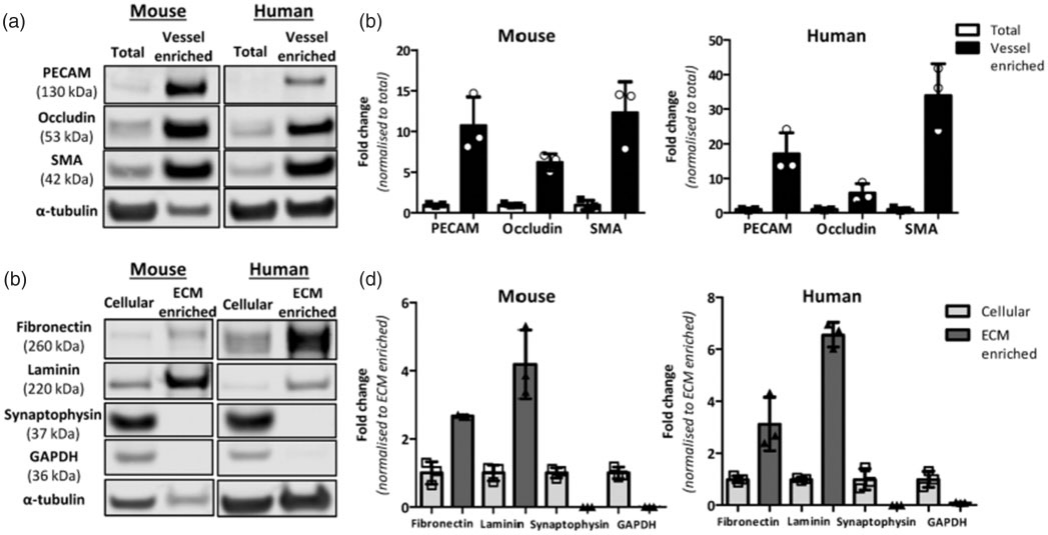
Cerebrovascular enrichment, extracellular matrix enrichment and validation in mouse and human brain. a. vessel-enriched fraction was generated from a total brain homogenate in mouse and human brain and validated by increased levels of vascular-related proteins; smooth muscle actin (SMA), (PECAM (endothelial cells) and occludin (tight junction protein) compared to total brain homogenates (antibodies details are in Supplementary Table S1). b. Measurement of protein levels in mouse and human (n = 3) total and vessel-enriched fractions highlights the marked increase in these proteins in the vessel-enriched fractions compared to total brain. c. Following vessel-enrichment, subsequent fractionation generated cellular and ECM-enriched fractions. d. ECM-enrichment was validated by increased levels of ECM-associated proteins (laminin, fibronectin) and reduced levels of synaptic and cytoplasmic proteins (synaptophysin, GAPDH) compared to cellular fractions. α-tubulin is shown as the loading control (a,c) and to which protein bands are normalised and then expressed as fold change to total protein (b) or ECM enriched (d).

Levels of cerebrovascular proteins were measured by western blotting in vessel-enriched fractions and total brain samples to confirm successful vessel enrichment. The selection of protein markers was based on their association with known cellular compartments of cerebral vessels including markers of vascular smooth muscle (SMA), endothelial cells (PECAM), and tight junctions (occludin). The data demonstrate that vascular proteins in the vessel-enriched fraction are markedly enriched: with a 12- and 34-fold increase for SMA in mouse and human tissues respectively (P < 0.05), an 11- and 17-fold increase for PECAM in mouse and human tissues respectively (P < 0.05) and a 6-fold increase for occludin in mouse and human samples (P < 0.05) (Figures 1(a) and (b), S1).

Using these vessel-enriched fractions from brain tissue we then developed a fractionation approach to collect vessel-associated ECM proteins. Western blotting confirmed enrichment of ECM proteins (3-fold increase for fibronectin in mouse and human samples, P < 0.05; 4- and 7-fold increase for laminin in mouse and human samples respectively, P < 0.05). The efficiency of the ECM enrichment was further confirmed by measurement of synaptic (synaptophysin) and cytoplasmic (GAPDH) proteins. There was a profound reduction in the levels of these proteins which were barely detectable in the ECM-enriched fractions in both mouse and human tissues (P < 0.05, Figures 1(c) and (d), S2).

**Figure 2.**
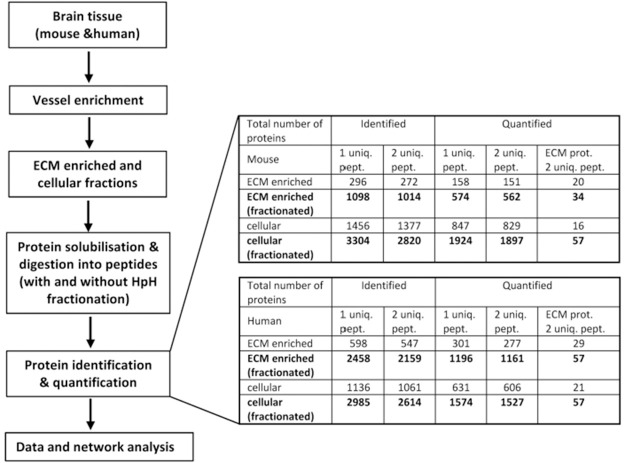
Proteomic workflow, numbers of proteins identified and quantified. Workflow, left, shows steps by which mouse and human vessel enriched brain tissue is then ECM enriched and cellular extracts generated. Subsequent to this, proteins are identified and quantified with and without HpH fractionation using MS and undergo downstream analysis. The numbers shown on right are based on 2 biological replicates and shown for ECM-enriched and cellular fractions, described in “Extracellular matrix enrichment” section of Materials and Methods. MS proteins were identified and quantified using MaxQuant as described in Materials and Methods. The numbers calculated for MS samples with or without pre-fractionation are shown by bold and normal fonts, respectively.

Following vascular ECM enrichment, samples were analysed by mass spectrometry (MS) with and without high-pH reversed-phase (HpH) fractionation to determine how much additional depth is obtained from the fractionated sample. The proteomic workflow along with a summary of results highlighting the total number of proteins identified and quantified based on one or two unique peptides is presented in Figure 2. HpH fractionation of both ECM-enriched and cellular fractions in mouse and human brain markedly increased the number of identified proteins. In particular, in the ECM-enriched fraction, fractionation resulted in a 3- and 3.2-fold increase in the number of identified proteins for mouse and human samples, respectively (Figure S3). The protein abundances were linear between fractionated and non-fractionated proteins, demonstrating that HpH fractionation did not introduce any bias in protein level measurements Figure S4). The protein levels also clustered together on MA (“minus over average”) plots of fractionated and non-fractionated samples, with no outlier proteins (Figure S5).

Comparing the identified proteins in the vascular ECM-enriched and cellular fractions with previously published vascular proteome datasets indicated that most of our proteins were present in these datasets. In particular, more than 73% of human proteins identified in the ECM-enriched fraction and more than 75% in the cellular fraction were present in the vascular retinal proteome, consisting of 5042 endothelial cell (EC) proteins^23^; enrichment P value < 2.2·10^−16^). The majority of the identified vascular proteins (87% in ECM fraction) were common between the ECM-enriched and cellular fractions (Supplementary Table S2). Moreover, more than 90% of previously identified 653 vascular proteins from mouse brain^16^ was present in either ECM-enriched or cellular fractions of our mouse samples (Supplementary Table S2), further confirming the vascular enrichment.

### Identification of cerebrovascular matrisome related proteins

Since HpH fractionation substantially increased the yield of proteins, this approach was subsequently used to identify and quantify proteins in both the ECM-enriched and cellular fractions of mouse and human vasculature from three biological replicates. Based on data acquired on two replicates, MS identified 575 proteins in the ECM-enriched samples and 2820 proteins in the soluble or cellular fraction from mouse samples, and 1168 proteins in the ECM-enriched samples and 2614 in the soluble or cellular fraction from human tissues, (Figure S6; Supplementary Table S3). While a number of proteins found in the ECM-enriched samples were also found in the cellular fraction, many were detected at significantly different levels, as demonstrated by differential expression analysis below.

**Figure 3.**
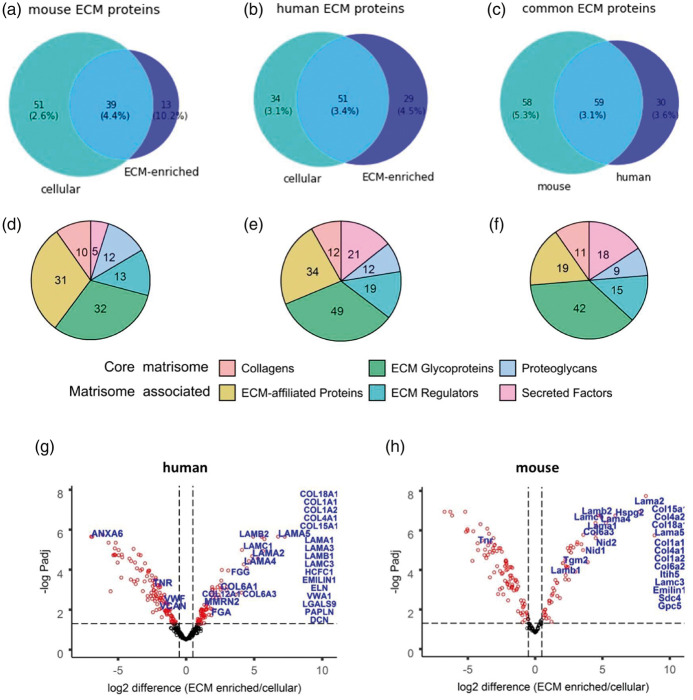
Protein classes in brain vascular ECM and cellular fractions. a–f. Number of ECM proteins annotated with MatrisomeDB. Venn diagrams correspond to the mouse (a) or human (b) ECM proteins identified in ECM-enriched and cellular fractions in at least 2 biological replicates. c. The overlap between orthologous mouse and human ECM proteins. (d–f) The pie charts show total numbers of identified ECM proteins grouped by matrisome categories corresponding to a–c. Two main categories are core matrisome and matrisome associated protein, each subdivided into 3 subgroups (collagens, ECM glycoproteins, proteoglycans and ECM affiliated, ECM regulators and secreted factors, respectively^14^). g,h. Volcano plots for proteins differentially expressed between ECM-enriched and cellular fractions in HpH fractionated samples. Proteins quantified in at least 2 biological replicates were used for this analysis. Proteins with significantly differential expression (FDR ≤ 0.05) are marked in blue. ECM proteins quantified exclusively in the ECM fraction are listed on the right.

The previously published matrisome list was used to annotate known ECM and ECM-associated proteins found in our samples (Figure 2 ^20,21^). We found that a total of 103 out of 2890 of the proteins identified in our mouse samples were annotated as matrisome proteins and 114 out of 2952 in human samples (Figure 3(a) and (b); Supplementary Table S4). More specifically, in the ECM-enriched fraction, 52 out of 575 proteins detected in the ECM-enriched samples from mouse tissues and 80 of the 1168 proteins detected in the ECM-enriched samples from human tissues were annotated as matrisome proteins (Figure 3(a) and (b), Table 1). The cerebrovascular ECM proteins include 10 mouse and 11 human collagens; 26 mouse and 36 human glycoproteins; and 5 mouse and 6 human proteoglycans. Notably, a substantial number of identified ECM proteins (19 for mouse and 20 for human) were basement membrane proteins (Table 1^14^). Furthermore, 59 of the identified ECM proteins were shared between species (encoded by orthologous genes; Figure 3(c)). Approximately half of them (52-54% in mouse and human) were core matrisome proteins, comprised mostly of glycoproteins, with the most abundant ECM-enriched proteins being fibronectins, collagens, laminins and nidogens, of which the last three are core basement membrane components (Figure 3(d) to (f); Supplementary Table S4). The largest sub-group of the remainder were ECM-affiliated proteins, such as lectins or annexins - known secreted proteins that associate with the solid-phase.

**Table 1.** Matrisome proteins identified in ECM-enriched fraction of mouse and human samples in at least 2 biological replicates. Matrisome categories of the core matrisome and matrisome-associated groups are indicated by the colours. Averaged intensities are shown together with the gene names of the identified proteins. Gene encoding basement membrane proteins are shown by bold.

Core matrisome				Core matrisome			
Mouse		Human		Mouse		Human	
Gene	Intensity	Gene	Intensity	Gene	Intensity	Gene	Intensity
***Collagens***				***ECM glycoproteins***			
**Col4a2**	8.7E + 06	**COL4A2**	2.0E + 07			DSP	9.4E + 04
Col1a1	4.7E + 06	**COL6A3**	9.5E + 06			EFEMP1	8.2E + 04
**Col6a3**	2.1E + 06	COL1A2	6.6E + 06			LGI3	6.6E + 04
**Col6a1**	1.3E + 06	**COL6A1**	3.0E + 06			TGFBI	3.5E + 04
**Col18a1**	1.2E + 06	**COL4A1**	2.5E + 06			PXN	3.0E + 04
Col1a2	5.9E + 05	COL1A1	2.3E + 06			LTBP1	2.3E + 04
**Col4a1**	4.8E + 05	**COL18A1**	2.0E + 06			MATN2	2.2E + 04
**Col6a2**	4.8E + 05	COL12A1	9.2E + 05			EDIL3	2.6E + 03
Col12a1	2.1E + 05	**COL15A1**	4.3E + 05	***Proteoglycans***			
**Col15a1**	1.4E + 05	COL14A1	1.7E + 05	Dcn	5.2E + 04	**HSPG2**	2.2E + 07
		**COL4A6**	6.8E + 04	Bgn	3.8E + 04	HAPLN2	5.5E + 05
***ECM glycoproteins***				Prelp	2.5E + 04	BGN	3.9E + 05
**Lamc1**	3.2E + 07	**LAMB2**	4.4E + 07	Hapln1	2.1E + 04	DCN	3.8E + 05
**Lamb2**	3.2E + 07	**LAMC1**	3.2E + 07	Ncan	1.2E + 04	VCAN	8.1E + 04
**Lama5**	1.1E + 07	**LAMA5**	1.5E + 07			PRELP	6.1E + 04
**Nid1**	1.0E + 07	**NID1**	1.3E + 07	**Matrisome-associated**			
**Agrn**	7.8E + 06	**LAMA2**	1.3E + 07	Mouse		Human	
Tinagl1	5.3E + 06	**AGRN**	1.2E + 07	Gene	Intensity	Gene	Intensity
**Nid2**	5.2E + 06	FN1	9.1E + 06	***ECM-affiliated proteins***			
**Lama2**	4.4E + 06	**NID2**	8.7E + 06	Gpc5	6.2E + 04	ANXA1	7.8E + 05
**Lama1**	3.3E + 06	TINAGL1	8.5E + 06	Sdc4	5.7E + 04	LGALS9	2.2E + 05
Vwa1	2.5E + 06	FGG	7.2E + 06	Gpc4	2.6E + 04	LGALS1	4.7E + 04
**Lama4**	2.0E + 06	FGA	2.1E + 06	Gpc1	9.2E + 03	ANXA6	3.6E + 04
**Lamb1**	1.4E + 06	**LAMA1**	1.8E + 06	Anxa2	8.1E + 03	ANXA5	3.1E + 04
Vwf	1.4E + 05	**LAMA4**	1.5E + 06			ANXA2	2.7E + 04
**Lamc3**	9.8E + 04	VWA1	1.2E + 06			ANXA7	6.2E + 03
Tnr	8.5E + 04	EMILIN1	9.7E + 05	***ECM regulators***			
**Lama3**	7.9E + 04	**LAMC3**	8.2E + 05	Itih5	9.2E + 05	TGM2	4.2E + 06
Mmrn2	5.1E + 04	VWA7	7.7E + 05	Tgm2	6.0E + 05	CTSD	1.1E + 06
Ltbp4	4.9E + 04	ELN	7.3E + 05	Gm5409	3.0E + 05	CAP2	3.7E + 05
Emilin1	4.4E + 04	LAMB1	7.0E + 05	Serpinh1	1.1E + 05	SERPINH1	1.1E + 05
Dsp	4.3E + 04	VWF	5.6E + 05			TIMP3	3.2E + 05
Vtn	2.8E + 04	TNXB	4.5E + 05			PLAT	1.1E + 05
**Papln**	2.2E + 04	MMRN2	4.1E + 05			HTRA1	5.7E + 04
Fbln5	1.7E + 04	FBN1	3.5E + 05	***Secreted factors***			
Lgi3	1.6E + 04	TNR	3.1E + 05	Sart1	8.4E + 04	PC	1.9E + 06
Lgi1	1.1E + 04	SBSPON	1.5E + 05			LMNB2	1.0E + 06
Sbspon	5.8E + 03	**LAMA3**	1.5E + 05			MLF2	2.2E + 05
Mfap1	8.2E + 04	**PAPLN**	1.1E + 05			NBN	1.3E + 05
		MFAP1	1.2E + 04			HCFC1	1.2E + 05
						RIF1	1.0E + 05
						S100A8	9.2E + 04
						STK3	8.5E + 04
						ZFP91-CNTF	5.6E + 04
						HCFC2	2.6E + 04
						MIF	7.6E + 03
						EGFL8	4.0E + 03

### Differential protein analysis of cerebrovascular ECM-enriched and cellular fractions

89% of mouse ECM-enriched proteins and 71% of human ECM-enriched proteins were also detected in their counterpart cellular fraction (Supplementary Table S3). To explore the differences in protein levels between the ECM-enriched and cellular fractions, differential expression analysis of the proteins was performed, using 189 mouse and 356 human proteins quantified in both HpH-fractionated samples in at least 2 replicates, with at least 2 unique peptides (Supplementary Table S5).

The respective volcano plots of differentially expressed fractionated proteins (FDR ≤ 0.05, fold change ≥ 2) show that for 180 human proteins their protein levels were significantly different between the ECM-enriched and cellular fractions (Figure 3(g) and (h)). This includes 21 known human ECM proteins (coloured by blue, Figure 3(g) and (h)), such as the key ECM constituents COL6A1, COL6A3, COL12A1, LAMA2, LAMA4, LAMA5, LAMB2 and LAMC1. In addition, another 16 known ECM proteins were present exclusively in the ECM-enriched fraction, such as COL1A1, COL1A2, COL4A1, COL15A1, COL18A1, EMILIN1, LAMA3 and LAMC3 (shown as columns on the right, Figure 3(g) and (h)). In mouse samples, there were 128 differentially expressed proteins, with 13 of them being known ECM proteins. Another 12 ECM proteins were exclusively present in the ECM-enriched fraction (Figure 3(g) and (h)). As expected, there was a substantial reduction in the numbers of proteins that were differentially expressed between these fractions when samples were not HpH fractionated prior to MS, with 39 and 28 proteins in human and mouse, respectively (Figure S7, Supplementary Table S5).

### Identification of cerebrovascular proteins that potentially interact with the ECM

**Figure 4.**
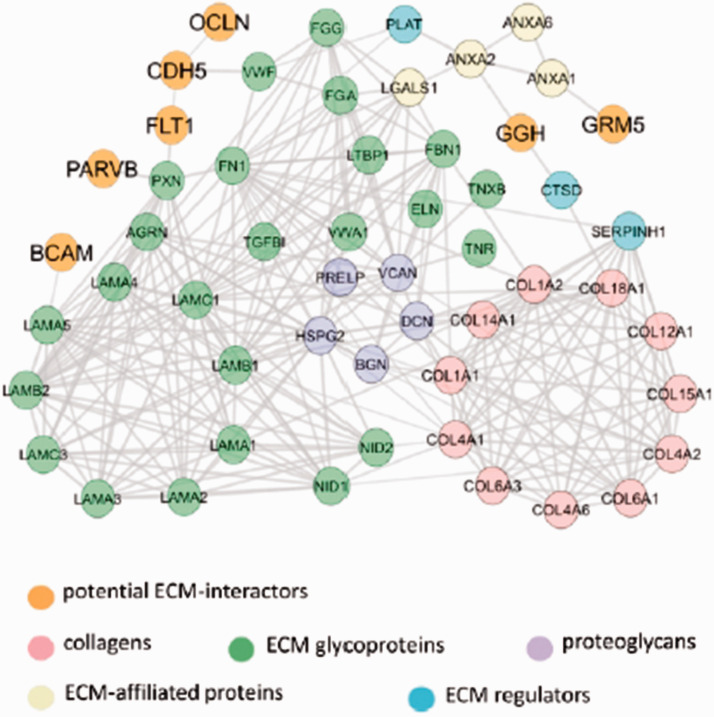
Network of interactions between known ECM proteins and predicted ECM-interacting proteins. The network comprises proteins either ≥ 2-fold enriched or exclusively present in the ECM fraction. 7 predicted ECM-interacting proteins are not annotated as part of the matrisome and predicted to have signal peptides or extracellular domains. Previously established ECM proteins identified by Matrisome DB, as well as predicted ECM-interactors are coloured as indicated. The networks were built using the STRING database with a high confidence of interactions.

To identify potential ECM-interacting proteins not previously annotated as matrisome proteins, we used all proteins identified with ≥ 2-fold higher average levels in ECM-enriched compared to cellular fraction or uniquely expressed in at least two replicates in the ECM-enriched fraction. 337 proteins (Supplementary Table S6) were detected in the ECM-enriched fraction of human samples, but not annotated as being part of the matrisome. Based on previously annotated protein subcellular location and function, 50 of these proteins were identified as potential candidates for interaction with brain ECM (Supplementary Table S6). Comparison of the identified 50 proteins with the reported list of 2412 proteins involved in cell adhesion (so-called “adhesome”^42^) confirmed significant enrichment in adhesion proteins (P value < 1.2·10^−6^). We next assessed the 50 ECM-enriched proteins for the presence of secreted or transmembrane domains^24–26^ and identified 11 proteins that were putatively secreted or had signal peptides or transmembrane domains (Supplementary Table S7). Network analysis with STRING was undertaken on these 11 proteins and the 80 Matrisome proteins identified in the ECM-enriched fraction. The resulting PPI network revealed that 7 of these predicted ECM-interacting proteins (coloured in orange on Figure 4) were connected to known ECM proteins.

### Genes relevant to cerebrovascular disease are enriched in the brain ECM-interacting proteins

**Figure 5.**
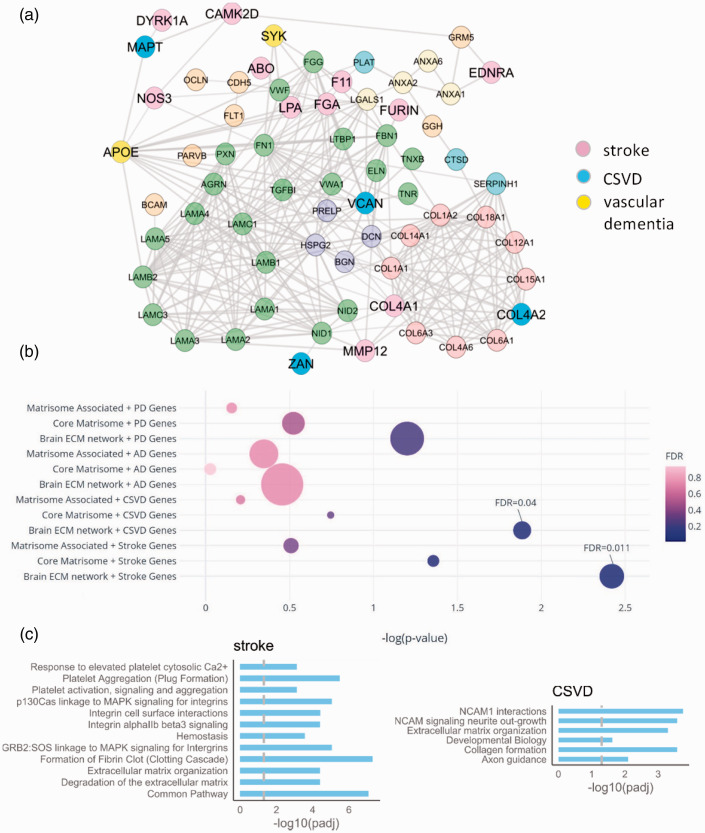
Disease genes and pathways in the human brain vascular ECM network. a. PPI network of cerebrovascular disease-associated genes (stroke, SVD and vascular dementia) connected to the ECM network of Figure 4. b. The overlap between brain disease genes (based on Open Target Platform) and ECM-related gene sets. PD = Parkinson’s disease, AD= Alzheimer’s disease, CSVD= cerebral small vessel disease, Stroke = ischemic stroke, ‘brain ECM network’ is the ECM network with interacting genes on STRING, ‘core matrisome’ and ‘matrisome associated’ are matrisome genes.^14^ The bubble size represents the number of overlapping genes. Significant results from brain ECM network are labelled. c. Enriched pathways amongst stroke and CSVD genes, overlapping with the brain ECM network. The vertical line is the FDR cut-off at 0.05.

To explore whether the constructed ECM network of Figure 4 has relevance to cerebrovascular disease, we overlaid the network with the disease-associated genes reported in published GWAS studies on stroke,^29,30^ vascular dementia (VaD^31,32^) and cerebral SVD.^9^ We found that this network was significantly enriched (P value 7·10^−4^) with the cerebrovascular disease-associated genes (Figure 5(a); Supplementary Table S7). There were 17 disease genes directly connected to the ECM network, with several genes (*COL4A1, COL4A2, VCAN, FGA, MMP12*) representing the main hubs across the brain vascular matrisome. Interestingly, APOE, a key regulator of BBB and cerebrovascular integrity^43^ and one of the biggest genetic risk factors for dementia, is highly connected to this network.

As a complementary approach, we compiled a list of likely causal genes in ischemic stroke, cerebral SVD, Alzheimer’s disease and Parkinson’s disease based on the ‘Variant-to-Gene’ pipeline within the Open Target Platform (Supplementary Table S8). Notably, we found a significant enrichment of genes relevant to cerebrovascular disease among genes that code for ECM and interacting proteins (stroke: number of overlapping genes = 21, FDR = 0.011; SVD: number of overlapping genes = 11, FDR = 0.04) (Figure 5(b) and Supplementary Table S8). Interestingly, we did not find any enrichment of genes relevant to Alzheimer’s disease (number of overlapping genes = 57, FDR= 0.68) and Parkinson’s disease (number of overlapping genes = 37, FDR= 0.19); supporting a possible specific role for ECM and interacting proteins in cerebrovascular disease.

The 21 ischemic stroke genes overlapping with genes that code for ECM-related proteins (Supplementary Table S8) included *COL3A*, *MMP1*, *MMP12* and *MMP3*. We next carried out pathway enrichment analyses to provide biological insight into this overlap, using the Reactome Knowledgebase.^38^ We identified several key pathways that were significantly enriched (Figure 5(c) and Supplementary Table S8). These included formation of fibrin clot (fold change= 18.7, 5.00E-08), integrin cell surface interactions (fold change= 9.25, FDR = 0.000038) and degradation of the extracellular matrix (fold change = 11.7, FDR= 0.000039). Furthermore, the 11 enriched cerebral small vessel genes overlapped with genes that code for brain ECM-related constituents included *APOE*, *COL4A*, *COL4A2* and *COLGALT1* and were significantly enriched in several informative pathways (Figure 5(c) and Supplementary Table S8). The key pathways included NCAM1 interactions (fold change = 11.5, FDR = 0.00018) and Collagen formation (fold change = 9.32, FDR = 0.00027).

## Discussion

ECM deregulation is emerging as a common contributory, and sometimes causal, mechanism in cerebrovascular disease.^2,3^ However, very little is known about the composition of the cerebrovascular ECM due to its complexity and insolubility which has significantly hindered its biochemical and molecular analysis. Here we report an efficient method to extract and analyse cerebrovascular ECM proteins which can be used as a platform to establish for the first time, the proteome of mouse and human cerebrovascular ECM in normal brain tissues. We then used network analysis to explore the connection between the identified ECM proteins and proteins which are known to be involved in cerebrovascular disease. Interestingly, we found that genes relevant to cerebrovascular disease including ischemic stroke and cerebral SVD are exclusively overrepresented among genes that code for brain ECM and interacting proteins.

The method that we outline is based on cerebral vessel extraction, separation of cellular and ECM-enriched fractions based on their solubility, followed by off-line high-pH reversed-phase fractionation to increase protein detection by MS. Our approach successfully enriched for ECM proteins and the HpH fractionation step results in ∼3.5–3.9 fold increase in proteome depth, which highlights the complexity of pre-fractionated samples. The ECM-enriched proteins include collagens I and VI, main constituents of the brain ECM, with considerable enrichment of major basement membrane proteins collagen IV and laminins (Figure 3(g) and (h)). Furthermore, many ECM proteins that we discovered as constituting the human brain vascular matrisome are annotated matrisome proteins (Naba et al., 2012 and 2016). There was also significant overlap with proteins identified from the retinal vascular proteome (Smith *et al.*, 2018), mouse cerebrovascular proteome (Searcy *et al.*, 2014) and the adhesome (Horton *et al.*, 2015).

A large proportion of proteins identified in the ECM-enriched fractions, but not previously annotated as being part of the matrisome, include intracellular insoluble proteins, such as chromatin-bound and cytoskeleton components. After removing these likely contaminants, the remainder may represent putative secreted factors, transmembrane proteins or adhesion molecules based upon our enrichment analysis. Importantly we found 7 proteins, present in our samples, are connected to the network of matrisome proteins. These include BCAM and PARVB, which are involved in cell adhesion and migration. The network also includes VE-Cadherin (CDH5) and vascular endothelial growth factor VEGFR1 (FLT1), both of which play an important role in angiogenesis and vasculogenesis. Further studies of these proteins might uncover their potential involvement in regulating normal and pathological states of the vascular ECM.

We found that many proteins, identified by MS, were present in both ECM-enriched and cellular fractions. This is perhaps not unexpected since the cells of the NVGU will generate these proteins for secretion and incorporation into the ECM. It will be interesting to understand from future studies how much of disease associated changes in the matrisome are captured by the soluble fraction given the considerable overlap we found between fractions. Comparisons between these fractions might also give important insights into disease induced changes that exclusively occur outside the cells. Our data also demonstrated a significant overlap between mouse and human brain ECM samples, with 70% of all identified human protein and 66% of human ECM markers present in mouse samples. This gives confidence in the murine models to study disease and other processes that might affect the brain matrisome. It was noted that there were fewer proteins identified in the mouse compared to the human ECM proteome (Table 1). This may be due to technical reasons related to differences in the initial weight of the tissues used to generate vascular and subsequent ECM enriched fractions. The starting material of the mouse brain tissue (approx. 0.2 g) was smaller than the human brain tissue (0.3-0.4g), potentially leading to a greater enrichment of the vasculature and then ECM in human compared to mouse samples (Figure 1).

There are several potential limitations of our study. Firstly, our preparations were done using the whole brain vasculature, consisting of different vascular segments. Therefore, our study can be considered as a first, global characterization of the cerebrovascular ECM proteome. Further developments of the vascular dissection methods might allow analysis of more specific vascular regions. Secondly, our cellular fractions represent a mixture of different cell types that are present in the cerebrovasculature. Therefore, the proteins identified in the cellular fractions originate from different cell types. Thirdly, similar to previously used methods of ECM enrichment, our method might extract contaminating insoluble constituents from nucleus, mitochondria and other intracellular compartments. While we filtered out those potentially contaminating proteins from the ECM fractions, our data might still include some proportion of poorly annotated proteins, originating from these insoluble constituents. Finally future studies are required to assess directly the effect of disorders such as SVD or vascular dementia have on the vascular ECM. Our studies have served to highlight that proteins relevant to these diseases are present in the network and it will be interesting to investigate whether these are dysregulated in pathological states.

To explore the potential pathogenicity of the ECM, we built the PPI network linking the ECM proteins, identified in our samples, to the proteins encoded by candidate GWAS genes associated with cerebrovascular diseases (Figure 5(a)). In addition, we used Open Target Platform to further evaluate the relevance of our ECM network to neurologic disease. Both approaches demonstrate the significant overlap between the ECM-related genes and cerebrovascular disease genes. The differences in the overlapping gene sets (Figure 5(a), Table S8) were mainly due to different strategies to prioritize the genes (based on GWAS alone, or in combination with other types of relevant information, such as QTLs, chromatin interactions and in silico functional predictions). Interestingly, the overlap between the ECM-related genes and genes associated with general neurologic diseases (Parkinson’s or Alzheimer’s disease) was not significant. Therefore, by using proteomics of ECM-enriched healthy brain tissues and network analysis we found that the brain ECM network is specifically connected to cerebrovascular disease genes.

Recent studies have also implicated cerebrovascular ECM proteins in mechanisms of other relevant pathological processes including paediatric stroke,^44^ hypertension,^45^ and disruption of the blood brain barrier (BBB).^2,46^ It has been shown that increased levels of collagens (*e.g.*, COL4A1) might be conducive to stroke by increasing the stiffness of cerebral arteries, leading to hypoperfusion and hypertension.^45^ Cerebral hypoperfusion is also known to be associated with microvascular inflammation including dysfunction of endothelial cells and activation of microglia which release metalloproteinases leading to BBB disruption in SVD and other cerebrovascular diseases.^2^ In line with these studies our ECM-related disease network includes genes involved in structural stability (*e.g.*, collagens), adhesion, ECM remodelling (*e.g.*, MMP1, MMP3, MMP12), angiogenesis and activation of CNS inflammation. Interestingly, APOE is highly connected to this network, including to metalloproteinases and fibrinogens and previous studies have indicated APOE is an important regulator of cerebrovascular integrity,^43,47^ highlighting the potential importance of this disease gene in remodelling of the brain vascular matrisome.

It is worth noting that although the present study did not investigate gender effects, some gender-related differences in the microvascular proteome^48^ might contribute to cerebrovascular pathology. These gender effects are largely related to mitochondrial metabolism and its ECM regulators, such as annexins,^48^ which might be explored in future studies. Another important factor to be considered for future disease-related studies is age, a major risk factor for SVD and cerebrovascular disease, affecting disease severity and outcome, including post-stroke remodelling of the brain tissues.^49^ We previously demonstrated an age-dependent impact on the cerebrovascular proteome in mice.^16^ Age also modifies axonal regeneration, a process depending on cellular interactions with the ECM, migration and angiogenesis and mediated by multiple signalling molecules, such as BDNF, VEGF and TGF-β.^49^ In support of such a role, our pathway analysis of the ECM-related disease genes demonstrated enrichment with processes such as neurite growth, axon guidance, integrin signalling and ECM degradation.

In conclusion, we present a refined method for efficient enrichment of the mouse and human brain matrisome associated with the cerebrovasculature. The human brain vascular matrisome and its interacting proteins are enriched in proteins implicated in cerebrovascular disease, suggesting that the brain matrisome has a key role in pathogenic mechanisms. Our method of cerebrovascular ECM enrichment can be further applied to various pathological conditions and our data represent a useful resource for exploration of the cerebrovascular matrisome.

## Supplemental Material

sj-pdf-1-jcb-10.1177_0271678X211004307 - Supplemental material for Global proteomic analysis of extracellular matrix in mouse and human brain highlights relevance to cerebrovascular disease Click here for additional data file. Supplemental material, sj-pdf-1-jcb-10.1177_0271678X211004307 for Global proteomic analysis of extracellular matrix in mouse and human brain highlights relevance to cerebrovascular disease by Alexandra Pokhilko, Gaia Brezzo, Lahiru Handunnetthi, Raphael Heilig, Rachel Lennon, Colin Smith, Stuart M Allan, Alessandra Granata, Sanjay Sinha, Tao Wang, Hugh S Markus, Alexandra Naba, Roman Fischer, Tom Van Agtmael, Karen Horsburgh and M Zameel Cader in Journal of Cerebral Blood Flow & Metabolism

sj-xlsx-2-jcb-10.1177_0271678X211004307 - Supplemental material for Global proteomic analysis of extracellular matrix in mouse and human brain highlights relevance to cerebrovascular disease Click here for additional data file. Supplemental material, sj-xlsx-2-jcb-10.1177_0271678X211004307 for Global proteomic analysis of extracellular matrix in mouse and human brain highlights relevance to cerebrovascular disease by Alexandra Pokhilko, Gaia Brezzo, Lahiru Handunnetthi, Raphael Heilig, Rachel Lennon, Colin Smith, Stuart M Allan, Alessandra Granata, Sanjay Sinha, Tao Wang, Hugh S Markus, Alexandra Naba, Roman Fischer, Tom Van Agtmael, Karen Horsburgh and M Zameel Cader in Journal of Cerebral Blood Flow & Metabolism

sj-xlsx-3-jcb-10.1177_0271678X211004307 - Supplemental material for Global proteomic analysis of extracellular matrix in mouse and human brain highlights relevance to cerebrovascular disease Click here for additional data file. Supplemental material, sj-xlsx-3-jcb-10.1177_0271678X211004307 for Global proteomic analysis of extracellular matrix in mouse and human brain highlights relevance to cerebrovascular disease by Alexandra Pokhilko, Gaia Brezzo, Lahiru Handunnetthi, Raphael Heilig, Rachel Lennon, Colin Smith, Stuart M Allan, Alessandra Granata, Sanjay Sinha, Tao Wang, Hugh S Markus, Alexandra Naba, Roman Fischer, Tom Van Agtmael, Karen Horsburgh and M Zameel Cader in Journal of Cerebral Blood Flow & Metabolism

sj-xlsx-4-jcb-10.1177_0271678X211004307 - Supplemental material for Global proteomic analysis of extracellular matrix in mouse and human brain highlights relevance to cerebrovascular disease Click here for additional data file. Supplemental material, sj-xlsx-4-jcb-10.1177_0271678X211004307 for Global proteomic analysis of extracellular matrix in mouse and human brain highlights relevance to cerebrovascular disease by Alexandra Pokhilko, Gaia Brezzo, Lahiru Handunnetthi, Raphael Heilig, Rachel Lennon, Colin Smith, Stuart M Allan, Alessandra Granata, Sanjay Sinha, Tao Wang, Hugh S Markus, Alexandra Naba, Roman Fischer, Tom Van Agtmael, Karen Horsburgh and M Zameel Cader in Journal of Cerebral Blood Flow & Metabolism

sj-xlsx-5-jcb-10.1177_0271678X211004307 - Supplemental material for Global proteomic analysis of extracellular matrix in mouse and human brain highlights relevance to cerebrovascular disease Click here for additional data file. Supplemental material, sj-xlsx-5-jcb-10.1177_0271678X211004307 for Global proteomic analysis of extracellular matrix in mouse and human brain highlights relevance to cerebrovascular disease by Alexandra Pokhilko, Gaia Brezzo, Lahiru Handunnetthi, Raphael Heilig, Rachel Lennon, Colin Smith, Stuart M Allan, Alessandra Granata, Sanjay Sinha, Tao Wang, Hugh S Markus, Alexandra Naba, Roman Fischer, Tom Van Agtmael, Karen Horsburgh and M Zameel Cader in Journal of Cerebral Blood Flow & Metabolism

sj-pdf-6-jcb-10.1177_0271678X211004307 - Supplemental material for Global proteomic analysis of extracellular matrix in mouse and human brain highlights relevance to cerebrovascular disease Click here for additional data file. Supplemental material, sj-pdf-6-jcb-10.1177_0271678X211004307 for Global proteomic analysis of extracellular matrix in mouse and human brain highlights relevance to cerebrovascular disease by Alexandra Pokhilko, Gaia Brezzo, Lahiru Handunnetthi, Raphael Heilig, Rachel Lennon, Colin Smith, Stuart M Allan, Alessandra Granata, Sanjay Sinha, Tao Wang, Hugh S Markus, Alexandra Naba, Roman Fischer, Tom Van Agtmael, Karen Horsburgh and M Zameel Cader in Journal of Cerebral Blood Flow & Metabolism

sj-pdf-7-jcb-10.1177_0271678X211004307 - Supplemental material for Global proteomic analysis of extracellular matrix in mouse and human brain highlights relevance to cerebrovascular disease Click here for additional data file. Supplemental material, sj-pdf-7-jcb-10.1177_0271678X211004307 for Global proteomic analysis of extracellular matrix in mouse and human brain highlights relevance to cerebrovascular disease by Alexandra Pokhilko, Gaia Brezzo, Lahiru Handunnetthi, Raphael Heilig, Rachel Lennon, Colin Smith, Stuart M Allan, Alessandra Granata, Sanjay Sinha, Tao Wang, Hugh S Markus, Alexandra Naba, Roman Fischer, Tom Van Agtmael, Karen Horsburgh and M Zameel Cader in Journal of Cerebral Blood Flow & Metabolism

sj-pdf-8-jcb-10.1177_0271678X211004307 - Supplemental material for Global proteomic analysis of extracellular matrix in mouse and human brain highlights relevance to cerebrovascular disease Click here for additional data file. Supplemental material, sj-pdf-8-jcb-10.1177_0271678X211004307 for Global proteomic analysis of extracellular matrix in mouse and human brain highlights relevance to cerebrovascular disease by Alexandra Pokhilko, Gaia Brezzo, Lahiru Handunnetthi, Raphael Heilig, Rachel Lennon, Colin Smith, Stuart M Allan, Alessandra Granata, Sanjay Sinha, Tao Wang, Hugh S Markus, Alexandra Naba, Roman Fischer, Tom Van Agtmael, Karen Horsburgh and M Zameel Cader in Journal of Cerebral Blood Flow & Metabolism
