# Supplementary material for: Global proteomic analysis of extracellular matrix in mouse and human brain highlights relevance to cerebrovascular disease
Source: J Cereb Blood Flow Metab. 2021 Mar 17;41(9):2423–38. doi: 10.1177/0271678X211004307 (PMC8392779; doi:10.1177/0271678X211004307)
Supplement: sj-pdf-1-jcb-10.1177_0271678X211004307 - Supplemental material for Global proteomic analysis of extracellular matrix in mouse and human brain highlights relevance to cerebrovascular disease [file sj-pdf-1-jcb-10.1177_0271678X211004307.pdf]

Supplementary Figures

Figures S1 and S2 with full unedited Western blots of Fig. 1 are supplied in a separate file

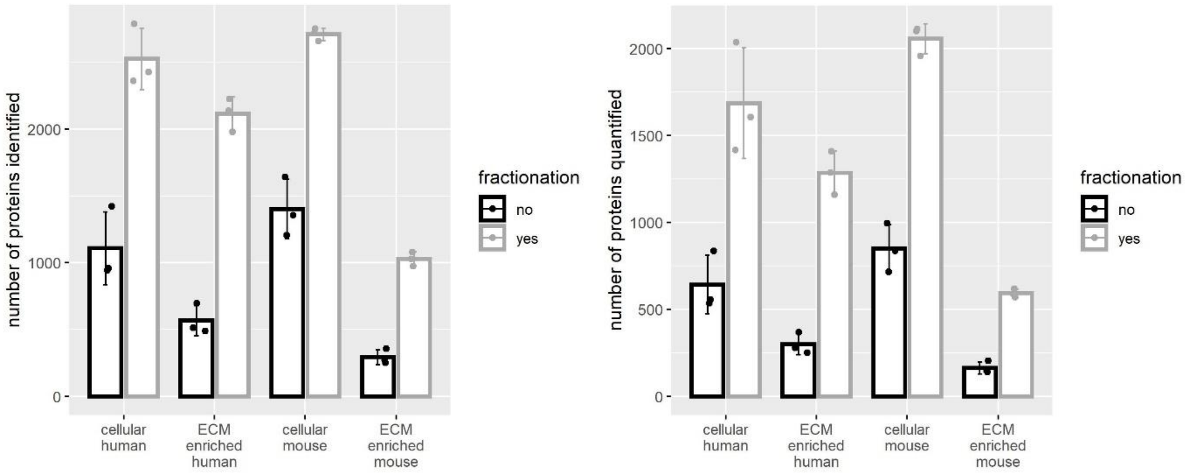

Figure S3. HpH fractionation increases MS yield of proteins quantified and identified in mouse and human ECM-enriched and cellular fractions. Average numbers of proteins identified (left) or quantified (right) using MaxQuant for human or mouse MS samples with (grey) or without (black) HpH fractionation. Error bars show SD based on values in 3 biological replicates, shown by dots. Only proteins identified or quantified in ≥ 2 replicates are counted.

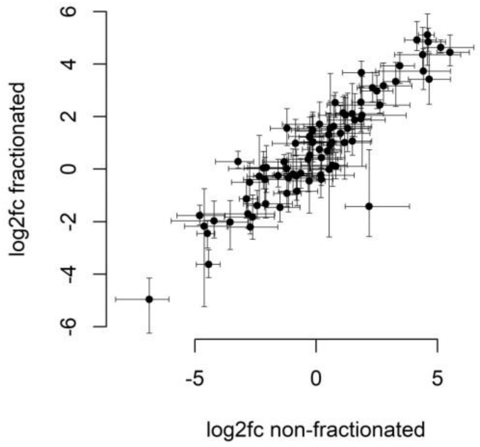

Figure S4. Linear relationship between identified changes in protein levels in MS samples with and without HpH fractionation.

The log2 fold changes in mean protein abundance in ECM relative to cellular fraction for proteins identified in both fractions in 3 biological replicates. Error bars show the range of changes calculated based on minimum and maximum values between the 3 replicates.

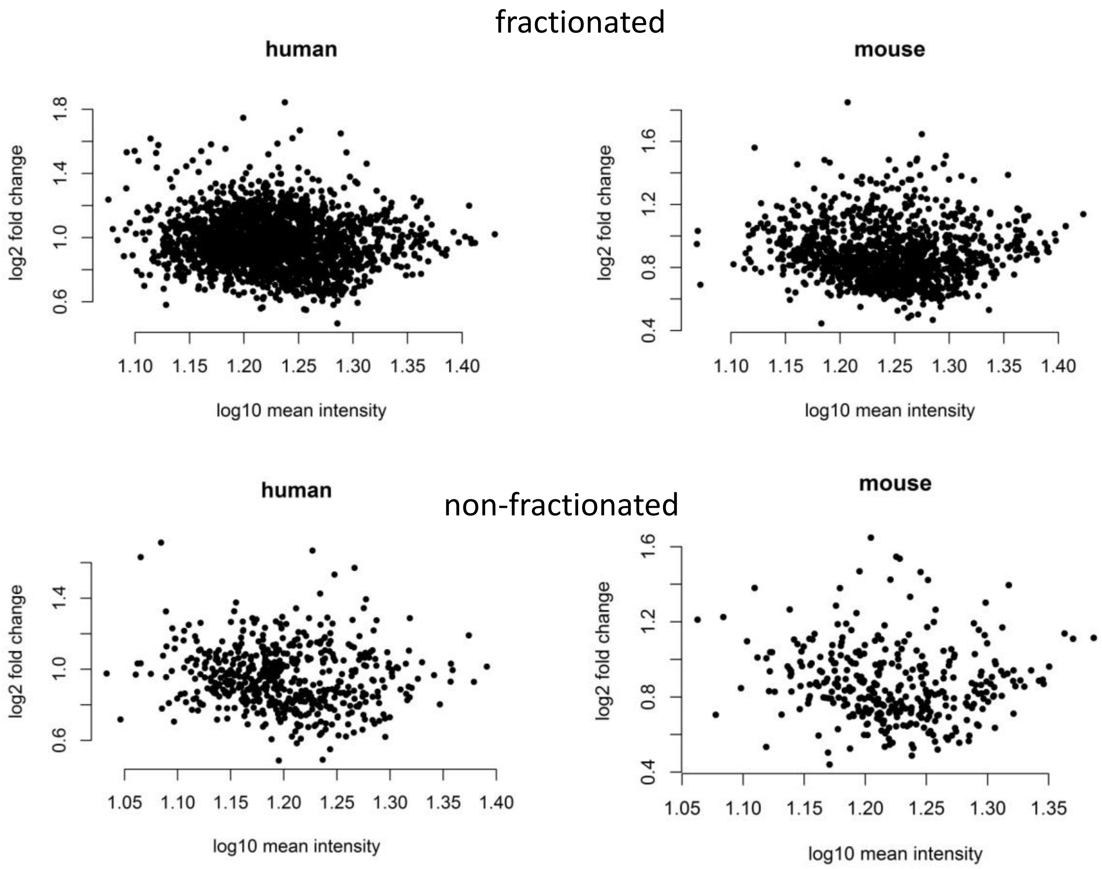

Figure S5. MA plots of changes in protein levels for MS samples with or without HpH fractionation.

The plots show that the protein levels cluster together on MA plots of fractionated and non-fractionated samples, with no outlier proteins. The log2 of fold changes in mean ECM/cellular protein abundance is shown against log10 of the averaged protein abundance. Proteins were identified in at least 2 biological replicates.

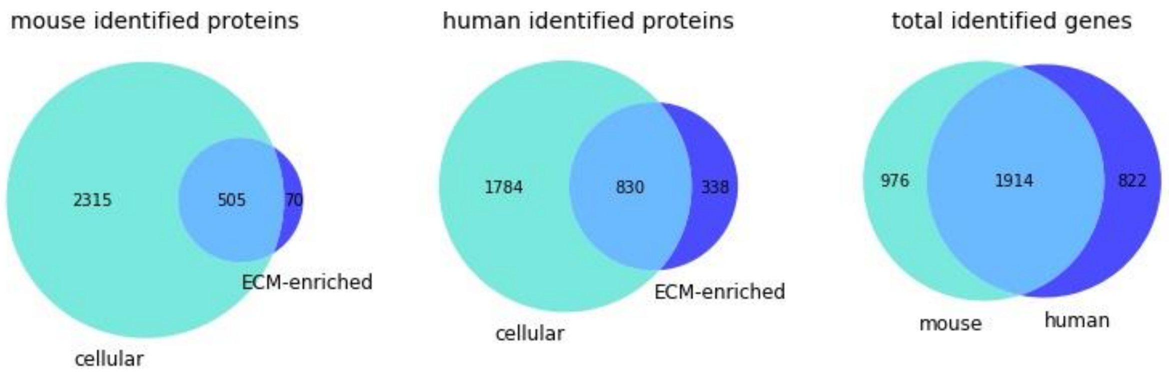

Figure S6. Venn diagrams of the total numbers of identified proteins in ECM-enriched and cellular fractions identified by mass spectrometry with sample HpH fractionation. Numbers shown for mouse (left) or human (middle) proteins identified in at least 2 biological replicates. The overlap between ECM-enriched and cellular fractions is indicated. The Venn diagram on the right shows the overlap between orthologous mouse and human genes, encoding the identified proteins.

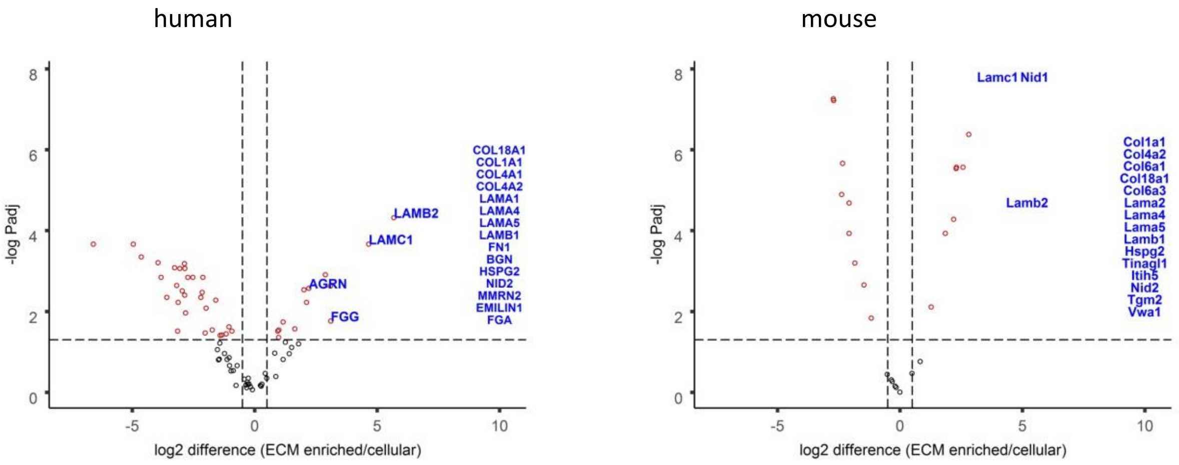

Figure S7. Volcano plots for proteins differentially expressed between ECM-enriched and cellular fractions in MS samples without HpH fractionation.

The proteins quantified in at least 2 biological replicates for HpH fractionated MS samples were used. Proteins with significantly different levels of ECM markers (FDR ≤ 0.05) are marked by blue. ECM markers quantified only in ECM fraction (in at least 2 replicates) are shown as columns on the right.
