# Supplementary material for: Global proteomic analysis of extracellular matrix in mouse and human brain highlights relevance to cerebrovascular disease
Source: J Cereb Blood Flow Metab. 2021 Mar 17;41(9):2423–38. doi: 10.1177/0271678X211004307 (PMC8392779; doi:10.1177/0271678X211004307)
Supplement: sj-pdf-6-jcb-10.1177_0271678X211004307 - Supplemental material for Global proteomic analysis of extracellular matrix in mouse and human brain highlights relevance to cerebrovascular disease [file sj-pdf-6-jcb-10.1177_0271678X211004307.pdf]

Supplementary Tables S1-S2

Table S1. Antibodies used for vessel and ECM enrichment validation

**Antibody**	**Species**	**Supplier**	**Concentration**
SMA	Mouse	Millipore, CBL171	1:500
Occludin	Rabbit	Invitrogen, 711500	1:500
PECAM	Rabbit	Abcam, ab28364	1:500
Laminin	Rabbit	Abcam, ab11575	1:500
Fibronectin	Rabbit	Abcam, ab2413	1:500
GAPDH	Rabbit	Sigma, G8545,	1:20,000
Synaptophysin	Mouse	Sigma, S5768	1:1000
a-tubulin (loading control)	Mouse	Abcam, ab7291	1:5000

Table S2. Vascular proteins identified in ECM-enriched and cellular fractions of mouse and human samples in at least 2 biological replicates. The Table reports 592 mouse proteins identified in either ECM-enriched or cellular fraction, which are present in published mouse cerebrovascular dataset [16]. The Table also reports 1571 human vascular EC proteins [23], identified in both ECM-enriched or cellular fractions. Mouse proteins are shown in the first part of the Table and human proteins - in the second part of the Table. Mouse gene names are in small letters and capitalized and human genes are in capital letters.

Mouse vascular proteins

gene	protein	Protein.names	cellular1	cellular2	cellular3	ECM1	ECM2	ECM3
Aak1	Q3UHJ0	AP2-associated protein kinase 1	1356300	1.34E+06	1003300	1.68E+04	2.60E+04	6.79E+03
Acaa2	Q8BWT1	3-ketoacyl-CoA thiolase, mitochondrial	1997500	2.22E+06	2246200	0.00E+00	1.11E+03	0.00E+00
Acin1	F6RJ39	Apoptotic chromatin condensation inducer in the nucleus	2141200	3.15E+06	3002900	2.05E+06	3.42E+06	2.87E+06
Aco2	Q99KI0	Aconitate hydratase, mitochondrial	26404000	2.24E+07	21578000	2.62E+05	1.94E+05	7.29E+04
Actn1	A1BN54		14350000	1.68E+07	14455000	1.53E+06	1.21E+06	1.12E+06
Actn4	A0A1L1SV25	Alpha-actinin-4	6105200	7.39E+06	6522200	5.83E+05	6.56E+05	4.24E+05
Add1	Q8K232	Alpha-adducin	4602000	3.67E+06	2336300	1.04E+05	4.95E+04	1.07E+05

Add2	Q9QYB8	Beta-adducin	1532500	1.85E+06	1624900	6.31E+04	0.00E+00	0.00E+00
Ahcyl1	Q80SW1	Putative adenosylhomocysteinase 2	4189600	2.75E+06	1264000	5.75E+04	3.98E+03	0.00E+00
Akap5	H3BIV5	A-kinase anchor protein 5	0	1.41E+06	481080	2.20E+04	1.06E+04	9.52E+03
Aldh2	Q544B1	Aldehyde dehydrogenase, mitochondrial	4257800	3.19E+06	2742800	1.11E+04	0.00E+00	0.00E+00
Aldh5a1	B2RS41	Succinate-semialdehyde dehydrogenase, mitochondrial	5565000	5.48E+06	3317000	0.00E+00	1.50E+04	0.00E+00
Aldh6a1	Q9EQ20	Methylmalonate-semialdehyde dehydrogenase [acylating], mitochondrial	2205100	2.52E+06	2083300	1.74E+04	0.00E+00	0.00E+00
Aldoa	Q5FWB7	Fructose-bisphosphate aldolase;Fructose-bisphosphate aldolase A	12628000	8.65E+06	7852200	3.63E+04	3.84E+04	9.65E+04
Aldoc	P05063	Fructose-bisphosphate aldolase C	4913400	2.42E+06	2150400	8.13E+03	0.00E+00	0.00E+00
Amph	A0A0G2JEG8	Amphiphysin	2439900	0.00E+00	0	0.00E+00	2.90E+04	4.47E+04
Ank2	S4R2F3	Ankyrin-2	117420	1.87E+04	143540	4.03E+05	2.81E+05	2.32E+05
Anks1b	Q8BIZ1	Ankyrin repeat and sterile alpha motif domain-containing protein 1B	241890	1.63E+05	150790	3.48E+05	3.34E+05	3.29E+05
Ap1b1	Q5SVG5	AP complex subunit beta;AP-1 complex subunit beta-1	480770	5.67E+05	405880	1.60E+04	0.00E+00	0.00E+00
Ap2a1	P17426	AP-2 complex subunit alpha-1	6560100	6.02E+06	6078100	3.18E+04	9.74E+04	4.18E+04
Ap2b1	Q5SWR1	AP complex subunit beta;AP-2 complex subunit beta	11113000	9.23E+06	8787400	6.69E+04	1.04E+05	2.45E+04
Ap2m1	Q3TWV4	AP-2 complex subunit mu	3061300	3.80E+06	2505400	0.00E+00	7.25E+04	1.90E+03
Aqp4	Q8BR89	Aquaporin-4	361180	1.01E+06	1462600	1.17E+04	0.00E+00	0.00E+00
Arf3	Q3U344	ADP-ribosylation factor 1;ADP-ribosylation factor 3;ADP-ribosylation factor 2	5355200	4.23E+06	2678200	3.83E+05	3.00E+05	4.26E+05
Atp1a1	Q8VDN2	Sodium/potassium-transporting ATPase subunit alpha-1	0	2.94E+07	14772000	4.32E+05	2.12E+05	1.98E+04
Atp1a2	Q3UHK5	Sodium/potassium-transporting ATPase subunit alpha-2	1.22E+08	1.17E+08	0	1.92E+06	1.39E+06	7.25E+05
Atp1a3	A0A0G2JGX4	Sodium/potassium-transporting ATPase subunit alpha-3	42688000	4.22E+07	40349000	4.23E+05	4.34E+05	3.11E+05
Atp1b1	Q545P0	Sodium/potassium-transporting ATPase subunit beta-1	19198000	2.07E+07	15238000	1.18E+06	1.05E+06	3.54E+05
Atp2a2	Q5DTI2	Sarcoplasmic/endoplasmic reticulum calcium ATPase 2	10572000	1.21E+07	9433900	1.39E+05	2.59E+05	6.12E+04
Atp2b1	G5E829	Plasma membrane calcium-transporting ATPase 1	9411400	1.05E+07	7835200	7.76E+04	2.98E+05	0.00E+00
Atp2b2	Q3UHH0	Calcium-transporting ATPase;Plasma membrane calcium-transporting ATPase 2	19120000	1.96E+07	14271000	1.41E+05	1.19E+05	7.89E+04
Atp2b3	Q0VF55	Calcium-transporting ATPase	2608500	2.14E+06	1787400	7.87E+03	0.00E+00	0.00E+00
Atp2b4	H2BL43	Calcium-transporting ATPase	991580	1.24E+06	1212700	1.59E+04	0.00E+00	0.00E+00
Atp5a1	Q03265	ATP synthase subunit alpha, mitochondrial;ATP synthase subunit alpha	48485000	5.80E+07	38733000	2.17E+05	1.89E+05	1.47E+05
Atp5b	P56480	ATP synthase subunit beta, mitochondrial	76755000	8.57E+07	68003000	1.81E+05	1.22E+05	2.11E+04
Atp5c1	Q8C2Q8	ATP synthase subunit gamma;ATP synthase subunit gamma, mitochondrial	1622800	3.78E+06	2042600	4.50E+04	7.63E+04	3.00E+04
Atp5h	Q9DCX2	ATP synthase subunit d, mitochondrial	8049300	6.37E+06	8684800	0.00E+00	8.70E+03	6.86E+03

Atp5j2	F8WHP8	ATP synthase subunit f, mitochondrial	231340	1.86E+05	0	1.12E+04	0.00E+00	1.28E+04
Atp6v0a1	Q6NXX6	V-type proton ATPase subunit a;V-type proton ATPase 116 kDa subunit a isoform 1	9697600	7.19E+06	6293800	6.77E+05	3.21E+05	3.51E+05
Atp6v0d1	P51863	V-type proton ATPase subunit d 1	1107900	1.25E+06	794690	6.52E+04	0.00E+00	5.28E+04
Atp6v1a	P50516	V-type proton ATPase catalytic subunit A	11823000	7.90E+06	6276900	1.39E+05	9.04E+04	9.99E+04
Atp6v1b2	A0A1S6GWG6	V-type proton ATPase subunit B, brain isoform	5134800	2.44E+06	3154400	1.63E+05	5.66E+04	8.82E+04
Atp6v1e1	P50518	V-type proton ATPase subunit E 1	1450200	1.40E+06	790690	4.42E+03	0.00E+00	0.00E+00
Atp8a1	A1L332	Phospholipid-transporting ATPase;Phospholipid-transporting ATPase IA	2290100	1.50E+06	777780	3.94E+04	0.00E+00	0.00E+00
Baiap2	B1AZ46	Brain-specific angiogenesis inhibitor 1-associated protein 2	1222500	1.07E+06	532490	4.59E+05	4.15E+05	2.20E+05
Basp1	Q91XV3	Brain acid soluble protein 1	0	0.00E+00	14743000	9.80E+06	4.05E+06	4.10E+06
Bcas1	Q14DK3	Breast carcinoma-amplified sequence 1 homolog	1081300	8.44E+05	337280	5.39E+05	3.13E+05	2.11E+05
Bclaf1	Q8K019	Bcl-2-associated transcription factor 1	2980900	5.13E+06	4630400	7.46E+05	1.04E+06	5.28E+05
Bdh1	Q80XN0	D-beta-hydroxybutyrate dehydrogenase, mitochondrial	701600	1.12E+06	934000	0.00E+00	5.51E+03	0.00E+00
Bsg	J3QP71	Basigin	1084800	1.56E+06	139980	0.00E+00	1.33E+04	0.00E+00
Bsn	O88737	Protein bassoon	4569600	0.00E+00	0	4.94E+05	2.89E+05	1.36E+05
Cacna2d1	Q14BH8		2918900	1.99E+06	1355500	3.33E+05	4.08E+05	3.65E+05
Cadps	Q80TJ1	Calcium-dependent secretion activator 1	1632500	8.80E+05	1032200	5.67E+04	3.04E+04	3.93E+04
Camk2a	Q80TN1	Calcium/calmodulin-dependent protein kinase type II subunit alpha	10023000	9.43E+06	6986600	8.64E+06	9.43E+06	6.95E+06
Camk2b	P28652	Calcium/calmodulin-dependent protein kinase type II subunit beta	26743000	2.55E+07	13934000	1.39E+07	6.32E+06	8.54E+06
Camkv	Q3UHL1	CaM kinase-like vesicle-associated protein	5154100	2.28E+06	2260800	7.79E+04	6.31E+04	8.41E+04
Cand1	Q6ZQ38	Cullin-associated NEDD8-dissociated protein 1	1016100	3.68E+05	640470	9.96E+03	0.00E+00	1.40E+04
Capza2	Q5DQJ3	F-actin-capping protein subunit alpha-2	1463400	1.15E+06	1296600	1.36E+05	6.03E+03	1.52E+04
Capzb	Q3TRH8	F-actin-capping protein subunit beta	1292000	9.16E+05	1255400	1.76E+05	1.78E+04	0.00E+00
Cct8	H3BL49	T-complex protein 1 subunit theta	310230	1.27E+05	151790	0.00E+00	4.98E+03	0.00E+00
Cds2	A2AMQ5	Phosphatidate cytidylyltransferase;Phosphatidate cytidylyltransferase 2	4773800	5.63E+06	4612400	1.52E+04	0.00E+00	5.20E+04
Celf2	Q9Z0H4	CUGBP Elav-like family member 2	3327700	4.97E+06	5501100	6.40E+04	4.67E+04	4.71E+04
Cenpv	Q9CXS4	Centromere protein V	94893	5.54E+05	261270	1.67E+07	2.46E+07	1.68E+07
Chchd6	E9Q4M4	MICOS complex subunit Mic25	419280	3.54E+05	289170	0.00E+00	1.84E+04	9.81E+03
Ckb	Q04447	Creatine kinase B-type	32093000	2.31E+07	15318000	7.40E+04	6.37E+04	5.50E+04
Ckmt1	Q545N7	Creatine kinase U-type, mitochondrial	8820300	9.18E+06	5628500	9.63E+03	1.97E+04	0.00E+00
Cldn11	Q60771	Claudin-11	1721500	2.28E+06	2824800	3.25E+06	2.01E+06	1.45E+06

Cltc	Q5SXR6	Clathrin heavy chain;Clathrin heavy chain 1	33133000	2.62E+07	23146000	6.22E+05	4.89E+05	4.07E+05
Cnp	Q3TYL9	2,3-cyclic-nucleotide 3-phosphodiesterase	39038000	0.00E+00	42297000	4.00E+06	2.45E+06	2.39E+06
Cnrip1	Q5M8N0	CB1 cannabinoid receptor-interacting protein 1	1765600	1.33E+06	959730	7.33E+04	3.64E+04	2.37E+04
Cntn1	P12960	Contactin-1	8203500	7.09E+06	4487200	1.50E+06	1.08E+06	8.58E+05
Cntnap1	O54991	Contactin-associated protein 1	2425700	1.93E+06	768070	9.22E+04	1.58E+05	8.34E+04
Col4a2	B2RQQ8	Collagen alpha-2(IV) chain;Canstatin	0	6.99E+04	155430	7.66E+06	1.13E+07	7.01E+06
Cox4i1	P19783	Cytochrome c oxidase subunit 4 isoform 1, mitochondrial	5355600	6.89E+06	8987600	6.35E+04	5.02E+04	1.65E+04
Crmp1	Q6P1J1	Dihydropyrimidinase-related protein 1	2924200	1.18E+06	1171000	3.18E+04	1.66E+04	3.23E+05
Cryab	P23927	Alpha-crystallin B chain	54570	2.01E+05	99082	1.84E+04	0.00E+00	4.77E+03
Csnk2a1	Q61177	Casein kinase II subunit alpha	1542900	1.79E+06	1844800	0.00E+00	1.03E+04	2.92E+03
Ctnna2	E0CXB9	Catenin alpha-2	3158200	3.78E+06	2442600	1.35E+05	1.66E+05	1.13E+05
Ctnnb1	Q3UZT7	Catenin beta-1	1490600	1.78E+06	2032900	2.49E+05	1.87E+05	1.39E+05
Cyfip2	Q5SQX6	Cytoplasmic FMR1-interacting protein 2	1174700	7.61E+05	814780	2.66E+04	5.42E+04	1.53E+04
Dbn1	Q9QXS6	Drebrin	3003600	2.39E+06	1796700	1.16E+05	8.97E+04	1.33E+04
Dclk1	Q9JLM8	Serine/threonine-protein kinase DCLK1	1027400	6.75E+05	562300	1.73E+05	1.19E+05	1.93E+05
Ddx17	Q3U741	Probable ATP-dependent RNA helicase DDX17	10571000	1.52E+07	11598000	4.42E+06	2.80E+06	2.67E+06
Ddx39b	A0A1S6GWH2	Spliceosome RNA helicase Ddx39b	1202300	1.71E+06	2272900	1.08E+04	0.00E+00	3.11E+04
Ddx3x	B9EKE9	ATP-dependent RNA helicase DDX3X;Putative ATP-dependent RNA helicase Pl10	71561	1.73E+05	73375	6.78E+04	1.03E+05	9.80E+04
Ddx5	Q8BTS0	Probable ATP-dependent RNA helicase DDX5	5092500	5.96E+06	6689400	2.20E+06	2.23E+06	1.84E+06
Dkc1	Q3TM67	H/ACA ribonucleoprotein complex subunit 4	966230	1.33E+06	1530200	3.17E+05	4.63E+05	3.79E+05
Dlat	Q8BMF4	Dihydrolipoyllysine-residue acetyltransferase component of pyruvate dehydrogenase complex, mitochondrial	4660200	6.49E+06	4954500	1.95E+04	6.12E+04	5.31E+03
Dld	O08749	Dihydrolipoyl dehydrogenase, mitochondrial;Dihydrolipoyl dehydrogenase	7262700	3.81E+06	1850400	0.00E+00	2.79E+04	0.00E+00
Dlg1	D3Z3B8	Disks large homolog 1	550950	7.47E+05	541550	9.62E+04	3.85E+04	4.69E+04
Dlg4	Q62108	Disks large homolog 4	1749000	8.90E+05	730340	3.12E+06	2.76E+06	2.61E+06
Dlst	Q9D2G2	Dihydrolipoyllysine-residue succinyltransferase component of 2-oxoglutarate dehydrogenase complex, mitochondrial	3045800	3.09E+06	2181000	4.14E+04	0.00E+00	0.00E+00
Dpysl2	O08553	Dihydropyrimidinase-related protein 2	37871000	2.16E+07	21205000	1.05E+06	1.30E+06	9.26E+05
Dpysl3	Q3TT92	Dihydropyrimidinase-related protein 3	1139000	5.39E+05	171930	1.08E+04	0.00E+00	2.55E+04
Dync1h1	Q9JHU4	Cytoplasmic dynein 1 heavy chain 1	5877300	4.85E+06	3046600	1.04E+05	2.83E+05	1.72E+05
Dynll2	Q9D0M5	Dynein light chain 2, cytoplasmic	1615900	1.31E+06	2121300	2.11E+05	2.65E+05	2.38E+05
Echs1	Q8BH95	Enoyl-CoA hydratase, mitochondrial	1610700	1.87E+06	1641300	0.00E+00	2.26E+04	0.00E+00
Eef1a1	Q58E64	Elongation factor 1-alpha;Elongation factor 1-alpha 1	1489700	5.08E+05	1149300	7.39E+04	2.81E+05	9.61E+04

Eftud2	Q543F1	116 kDa U5 small nuclear ribonucleoprotein component	3960100	5.62E+06	4627800	7.61E+05	1.31E+06	5.31E+05
EG433182	Q5FW97	Alpha-enolase	7273400	5.40E+06	5752500	3.04E+05	9.03E+04	5.45E+04
Eif4a3	Q91VC3	Eukaryotic initiation factor 4A-III;Eukaryotic initiation factor 4A-III, N-terminally processed	2376600	2.48E+06	4127000	9.53E+05	1.03E+06	7.58E+05
Elavl1	Q8BW03	ELAV-like protein;ELAV-like protein 1	9113600	1.04E+07	11579000	2.71E+05	4.25E+05	2.22E+05
Eno2	Q545V3	Gamma-enolase;Enolase	7216000	4.13E+06	3907900	8.48E+04	1.26E+04	0.00E+00
Epb4.1l2	Q80UE5	Band 4.1-like protein 2	1692400	1.59E+06	1662200	4.45E+03	0.00E+00	1.63E+04
Erh	Q4FZH7	Enhancer of rudimentary homolog	435220	5.81E+05	723000	3.71E+05	3.85E+05	5.32E+05
Fh	P97807	Fumarate hydratase, mitochondrial	5124700	5.84E+06	1984900	1.12E+04	2.59E+04	0.00E+00
Fkbp1a	Q3ULN5	Peptidyl-prolyl cis-trans isomerase;Peptidyl-prolyl cis-trans isomerase FKBP1A	297280	1.25E+05	64334	1.35E+04	0.00E+00	3.74E+03
Fus	Q8CFQ9	RNA-binding protein FUS	8001600	0.00E+00	11387000	3.63E+05	2.55E+05	3.06E+05
Gabra1	Q544F7	Gamma-aminobutyric acid receptor subunit alpha-1	356550	4.97E+05	308640	5.35E+03	0.00E+00	0.00E+00
Gad2	P48320	Glutamate decarboxylase 2	241300	1.34E+05	31077	1.38E+04	0.00E+00	0.00E+00
Gap43	P06837	Neuromodulin	5665000	4.37E+06	3114600	7.87E+05	2.40E+05	1.65E+05
Gfap	P03995	Glial fibrillary acidic protein	22855000	3.65E+07	27671000	3.37E+07	3.62E+07	3.88E+07
Gja1	Q7TMQ1	Gap junction protein;Gap junction alpha-1 protein	1455700	1.27E+06	1045700	7.78E+05	9.98E+05	4.15E+05
Glud1	P26443	Glutamate dehydrogenase 1, mitochondrial	8766800	1.20E+07	9319300	2.20E+04	1.23E+04	0.00E+00
Glul	P15105	Glutamine synthetase	6788200	4.81E+06	3591400	9.39E+03	9.28E+03	2.37E+04
Gnai1	B2RSH2	Guanine nucleotide-binding protein G(i) subunit alpha-1	2553400	2.67E+06	1777700	1.69E+05	1.22E+05	9.08E+04
Gnai2	P08752	Guanine nucleotide-binding protein G(i) subunit alpha-2	7253200	7.36E+06	4830500	4.72E+05	6.00E+05	5.06E+05
Gnaq	Q3UHH5	Guanine nucleotide-binding protein G(q) subunit alpha	6349600	5.63E+06	3437200	1.02E+05	1.01E+05	7.23E+04
Gnaz	O70443	Guanine nucleotide-binding protein G(z) subunit alpha	1448600	9.63E+05	911690	1.33E+05	4.25E+04	1.29E+05
Gnb1	Q3TQ70	Guanine nucleotide-binding protein G(I)/G(S)/G(T) subunit beta-1	11708000	1.22E+07	0	8.64E+03	4.38E+04	0.00E+00
Got2	P05202	Aspartate aminotransferase, mitochondrial	11488000	1.25E+07	7796500	4.26E+04	1.10E+05	4.11E+04
Gpd2	A2AQR0	Glycerol-3-phosphate dehydrogenase;Glycerol-3-phosphate dehydrogenase, mitochondrial	5900900	6.61E+06	4617000	1.91E+04	0.00E+00	0.00E+00
Gpm6a	Q8R1P3	Neuronal membrane glycoprotein M6-a	4027700	1.49E+06	1387400	2.88E+05	0.00E+00	2.13E+05
Gria2	C9K0Z0	Glutamate receptor 2	2731900	3.02E+06	2027000	2.18E+05	2.36E+05	6.22E+04
H1f0	Q8C1Y3	Histone H1.0;Histone H1.0, N-terminally processed	19315	1.13E+05	83064	1.76E+07	1.29E+07	1.48E+07
H2afv	B2RVP5	Histone H2A;Histone H2A.V;Histone H2A.Z	373120	1.76E+05	2147300	5.01E+06	6.19E+06	2.87E+06
H2afy	Q9QZQ8	Core histone macro-H2A.1	3782100	5.02E+06	5513000	3.30E+07	3.17E+07	3.43E+07
H2afy2	Q8CCK0	Core histone macro-H2A.2;Core histone macro-H2A	595280	8.68E+05	686850	7.36E+06	7.65E+06	7.24E+06
Hadha	Q8BMS1	Trifunctional enzyme subunit alpha, mitochondrial;Long-chain enoyl-CoA hydratase;Long chain 3-hydroxyacyl-CoA	2745100	1.75E+06	2336800	2.40E+04	3.24E+04	1.96E+04

		dehydrogenase						
Hist1h1b	Q1WWK3	Histone H1.5	0	0.00E+00	0	9.28E+06	8.20E+06	6.17E+06
Hist1h1e	P43274	Histone H1.4	415390	4.31E+05	695870	7.62E+07	9.46E+07	7.13E+07
Hist2h2ab	Q64522	Histone H2A type 2-B;Histone H2AX	194580	4.88E+05	483400	3.52E+05	1.43E+06	8.58E+05
Hist2h2ac	Q149V4	Histone H2A;Histone H2A type 2-C;Histone H2A type 2-A	22847000	3.09E+07	32501000	6.40E+07	1.23E+08	6.72E+07
Hist2h4	B2RTM0	Histone H4	24627000	3.18E+07	29750000	2.68E+08	3.38E+08	3.21E+08
Hk1	P17710	Hexokinase-1;Hexokinase	10857000	1.07E+07	9743200	4.69E+04	2.58E+04	2.14E+04
Hnrnpa1	Q3U7F3	Heterogeneous nuclear ribonucleoprotein A1;Heterogeneous nuclear ribonucleoprotein A1, N-terminally processed	18311000	2.80E+07	27541000	6.53E+06	8.81E+06	5.15E+06
Hnrnpa2b1	O88569	Heterogeneous nuclear ribonucleoproteins A2/B1	66395000	9.57E+07	0	1.71E+07	1.09E+07	9.57E+06
Hnrnpab	Q9D6G1	Heterogeneous nuclear ribonucleoprotein A/B	6961600	7.94E+06	6861900	1.70E+06	6.73E+05	7.53E+05
Hnrnpc	Q9Z204	Heterogeneous nuclear ribonucleoproteins C1/C2	4778300	6.93E+06	11433000	1.20E+07	1.72E+07	9.92E+06
Hnrnpd	Q60668	Heterogeneous nuclear ribonucleoprotein D0	20006000	1.85E+07	25492000	3.23E+06	2.13E+06	1.50E+06
Hnrnph1	Q811L7	Heterogeneous nuclear ribonucleoprotein H;Heterogeneous nuclear ribonucleoprotein H, N-terminally processed	14834000	0.00E+00	19689000	9.83E+05	1.78E+06	9.32E+05
Hnrnph2	P70333	Heterogeneous nuclear ribonucleoprotein H2	7564500	1.19E+07	9855800	2.28E+05	7.68E+05	1.16E+05
Hnrnph3	D3Z3N4		3925800	4.93E+06	6660200	1.25E+06	1.19E+06	4.49E+05
Hnrnpl	Q3UMT7	Heterogeneous nuclear ribonucleoprotein L	0	6.22E+07	0	8.59E+06	8.75E+06	4.37E+06
Hnrnpm	Q3THB3	Heterogeneous nuclear ribonucleoprotein M	2429700	5.34E+06	4813300	2.51E+06	2.29E+06	1.71E+06
Hnrnpr	Q8VHM5		8415600	1.32E+07	11045000	3.12E+06	5.67E+06	2.36E+06
Hnrnpu	Q3ULH5	Heterogeneous nuclear ribonucleoprotein U	13336000	0.00E+00	17837000	2.60E+07	2.11E+07	1.82E+07
Hnrnpul2	Q00PI9	Heterogeneous nuclear ribonucleoprotein U-like protein 2	7135100	1.08E+07	10065000	8.34E+06	1.40E+07	5.27E+06
Homer1	Q9Z2Y3	Homer protein homolog 1	726970	4.24E+05	392450	1.81E+06	1.45E+06	1.09E+06
Hp1bp3	Z4YKB8	Heterochromatin protein 1-binding protein 3	612100	1.05E+06	1427200	2.90E+06	6.25E+06	0.00E+00
Hpca	P84075	Neuron-specific calcium-binding protein hippocalcin	938320	1.88E+06	746300	2.73E+04	4.03E+04	1.90E+04
Hsp90aa1	Q80Y52	Heat shock protein HSP 90-alpha	0	5.62E+06	5941500	4.05E+05	3.41E+05	3.21E+05
Hsp90ab1	Q71LX8	Heat shock protein HSP 90-beta	3526100	2.36E+06	1152100	9.66E+04	9.32E+04	1.37E+05
Hsp90b1	Q91V38	Endoplasmin	2877000	3.79E+06	4616400	1.10E+05	8.91E+03	5.66E+03
Hspa12a	Q8K0U4	Heat shock 70 kDa protein 12A	3389000	3.19E+06	2244900	5.13E+05	6.10E+05	2.92E+05
Hspa4l	P48722	Heat shock 70 kDa protein 4L	1897800	1.60E+06	1637000	9.99E+03	0.00E+00	3.13E+03
Hspa5	Q9DC41	78 kDa glucose-regulated protein	15841000	1.75E+07	15804000	3.26E+05	3.66E+05	2.68E+05
Hspa8	P63017	Heat shock cognate 71 kDa protein	41585000	3.37E+07	30783000	0.00E+00	7.06E+06	4.06E+06
Hspa9	P38647	Stress-70 protein, mitochondrial	4303900	6.19E+06	4123700	1.29E+05	5.26E+04	3.81E+04

Hspd1	P63038	60 kDa heat shock protein, mitochondrial	5536500	5.50E+06	3965500	1.33E+03	0.00E+00	0.00E+00
Hsph1	E9Q0U7	Heat shock protein 105 kDa	620970	3.00E+05	686920	1.50E+04	1.83E+04	0.00E+00
Idh2	P54071	Isocitrate dehydrogenase [NADP], mitochondrial	827610	2.12E+06	1191800	5.97E+03	7.71E+03	0.00E+00
Idh3a	A0A1L1STE6	Isocitrate dehydrogenase [NAD] subunit alpha, mitochondrial	5199600	6.63E+06	5154800	2.91E+04	0.00E+00	3.40E+04
Idh3b	Q91VA7	Isocitrate dehydrogenase [NAD] subunit, mitochondrial	5168100	4.24E+06	4003100	9.93E+04	1.43E+05	5.84E+04
Ilf2	Q3UXI9	Interleukin enhancer-binding factor 2	4441000	5.83E+06	5296200	1.30E+06	1.09E+06	6.67E+05
Immt	Q3TVZ5	MICOS complex subunit Mic60	5477200	5.97E+06	6279500	6.12E+05	6.75E+05	3.65E+05
Ina	P46660	Alpha-internexin	18360000	1.77E+07	16507000	6.34E+06	3.45E+06	2.62E+06
Kcna2	P63141	Potassium voltage-gated channel subfamily A member 2	668320	8.83E+05	303040	9.82E+04	2.97E+04	1.71E+05
Khdrbs1	Q60749	KH domain-containing, RNA-binding, signal transduction-associated protein 1	916980	2.02E+06	1190900	0.00E+00	1.25E+06	1.13E+04
Kiaa1045	Q80TL4	Protein KIAA1045	873840	5.80E+05	211710	9.28E+03	3.64E+03	2.18E+03
Kpnb1	Q3TFE8	Importin subunit beta-1	1977800	4.23E+06	2791400	4.03E+04	2.35E+04	0.00E+00
Lama5	Q61001	Laminin subunit alpha-5	15705	2.19E+05	90984	9.31E+06	1.48E+07	9.69E+06
Lamb2	Q61292	Laminin subunit beta-2	2137400	3.24E+06	2830700	2.96E+07	3.64E+07	2.91E+07
Lamc1	F8VQJ3	Laminin subunit gamma-1	1803900	3.02E+06	2547200	2.96E+07	3.98E+07	2.58E+07
Lancl2	F6RJV6	LanC-like protein 2	950260	6.91E+05	996800	6.27E+03	5.66E+04	1.82E+04
Ldha	P06151	L-lactate dehydrogenase A chain;L-lactate dehydrogenase	1223300	6.98E+05	1065100	7.02E+04	2.16E+04	1.54E+04
Ldhb	P16125	L-lactate dehydrogenase B chain;L-lactate dehydrogenase	3113700	1.96E+06	1584700	4.98E+04	3.37E+04	2.07E+04
Lgi1	A0A0G2JGB7	Leucine-rich glioma-inactivated protein 1	1490600	1.59E+06	855550	2.47E+04	0.00E+00	9.39E+03
Lmna	P48678	Prelamin-A/C;Lamin-A/C	31577000	4.83E+07	32413000	6.97E+06	5.45E+06	4.90E+06
Lmnb1	P14733	Lamin-B1	19263000	2.83E+07	28580000	6.78E+06	7.94E+06	5.36E+06
Lmnb2	P21619	Lamin-B2	6853200	1.25E+07	18544000	4.16E+06	5.94E+06	2.96E+06
Lsamp	Q3TYE5	Limbic system-associated membrane protein	766880	2.74E+05	361510	6.71E+05	7.79E+05	6.82E+05
Ly6h	Q544M1	Lymphocyte antigen 6H	1318500	1.27E+06	1439700	7.40E+04	5.41E+04	4.42E+04
Map1a	A2ARP8	Microtubule-associated protein 1A;MAP1A heavy chain;MAP1 light chain LC2	7063500	6.13E+06	3932700	1.03E+05	3.08E+05	2.11E+05
Map1b	B2RQQ5	Microtubule-associated protein 1B;MAP1B heavy chain;MAP1 light chain LC1	9944600	9.92E+06	7030800	7.84E+05	8.92E+05	3.31E+05
Map2	P20357	Microtubule-associated protein 2	8209400	4.74E+06	3814600	3.29E+05	8.40E+05	1.43E+05
Map2k1	Q9JJE1	Dual specificity mitogen-activated protein kinase kinase 1	834120	2.56E+05	230730	1.14E+05	1.07E+05	1.36E+05
Map6	Q7TSJ2	Microtubule-associated protein 6	2126300	1.37E+06	1145400	3.47E+05	3.07E+05	3.23E+05
Marcks	P26645	Myristoylated alanine-rich C-kinase substrate	0	0.00E+00	2258400	6.57E+02	0.00E+00	0.00E+00
Matr3	Q8K310	Matrin-3	46770000	6.22E+07	63394000	9.50E+06	1.60E+07	7.37E+06
Mdh1	P14152	Malate dehydrogenase, cytoplasmic	7796400	5.59E+06	4302500	3.03E+04	1.06E+05	2.08E+03

Mdh2	P08249	Malate dehydrogenase, mitochondrial	26995000	2.94E+07	20412000	8.38E+03	0.00E+00	0.00E+00
Mecp2	Q9Z2D6	Methyl-CpG-binding protein 2	2451300	5.28E+06	0	1.84E+07	2.95E+07	2.18E+07
Mobp	Q9D2P8	Myelin-associated oligodendrocyte basic protein	145360	2.84E+05	125230	3.41E+06	2.49E+06	2.50E+06
Mog	Q3UY21	Myelin-oligodendrocyte glycoprotein	13262000	1.27E+07	6850400	4.08E+05	2.23E+05	2.55E+05
Msn	Q3TZQ2	Moesin	638600	4.40E+05	502210	0.00E+00	7.97E+03	0.00E+00
Myh10	Q5SV64	Myosin-10	3732500	4.40E+06	3766700	1.06E+06	1.01E+06	6.14E+05
Myh11	A0A338P6K2		3122200	6.44E+06	3440600	1.57E+07	1.69E+07	1.81E+07
Myl6	A0A1W2P6G5	Myosin light polypeptide 6	1531400	2.28E+06	2423800	1.62E+06	8.95E+05	2.23E+06
Myo5a	D3YZ62	Unconventional myosin-Va	5186200	4.31E+06	3434000	9.59E+05	9.29E+05	6.83E+05
Napa	Q9DB05	Alpha-soluble NSF attachment protein	2116200	1.72E+06	814770	9.32E+03	0.00E+00	1.07E+04
Napb	P28663	Beta-soluble NSF attachment protein	7115500	5.13E+06	2975000	3.49E+04	5.46E+04	3.88E+04
Ncam1	A0A0A6YY91	Neural cell adhesion molecule 1	6497200	7.93E+06	4686500	5.07E+05	3.11E+05	3.04E+05
Ncdn	Q9Z0E0	Neurochondrin	1325500	9.00E+05	824490	3.59E+04	9.94E+04	4.10E+04
Nckap1	A2AS98	Nck-associated protein 1	1095900	3.76E+05	356730	0.00E+00	1.59E+04	0.00E+00
Ndufa4	A0A0N4SVQ1	Cytochrome c oxidase subunit NDUFA4	1408100	1.72E+06	950160	1.70E+04	5.29E+04	5.84E+04
Ndufs1	Q3TIU7	NADH-ubiquinone oxidoreductase 75 kDa subunit, mitochondrial	7171900	9.42E+06	7453100	5.45E+04	3.76E+04	1.46E+04
Ndufs2	Q91WD5	NADH dehydrogenase [ubiquinone] iron-sulfur protein 2, mitochondrial	3091400	3.33E+06	3664100	0.00E+00	8.70E+03	0.00E+00
Ndufs3	Q9DCT2	NADH dehydrogenase [ubiquinone] iron-sulfur protein 3, mitochondrial	3806600	2.55E+06	3824900	4.48E+04	1.35E+04	6.87E+03
Nefh	Q80TQ3	Neurofilament heavy polypeptide	7427700	1.11E+07	8845100	1.89E+06	1.06E+06	7.10E+05
Nefl	P08551	Neurofilament light polypeptide	26553000	3.73E+07	33955000	1.16E+07	6.86E+06	5.27E+06
Nefm	P08553	Neurofilament medium polypeptide	0	2.08E+07	25366000	8.76E+06	3.49E+06	4.34E+06
Negr1	A0A4W9	Neuronal growth regulator 1	777560	2.25E+05	186860	3.92E+05	5.84E+05	3.92E+05
Nid1	P10493	Nidogen-1	937940	1.59E+06	1274000	9.14E+06	1.33E+07	8.59E+06
Nid2	Q8R5G0	Nidogen-2	196390	4.14E+05	544570	4.98E+06	5.62E+06	5.08E+06
Nono	Q4FK11	Non-POU domain-containing octamer-binding protein	7246100	1.32E+07	8282400	0.00E+00	7.76E+04	4.23E+04
Nop56	Q9D6Z1	Nucleolar protein 56	5788100	8.25E+06	5291400	3.18E+06	4.86E+06	4.04E+06
Nop58	Q6DFW4	Nucleolar protein 58	4089800	4.74E+06	5040700	4.07E+06	5.99E+06	3.36E+06
Npm1	Q5SQB0	Nucleophosmin	3269700	4.44E+06	4582700	1.93E+06	2.83E+06	1.65E+06
Nptn	P97300	Neuroplastin	3619900	1.28E+06	1640000	2.52E+04	4.38E+04	1.87E+04
Nsf	P46460	Vesicle-fusing ATPase	13235000	1.09E+07	6594900	5.17E+05	6.96E+05	4.95E+05
Ntm	D3Z396	Neurotrimin	1980200	9.43E+05	1171800	1.40E+06	1.09E+06	9.99E+05
Ogdh	Q60597	2-oxoglutarate dehydrogenase, mitochondrial	4469200	4.36E+06	3848600	1.28E+04	0.00E+00	0.00E+00

Ogdhl	B2RXT3		2105900	2.79E+06	1236500	0.00E+00	2.00E+04	0.00E+00
Omg	Q3UVV3	Oligodendrocyte-myelin glycoprotein	458390	4.53E+05	309840	2.46E+04	0.00E+00	3.55E+04
Opa1	P58281	Dynamin-like 120 kDa protein, mitochondrial;Dynamin-like 120 kDa protein, form S1	2536400	2.43E+06	2220000	0.00E+00	2.08E+04	0.00E+00
Opcml	Q6DFY2		1016100	2.37E+05	238430	6.47E+05	3.19E+05	4.02E+05
Oxr1	Q4KMM3	Oxidation resistance protein 1	1169600	5.92E+05	664200	1.27E+03	9.21E+03	2.14E+04
Pacsin1	Q543Y7	Protein kinase C and casein kinase substrate in neurons protein 1	3391300	2.03E+06	2149500	5.99E+03	6.70E+04	4.17E+04
Palm	Q6ZQE7	Paralemmin-1	4358200	2.74E+06	3430900	4.22E+05	2.89E+05	2.26E+05
Pcbp1	P60335	Poly(rC)-binding protein 1	3128100	3.11E+06	2762100	3.55E+05	4.73E+05	2.33E+05
Pcbp2	A0A2R8VI25	Poly(rC)-binding protein 2	587800	7.91E+05	431600	0.00E+00	1.97E+05	2.04E+05
Pcx	G5E8R3	Pyruvate carboxylase;Pyruvate carboxylase, mitochondrial	2990200	4.49E+06	2091000	1.08E+04	0.00E+00	0.00E+00
Pde2a	Q3TD37	cGMP-dependent 3,5-cyclic phosphodiesterase	1686600	8.77E+05	896150	7.96E+04	5.24E+03	6.69E+04
Pdha1	Q3UFJ3	Pyruvate dehydrogenase E1 component subunit alpha;Pyruvate dehydrogenase E1 component subunit alpha, somatic form, mitochondrial	3664100	3.95E+06	4232900	3.32E+04	0.00E+00	0.00E+00
Pfkp	Q8C605	ATP-dependent 6-phosphofructokinase;ATP-dependent 6-phosphofructokinase, platelet type	2070500	1.61E+06	739660	7.23E+04	2.26E+04	6.20E+04
Pgk1	P09411	Phosphoglycerate kinase 1;Phosphoglycerate kinase	3122800	1.62E+06	1866300	3.17E+04	0.00E+00	0.00E+00
Phb	P67778	Prohibitin	1477500	2.20E+06	1542700	1.23E+04	1.66E+04	3.19E+03
Phgdh	Q61753	D-3-phosphoglycerate dehydrogenase	1063500	2.94E+05	712940	2.04E+04	0.00E+00	4.59E+04
Pi4ka	A0A140T8I9		2101700	1.44E+06	939400	1.99E+04	2.63E+04	0.00E+00
Pkm	P52480	Pyruvate kinase PKM	7655700	4.38E+06	3373700	5.55E+04	2.00E+04	1.64E+04
Plec	Q6S388	Plectin	12769000	1.68E+07	15104000	6.24E+06	5.29E+06	3.94E+06
Plp1	P60202	Myelin proteolipid protein	31667000	3.96E+07	18831000	9.24E+04	2.51E+06	1.00E+06
Ppia	Q5SVY2	Peptidyl-prolyl cis-trans isomerase;Peptidyl-prolyl cis-trans isomerase A;Peptidyl-prolyl cis-trans isomerase A, N-terminally processed	5419400	3.83E+06	2780200	6.15E+04	1.84E+04	2.23E+04
Ppp1ca	Q3U8W0	Serine/threonine-protein phosphatase PP1-alpha catalytic subunit;Serine/threonine-protein phosphatase	2529900	1.93E+06	3005500	1.89E+04	0.00E+00	0.00E+00
Ppp1r9b	Q6R891	Neurabin-2	1258200	1.11E+06	608110	4.37E+05	2.98E+05	3.03E+05
Ppp3ca	B2RRX2	Serine/threonine-protein phosphatase;Serine/threonine-protein phosphatase 2B catalytic subunit alpha isoform	4560900	3.51E+06	3240000	1.11E+05	6.50E+04	6.56E+04
Prkar2b	P31324	cAMP-dependent protein kinase type II-beta regulatory subunit	2663800	2.00E+06	1248500	1.24E+04	4.96E+04	0.00E+00
Prkcg	P63318	Protein kinase C gamma type;Protein kinase C	4283800	5.70E+06	3689500	2.19E+04	0.00E+00	0.00E+00
Prpf19	Q99KP6	Pre-mRNA-processing factor 19	2050200	3.26E+06	2126300	7.15E+04	1.72E+05	9.57E+04

Prpf8	Q99PV0	Pre-mRNA-processing-splicing factor 8	6664800	1.12E+07	9012300	6.42E+05	1.45E+06	8.71E+05
Psd3	F6Z9E6	PH and SEC7 domain-containing protein 3	1826400	2.41E+06	1176300	1.88E+05	1.83E+05	7.58E+04
Psip1	A2BI12		4019800	7.48E+06	7652200	1.17E+06	4.81E+05	5.02E+05
Ptbp2	Q91Z31	Polypyrimidine tract-binding protein 2	2967900	4.36E+06	4221400	3.81E+04	8.67E+04	0.00E+00
Ptrf	O54724	Polymerase I and transcript release factor	2159100	3.34E+06	3899200	3.55E+05	3.12E+05	2.55E+05
Pura	P42669	Transcriptional activator protein Pur-alpha	4946800	9.57E+06	7690100	1.86E+05	2.44E+06	4.71E+05
Rab10	Q4FJL0	Ras-related protein Rab-10	848280	1.09E+06	625160	4.75E+04	0.00E+00	2.18E+04
Rab2a	Q0PD65	Ras-related protein Rab-2A	2628800	3.05E+06	3667900	6.12E+04	9.78E+03	0.00E+00
Rab3a	Q0PD63	Ras-related protein Rab-3A	12591000	1.40E+07	12278000	3.40E+05	3.43E+05	1.95E+05
Rab5c	Q3TJ39	Ras-related protein Rab-5C	1826000	1.41E+06	1728200	2.07E+04	0.00E+00	0.00E+00
Rac1	Q8BPG5	Ras-related C3 botulinum toxin substrate 1	857370	3.16E+05	420070	3.38E+05	2.72E+05	3.42E+05
Rala	P63321	Ras-related protein Ral-A	1504800	1.29E+06	1059700	1.74E+04	3.44E+04	1.94E+04
Ran	Q3ULW0	GTP-binding nuclear protein Ran	761190	1.72E+06	773730	0.00E+00	3.33E+06	3.50E+05
Ranbp2	Q9ERU9	E3 SUMO-protein ligase RanBP2	4971000	7.16E+06	6228700	5.63E+05	9.39E+05	7.80E+05
Rap1a	P62835	Ras-related protein Rap-1A	2459600	2.38E+06	1314500	4.00E+04	7.93E+04	4.32E+04
Rbmxl1	A0A2I3BRL8	RNA binding motif protein, X-linked-like-1;RNA-binding motif protein, X chromosome;RNA-binding motif protein, X chromosome, N-terminally processed	3697300	5.43E+06	2630200	1.41E+07	1.17E+07	8.29E+06
Rgs7	Q80XD3	Regulator of G-protein signaling 7	731940	9.44E+05	427620	2.73E+04	0.00E+00	0.00E+00
Rhob	Q4FJM5	Rho-related GTP-binding protein RhoB	1546800	9.79E+05	1229300	0.00E+00	5.79E+04	3.20E+04
Rpl13	Q5RKP3	60S ribosomal protein L13	155950	6.89E+04	117440	3.90E+05	7.88E+05	1.91E+06
Rpl4	Q564E8	60S ribosomal protein L4	359910	7.62E+05	689710	8.58E+05	2.11E+06	1.25E+06
Rpl7	Q5M9N8	60S ribosomal protein L7	413330	4.67E+05	332330	1.17E+06	1.11E+06	1.06E+06
Rps27a	P62983	Ubiquitin-40S ribosomal protein S27a;Ubiquitin;40S ribosomal protein S27a;Polyubiquitin-C;Ubiquitin;Ubiquitin-related 1;Ubiquitin-related 2;Ubiquitin-60S ribosomal protein L40;Ubiquitin;60S ribosomal protein L40;Polyubiquitin-B;Ubiquitin	304050	7.86E+05	582160	6.46E+06	4.17E+06	2.34E+06
Rtn1	Q4FJL2	Reticulon;Reticulon-1	3107100	2.19E+06	2955500	9.66E+03	1.34E+04	0.00E+00
Rtn4	Q99P72	Reticulon-4	3190200	2.66E+06	2282500	1.39E+04	1.03E+05	0.00E+00
Ruvbl1	Q3UJN2	RuvB-like 1	422530	1.31E+06	849410	0.00E+00	5.52E+04	0.00E+00
Safb2	Q80YR5	Scaffold attachment factor B2	288070	2.23E+05	634030	5.02E+05	5.57E+05	4.13E+05
Sdha	Q8K2B3	Succinate dehydrogenase [ubiquinone] flavoprotein subunit, mitochondrial	5537800	6.36E+06	4454100	1.87E+04	0.00E+00	0.00E+00
Sdhb	Q0QEZ4	Succinate dehydrogenase [ubiquinone] iron-sulfur subunit, mitochondrial	1636400	2.41E+06	860270	0.00E+00	3.76E+03	0.00E+00

Sep-06	Q9R1T4	Septin-6	1916300	9.74E+05	498830	1.12E+04	1.15E+04	0.00E+00
Sep-07	E9Q1G8	Septin-7	10263000	7.74E+06	6347200	8.90E+04	1.59E+05	9.30E+04
Sep-08	B1AQZ0	Septin-8	2601500	2.45E+06	1812200	1.55E+04	0.00E+00	0.00E+00
Sep-11	A0A0J9YTY0	Septin-11	2765300	1.07E+06	1683800	1.73E+04	8.43E+04	2.75E+03
Sf3a1	Q8K4Z5	Splicing factor 3A subunit 1	3414500	5.19E+06	3216800	4.20E+05	5.68E+05	3.06E+05
Sf3a3	Q58E59	Splicing factor 3A subunit 3	2981300	3.58E+06	1708900	2.65E+05	1.03E+05	1.20E+05
Sf3b1	G5E866	Splicing factor 3B subunit 1	4157400	5.10E+06	4961200	1.17E+06	6.06E+05	6.25E+05
Sf3b3	B2RSV4	Splicing factor 3B subunit 3	5479200	5.12E+06	6100600	2.58E+05	4.12E+05	2.55E+05
Sfpq	Q8VIJ6	Splicing factor, proline- and glutamine-rich	8854100	1.58E+07	9340900	1.62E+05	1.44E+05	0.00E+00
Sfxn3	Q91V61	Sideroflexin-3	4660300	5.37E+06	3093400	8.58E+04	1.17E+05	6.23E+04
Sfxn5	Q925N0	Sideroflexin-5;Sideroflexin	669130	1.14E+06	522010	0.00E+00	4.45E+03	0.00E+00
Sh3gl2	A2ALV3	Endophilin-A1	4223100	2.22E+06	1782700	2.95E+04	1.04E+05	5.57E+04
Sh3glb2	A2AWI9	Endophilin-B2	273860	1.36E+05	126760	0.00E+00	2.63E+04	4.56E+03
Sirt2	Q8VDQ8	NAD-dependent protein deacetylase sirtuin-2	9319100	9.88E+06	6802400	0.00E+00	6.27E+04	2.61E+04
Slc1a2	A2APL8	Amino acid transporter;Excitatory amino acid transporter 2	23700000	1.92E+07	16330000	3.24E+04	1.75E+05	9.24E+04
Slc1a3	Q8C7W8	Amino acid transporter;Excitatory amino acid transporter 1	13477000	1.12E+07	9006100	1.21E+04	2.54E+04	0.00E+00
Slc25a11	Q9CTC7	Mitochondrial 2-oxoglutarate/malate carrier protein	1438800	1.30E+06	648850	8.88E+04	9.41E+04	8.62E+04
Slc25a12	Q8BH59	Calcium-binding mitochondrial carrier protein Aralar1	8925000	6.65E+06	6341400	1.99E+05	3.11E+05	6.98E+04
Slc25a22	Q9D6M3	Mitochondrial glutamate carrier 1	3293200	3.06E+06	2177700	1.21E+04	9.90E+03	0.00E+00
Slc25a3	Q3UB63	Phosphate carrier protein, mitochondrial	13192000	9.90E+06	5619500	3.01E+05	2.59E+05	9.02E+04
Slc25a4	Q8BVI9	ADP/ATP translocase 1	11151000	1.61E+07	11297000	1.90E+05	3.76E+05	2.27E+04
Slc25a5	Q545A2	ADP/ATP translocase 2;ADP/ATP translocase 2, N-terminally processed	2711000	3.91E+06	1974200	1.50E+05	0.00E+00	4.21E+03
Slc2a1	Q3TD17	Solute carrier family 2, facilitated glucose transporter member 1	3893800	5.81E+06	6098300	2.22E+04	0.00E+00	3.73E+04
Slc3a2	P10852	4F2 cell-surface antigen heavy chain	4390200	6.06E+06	3419300	6.03E+04	6.47E+04	2.93E+04
Slc4a4	E9Q8N8	Anion exchange protein	4612000	6.01E+06	2995100	1.71E+04	1.80E+04	0.00E+00
Slc6a1	Q6PCX2	Transporter;Sodium- and chloride-dependent GABA transporter 1	2959800	1.36E+06	1183600	4.22E+04	0.00E+00	0.00E+00
Slc6a11	P31650	Sodium- and chloride-dependent GABA transporter 3;Transporter	605240	8.10E+05	441570	0.00E+00	1.28E+04	0.00E+00
Sltm	B9EI57	SAFB-like transcription modulator	388800	9.80E+05	1224100	5.87E+05	7.15E+05	5.90E+05
Smu1	Q3UKJ7	WD40 repeat-containing protein SMU1;WD40 repeat-containing protein SMU1, N-terminally processed	593890	1.18E+06	891990	0.00E+00	1.38E+04	0.00E+00
Snap25	P60879	Synaptosomal-associated protein 25	13046000	1.40E+07	9926800	1.72E+05	1.47E+05	1.70E+05

Snap91	Q3UI39	Clathrin coat assembly protein AP180	1252400	1.27E+06	1099000	0.00E+00	4.07E+04	1.41E+04
Snrnp200	Q6P4T2	U5 small nuclear ribonucleoprotein 200 kDa helicase	4895100	9.11E+06	7033200	5.34E+05	4.10E+05	5.17E+05
Snrnp70	A2RS68	U1 small nuclear ribonucleoprotein 70 kDa	6328900	7.45E+06	7283600	4.96E+05	6.40E+05	5.60E+05
Snrpd1	P62315	Small nuclear ribonucleoprotein Sm D1	451830	3.64E+06	2659300	7.12E+04	1.62E+04	6.99E+04
Snrpd2	Q14AF6	Small nuclear ribonucleoprotein Sm D2	567830	1.02E+06	1133600	3.64E+05	3.96E+05	1.69E+05
Snrpd3	P62320	Small nuclear ribonucleoprotein Sm D3	458360	3.89E+05	268410	2.55E+05	3.14E+04	2.57E+05
Snrpf	Q497K3	Small nuclear ribonucleoprotein F	1445700	1.93E+06	1598900	5.73E+04	6.73E+04	9.35E+04
Snrpn	Q3UN87	Small nuclear ribonucleoprotein-associated protein;Small nuclear ribonucleoprotein-associated protein B;Small nuclear ribonucleoprotein-associated protein N	1818700	0.00E+00	359590	1.37E+04	0.00E+00	4.71E+04
Sptbn1	Q62261	Spectrin beta chain, non-erythrocytic 1	79486000	7.44E+07	53541000	4.86E+06	4.16E+06	3.83E+06
Sptbn2	Q68FG2		14988000	1.05E+07	8095000	1.26E+06	5.69E+05	3.81E+05
Srcin1	B1AQX6	SRC kinase signaling inhibitor 1	3516800	3.84E+06	2211200	3.59E+05	2.38E+05	2.54E+05
Srrm2	Q8BTI8	Serine/arginine repetitive matrix protein 2	2905600	3.65E+06	3282500	2.42E+06	2.45E+06	1.56E+06
Srsf1	H7BX95	Serine/arginine-rich splicing factor 1	7687500	1.23E+07	11443000	6.26E+06	7.67E+06	5.52E+06
Srsf3	Q3U781	Serine/arginine-rich splicing factor 3	6951000	1.40E+07	4336300	4.12E+06	1.76E+06	2.74E+06
Srsf7	Q3THA6	Serine/arginine-rich splicing factor 7	786120	9.18E+05	1232400	4.39E+05	4.43E+05	3.11E+05
Ssb	Q8BTU4	Lupus La protein homolog	1509200	2.93E+06	2689700	3.50E+05	7.12E+05	4.26E+05
Stx1a	Q5D0A4	Syntaxin-1A	7847300	7.37E+06	3458900	4.65E+04	2.40E+04	2.06E+04
Stx1b	P61264	Syntaxin-1B	25127000	2.03E+07	11242000	2.80E+05	2.79E+05	1.11E+05
Stxbp1	O08599	Syntaxin-binding protein 1	44682000	3.69E+07	24676000	7.30E+05	5.35E+05	4.21E+05
Sucla2	Q3UCC6	Succinyl-CoA ligase subunit beta;Succinyl-CoA ligase [ADP-forming] subunit beta, mitochondrial	2675100	3.35E+06	2653600	0.00E+00	2.05E+04	0.00E+00
Sv2a	Q9JIS5	Synaptic vesicle glycoprotein 2A	4906800	3.32E+06	1677100	6.60E+04	3.60E+04	3.28E+04
Sv2b	Q8BG39	Synaptic vesicle glycoprotein 2B	4508400	3.27E+06	1755000	1.22E+04	0.00E+00	0.00E+00
Syn1	O88935	Synapsin-1	12669000	8.10E+06	6735700	9.48E+05	1.09E+06	1.21E+06
Syn2	Q64332	Synapsin-2	10499000	5.98E+06	5428800	5.17E+05	1.00E+06	6.33E+05
Synj1	E9Q7S0	Synaptojanin-1	1431300	7.20E+05	455090	4.20E+04	8.29E+04	9.10E+03
Synpo	Q3URF1	Synaptopodin	1878300	1.37E+06	1759800	3.51E+05	3.06E+05	1.35E+05
Syp	Q62277	Synaptophysin	5668000	1.66E+06	5523300	6.88E+03	0.00E+00	0.00E+00
Syt1	Q3TPT3	Synaptotagmin-1	8624800	8.11E+06	6590100	1.05E+05	2.73E+04	3.82E+04
Tardbp	Q921F2	TAR DNA-binding protein 43	8860300	1.15E+07	12719000	7.17E+05	1.29E+06	7.64E+05
Thrap3	Q569Z6	Thyroid hormone receptor-associated protein 3	2245600	3.65E+06	3570000	3.67E+05	1.46E+05	1.06E+05
Thy1	A0A1L1SUX8	Thy-1 membrane glycoprotein	11044000	1.06E+07	10681000	7.22E+06	5.26E+06	5.44E+06
Tinagl1	H3BJ97	Tubulointerstitial nephritis antigen-like	16329	3.29E+04	61583	5.07E+06	5.99E+06	4.79E+06

Tln1	Q80TM2	Talin-1	0	6.53E+06	5498400	1.05E+05	3.24E+05	1.80E+05
Tln2	E9PUM4	Talin-2	880590	6.47E+05	988480	0.00E+00	2.32E+04	4.69E+03
Tmod2	Q9JKK7	Tropomodulin-2	1094900	1.21E+06	1188600	1.05E+05	1.25E+05	8.01E+04
Tmpo	Q3TNH0	Lamina-associated polypeptide 2, isoforms beta/delta/epsilon/gamma	3574800	4.80E+06	4353400	1.51E+04	2.38E+04	1.26E+04
Tnr	Q8BYI9	Tenascin-R	4851300	5.89E+06	3158800	7.80E+04	9.07E+04	8.60E+04
Tomm70a	Q80TT4	Mitochondrial import receptor subunit TOM70	2966600	3.05E+06	2438700	7.57E+03	8.06E+03	0.00E+00
Tppp	Q3URG1	Tubulin polymerization-promoting protein	1500800	4.81E+05	661990	9.22E+04	1.15E+05	0.00E+00
Tra2a	Q3TAP5	Transformer-2 protein homolog alpha	1376900	2.21E+06	1644800	4.58E+05	3.05E+06	1.26E+06
Tuba4a	A0A0A0MQA5	Tubulin alpha-4A chain	1694200	1.76E+06	1722000	1.36E+05	8.25E+04	5.74E+04
Tubb2a	Q7TMM9	Tubulin beta-2A chain	18420000	1.04E+07	8973200	6.78E+06	8.27E+06	6.26E+06
Tubb3	Q9ERD7	Tubulin beta-3 chain	8091200	6.35E+06	5476500	1.81E+06	2.43E+06	1.55E+06
Tubb4a	Q9D6F9	Tubulin beta-4A chain	49310000	4.20E+07	39192000	2.20E+07	3.18E+07	2.40E+07
Tubb5	P99024	Tubulin beta-5 chain	2414300	2.40E+06	0	1.30E+06	5.36E+06	8.39E+05
Tufm	Q8BFR5	Elongation factor Tu, mitochondrial	2289100	1.68E+06	1694800	7.22E+04	1.02E+05	7.27E+04
U2af2	Q505Q1	Splicing factor U2AF 65 kDa subunit	1964200	2.62E+06	2891800	1.15E+05	1.21E+06	1.63E+05
Uba1	B9EHN0	Ubiquitin-like modifier-activating enzyme 1	3482200	1.91E+06	1320900	0.00E+00	6.44E+03	0.00E+00
Uqcrc1	Q3TIC8	Cytochrome b-c1 complex subunit 1, mitochondrial	8514800	8.56E+06	7076400	1.76E+04	0.00E+00	0.00E+00
Uqcrc2	Q9DB77	Cytochrome b-c1 complex subunit 2, mitochondrial	0	2.67E+07	19879000	8.58E+04	5.48E+04	6.29E+04
Uqcrfs1	Q9CR68	Cytochrome b-c1 complex subunit Rieske, mitochondrial;Cytochrome b-c1 complex subunit 11	1371700	2.22E+06	1043400	0.00E+00	2.26E+03	0.00E+00
Vapb	Q8BH80	Vesicle-associated membrane protein-associated protein B	3064200	3.41E+06	1811400	1.67E+03	0.00E+00	0.00E+00
Vcl	Q64727	Vinculin	3073400	3.41E+06	3831700	0.00E+00	6.74E+04	1.10E+05
Vdac1	Q60932	Voltage-dependent anion-selective channel protein 1	21429000	2.08E+07	17021000	5.44E+05	2.99E+05	9.10E+04
Vim	Q5FWJ3	Vimentin	19824000	3.70E+07	47674000	1.55E+07	1.67E+07	1.38E+07
Vsnl1	Q4W4C9	Visinin-like protein 1	7689800	6.32E+06	4816600	0.00E+00	3.25E+04	3.66E+04
Ywhab	Q9CQV8	14-3-3 protein beta/alpha;14-3-3 protein beta/alpha, N-terminally processed	4406500	2.04E+06	1697400	9.10E+04	2.43E+04	2.16E+04
Ywhae	Q5SS40	14-3-3 protein epsilon	2283100	1.81E+06	2664400	0.00E+00	1.48E+04	0.00E+00
Ywhag	A8IP69	14-3-3 protein gamma;14-3-3 protein gamma, N-terminally processed	7743400	5.45E+06	3588900	2.69E+05	3.59E+05	2.14E+05
Ywhah	P68510	14-3-3 protein eta	2960400	2.01E+06	1856000	1.53E+05	0.00E+00	0.00E+00
Ywhaq	A3KML3	14-3-3 protein theta	2005900	5.59E+05	1256900	5.49E+04	6.96E+04	3.51E+04
Ywhaz	P63101	14-3-3 protein zeta/delta	15610000	1.33E+07	6169700	5.28E+05	3.45E+05	3.31E+05
Abat	P61922	4-aminobutyrate aminotransferase, mitochondrial	7528500	6.41E+06	5171100	0.00E+00	0.00E+00	0.00E+00

Abcb1a	P21447	Multidrug resistance protein 1A	1512900	2.21E+06	1972400	0.00E+00	0.00E+00	0.00E+00
Acadl	A0A0R4J083	Long-chain specific acyl-CoA dehydrogenase, mitochondrial	1135400	1.02E+06	489930	0.00E+00	0.00E+00	0.00E+00
Acat1	Q3TQP7	Acetyl-CoA acetyltransferase, mitochondrial	7758900	8.76E+06	6775000	0.00E+00	0.00E+00	0.00E+00
Acot7	E9PYH2	Cytosolic acyl coenzyme A thioester hydrolase	97105	2.53E+05	12006	0.00E+00	0.00E+00	0.00E+00
Acsl6	Q5ICG5		2201700	3.05E+06	2724600	0.00E+00	0.00E+00	0.00E+00
Actr1a	P61164	Alpha-centractin	98454	1.98E+05	104310	0.00E+00	0.00E+00	0.00E+00
Actr2	P61161	Actin-related protein 2	641970	2.45E+05	899500	0.00E+00	0.00E+00	0.00E+00
Actr3	Q3ULF7	Actin-related protein 3	1652800	1.36E+06	1623300	0.00E+00	0.00E+00	0.00E+00
Adam22	A4FUT9	Disintegrin and metalloproteinase domain-containing protein 22	1989300	2.29E+06	1498900	0.00E+00	0.00E+00	0.00E+00
Adam23	Q9R1V7	Disintegrin and metalloproteinase domain-containing protein 23	1001500	9.24E+05	799300	0.00E+00	0.00E+00	0.00E+00
Ak3	Q9WTP7	GTP:AMP phosphotransferase AK3, mitochondrial	1347400	1.09E+06	1393700	0.00E+00	0.00E+00	0.00E+00
Akr1a1	Q80XJ7	Alcohol dehydrogenase [NADP(+)]	312260	2.98E+04	144220	0.00E+00	0.00E+00	0.00E+00
Alb	P07724	Serum albumin	315790	2.43E+05	264610	0.00E+00	0.00E+00	0.00E+00
Alcam	E9Q4G8	CD166 antigen	945610	1.41E+06	1149300	0.00E+00	0.00E+00	0.00E+00
Aldh7a1	Q9DBF1	Alpha-aminoadipic semialdehyde dehydrogenase	638270	6.40E+05	303700	0.00E+00	0.00E+00	0.00E+00
Ap2a2	Q6PEE6	AP-2 complex subunit alpha-2	10748000	9.90E+06	3883400	0.00E+00	0.00E+00	0.00E+00
Ap2s1	Q3UJ76	AP-2 complex subunit sigma	366210	8.12E+05	201220	0.00E+00	0.00E+00	0.00E+00
Apoe	Q6GTX3	Apolipoprotein E	482220	3.17E+05	455550	0.00E+00	0.00E+00	0.00E+00
Arhgdia	Q99PT1	Rho GDP-dissociation inhibitor 1	1344900	9.27E+05	345460	0.00E+00	0.00E+00	0.00E+00
Arpc1a	Q9R0Q6	Actin-related protein 2/3 complex subunit 1A	502280	2.92E+05	0	0.00E+00	0.00E+00	0.00E+00
Arpc4	Q9D3C4	Actin-related protein 2/3 complex subunit 4	404190	4.62E+05	266690	0.00E+00	0.00E+00	0.00E+00
Atl1	Q8BH66	Atlastin-1	738640	8.29E+05	581310	0.00E+00	0.00E+00	0.00E+00
Atp1b2	P14231	Sodium/potassium-transporting ATPase subunit beta-2	2477200	4.97E+06	6063400	0.00E+00	0.00E+00	0.00E+00
Atp5f1	Q5I0W0	ATP synthase F(0) complex subunit B1, mitochondrial	3352500	3.65E+06	2248300	0.00E+00	0.00E+00	0.00E+00
Atp5i	Q06185	ATP synthase subunit e, mitochondrial	197080	4.09E+05	304170	0.00E+00	0.00E+00	1.46E+04
Atp5l	Q9D037	ATP synthase subunit g, mitochondrial	1420500	1.23E+06	1086000	0.00E+00	0.00E+00	0.00E+00
Atp5o	Q9DB20	ATP synthase subunit O, mitochondrial	6325100	4.78E+06	4427800	0.00E+00	0.00E+00	0.00E+00
Atp6v1c1	Q9D9Z4	V-type proton ATPase subunit C 1	1923100	1.15E+06	1098000	0.00E+00	0.00E+00	0.00E+00
Atp6v1d	Q3UK81	V-type proton ATPase subunit D	735750	1.30E+06	1264300	0.00E+00	0.00E+00	0.00E+00
Atp6v1h	A0A0A6YX18	V-type proton ATPase subunit H	2171600	1.61E+06	1040000	0.00E+00	0.00E+00	0.00E+00
Bin1	O08539	Myc box-dependent-interacting protein 1	1462000	1.09E+06	363300	0.00E+00	0.00E+00	0.00E+00
Ca2	P00920	Carbonic anhydrase 2	743390	7.04E+05	597850	0.00E+00	0.00E+00	0.00E+00

Cadm1	Q1WIL9	Cell adhesion molecule 1	1932300	1.88E+06	887000	0.00E+00	0.00E+00	0.00E+00
Cadm2	Q8BLQ9	Cell adhesion molecule 2	3319700	4.12E+06	1021500	0.00E+00	0.00E+00	0.00E+00
Cadm3	K4DI58	Cell adhesion molecule 3	1508100	1.88E+06	1242900	0.00E+00	0.00E+00	0.00E+00
Cadm4	Q8R464	Cell adhesion molecule 4	1215900	9.98E+05	530470	0.00E+00	0.00E+00	0.00E+00
Calm1	P0DP28		23958000	1.79E+07	8293900	0.00E+00	0.00E+00	0.00E+00
Calr	B2MWM9	Calreticulin	2089100	1.67E+06	1458100	0.00E+00	0.00E+00	0.00E+00
Canx	P35564	Calnexin	5208900	4.94E+06	5550000	0.00E+00	0.00E+00	0.00E+00
Cap1	Q3UVJ2	Adenylyl cyclase-associated protein;Adenylyl cyclase-associated protein 1	0	9.67E+05	724570	0.00E+00	0.00E+00	0.00E+00
Cbr1	B2RXY7	Carbonyl reductase [NADPH] 1	228040	1.69E+05	182200	0.00E+00	0.00E+00	0.00E+00
Cct2	Q542X7	T-complex protein 1 subunit beta	819580	5.60E+05	457520	0.00E+00	0.00E+00	0.00E+00
Cd81	P35762	CD81 antigen;Tetraspanin	6246800	4.29E+06	4807200	0.00E+00	0.00E+00	0.00E+00
Cd9	P40240	CD9 antigen	136730	3.39E+05	112200	0.00E+00	0.00E+00	0.00E+00
Cdc42	P60766	Cell division control protein 42 homolog	230070	3.21E+05	538800	0.00E+00	0.00E+00	0.00E+00
Cend1	Q9JKC6	Cell cycle exit and neuronal differentiation protein 1	34693	3.86E+04	46160	0.00E+00	0.00E+00	0.00E+00
Cfl1	Q544Y7	Cofilin-1	3531500	2.65E+06	1720100	0.00E+00	0.00E+00	0.00E+00
Cisd1	Q91WS0	CDGSH iron-sulfur domain-containing protein 1	268730	7.39E+05	906760	0.00E+00	0.00E+00	0.00E+00
Clta	B1AWE0	Clathrin light chain A	254010	2.24E+05	22230	0.00E+00	0.00E+00	0.00E+00
Cltb	Q6IRU5	Clathrin light chain B	74270	1.81E+04	171760	0.00E+00	0.00E+00	0.00E+00
Coro1a	Q3U9K3	Coronin;Coronin-1A	956180	4.50E+05	360700	0.00E+00	0.00E+00	0.00E+00
Coro1c	Q5PPQ7	Coronin;Coronin-1C	262540	9.14E+04	43269	0.00E+00	0.00E+00	0.00E+00
Coro2b	Q8BH44	Coronin-2B;Coronin	200180	5.70E+05	70153	0.00E+00	0.00E+00	0.00E+00
Cox5a	P12787	Cytochrome c oxidase subunit 5A, mitochondrial	2559000	2.88E+06	4203500	0.00E+00	0.00E+00	0.00E+00
Cox5b	Q9D881	Cytochrome c oxidase subunit 5B, mitochondrial	5145800	4.04E+06	2239200	0.00E+00	0.00E+00	0.00E+00
Cox6b1	P56391	Cytochrome c oxidase subunit 6B1	998610	8.57E+05	953900	0.00E+00	0.00E+00	0.00E+00
Cpn10-rs1	Q9JI95	10 kDa heat shock protein, mitochondrial	1307200	7.35E+05	992290	0.00E+00	0.00E+00	0.00E+00
Crym	Q3UPX0	Ketimine reductase mu-crystallin	631410	4.41E+05	300210	0.00E+00	0.00E+00	0.00E+00
Cs	Q9CZU6	Citrate synthase, mitochondrial;Citrate synthase	3912100	3.54E+06	2645300	0.00E+00	0.00E+00	0.00E+00
Csrp1	Q4FJX4	Cysteine and glycine-rich protein 1	575990	3.39E+05	166850	0.00E+00	0.00E+00	0.00E+00
Ctbp1	A0A0J9YU62	C-terminal-binding protein 1	176900	5.03E+04	78997	0.00E+00	0.00E+00	0.00E+00
Cyc1	Q9D0M3	Cytochrome c1, heme protein, mitochondrial	3713900	5.32E+06	2286000	0.00E+00	0.00E+00	0.00E+00
D10Jhu81e	Q9D172	ES1 protein homolog, mitochondrial	211460	6.13E+05	594600	0.00E+00	0.00E+00	0.00E+00
Dctn2	Q3TPZ5	Dynactin subunit 2	976940	9.57E+05	693610	0.00E+00	0.00E+00	0.00E+00
Ddah1	D3YU15	N(G),N(G)-dimethylarginine dimethylaminohydrolase 1	1133500	6.41E+05	398850	0.00E+00	0.00E+00	0.00E+00
Dmxl2	B0V2P5	DmX-like protein 2	1359600	4.78E+05	96211	0.00E+00	0.00E+00	0.00E+00

Dnajc5	P60904	DnaJ homolog subfamily C member 5	752800	7.19E+05	697740	0.00E+00	0.00E+00	0.00E+00
Dnajc6	Q80TZ3	Putative tyrosine-protein phosphatase auxilin	404000	1.12E+05	149940	0.00E+00	0.00E+00	0.00E+00
Dnm1l	Q8K1M6	Dynamin-1-like protein	1755200	1.27E+06	988730	0.00E+00	0.00E+00	0.00E+00
Dpp6	Q80VM5	Dipeptidyl aminopeptidase-like protein 6	638050	6.62E+05	575280	0.00E+00	0.00E+00	0.00E+00
Eef2	P58252	Elongation factor 2	792050	4.39E+05	516170	0.00E+00	0.00E+00	1.07E+04
Efhd2	Q8C845	EF-hand domain-containing protein D2	689430	4.81E+05	288070	0.00E+00	0.00E+00	0.00E+00
Epb4.1l1	A2AUK8	Band 4.1-like protein 1	1201800	1.15E+06	750450	0.00E+00	0.00E+00	0.00E+00
Etfa	Q99LC5	Electron transfer flavoprotein subunit alpha, mitochondrial	1639700	1.54E+06	1969800	0.00E+00	0.00E+00	0.00E+00
Etfb	Q9DCW4	Electron transfer flavoprotein subunit beta	507070	7.66E+05	860430	0.00E+00	0.00E+00	0.00E+00
Etfdh	Q6PF96	Electron transfer flavoprotein-ubiquinone oxidoreductase, mitochondrial	738100	2.53E+05	1109100	0.00E+00	0.00E+00	0.00E+00
Fam213a	Q3U125	Redox-regulatory protein FAM213A	1201200	1.11E+06	1275100	0.00E+00	0.00E+00	0.00E+00
Fam49b	Q921M7	Protein FAM49B	758060	4.36E+05	378570	0.00E+00	0.00E+00	0.00E+00
Fscn1	Q61553	Fascin	459880	3.78E+05	242350	0.00E+00	0.00E+00	0.00E+00
Gbas	Q3TD78	Protein NipSnap homolog 2	1274900	0.00E+00	84612	0.00E+00	0.00E+00	0.00E+00
Gda	Q548F2	Guanine deaminase	678630	2.58E+05	240560	0.00E+00	0.00E+00	0.00E+00
Gdi1	P50396	Rab GDP dissociation inhibitor alpha	7749700	3.95E+06	3009600	0.00E+00	0.00E+00	0.00E+00
Gdi2	Q3UUX9	Rab GDP dissociation inhibitor beta	1449700	6.43E+05	546020	0.00E+00	0.00E+00	0.00E+00
Gls	D3Z7P3	Glutaminase kidney isoform, mitochondrial	3220200	2.67E+06	1892900	0.00E+00	0.00E+00	0.00E+00
Gm20390	E9PZF0	Nucleoside diphosphate kinase;Nucleoside diphosphate kinase B	63680	5.17E+04	144820	0.00E+00	0.00E+00	0.00E+00
Gna11	Q91X95	Guanine nucleotide-binding protein subunit alpha-11	574950	6.36E+05	263200	0.00E+00	0.00E+00	0.00E+00
Gnb2	Q3U9V4	Guanine nucleotide-binding protein G(I)/G(S)/G(T) subunit beta-2	7404000	7.38E+06	4697000	0.00E+00	0.00E+00	0.00E+00
Got1	P05201	Aspartate aminotransferase, cytoplasmic	4381900	2.95E+06	2630000	0.00E+00	0.00E+00	0.00E+00
Gpm6b	A2AEG6	Neuronal membrane glycoprotein M6-b	1280700	5.97E+05	894570	0.00E+00	0.00E+00	0.00E+00
Grm3	Q9QYS2	Metabotropic glutamate receptor 3	1156000	1.13E+06	1046600	0.00E+00	0.00E+00	0.00E+00
Gstm1	P10649	Glutathione S-transferase Mu 1	1918600	1.49E+06	1325200	0.00E+00	0.00E+00	0.00E+00
Gstp1	P19157	Glutathione S-transferase P 1;Glutathione S-transferase P 2	3068600	1.45E+06	1156400	0.00E+00	0.00E+00	0.00E+00
Hadh	Q61425	Hydroxyacyl-coenzyme A dehydrogenase, mitochondrial	392620	4.18E+05	168100	0.00E+00	0.00E+00	0.00E+00
haemaglobin alpha 2	Q9CY10	Hemoglobin subunit alpha	0	1.55E+05	0	0.00E+00	0.00E+00	0.00E+00
Hepacam	B2RSY3	Hepatocyte cell adhesion molecule	1474400	1.74E+06	1627300	0.00E+00	0.00E+00	0.00E+00
Hibadh	Q99L13	3-hydroxyisobutyrate dehydrogenase, mitochondrial;3-hydroxyisobutyrate dehydrogenase	571770	6.03E+05	746130	0.00E+00	0.00E+00	0.00E+00

Hsd17b10	Q99N15	3-hydroxyacyl-CoA dehydrogenase type-2	917420	8.48E+05	717110	0.00E+00	0.00E+00	0.00E+00
Hspa4	Q3U2G2	Heat shock 70 kDa protein 4	3427600	1.87E+06	1559600	0.00E+00	0.00E+00	0.00E+00
Hspg2	E9PZ16	Basement membrane-specific heparan sulfate proteoglycan core protein;Endorepellin;LG3 peptide	519350	1.19E+06	620230	0.00E+00	0.00E+00	0.00E+00
Htra1	Q9R118	Serine protease HTRA1	13073	1.19E+04	0	0.00E+00	0.00E+00	1.73E+04
Iars2	Q8BIJ6	Isoleucine--tRNA ligase, mitochondrial	606260	6.77E+05	672180	0.00E+00	0.00E+00	0.00E+00
Idh3g	Q3TKM5	Isocitrate dehydrogenase [NAD] subunit, mitochondrial;Isocitrate dehydrogenase [NAD] subunit gamma 1, mitochondrial	1863100	1.91E+06	1547900	0.00E+00	0.00E+00	0.00E+00
Igsf8	G3UYZ1	Immunoglobulin superfamily member 8	1369100	8.95E+05	982220	0.00E+00	0.00E+00	0.00E+00
Isoc2a	B2RY90	Isochorismatase domain-containing protein 2A, mitochondrial	99135	0.00E+00	32298	0.00E+00	0.00E+00	0.00E+00
Kcnab2	P62482	Voltage-gated potassium channel subunit beta-2	1631900	0.00E+00	808210	0.00E+00	0.00E+00	0.00E+00
Kctd16	Q5DTY9	BTB/POZ domain-containing protein KCTD16	198490	1.98E+05	127540	0.00E+00	0.00E+00	0.00E+00
Letm1	Q9Z2I0	LETM1 and EF-hand domain-containing protein 1, mitochondrial	1030400	1.21E+06	1254600	0.00E+00	0.00E+00	0.00E+00
Mag	A0A087WPR1	Myelin-associated glycoprotein	6807800	8.64E+06	5615400	0.00E+00	0.00E+00	2.18E+04
Maoa	Q3TPD9	Amine oxidase [flavin-containing] A	2270800	3.15E+06	2085000	0.00E+00	0.00E+00	0.00E+00
Mapk1	Q3UF82	Mitogen-activated protein kinase;Mitogen-activated protein kinase 1	34129	9.48E+04	31851	0.00E+00	0.00E+00	0.00E+00
Mapt	A0A0A0MQC7	Microtubule-associated protein;Microtubule-associated protein tau	2453400	1.13E+06	810700	0.00E+00	0.00E+00	0.00E+00
Mar-02	Q922Q1	Mitochondrial amidoxime reducing component 2	182620	4.41E+05	604430	0.00E+00	0.00E+00	0.00E+00
Mpc2	Q9D023	Mitochondrial pyruvate carrier 2	110380	1.43E+05	62545	0.00E+00	0.00E+00	0.00E+00
mt-Co2	Q7JCZ1	Cytochrome c oxidase subunit 2	4158500	5.57E+06	2465300	0.00E+00	0.00E+00	0.00E+00
Mtch1	Q791T5	Mitochondrial carrier homolog 1	211350	1.85E+05	33068	0.00E+00	0.00E+00	0.00E+00
Mtch2	Q3UA17	Mitochondrial carrier homolog 2	1210200	1.51E+06	1192600	0.00E+00	0.00E+00	1.61E+03
Napg	D3Z4B2	Gamma-soluble NSF attachment protein	1933300	1.37E+06	1443400	0.00E+00	0.00E+00	0.00E+00
Ncam2	O35136	Neural cell adhesion molecule 2	960340	1.04E+06	845220	0.00E+00	0.00E+00	0.00E+00
ND4	Q9ME04	NADH-ubiquinone oxidoreductase chain 4	39436	6.58E+04	23407	0.00E+00	0.00E+00	0.00E+00
Ndrg1	Q545R3	Protein NDRG1	1977900	2.33E+06	1360900	0.00E+00	0.00E+00	2.04E+04
Ndrg2	Q9QYG0	Protein NDRG2	1631400	7.32E+05	631840	0.00E+00	0.00E+00	0.00E+00
Ndufa10	Q99LC3	NADH dehydrogenase [ubiquinone] 1 alpha subcomplex subunit 10, mitochondrial	2200800	1.71E+06	1619000	0.00E+00	0.00E+00	0.00E+00
Ndufa11	G5E814	NADH dehydrogenase [ubiquinone] 1 alpha subcomplex subunit 11	357710	3.87E+05	45760	0.00E+00	0.00E+00	0.00E+00
Ndufa12	Q8BME2	NADH dehydrogenase [ubiquinone] 1 alpha subcomplex subunit 12	1556200	1.88E+06	1223800	0.00E+00	0.00E+00	0.00E+00

Ndufa13	Q9ERS2	NADH dehydrogenase [ubiquinone] 1 alpha subcomplex subunit 13	943840	1.88E+06	1455700	0.00E+00	0.00E+00	0.00E+00
Ndufa6	Q9CQZ5	NADH dehydrogenase [ubiquinone] 1 alpha subcomplex subunit 6	640410	4.40E+05	609360	0.00E+00	0.00E+00	0.00E+00
Ndufa7	A0A068BGR9	NADH dehydrogenase [ubiquinone] 1 alpha subcomplex subunit 7	73086	4.00E+04	126780	0.00E+00	0.00E+00	0.00E+00
Ndufa8	Q9DCJ5	NADH dehydrogenase [ubiquinone] 1 alpha subcomplex subunit 8	1326300	1.24E+06	742620	0.00E+00	0.00E+00	0.00E+00
Ndufa9	A0A0R3P9C8	NADH dehydrogenase [ubiquinone] 1 alpha subcomplex subunit 9, mitochondrial	4571000	4.42E+06	3585100	0.00E+00	0.00E+00	0.00E+00
Ndufb10	Q9DCS9	NADH dehydrogenase [ubiquinone] 1 beta subcomplex subunit 10	162720	4.29E+05	233070	0.00E+00	0.00E+00	0.00E+00
Ndufb4	Q9CQC7	NADH dehydrogenase [ubiquinone] 1 beta subcomplex subunit 4	960830	1.43E+06	955010	0.00E+00	0.00E+00	0.00E+00
Ndufb9	Q9CQJ8	NADH dehydrogenase [ubiquinone] 1 beta subcomplex subunit 9	327010	2.59E+04	842540	0.00E+00	0.00E+00	0.00E+00
Ndufs5	B1ARW4	NADH dehydrogenase [ubiquinone] iron-sulfur protein 5;NADH dehydrogenase [ubiquinone] iron-sulfur protein 5, N-terminally processed	1277700	1.80E+06	1173000	0.00E+00	0.00E+00	0.00E+00
Ndufs6	P52503	NADH dehydrogenase [ubiquinone] iron-sulfur protein 6, mitochondrial	111460	4.91E+05	249340	0.00E+00	0.00E+00	0.00E+00
Ndufs8	Q8VC72	NADH dehydrogenase [ubiquinone] iron-sulfur protein 8, mitochondrial	896020	6.88E+05	439060	0.00E+00	0.00E+00	0.00E+00
Ndufv1	D3YUM1	NADH dehydrogenase [ubiquinone] flavoprotein 1, mitochondrial	3237000	3.88E+06	1692800	0.00E+00	0.00E+00	0.00E+00
Ndufv2	Q9D6J6	NADH dehydrogenase [ubiquinone] flavoprotein 2, mitochondrial	2527700	3.73E+06	1811800	0.00E+00	0.00E+00	0.00E+00
Nipsnap1	Q5SVF7	Protein NipSnap homolog 1	416540	1.61E+05	75527	0.00E+00	0.00E+00	0.00E+00
Nlgn2	Q69ZK9	Neuroligin-2	194560	1.85E+05	132670	0.00E+00	0.00E+00	0.00E+00
Nme1	P15532	Nucleoside diphosphate kinase A;Nucleoside diphosphate kinase	2458500	2.33E+06	2401300	0.00E+00	0.00E+00	8.17E+03
Nudt21	Q9CQF3	Cleavage and polyadenylation specificity factor subunit 5	433120	1.84E+05	447570	0.00E+00	0.00E+00	0.00E+00
Oxct1	Q9D0K2	Succinyl-CoA:3-ketoacid coenzyme A transferase 1, mitochondrial;Succinyl-CoA:3-ketoacid-coenzyme A transferase	1254700	1.39E+06	1314100	0.00E+00	0.00E+00	0.00E+00
P4hb	Q3UDR2	Protein disulfide-isomerase	1794400	1.74E+06	1319500	0.00E+00	0.00E+00	0.00E+00
Park7	A2A815	Protein deglycase DJ-1	911310	7.13E+05	261130	0.00E+00	0.00E+00	0.00E+00
Pcsk1n	Q9QXV0	ProSAAS;KEP;Big SAAS;Little SAAS;Big PEN-LEN;PEN;PEN-20;PEN-19;Little LEN;Big LEN	1780600	1.04E+06	769200	0.00E+00	0.00E+00	0.00E+00
Pdhb	Q9D051	Pyruvate dehydrogenase E1 component subunit beta,	5328500	8.38E+06	6838800	0.00E+00	0.00E+00	0.00E+00

		mitochondrial						
Pdia3	P27773	Protein disulfide-isomerase A3	4971300	4.58E+06	4017000	0.00E+00	0.00E+00	0.00E+00
Pebp1	Q5EBQ2	Phosphatidylethanolamine-binding protein 1;Hippocampal cholinergic neurostimulating peptide	1084700	8.52E+05	547440	0.00E+00	0.00E+00	1.26E+04
Pfkm	P47857	ATP-dependent 6-phosphofructokinase, muscle type;ATP-dependent 6-phosphofructokinase	1739500	1.19E+06	800210	0.00E+00	0.00E+00	0.00E+00
Pfn2	D3YWS3	Profilin;Profilin-2	1315400	8.99E+05	864090	0.00E+00	0.00E+00	5.66E+04
Pgam1	Q3U7Z6	Phosphoglycerate mutase 1	3649100	2.93E+06	2638900	0.00E+00	0.00E+00	0.00E+00
Pgrmc1	Q3TFP8	Membrane-associated progesterone receptor component 1	2589200	2.79E+06	2678500	0.00E+00	0.00E+00	0.00E+00
Phb2	Q3V235	Prohibitin-2	1066700	8.98E+05	1473900	0.00E+00	0.00E+00	0.00E+00
Pip4k2a	Q9CZS7	Phosphatidylinositol 5-phosphate 4-kinase type-2 alpha	552790	6.14E+05	426240	0.00E+00	0.00E+00	0.00E+00
Pip5k1c	F8WHW6	Phosphatidylinositol 4-phosphate 5-kinase type-1 gamma	325670	1.39E+05	61794	0.00E+00	0.00E+00	0.00E+00
Plcb1	Q2M4J2	Phosphoinositide phospholipase C;1-phosphatidylinositol 4,5-bisphosphate phosphodiesterase beta-1	1321900	1.10E+06	471120	0.00E+00	0.00E+00	0.00E+00
Ppp2r1a	Q76MZ3	Serine/threonine-protein phosphatase 2A 65 kDa regulatory subunit A alpha isoform	2206800	1.45E+06	2048900	0.00E+00	0.00E+00	0.00E+00
Ppp3cb	E0CZ78	Serine/threonine-protein phosphatase;Serine/threonine-protein phosphatase 2B catalytic subunit beta isoform	523160	2.43E+05	176600	0.00E+00	0.00E+00	0.00E+00
Ppp3r1	Q63810	Calcineurin subunit B type 1	1193000	6.89E+05	1474300	0.00E+00	0.00E+00	0.00E+00
Prdx1	P35700	Peroxiredoxin-1	725350	8.27E+05	109450	0.00E+00	0.00E+00	0.00E+00
Prdx2	Q61171	Peroxiredoxin-2	429240	5.15E+05	213350	0.00E+00	0.00E+00	0.00E+00
Prdx3	P20108	Thioredoxin-dependent peroxide reductase, mitochondrial	746780	6.49E+05	358290	0.00E+00	0.00E+00	0.00E+00
Prdx5	Q3UWS9	Peroxiredoxin-5, mitochondrial	3603300	2.14E+06	3175600	0.00E+00	0.00E+00	0.00E+00
Prdx6	Q6GT24	Peroxiredoxin-6	735040	4.55E+05	203210	0.00E+00	0.00E+00	0.00E+00
Prkar2a	A0A0A6YX73	cAMP-dependent protein kinase type II-alpha regulatory subunit	2016300	1.74E+06	1324800	0.00E+00	0.00E+00	0.00E+00
Prkcb	P68404	Protein kinase C beta type	4007600	2.89E+06	2019800	0.00E+00	0.00E+00	0.00E+00
Prrt2	E9PUL5	Proline-rich transmembrane protein 2	1342200	1.34E+06	953660	0.00E+00	0.00E+00	0.00E+00
Psat1	Q3U6K9	Phosphoserine aminotransferase	132150	1.91E+05	32078	0.00E+00	0.00E+00	0.00E+00
Pspc1	Q8R326	Paraspeckle component 1	1571600	1.60E+06	1575400	0.00E+00	0.00E+00	0.00E+00
Pygb	Q3V3U0	Alpha-1,4 glucan phosphorylase;Glycogen phosphorylase, brain form	1419200	8.04E+05	500550	0.00E+00	0.00E+00	0.00E+00
Qdpr	D3YWR7	Dihydropteridine reductase	708970	7.09E+05	445800	0.00E+00	0.00E+00	0.00E+00
Rab7	Q4FJQ0	Ras-related protein Rab-7a	886950	1.29E+06	870730	0.00E+00	0.00E+00	0.00E+00
Rangap1	Q91YS2	Ran GTPase-activating protein 1	981130	2.16E+06	1261700	0.00E+00	0.00E+00	9.58E+04

Reep5	Q9CQG4	Receptor expression-enhancing protein;Receptor expression-enhancing protein 5	130330	7.39E+04	41319	0.00E+00	0.00E+00	0.00E+00
Rhoa	A0A0A6YXF6	Transforming protein RhoA;Rho-related GTP-binding protein RhoC	374900	3.49E+05	80103	0.00E+00	0.00E+00	0.00E+00
Rph3a	Q768S5	Rabphilin-3A	1507500	7.24E+05	610040	0.00E+00	0.00E+00	0.00E+00
Rufy3	A0A0G2JFT8	Protein RUFY3	85654	8.41E+04	70945	0.00E+00	0.00E+00	1.37E+04
Sacm1l	Q9EP69	Phosphatidylinositide phosphatase SAC1	811630	8.49E+05	463310	0.00E+00	0.00E+00	0.00E+00
Scrn1	Q9CZC8	Secernin-1	155400	5.12E+04	182660	0.00E+00	0.00E+00	0.00E+00
Sec22b	E9Q6R3	Vesicle-trafficking protein SEC22b	1693300	2.33E+06	1711300	0.00E+00	0.00E+00	0.00E+00
Sep-03	Q9Z1S5	Neuronal-specific septin-3	724610	5.23E+05	578950	0.00E+00	0.00E+00	0.00E+00
Sep-05	Q9Z2Q6	Septin-5	4357100	2.31E+06	2571200	0.00E+00	0.00E+00	9.60E+03
Sfxn1	Q99JR1	Sideroflexin-1	868370	8.66E+05	916320	0.00E+00	0.00E+00	0.00E+00
Sirpa	Q6P6I8		1246500	1.19E+06	1315400	0.00E+00	0.00E+00	0.00E+00
Slc12a5	A0A076FRG6	Solute carrier family 12 member 5	5767200	5.52E+06	4182900	0.00E+00	0.00E+00	0.00E+00
Slc17a7	A0A1B0GRU0	Vesicular glutamate transporter 1	2604300	2.23E+06	1284700	0.00E+00	0.00E+00	0.00E+00
Slc1a4	Q9ESU8	Amino acid transporter;Neutral amino acid transporter A	390550	4.48E+05	675320	0.00E+00	0.00E+00	0.00E+00
Slc25a18	Q9DB41	Mitochondrial glutamate carrier 2	481940	6.43E+05	484210	0.00E+00	0.00E+00	0.00E+00
Slc2a3	Q8BLF7	Solute carrier family 2, facilitated glucose transporter member 3	1117600	8.38E+05	169330	0.00E+00	0.00E+00	0.00E+00
Slc32a1	O35633	Vesicular inhibitory amino acid transporter	144730	3.13E+04	30387	0.00E+00	0.00E+00	0.00E+00
Slc44a1	A2AMH5	Choline transporter-like protein 1	1153200	1.28E+06	658710	0.00E+00	0.00E+00	0.00E+00
Slc4a10	Q5DTL9	Sodium-driven chloride bicarbonate exchanger;Anion exchange protein	483010	3.64E+05	883590	0.00E+00	0.00E+00	0.00E+00
Slc6a17	A0A0R4J087	Sodium-dependent neutral amino acid transporter SLC6A17;Transporter	675970	4.11E+05	268240	0.00E+00	0.00E+00	0.00E+00
Slc7a5	Q9Z127	Large neutral amino acids transporter small subunit 1	524180	4.49E+05	614740	0.00E+00	0.00E+00	0.00E+00
Slc8a2	Q8K596		1679300	1.84E+06	1075700	0.00E+00	0.00E+00	0.00E+00
Slc9a3r1	P70441	Na(+)/H(+) exchange regulatory cofactor NHE-RF1;Na(+)/H(+) exchange regulatory cofactor NHE-RF	434090	8.24E+05	1136800	0.00E+00	0.00E+00	0.00E+00
Slc9a3r2	A0A0R4IZX2	Na(+)/H(+) exchange regulatory cofactor NHE-RF2;Na(+)/H(+) exchange regulatory cofactor NHE-RF	592510	2.57E+05	693030	0.00E+00	0.00E+00	0.00E+00
Snca	O55042	Alpha-synuclein	2064900	7.84E+05	504590	0.00E+00	0.00E+00	0.00E+00
Sncb	Q91ZZ3	Beta-synuclein	2113300	1.46E+06	895950	0.00E+00	0.00E+00	0.00E+00
Sod2	Q4FJX9	Superoxide dismutase;Superoxide dismutase [Mn], mitochondrial	1991200	1.42E+06	1531100	0.00E+00	0.00E+00	0.00E+00
Stip1	Q3THQ5	Stress-induced-phosphoprotein 1	1326400	6.72E+05	816830	0.00E+00	0.00E+00	0.00E+00
Stx12	Q9ER00	Syntaxin-12	672690	8.39E+05	270280	0.00E+00	0.00E+00	0.00E+00

Suclg1	Q9WUM5	Succinyl-CoA ligase [ADP/GDP-forming] subunit alpha, mitochondrial	1836200	2.21E+06	1363700	0.00E+00	0.00E+00	0.00E+00
Syngr1	Q3U6D7	Synaptogyrin-1	413980	7.09E+05	121400	0.00E+00	0.00E+00	0.00E+00
Tagln3	Q9R1Q8	Transgelin-3;Transgelin	197880	1.97E+05	68086	0.00E+00	0.00E+00	0.00E+00
Timm9	Q9WV98	Mitochondrial import inner membrane translocase subunit Tim9	53221	0.00E+00	55040	0.00E+00	0.00E+00	0.00E+00
Tkt	P40142	Transketolase	1084200	9.36E+05	853030	0.00E+00	0.00E+00	0.00E+00
Tmem33	A0A0R4J1Z3	Transmembrane protein 33	0	7.58E+03	11340	0.00E+00	0.00E+00	0.00E+00
Tom1l2	F6RBX1	TOM1-like protein 2	134760	1.51E+04	84831	0.00E+00	0.00E+00	0.00E+00
Tomm22	Q9CPQ3	Mitochondrial import receptor subunit TOM22 homolog	687600	8.53E+05	484170	0.00E+00	0.00E+00	0.00E+00
Tpi1	P17751	Triosephosphate isomerase	5387700	3.04E+06	2184800	0.00E+00	0.00E+00	0.00E+00
Tspan2	A0A0G2JDX4	Tetraspanin;Tetraspanin-2	1027000	1.63E+06	648020	0.00E+00	0.00E+00	0.00E+00
Tst	Q545S0	Sulfurtransferase;Thiosulfate sulfurtransferase	319700	3.13E+05	371910	0.00E+00	0.00E+00	0.00E+00
Ttyh1	A0A0U1RPU8	Protein tweety homolog 1	351900	1.85E+05	257460	0.00E+00	0.00E+00	0.00E+00
Uchl1	Q9R0P9	Ubiquitin carboxyl-terminal hydrolase isozyme L1;Ubiquitin carboxyl-terminal hydrolase	2252300	9.50E+05	940850	0.00E+00	0.00E+00	0.00E+00
Uqcrb	Q9CQB4	Cytochrome b-c1 complex subunit 7	259790	3.96E+05	582110	0.00E+00	0.00E+00	0.00E+00
Usp5	Q3U4W8	Ubiquitin carboxyl-terminal hydrolase;Ubiquitin carboxyl-terminal hydrolase 5	519910	3.63E+05	304770	0.00E+00	0.00E+00	0.00E+00
Vapa	Q9WV55	Vesicle-associated membrane protein-associated protein A	936490	5.49E+05	846880	0.00E+00	0.00E+00	0.00E+00
Vat1	Q5RKP0	Synaptic vesicle membrane protein VAT-1 homolog	935710	4.87E+05	269510	0.00E+00	0.00E+00	0.00E+00
Vcp	Q01853	Transitional endoplasmic reticulum ATPase	5733600	4.22E+06	3655000	0.00E+00	0.00E+00	4.50E+03
Vps35	Q3TRJ1	Vacuolar protein sorting-associated protein 35	1020700	7.20E+05	666320	0.00E+00	0.00E+00	0.00E+00

Human vascular proteins

gene	protein	Protein.names	cellular1	cellular2	cellular3	ECM1	ECM2	ECM3
AAK1	Q2M2I8	AP2-associated protein kinase 1	1.12E+05	2.65E+05	1.92E+05	1.04E+05	4.36E+03	1.61E+05
AAR2	A2A2Q9	Protein AAR2 homolog	9.58E+02	0.00E+00	0.00E+00	3.91E+04	0.00E+00	0.00E+00
AARS	P49588	Alanine--tRNA ligase, cytoplasmic	1.23E+06	7.33E+05	1.46E+06	1.31E+06	3.49E+05	9.73E+05
ABCB1	P08183-2	Multidrug resistance protein 1	1.44E+06	1.38E+06	6.25E+05	1.66E+04	0.00E+00	2.75E+04
ABCD3	P28288	ATP-binding cassette sub-family D member 3	2.22E+05	7.60E+04	0.00E+00	0.00E+00	1.14E+05	9.65E+04
ABCE1	P61221	ATP-binding cassette sub-family E member 1	3.12E+04	0.00E+00	3.27E+04	1.51E+04	2.24E+04	2.53E+04
ABCF1	Q8NE71-2	ATP-binding cassette sub-family F member 1	1.87E+05	6.48E+04	5.08E+04	1.82E+05	2.19E+05	2.44E+05
ABCF3	Q9NUQ8-2	ATP-binding cassette sub-family F member 3	0.00E+00	4.02E+04	0.00E+00	4.53E+04	7.56E+04	6.72E+04
ABCG2	Q9UNQ0-2	ATP-binding cassette sub-family G member 2	1.46E+05	2.82E+05	9.63E+04	7.76E+04	0.00E+00	0.00E+00

ABHD10	Q9NUJ1	Mycophenolic acid acyl-glucuronide esterase, mitochondrial	1.35E+06	3.00E+05	7.58E+05	0.00E+00	6.55E+03	0.00E+00
ABHD14B	F8W9U3	Alpha/beta hydrolase domain-containing protein 14B	3.32E+04	0.00E+00	4.16E+04	2.47E+04	0.00E+00	0.00E+00
ABI2	E9PEZ7	Abl interactor 2	2.84E+05	3.55E+04	4.15E+04	0.00E+00	4.94E+04	3.09E+04
ABLIM1	O14639-2	Actin-binding LIM protein 1	1.66E+05	1.56E+05	1.21E+05	4.43E+05	1.07E+05	2.81E+05
ABLIM3	A0A0C4DGA7	Actin-binding LIM protein 3	0.00E+00	2.00E+05	4.68E+05	1.24E+05	8.58E+04	9.39E+04
ABR	Q12979	Active breakpoint cluster region-related protein	2.49E+05	6.05E+04	2.19E+05	5.15E+05	3.85E+05	4.97E+05
ACAA2	A0A0B4J2A4	3-ketoacyl-CoA thiolase, mitochondrial	1.05E+06	5.80E+05	9.60E+05	3.62E+04	0.00E+00	1.39E+04
ACACA	Q13085-3	Acetyl-CoA carboxylase 1;Biotin carboxylase	1.47E+05	2.27E+04	7.96E+04	5.34E+04	1.18E+04	5.51E+04
ACADM	B7Z9I1	Medium-chain specific acyl-CoA dehydrogenase, mitochondrial	4.43E+05	3.54E+05	4.89E+05	4.64E+04	2.61E+04	2.17E+04
ACADSB	P45954	Short/branched chain specific acyl-CoA dehydrogenase, mitochondrial	2.35E+05	1.50E+04	1.27E+05	0.00E+00	1.42E+04	3.16E+04
ACADVL	P49748-2	Very long-chain specific acyl-CoA dehydrogenase, mitochondrial	1.25E+06	1.09E+06	2.83E+05	0.00E+00	5.49E+03	3.33E+04
ACAT1	P24752	Acetyl-CoA acetyltransferase, mitochondrial	6.08E+06	3.84E+06	3.51E+06	8.17E+03	2.35E+03	0.00E+00
ACBD3	Q9H3P7	Golgi resident protein GCP60	1.45E+05	0.00E+00	5.21E+03	2.61E+05	1.30E+05	2.06E+05
ACBD5	B7Z2R7	Acyl-CoA-binding domain-containing protein 5	2.64E+05	9.39E+04	1.97E+03	1.47E+05	1.28E+05	1.72E+05
ACIN1	S4R3H4	Apoptotic chromatin condensation inducer in the nucleus	7.09E+05	1.76E+05	5.37E+05	5.91E+05	9.61E+05	9.91E+05
ACLY	P53396-2	ATP-citrate synthase	8.03E+05	5.00E+05	7.77E+05	2.55E+04	8.21E+04	5.60E+04
ACO2	Q99798	Aconitate hydratase, mitochondrial	2.29E+07	0.00E+00	1.62E+07	3.46E+04	9.40E+04	0.00E+00
ACSL3	O95573	Long-chain-fatty-acid--CoA ligase 3	1.26E+06	5.14E+05	9.72E+05	2.23E+05	9.90E+04	1.40E+05
ACTG1	P63261	Actin, cytoplasmic 2;Actin, cytoplasmic 2, N-terminally processed;Actin, cytoplasmic 1;Actin, cytoplasmic 1, N-terminally processed	0.00E+00	6.35E+07	1.01E+08	3.84E+07	5.03E+07	0.00E+00
ACTL6A	O96019	Actin-like protein 6A	8.36E+04	3.70E+04	0.00E+00	2.43E+05	4.94E+05	5.76E+05
ACTN1	P12814-3	Alpha-actinin-1	8.88E+06	6.78E+06	4.82E+06	1.95E+06	5.25E+05	1.07E+06
ACTN2	P35609	Alpha-actinin-2	2.13E+06	3.22E+06	3.26E+06	5.11E+05	6.88E+05	1.27E+06
ACTN4	O43707	Alpha-actinin-4	3.54E+05	3.95E+05	3.94E+04	5.00E+06	1.39E+06	3.88E+06
ACTR1A	P61163	Alpha-centractin	1.04E+06	6.51E+05	8.20E+05	7.39E+04	0.00E+00	0.00E+00
ACTR2	P61160	Actin-related protein 2	1.52E+06	6.03E+05	1.06E+06	6.76E+04	6.21E+04	0.00E+00
ACTR3	P61158	Actin-related protein 3	1.57E+06	4.97E+05	1.24E+06	0.00E+00	5.35E+04	5.87E+04
ADAM10	O14672	Disintegrin and metalloproteinase domain-containing protein 10	8.69E+05	2.79E+05	1.23E+05	2.48E+03	0.00E+00	0.00E+00
ADAR	P55265-5	Double-stranded RNA-specific adenosine deaminase	1.21E+06	4.46E+05	7.23E+05	1.75E+06	7.66E+05	1.55E+06
ADD1	E7EV99	Alpha-adducin	2.55E+06	1.34E+06	1.80E+06	7.78E+04	1.97E+05	3.36E+05

ADD3	Q9UEY8-2	Gamma-adducin	8.55E+05	8.72E+05	4.18E+05	9.77E+04	5.11E+04	1.26E+04
AFG3L2	Q9Y4W6	AFG3-like protein 2	2.17E+05	0.00E+00	8.13E+04	0.00E+00	4.54E+03	0.00E+00
AGFG1	P52594-2	Arf-GAP domain and FG repeat-containing protein 1	2.76E+04	0.00E+00	6.50E+04	9.87E+04	2.42E+04	1.05E+05
AGK	E9PC15	Acylglycerol kinase, mitochondrial	9.14E+05	4.23E+05	6.97E+05	6.40E+04	6.87E+04	1.01E+05
AGL	P35573-2	Glycogen debranching enzyme;4-alpha-glucanotransferase;Amylo-alpha-1,6-glucosidase	2.08E+05	1.29E+05	1.49E+05	1.25E+05	2.15E+05	1.68E+05
AGPS	O00116	Alkyldihydroxyacetonephosphate synthase, peroxisomal	6.01E+05	2.83E+05	8.28E+04	5.31E+03	0.00E+00	0.00E+00
AGRN	O00468-6	Agrin;Agrin N-terminal 110 kDa subunit;Agrin C-terminal 110 kDa subunit;Agrin C-terminal 90 kDa fragment;Agrin C-terminal 22 kDa fragment	4.97E+06	4.87E+06	1.75E+06	1.44E+07	1.27E+07	9.05E+06
AHCTF1	Q8WYP5	Protein ELYS	7.17E+04	5.26E+04	2.06E+04	2.58E+05	1.54E+05	3.27E+05
AHCYL2	H0Y8B3	Adenosylhomocysteinase;Putative adenosylhomocysteinase 2;Putative adenosylhomocysteinase 3	5.04E+05	2.71E+05	4.72E+05	1.35E+05	0.00E+00	0.00E+00
AHNAK	Q09666	Neuroblast differentiation-associated protein AHNAK	9.96E+06	1.05E+07	6.10E+06	6.98E+06	4.26E+06	7.65E+06
AHNAK2	Q8IVF2	Protein AHNAK2	1.78E+05	6.32E+04	4.03E+04	0.00E+00	2.55E+05	8.22E+03
AHSA1	H0YJG7	Activator of 90 kDa heat shock protein ATPase homolog 1	5.07E+05	3.81E+04	2.14E+05	8.18E+02	4.38E+04	2.86E+05
AIMP1	Q12904-2	Aminoacyl tRNA synthase complex-interacting multifunctional protein 1;Endothelial monocyte-activating polypeptide 2	8.57E+04	8.91E+04	2.21E+05	0.00E+00	4.36E+04	7.18E+03
AIP	O00170	AH receptor-interacting protein	3.68E+05	8.51E+04	3.51E+04	3.43E+05	9.69E+04	3.05E+05
AK1	Q5T9B7	Adenylate kinase isoenzyme 1	3.21E+05	9.51E+04	6.17E+05	2.51E+04	0.00E+00	2.86E+04
AK2	G3V213	Adenylate kinase 2, mitochondrial;Adenylate kinase 2, mitochondrial;Adenylate kinase 2, mitochondrial, N-terminally processed	7.25E+03	3.63E+04	0.00E+00	0.00E+00	1.25E+05	0.00E+00
AK3	Q9UIJ7-2	GTP:AMP phosphotransferase AK3, mitochondrial	1.25E+05	5.91E+04	5.39E+05	0.00E+00	1.26E+04	0.00E+00
AK5	Q9Y6K8-3	Adenylate kinase isoenzyme 5	1.39E+05	1.53E+05	1.48E+05	0.00E+00	1.63E+05	2.25E+05
AKAP12	Q02952-3	A-kinase anchor protein 12	1.25E+06	8.29E+05	6.94E+05	5.96E+04	2.97E+05	3.41E+05
AKAP2	Q9Y2D5-6	A-kinase anchor protein 2	9.80E+03	2.33E+04	5.17E+04	2.86E+03	1.69E+05	2.50E+05
AKAP8L	Q9ULX6	A-kinase anchor protein 8-like	3.23E+05	3.11E+05	0.00E+00	7.40E+05	1.07E+06	1.08E+06
AKR1A1	P14550	Alcohol dehydrogenase [NADP(+)]	3.61E+05	4.16E+05	1.28E+04	2.39E+03	0.00E+00	0.00E+00
AKR7A2	O43488	Aflatoxin B1 aldehyde reductase member 2	5.44E+05	1.19E+05	1.90E+05	1.36E+04	0.00E+00	0.00E+00
AKT1	P31749	RAC-alpha serine/threonine-protein kinase	7.23E+03	0.00E+00	0.00E+00	3.55E+04	5.84E+04	4.09E+04
AKT3	Q9Y243	RAC-gamma serine/threonine-protein kinase	3.53E+04	0.00E+00	0.00E+00	0.00E+00	3.52E+03	2.70E+03
ALDH3A2	J3QRD1	Fatty aldehyde dehydrogenase	7.77E+05	5.37E+05	1.26E+06	4.51E+04	2.85E+04	5.94E+04

ALDH4A1	P30038	Delta-1-pyrroline-5-carboxylate dehydrogenase, mitochondrial	7.78E+05	7.37E+05	2.01E+05	1.04E+06	0.00E+00	0.00E+00
ALDH6A1	Q02252	Methylmalonate-semialdehyde dehydrogenase [acylating], mitochondrial	2.20E+06	1.63E+06	1.46E+06	8.94E+05	4.89E+05	6.76E+05
ALDOA	P04075	Fructose-bisphosphate aldolase A;Fructose-bisphosphate aldolase	0.00E+00	1.14E+07	0.00E+00	2.00E+05	0.00E+00	2.27E+05
ALDOC	P09972	Fructose-bisphosphate aldolase C;Fructose-bisphosphate aldolase	7.47E+06	6.24E+06	1.19E+07	2.09E+05	1.00E+05	4.23E+03
ALG2	Q9H553	Alpha-1,3/1,6-mannosyltransferase ALG2	7.78E+04	2.48E+04	0.00E+00	2.00E+04	0.00E+00	0.00E+00
ALYREF	E9PB61	THO complex subunit 4	1.14E+05	0.00E+00	0.00E+00	2.50E+05	9.91E+04	1.22E+05
ANAPC7	Q9UJX3	Anaphase-promoting complex subunit 7	1.97E+04	0.00E+00	0.00E+00	2.82E+04	0.00E+00	2.12E+04
ANKFY1	Q9P2R3	Rabankyrin-5	1.92E+05	1.42E+05	1.56E+05	2.37E+05	2.00E+04	6.36E+04
ANLN	Q9NQW6-2	Actin-binding protein anillin	3.54E+05	2.36E+05	0.00E+00	5.00E+04	8.08E+04	4.70E+04
ANP32E	Q9BTT0	Acidic leucine-rich nuclear phosphoprotein 32 family member E	5.93E+05	0.00E+00	4.58E+05	7.08E+05	1.31E+06	2.18E+06
ANPEP	P15144	Aminopeptidase N	6.24E+05	5.35E+05	3.03E+05	0.00E+00	9.38E+03	0.00E+00
ANXA1	P04083	Annexin A1;Annexin	7.12E+05	4.10E+05	5.32E+05	1.41E+06	1.65E+05	7.67E+05
ANXA2	P07355-2	Annexin A2;Annexin	3.87E+06	2.01E+06	2.39E+06	0.00E+00	2.45E+04	5.52E+04
ANXA5	P08758	Annexin A5;Annexin	2.15E+06	1.48E+06	9.15E+05	0.00E+00	4.43E+04	4.98E+04
ANXA6	P08133	Annexin A6;Annexin	3.10E+04	4.36E+04	7.59E+04	5.53E+04	3.79E+04	1.48E+04
ANXA7	P20073-2	Annexin A7	3.28E+05	2.60E+05	2.80E+05	0.00E+00	6.11E+03	1.24E+04
AP1B1	Q10567-4	AP-1 complex subunit beta-1	2.43E+05	8.98E+04	1.24E+05	1.47E+04	0.00E+00	7.27E+03
AP2A1	O95782-2	AP-2 complex subunit alpha-1	3.98E+06	2.15E+06	4.42E+06	1.55E+04	7.65E+04	7.31E+04
AP2A2	O94973	AP-2 complex subunit alpha-2	3.00E+06	1.05E+06	2.18E+06	1.53E+05	4.48E+04	5.73E+04
AP2B1	P63010	AP-2 complex subunit beta	3.70E+06	2.78E+06	4.68E+06	2.03E+05	1.72E+05	5.24E+05
AP2M1	A0A087WY71	AP-2 complex subunit mu	3.67E+06	1.14E+06	3.68E+06	3.06E+05	9.19E+04	1.66E+05
AP3B2	Q13367	AP-3 complex subunit beta-2	3.96E+05	1.55E+05	2.94E+05	2.97E+04	2.47E+04	2.08E+04
AP3D1	O14617-5	AP-3 complex subunit delta-1	2.75E+05	3.27E+04	2.67E+05	2.64E+04	1.04E+04	1.28E+04
AP3S1	F5H459	AP-3 complex subunit sigma-1	0.00E+00	5.45E+04	2.81E+05	5.87E+04	0.00E+00	0.00E+00
API5	G3V1C3	Apoptosis inhibitor 5	6.53E+04	4.84E+04	2.23E+04	1.99E+04	0.00E+00	4.58E+04
APP	P05067-7	Amyloid beta A4 protein;N-APP;Soluble APP-alpha;Soluble APP-beta;C99;Beta-amyloid protein 42;Beta-amyloid protein 40;C83;P3(42);P3(40);C80;Gamma-secretase C-terminal fragment 59;Gamma-secretase C-terminal fragment 57;Gamma-secretase C-terminal fragment 50;C31	4.28E+05	2.33E+05	4.25E+04	0.00E+00	1.70E+04	7.17E+03

APPL1	Q9UKG1	DCC-interacting protein 13-alpha	1.51E+05	4.12E+04	1.97E+05	1.70E+04	2.34E+04	1.08E+05
AQR	O60306	Intron-binding protein aquarius	1.34E+05	0.00E+00	2.87E+04	2.99E+04	2.16E+04	2.69E+04
ARCN1	B0YIW6	Coatomer subunit delta	2.32E+05	8.48E+04	7.39E+04	4.33E+04	0.00E+00	1.16E+04
ARF1	P84077	ADP-ribosylation factor 1;ADP-ribosylation factor 3	4.04E+06	3.19E+06	1.80E+06	6.07E+05	6.09E+05	3.30E+05
ARF4	P18085	ADP-ribosylation factor 4	5.57E+05	4.57E+05	1.85E+05	9.68E+04	2.63E+05	1.14E+05
ARF6	P62330	ADP-ribosylation factor 6	5.02E+05	1.11E+05	2.11E+05	3.14E+05	4.24E+05	3.59E+05
ARFGAP1	Q8N6T3	ADP-ribosylation factor GTPase-activating protein 1	1.60E+05	9.66E+04	3.79E+04	6.17E+04	4.50E+04	1.48E+05
ARFGAP2	A0A0D9SF70	ADP-ribosylation factor GTPase-activating protein 2	5.30E+04	0.00E+00	7.41E+04	1.55E+05	1.93E+05	1.14E+05
ARFGEF2	Q9Y6D5	Brefeldin A-inhibited guanine nucleotide-exchange protein 2	2.05E+05	6.89E+04	3.81E+04	1.33E+05	3.22E+04	1.46E+05
ARFIP1	P53367	Arfaptin-1	2.78E+04	0.00E+00	0.00E+00	0.00E+00	4.44E+04	6.12E+03
ARHGAP1	Q07960	Rho GTPase-activating protein 1	1.61E+06	7.35E+05	9.39E+05	6.09E+04	1.97E+05	4.81E+05
ARHGAP21	E7ESW5	Rho GTPase-activating protein 21	8.06E+02	0.00E+00	3.03E+04	4.63E+03	0.00E+00	4.07E+04
ARHGAP35	Q9NRY4	Rho GTPase-activating protein 35	7.86E+04	0.00E+00	2.44E+05	4.14E+04	0.00E+00	4.89E+04
ARHGDIA	J3KTF8	Rho GDP-dissociation inhibitor 1	2.56E+06	1.73E+06	2.27E+06	5.64E+05	1.48E+05	2.93E+05
ARHGEF1	M0QZR4	Rho guanine nucleotide exchange factor 1	3.62E+04	0.00E+00	0.00E+00	0.00E+00	6.68E+03	6.64E+03
ARHGEF10	H0YAN8	Rho guanine nucleotide exchange factor 10	1.76E+04	0.00E+00	0.00E+00	3.60E+04	1.96E+04	8.21E+04
ARHGEF12	E9PMR6	Rho guanine nucleotide exchange factor 12	3.06E+04	2.98E+04	0.00E+00	1.18E+05	1.62E+05	1.18E+05
ARHGEF17	Q96PE2	Rho guanine nucleotide exchange factor 17	5.15E+03	5.44E+04	6.90E+03	2.28E+05	2.31E+05	2.79E+05
ARHGEF2	V9GYM8	Rho guanine nucleotide exchange factor 2	2.12E+05	2.50E+04	1.52E+05	2.54E+05	1.26E+05	8.99E+04
ARHGEF7	Q14155-3	Rho guanine nucleotide exchange factor 7	2.89E+05	1.30E+05	2.04E+05	1.39E+05	1.72E+05	1.66E+05
ARL2	P36404	ADP-ribosylation factor-like protein 2	1.01E+05	3.20E+04	1.43E+05	9.00E+04	1.21E+05	1.22E+05
ARL3	P36405	ADP-ribosylation factor-like protein 3	6.13E+04	3.10E+04	1.20E+05	2.55E+04	0.00E+00	0.00E+00
ARL6IP4	F5GYV5	ADP-ribosylation factor-like protein 6-interacting protein 4	4.46E+04	1.84E+04	3.45E+04	9.42E+04	6.42E+04	1.61E+05
ARL8B	Q9NVJ2	ADP-ribosylation factor-like protein 8B	1.25E+06	2.70E+05	4.50E+05	2.19E+04	3.57E+03	0.00E+00
ARMC6	Q6NXE6-2	Armadillo repeat-containing protein 6	4.19E+03	7.27E+03	2.38E+03	7.88E+03	5.16E+04	0.00E+00
ARPC1A	A0A1W2PNV4	Actin-related protein 2/3 complex subunit 1B	1.63E+05	1.05E+05	3.74E+05	2.59E+04	2.03E+04	2.79E+04
ARPC2	O15144	Actin-related protein 2/3 complex subunit 2	4.97E+05	2.06E+05	5.15E+05	1.61E+04	0.00E+00	0.00E+00
ASAH1	Q13510-2	Acid ceramidase;Acid ceramidase subunit alpha;Acid ceramidase subunit beta	2.61E+06	1.23E+06	1.99E+06	1.76E+05	0.00E+00	0.00E+00
ASH2L	F5H8F7	Set1/Ash2 histone methyltransferase complex subunit ASH2	7.87E+04	0.00E+00	0.00E+00	2.42E+05	9.41E+04	1.27E+05
ASMTL	O95671-3	N-acetylserotonin O-methyltransferase-like protein	0.00E+00	2.47E+04	0.00E+00	1.67E+04	0.00E+00	4.83E+03
ASPH	Q12797-10	Aspartyl/asparaginyl beta-hydroxylase	1.11E+06	5.76E+05	1.18E+06	3.06E+04	2.63E+04	4.06E+04
ATAD3A	Q9NVI7-2	ATPase family AAA domain-containing protein	2.06E+05	9.84E+04	1.37E+05	8.86E+04	9.31E+04	1.04E+05

		3A;ATPase family AAA domain-containing protein 3B						
ATG3	Q9NT62	Ubiquitin-like-conjugating enzyme ATG3	9.62E+04	1.27E+05	2.03E+05	1.71E+05	1.18E+05	3.98E+05
ATIC	P31939	Bifunctional purine biosynthesis protein PURH;Phosphoribosylaminoimidazolecarboxamide formyltransferase;IMP cyclohydrolase	1.29E+05	7.19E+04	3.30E+04	3.97E+04	0.00E+00	0.00E+00
ATL3	Q6DD88	Atlastin-3	8.04E+05	3.23E+05	3.98E+05	4.97E+04	0.00E+00	0.00E+00
ATP1A1	P05023-4	Sodium/potassium-transporting ATPase subunit alpha-1	5.32E+07	5.21E+07	4.46E+07	6.63E+05	2.25E+06	3.92E+05
ATP1A2	B1AKY9	Sodium/potassium-transporting ATPase subunit alpha-2	7.53E+06	1.02E+07	6.45E+06	6.53E+04	1.25E+05	6.81E+03
ATP1B1	P05026	Sodium/potassium-transporting ATPase subunit beta-1	1.22E+07	1.16E+07	8.99E+06	1.48E+05	1.38E+05	1.19E+04
ATP2A2	P16615-5	Sarcoplasmic/endoplasmic reticulum calcium ATPase 2	8.08E+06	7.57E+06	6.82E+06	4.85E+04	3.72E+05	6.92E+04
ATP2B1	P20020-5	Plasma membrane calcium-transporting ATPase 1;Calcium-transporting ATPase	9.10E+06	7.99E+06	1.14E+07	3.46E+05	5.24E+05	3.74E+05
ATP2B4	P23634-7	Plasma membrane calcium-transporting ATPase 4	7.19E+05	6.77E+05	2.96E+05	2.68E+04	0.00E+00	0.00E+00
ATP5A1	P25705	ATP synthase subunit alpha, mitochondrial	5.25E+07	2.65E+07	4.68E+07	1.92E+06	3.45E+06	4.14E+06
ATP5B	P06576	ATP synthase subunit beta, mitochondrial;ATP synthase subunit beta	8.41E+07	4.21E+07	7.93E+07	9.14E+05	1.28E+06	1.24E+06
ATP5C1	P36542	ATP synthase subunit gamma, mitochondrial	3.04E+06	1.62E+06	2.42E+06	2.22E+05	1.17E+05	2.60E+04
ATP5O	P48047	ATP synthase subunit O, mitochondrial	4.19E+06	3.97E+06	8.10E+06	0.00E+00	1.30E+03	0.00E+00
ATP6V0A1	Q93050-1	V-type proton ATPase 116 kDa subunit a isoform 1;V-type proton ATPase subunit a	3.08E+06	4.07E+06	2.51E+06	2.07E+04	2.55E+05	4.51E+05
ATP6V1A	P38606	V-type proton ATPase catalytic subunit A	7.43E+06	3.85E+06	7.15E+06	2.94E+05	5.44E+05	8.52E+05
ATP6V1B2	P21281	V-type proton ATPase subunit B, brain isoform	2.76E+06	1.74E+06	2.32E+06	0.00E+00	1.16E+05	6.95E+04
ATP6V1C1	P21283	V-type proton ATPase subunit C 1	9.68E+05	6.29E+05	8.45E+05	0.00E+00	1.20E+04	7.26E+03
ATP6V1E1	P36543	V-type proton ATPase subunit E 1	1.84E+06	1.07E+06	1.37E+06	6.15E+04	0.00E+00	0.00E+00
ATRX	P46100-5	Transcriptional regulator ATRX	4.62E+04	2.48E+04	1.14E+05	6.54E+05	9.90E+05	1.05E+06
ATXN10	Q9UBB4	Ataxin-10	9.96E+04	9.93E+04	4.55E+05	2.51E+04	4.06E+04	2.80E+04
BABAM1	M0R0I0	BRISC and BRCA1-A complex member 1	1.29E+04	0.00E+00	0.00E+00	0.00E+00	8.55E+03	0.00E+00
BAG5	Q9UL15-2	BAG family molecular chaperone regulator 5	4.03E+04	2.14E+04	2.50E+04	2.58E+04	8.97E+03	3.50E+04
BAIAP2	Q9UQB8	Brain-specific angiogenesis inhibitor 1-associated protein 2	2.83E+05	1.16E+05	8.25E+04	8.67E+04	1.62E+05	1.42E+05
BASP1	P80723	Brain acid soluble protein 1	2.13E+06	1.48E+06	1.02E+06	1.03E+05	8.60E+05	6.30E+05
BAT3	A0A024RCR6	Large proline-rich protein BAG6	3.52E+04	2.07E+04	7.89E+04	7.22E+04	7.03E+04	1.28E+05
BAZ1B	Q9UIG0-2	Tyrosine-protein kinase BAZ1B	8.70E+04	0.00E+00	6.63E+03	1.31E+06	1.37E+06	1.83E+06
BCAM	A0A087WXM8	Basal cell adhesion molecule	6.63E+05	8.36E+05	8.98E+04	2.51E+06	9.34E+05	6.67E+05
BCAS2	O75934	Pre-mRNA-splicing factor SPF27	7.52E+04	3.08E+04	2.47E+04	1.31E+04	1.60E+04	3.33E+04

BCKDHA	F5H5P2	2-oxoisovalerate dehydrogenase subunit alpha, mitochondrial	6.88E+05	2.38E+05	5.68E+04	2.40E+04	2.91E+04	3.84E+04
BCLAF1	Q9NYF8-2	Bcl-2-associated transcription factor 1	6.86E+05	6.84E+04	3.23E+04	1.27E+05	2.00E+04	1.00E+05
BCR	P11274	Breakpoint cluster region protein	4.52E+04	2.13E+04	0.00E+00	2.19E+05	6.29E+04	1.78E+05
BGN	P21810	Biglycan	6.29E+05	3.52E+04	1.37E+04	9.19E+05	1.98E+05	5.49E+04
BIN1	O00499-9	Myc box-dependent-interacting protein 1	2.69E+06	2.08E+06	1.15E+06	8.43E+03	2.55E+04	2.00E+04
BLVRB	P30043	Flavin reductase (NADPH)	8.08E+04	0.00E+00	0.00E+00	1.66E+05	0.00E+00	0.00E+00
BMS1	Q14692	Ribosome biogenesis protein BMS1 homolog	0.00E+00	5.42E+05	0.00E+00	1.15E+05	1.97E+05	7.97E+04
BOP1	Q14137	Ribosome biogenesis protein BOP1	1.22E+04	0.00E+00	0.00E+00	1.94E+05	5.24E+05	2.55E+05
BPTF	F5GXF5	Nucleosome-remodeling factor subunit BPTF	7.66E+03	0.00E+00	0.00E+00	1.41E+05	1.14E+05	1.93E+05
BRD3	Q15059	Bromodomain-containing protein 3	2.99E+04	0.00E+00	0.00E+00	1.58E+05	0.00E+00	6.94E+03
BROX	Q5VW32-2	BRO1 domain-containing protein BROX	2.82E+04	4.25E+04	0.00E+00	3.16E+03	0.00E+00	0.00E+00
BSG	P35613-2	Basigin	9.62E+05	4.92E+05	2.21E+05	8.04E+04	0.00E+00	0.00E+00
BST2	Q10589-2	Bone marrow stromal antigen 2	3.39E+04	3.14E+04	1.36E+04	1.09E+04	0.00E+00	2.08E+04
C14orf159	Q7Z3D6-2	UPF0317 protein C14orf159, mitochondrial	6.34E+05	5.23E+04	0.00E+00	5.35E+04	0.00E+00	0.00E+00
C17orf85	Q53F19	Uncharacterized protein C17orf85	0.00E+00	1.40E+04	0.00E+00	9.49E+04	1.15E+05	1.25E+05
C1orf198	Q9H425	Uncharacterized protein C1orf198	3.27E+04	3.32E+03	0.00E+00	1.91E+05	1.26E+05	2.33E+05
C1QBP	Q07021	Complement component 1 Q subcomponent-binding protein, mitochondrial	2.84E+06	2.43E+06	1.48E+06	2.49E+05	1.48E+05	3.66E+05
C21orf33	A0A096LPI6	ES1 protein homolog, mitochondrial	1.22E+06	5.89E+05	3.07E+05	6.77E+04	5.73E+03	8.11E+04
C2CD2	Q9Y426	C2 domain-containing protein 2	8.80E+04	4.27E+04	3.78E+04	0.00E+00	1.73E+04	0.00E+00
C4orf27	Q9NWY4	UPF0609 protein C4orf27	3.33E+04	0.00E+00	0.00E+00	2.41E+05	1.37E+04	3.48E+04
C7orf50	C9JQV0	Uncharacterized protein C7orf50	3.66E+03	0.00E+00	0.00E+00	5.90E+04	3.66E+03	0.00E+00
CAD	P27708	CAD protein;Glutamine-dependent carbamoyl-phosphate synthase;Aspartate carbamoyltransferase;Dihydroorotase	2.74E+04	0.00E+00	0.00E+00	0.00E+00	7.13E+03	0.00E+00
CALD1	Q05682	Caldesmon	1.54E+06	3.97E+05	8.07E+05	8.18E+05	1.66E+05	4.25E+05
CALR	P27797	Calreticulin	2.36E+06	2.56E+06	9.61E+05	5.94E+04	0.00E+00	2.63E+04
CALU	O43852	Calumenin	3.13E+05	4.98E+05	3.72E+05	2.85E+04	1.41E+04	5.96E+04
CAMK1	Q14012	Calcium/calmodulin-dependent protein kinase type 1	2.38E+05	1.46E+05	1.97E+05	1.05E+05	1.08E+05	1.16E+05
CAMK2B	Q13554-2	Calcium/calmodulin-dependent protein kinase type II subunit beta	7.68E+06	4.73E+06	7.67E+06	1.76E+07	2.09E+07	3.34E+07
CAMK2G	Q13555-6	Calcium/calmodulin-dependent protein kinase type II subunit gamma	9.36E+04	1.94E+05	1.67E+05	5.81E+05	1.90E+06	1.89E+06
CAND1	Q86VP6	Cullin-associated NEDD8-dissociated protein 1	1.03E+06	8.71E+05	1.18E+06	0.00E+00	1.05E+05	1.91E+04
CAND2	O75155-2	Cullin-associated NEDD8-dissociated protein 2	5.34E+04	2.72E+04	0.00E+00	8.11E+04	6.90E+04	7.59E+04

CANX	P27824	Calnexin	1.13E+07	4.28E+06	3.90E+06	3.48E+05	3.51E+05	5.24E+05
CAP1	Q01518-2	Adenylyl cyclase-associated protein 1	1.56E+06	1.10E+06	1.08E+06	3.99E+05	1.64E+05	3.12E+05
CAP2	P40123	Adenylyl cyclase-associated protein 2;Adenylyl cyclase-associated protein	1.15E+06	4.93E+05	1.14E+05	3.93E+05	1.93E+05	5.19E+05
CAPN1	P07384	Calpain-1 catalytic subunit	2.57E+05	1.45E+05	4.12E+04	1.18E+05	0.00E+00	5.12E+04
CAPRIN1	G3V153	Caprin-1	2.40E+05	8.32E+03	0.00E+00	1.28E+05	4.10E+04	1.30E+05
CAPZA1	P52907	F-actin-capping protein subunit alpha-1	4.93E+05	5.06E+05	6.59E+05	1.29E+05	1.81E+05	2.52E+05
CAPZA2	P47755	F-actin-capping protein subunit alpha-2	1.05E+06	6.90E+05	3.67E+05	7.72E+04	2.86E+04	5.01E+04
CASKIN2	Q8WXE0	Caskin-2	2.47E+04	2.65E+04	4.19E+04	1.06E+05	6.73E+04	7.56E+04
CAT	P04040	Catalase	5.29E+05	4.71E+05	2.14E+05	0.00E+00	1.47E+04	6.26E+03
CAV1	Q03135	Caveolin-1;Caveolin	9.69E+05	1.06E+06	2.32E+05	4.99E+05	6.52E+05	7.26E+05
CBR1	P16152	Carbonyl reductase [NADPH] 1	6.07E+06	3.46E+06	2.79E+06	2.10E+06	5.36E+05	4.96E+05
CBX3	Q13185	Chromobox protein homolog 3	2.07E+05	2.99E+05	3.55E+04	9.88E+05	1.41E+06	2.22E+06
CBX5	P45973	Chromobox protein homolog 5	8.46E+03	2.85E+04	0.00E+00	2.65E+05	3.91E+05	2.93E+05
CCAR1	F5H2E6	Cell division cycle and apoptosis regulator protein 1	8.86E+04	1.36E+04	1.14E+05	1.85E+04	0.00E+00	5.39E+04
CCAR2	Q8N163-2	Cell cycle and apoptosis regulator protein 2	1.12E+06	6.89E+05	8.60E+05	1.95E+06	2.24E+06	0.00E+00
CCBL2	Q6YP21-3	Kynurenine--oxoglutarate transaminase 3	8.26E+04	2.53E+05	6.07E+05	0.00E+00	3.48E+04	2.29E+04
CCDC132	Q96JG6-3	Coiled-coil domain-containing protein 132	6.67E+04	2.82E+04	8.68E+04	1.27E+04	0.00E+00	1.82E+04
CCDC6	Q16204	Coiled-coil domain-containing protein 6	4.27E+05	1.76E+05	1.44E+05	1.14E+04	3.87E+04	3.00E+04
CCDC88A	Q3V6T2-5	Girdin	4.37E+05	2.22E+04	0.00E+00	1.18E+05	4.52E+04	8.19E+04
CCNY	Q8ND76-3	Cyclin-Y	8.72E+04	2.73E+04	5.83E+04	4.98E+04	0.00E+00	0.00E+00
CCT2	P78371	T-complex protein 1 subunit beta	1.73E+06	8.51E+05	1.79E+06	4.47E+04	4.47E+04	8.39E+04
CCT3	B4DUR8	T-complex protein 1 subunit gamma	2.46E+06	1.12E+06	1.38E+06	1.30E+05	1.04E+05	0.00E+00
CCT4	P50991	T-complex protein 1 subunit delta	1.26E+06	1.08E+06	9.45E+05	9.56E+04	1.41E+05	6.01E+04
CCT6A	P40227	T-complex protein 1 subunit zeta	1.16E+06	4.04E+05	5.90E+05	2.53E+04	0.00E+00	0.00E+00
CCT7	Q99832-3	T-complex protein 1 subunit eta	2.11E+06	8.21E+05	1.17E+06	4.98E+04	5.09E+04	6.43E+04
CCT8	P50990-2	T-complex protein 1 subunit theta	1.04E+06	5.20E+05	1.19E+06	1.97E+04	1.02E+05	3.40E+04
CD2BP2	O95400	CD2 antigen cytoplasmic tail-binding protein 2	1.93E+03	4.46E+04	0.00E+00	3.11E+04	4.24E+04	1.46E+05
CD59	E9PNW4	CD59 glycoprotein	2.27E+06	6.16E+05	2.82E+05	0.00E+00	6.87E+04	5.68E+04
CD81	E9PJK1	Tetraspanin;CD81 antigen	4.36E+06	3.26E+06	2.18E+06	1.06E+04	8.44E+04	2.48E+04
CD9	A6NNI4	Tetraspanin;CD9 antigen	4.62E+06	2.10E+06	6.55E+05	0.00E+00	4.26E+04	0.00E+00
CDC16	Q13042-3	Cell division cycle protein 16 homolog	0.00E+00	2.69E+04	5.44E+04	4.44E+04	0.00E+00	0.00E+00
CDC27	G5EA36	Cell division cycle protein 27 homolog	1.08E+04	1.23E+04	5.78E+03	1.00E+04	0.00E+00	2.11E+04
CDC40	Q5SRN1	Pre-mRNA-processing factor 17	0.00E+00	3.01E+05	0.00E+00	1.72E+05	2.75E+04	8.03E+05
CDC42BPA	A0A0A0MRJ0	Non-specific serine/threonine protein	6.14E+04	1.70E+04	1.68E+04	1.25E+05	3.62E+04	1.10E+05

		kinase;Serine/threonine-protein kinase MRCK alpha						
CDC42BPB	Q9Y5S2	Serine/threonine-protein kinase MRCK beta	7.52E+04	3.70E+04	9.71E+04	1.91E+05	5.93E+04	3.36E+05
CDC42EP4	Q9H3Q1-2	Cdc42 effector protein 4	1.26E+04	4.54E+04	0.00E+00	1.14E+05	2.12E+05	1.97E+05
CDC5L	Q99459	Cell division cycle 5-like protein	2.07E+05	1.40E+05	0.00E+00	2.85E+05	6.59E+04	1.60E+05
CDH2	P19022-2	Cadherin-2	3.62E+05	1.12E+05	2.96E+05	3.50E+04	2.50E+04	2.86E+04
CDK11B	J3QR44	Cyclin-dependent kinase 11B;Cyclin-dependent kinase 11A	5.17E+04	0.00E+00	3.17E+04	1.70E+05	0.00E+00	6.07E+04
CDK18	Q07002	Cyclin-dependent kinase 18	1.48E+05	2.37E+03	1.40E+05	3.42E+05	5.88E+04	3.17E+04
CDK5	Q00535	Cyclin-dependent-like kinase 5	5.50E+05	8.73E+04	1.09E+05	3.23E+05	2.67E+05	2.93E+05
CDK5RAP3	Q96JB5	CDK5 regulatory subunit-associated protein 3	3.18E+05	2.16E+05	1.10E+05	7.55E+04	1.75E+05	2.63E+04
CDK9	P50750-2	Cyclin-dependent kinase 9	1.38E+04	1.61E+04	0.00E+00	1.72E+04	3.70E+04	0.00E+00
CDKN2AIP	Q9NXV6	CDKN2A-interacting protein	1.59E+04	0.00E+00	0.00E+00	6.82E+04	3.70E+04	5.93E+04
CEBPZ	Q03701	CCAAT/enhancer-binding protein zeta	0.00E+00	4.55E+04	0.00E+00	0.00E+00	1.69E+04	1.78E+04
CELF1	G5EA30	CUGBP Elav-like family member 1	7.78E+04	1.79E+05	1.78E+05	0.00E+00	8.05E+04	9.00E+04
CELF2	V9GYD9	CUGBP Elav-like family member 2	5.67E+05	4.77E+05	3.97E+05	4.80E+04	4.03E+05	3.33E+05
CEP170	Q5SW79-2	Centrosomal protein of 170 kDa	3.39E+04	8.29E+02	0.00E+00	8.46E+04	4.40E+04	5.64E+04
CETN2	P41208	Centrin-2	7.59E+04	9.07E+03	6.18E+04	2.38E+04	0.00E+00	1.39E+04
CFL1	G3V1A4	Cofilin-1	5.57E+06	4.33E+06	7.54E+06	6.85E+05	7.08E+04	3.37E+05
CHAMP1	Q96JM3	Chromosome alignment-maintaining phosphoprotein 1	5.66E+04	3.20E+04	0.00E+00	2.52E+05	4.31E+05	4.12E+05
CHCHD2	Q9Y6H1	Coiled-coil-helix-coiled-coil-helix domain-containing protein 2;Putative coiled-coil-helix-coiled-coil-helix domain-containing protein CHCHD2P9, mitochondrial	4.30E+03	0.00E+00	0.00E+00	1.44E+04	0.00E+00	8.24E+03
CHCHD3	C9JRZ6	MICOS complex subunit MIC19	7.21E+05	9.45E+05	3.50E+05	3.15E+05	3.04E+05	2.88E+05
CHCHD6	J3QTA6	MICOS complex subunit MIC25	1.82E+04	0.00E+00	3.16E+04	1.73E+04	0.00E+00	2.83E+04
CHD4	F5GWX5	Chromodomain-helicase-DNA-binding protein 4	5.60E+05	6.93E+04	6.66E+04	1.67E+06	1.14E+06	1.32E+06
CHERP	J3QK89	Calcium homeostasis endoplasmic reticulum protein	4.23E+05	3.10E+04	9.93E+04	8.88E+04	1.22E+05	1.38E+05
CHMP4B	Q9H444	Charged multivesicular body protein 4b	2.75E+05	0.00E+00	6.42E+03	2.33E+04	6.43E+04	3.98E+05
CHMP6	I3L4G8	Charged multivesicular body protein 6	3.30E+05	2.39E+05	8.35E+04	7.18E+04	7.88E+04	9.90E+04
CIAO1	O76071	Probable cytosolic iron-sulfur protein assembly protein CIAO1	9.81E+02	0.00E+00	0.00E+00	6.90E+04	0.00E+00	0.00E+00
CIRBP	Q14011	Cold-inducible RNA-binding protein	0.00E+00	3.47E+04	6.33E+03	8.31E+04	2.39E+04	5.83E+04
CKAP4	Q07065	Cytoskeleton-associated protein 4	1.07E+06	6.80E+05	6.51E+05	3.11E+04	8.98E+03	0.00E+00
CKAP5	Q14008-2	Cytoskeleton-associated protein 5	3.47E+05	2.13E+05	3.10E+05	7.82E+04	1.34E+05	1.33E+05
CKB	P12277	Creatine kinase B-type	7.11E+07	2.43E+07	4.25E+07	4.54E+07	1.28E+07	2.64E+07
CLASP1	H0Y5T1	CLIP-associating protein 1	0.00E+00	1.88E+04	0.00E+00	3.70E+04	0.00E+00	2.86E+04
CLASP2	E3W994	CLIP-associating protein 2	3.88E+05	6.48E+04	3.21E+05	2.72E+05	1.47E+05	1.86E+05

CLIC4	Q9Y696	Chloride intracellular channel protein 4	1.06E+06	5.45E+05	7.16E+04	1.89E+05	0.00E+00	5.34E+04
CLIP2	Q9UDT6	CAP-Gly domain-containing linker protein 2	8.30E+04	2.00E+05	5.86E+04	2.04E+07	1.55E+04	0.00E+00
CLNS1A	E9PMI6	Methylosome subunit pICln	1.16E+04	0.00E+00	0.00E+00	2.12E+05	7.14E+04	3.78E+04
CLPB	H0YGM0	Caseinolytic peptidase B protein homolog	9.77E+04	4.04E+04	8.05E+04	7.48E+03	2.24E+04	0.00E+00
CLTA	P09496-5	Clathrin light chain A	2.69E+05	1.05E+05	1.46E+05	4.12E+03	0.00E+00	3.32E+04
CLTC	A0A087WVQ6	Clathrin heavy chain;Clathrin heavy chain 1	3.20E+07	1.78E+07	2.86E+07	1.39E+06	9.94E+05	1.16E+06
CLTCL1	P53675	Clathrin heavy chain 2	0.00E+00	1.62E+04	0.00E+00	5.20E+04	0.00E+00	0.00E+00
CLU	P10909-4	Clusterin;Clusterin beta chain;Clusterin alpha chain;Clusterin	1.49E+06	9.65E+05	8.41E+05	1.08E+06	6.77E+05	9.02E+05
CMAS	Q8NFW8	N-acylneuraminate cytidylyltransferase	4.46E+05	1.16E+04	1.06E+05	2.17E+05	3.64E+05	6.13E+05
CMPK1	P30085	UMP-CMP kinase	1.33E+05	4.95E+03	3.93E+04	1.49E+05	8.49E+03	1.81E+05
CMPK2	Q5EBM0-3	UMP-CMP kinase 2, mitochondrial	1.64E+04	0.00E+00	0.00E+00	1.96E+04	2.50E+04	1.08E+05
CNDP2	Q96KP4	Cytosolic non-specific dipeptidase	1.96E+06	7.42E+05	8.35E+05	0.00E+00	2.26E+04	2.56E+04
CNN3	Q15417-3	Calponin-3	7.95E+04	2.25E+04	1.82E+04	4.26E+04	0.00E+00	5.44E+04
CNP	P09543-2	2,3-cyclic-nucleotide 3-phosphodiesterase	1.09E+08	6.42E+07	5.18E+07	3.51E+07	4.02E+07	3.90E+07
CNPY2	F8W1K5	Protein canopy homolog 2	7.55E+05	4.85E+05	3.44E+05	7.17E+04	5.08E+04	1.61E+05
CNPY3	Q9BT09	Protein canopy homolog 3	1.70E+05	1.27E+05	2.18E+04	0.00E+00	4.20E+04	3.72E+04
CNRIP1	B8ZZB8	CB1 cannabinoid receptor-interacting protein 1	3.35E+05	3.44E+05	2.10E+05	3.43E+04	5.81E+04	9.35E+04
CNTNAP1	P78357	Contactin-associated protein 1	6.26E+06	0.00E+00	4.05E+06	7.92E+05	2.03E+05	4.31E+05
COASY	Q13057	Bifunctional coenzyme A synthase;Phosphopantetheine adenylyltransferase;Dephospho-CoA kinase	4.90E+04	2.58E+04	5.94E+04	0.00E+00	5.89E+04	1.93E+04
COBLL1	A0A0X1KG75	Cordon-bleu protein-like 1	9.04E+04	0.00E+00	9.82E+04	9.26E+04	3.82E+04	1.55E+05
COCH	H0YJW4	Cochlin	1.66E+05	7.42E+04	1.80E+04	3.27E+04	0.00E+00	0.00E+00
COL12A1	D6RGG3	Collagen alpha-1(XII) chain	3.39E+05	8.63E+04	2.53E+04	1.18E+06	8.93E+05	6.92E+05
COL18A1	P39060-2	Collagen alpha-1(XVIII) chain;Endostatin	3.26E+04	2.73E+04	1.72E+04	2.20E+06	1.86E+06	2.10E+06
COL4A2	P08572	Collagen alpha-2(IV) chain;Canstatin	2.52E+04	1.99E+04	3.54E+04	2.28E+07	2.53E+07	1.27E+07
COL6A1	A0A087X0S5	Collagen alpha-1(VI) chain	2.30E+06	2.91E+05	1.76E+05	4.37E+06	3.13E+06	1.42E+06
COL6A3	E7ENL6	Collagen alpha-3(VI) chain	2.25E+06	2.15E+05	0.00E+00	1.19E+07	1.15E+07	5.11E+06
COPA	P53621	Coatomer subunit alpha;Xenin;Proxenin	1.33E+06	3.56E+05	3.89E+05	5.61E+05	4.32E+05	9.35E+05
COPB1	P53618	Coatomer subunit beta	3.41E+05	5.91E+04	1.18E+05	1.72E+05	0.00E+00	1.36E+05
COPB2	P35606-2	Coatomer subunit beta	3.62E+05	1.54E+05	3.77E+05	9.56E+04	2.81E+04	8.96E+04
COPG1	Q9Y678	Coatomer subunit gamma-1	2.76E+05	3.77E+04	2.05E+05	3.80E+04	1.59E+04	3.34E+04
COPS2	B4DIH5	COP9 signalosome complex subunit 2	1.01E+05	7.71E+04	0.00E+00	3.41E+04	0.00E+00	1.42E+04
COPS3	H7C3P9	COP9 signalosome complex subunit 3	8.38E+04	6.53E+04	3.59E+02	0.00E+00	9.03E+03	0.00E+00

COPS7A	F5H7C6	COP9 signalosome complex subunit 7a	1.22E+05	3.67E+04	8.82E+04	6.65E+03	0.00E+00	1.51E+04
CORO1A	P31146	Coronin-1A;Coronin	1.23E+05	9.79E+04	1.33E+04	1.02E+04	0.00E+00	0.00E+00
CORO1B	Q9BR76	Coronin-1B	1.44E+04	2.44E+04	0.00E+00	1.43E+05	0.00E+00	1.97E+04
CORO7-PAM16	A0A0A6YYL4	Coronin;Coronin-7	0.00E+00	4.05E+04	3.51E+04	1.19E+05	2.10E+05	2.48E+05
COX20	Q5RI15	Cytochrome c oxidase protein 20 homolog	2.71E+05	9.54E+04	8.43E+04	4.95E+03	0.00E+00	0.00E+00
COX4I1	P13073	Cytochrome c oxidase subunit 4 isoform 1, mitochondrial	1.81E+06	5.94E+05	7.61E+05	1.60E+04	0.00E+00	0.00E+00
COX5B	P10606	Cytochrome c oxidase subunit 5B, mitochondrial	2.66E+06	3.61E+05	2.35E+06	1.00E+04	0.00E+00	0.00E+00
COX6B1	P14854	Cytochrome c oxidase subunit 6B1	2.58E+06	1.11E+06	1.76E+06	2.89E+04	3.68E+04	3.59E+04
CPNE3	O75131	Copine-3	1.64E+04	2.31E+04	4.50E+04	3.30E+04	0.00E+00	0.00E+00
CPNE6	O95741	Copine-6	6.81E+05	2.00E+05	1.35E+05	2.79E+05	2.94E+05	4.68E+04
CPSF1	Q10570	Cleavage and polyadenylation specificity factor subunit 1	4.52E+04	8.28E+04	3.19E+03	7.44E+04	1.06E+05	1.13E+05
CPSF6	F8WJN3	Cleavage and polyadenylation specificity factor subunit 6	4.17E+04	5.55E+04	6.75E+04	0.00E+00	4.86E+04	3.34E+04
CPSF7	F5H6M0	Cleavage and polyadenylation specificity factor subunit 7	7.47E+04	8.24E+04	0.00E+00	7.22E+04	3.37E+04	6.76E+04
CREB1	C9JBT4	Cyclic AMP-responsive element-binding protein 1	0.00E+00	2.83E+04	0.00E+00	4.27E+04	1.05E+05	1.36E+05
CRIP2	H0YFA4	Cysteine-rich protein 2	3.38E+05	0.00E+00	8.81E+04	1.05E+06	1.02E+05	1.05E+06
CRKL	P46109	Crk-like protein	0.00E+00	1.12E+05	3.08E+04	4.04E+04	6.17E+04	0.00E+00
CRMP1	E9PD68	Dihydropyrimidinase-related protein 1	1.47E+06	9.30E+05	2.03E+06	1.53E+05	3.71E+05	2.62E+05
CRNKL1	Q5JY65	Crooked neck-like protein 1	4.41E+03	1.46E+04	0.00E+00	2.94E+05	1.39E+05	2.39E+05
CROCC	Q5TZA2	Rootletin	0.00E+00	6.69E+03	0.00E+00	3.27E+04	8.03E+04	2.17E+05
CRYAB	E9PR44	Alpha-crystallin B chain	2.07E+07	7.74E+06	7.06E+06	8.00E+06	9.32E+06	6.89E+06
CSDE1	O75534	Cold shock domain-containing protein E1	1.76E+05	1.32E+05	0.00E+00	4.11E+05	2.95E+05	2.51E+05
CSE1L	P55060-3	Exportin-2	6.11E+05	7.73E+04	2.34E+05	1.95E+05	1.17E+05	2.85E+04
CSK	P41240	Tyrosine-protein kinase CSK	2.71E+04	5.16E+04	0.00E+00	1.33E+05	1.92E+05	1.33E+05
CSNK1A1	P48729-3	Casein kinase I isoform alpha	6.76E+04	1.11E+04	0.00E+00	6.65E+04	9.43E+04	1.05E+05
CSNK2A1	E7EU96	Casein kinase II subunit alpha;Casein kinase II subunit alpha 3	2.68E+06	1.94E+06	2.33E+06	4.53E+04	8.03E+04	1.81E+05
CSRP1	P21291	Cysteine and glycine-rich protein 1	3.10E+06	1.88E+06	4.59E+05	1.76E+05	3.46E+04	1.35E+05
CTBP1	Q13363-2	C-terminal-binding protein 1	6.57E+04	0.00E+00	7.42E+04	0.00E+00	9.30E+03	0.00E+00
CTCF	P49711	Transcriptional repressor CTCF	4.11E+04	0.00E+00	0.00E+00	3.80E+04	0.00E+00	0.00E+00
CTDP1	A0A0J9YWB6	RNA polymerase II subunit A C-terminal domain phosphatase	4.05E+04	0.00E+00	0.00E+00	0.00E+00	4.70E+04	0.00E+00
CTNNA1	P35221	Catenin alpha-1	1.17E+06	9.96E+05	4.85E+05	1.51E+06	1.77E+06	2.52E+06

CTNNA2	P26232-2	Catenin alpha-2	3.22E+05	4.92E+05	1.01E+05	1.66E+05	7.02E+04	2.40E+05
CTNNB1	B4DGU4	Catenin beta-1	5.69E+05	1.14E+06	7.02E+05	1.40E+06	1.12E+06	1.25E+06
CTNND1	O60716	Catenin delta-1	1.28E+05	7.87E+04	2.95E+05	5.24E+05	2.82E+05	7.06E+05
CTPS1	P17812-2	CTP synthase 1	4.88E+04	6.60E+04	4.21E+04	3.23E+04	0.00E+00	4.71E+04
CTR9	Q6PD62	RNA polymerase-associated protein CTR9 homolog	4.58E+04	0.00E+00	9.82E+04	3.31E+05	1.57E+05	2.06E+05
CTSD	A0A1B0GW44	Cathepsin D;Cathepsin D light chain;Cathepsin D heavy chain	3.74E+06	1.48E+06	2.31E+06	1.59E+06	1.38E+06	3.11E+05
CTTN	Q14247	Src substrate cortactin	3.70E+05	2.71E+05	2.51E+04	7.82E+05	2.67E+05	3.51E+05
CUL1	A0A0C4DGX4	Cullin-1	2.60E+04	0.00E+00	0.00E+00	0.00E+00	6.47E+03	0.00E+00
CYB5R3	P00387-2	NADH-cytochrome b5 reductase 3;NADH-cytochrome b5 reductase 3 membrane-bound form;NADH-cytochrome b5 reductase 3 soluble form	7.23E+06	4.40E+06	4.26E+06	4.46E+04	4.13E+04	4.57E+04
CYFIP2	E7EVJ5	Cytoplasmic FMR1-interacting protein 2	1.53E+06	4.44E+05	7.95E+05	1.06E+05	6.64E+04	8.00E+04
CYP51A1	A0A0C4DFL7	Lanosterol 14-alpha demethylase	3.01E+04	0.00E+00	0.00E+00	0.00E+00	1.11E+04	6.02E+03
DAB2IP	H0Y3A3	Disabled homolog 2-interacting protein	5.28E+03	9.79E+03	9.28E+03	8.91E+04	1.61E+04	9.21E+04
DAG1	Q14118	Dystroglycan;Alpha-dystroglycan;Beta-dystroglycan	8.25E+04	7.69E+04	5.60E+04	2.27E+04	0.00E+00	0.00E+00
DARS	P14868	Aspartate--tRNA ligase, cytoplasmic	1.41E+06	4.23E+05	1.95E+05	4.96E+05	7.68E+04	4.13E+05
DBN1	Q16643	Drebrin	2.29E+05	4.42E+05	3.87E+05	4.86E+04	0.00E+00	1.20E+05
DBNL	Q9UJU6	Drebrin-like protein	1.65E+05	2.15E+05	4.09E+05	5.90E+05	1.69E+05	3.46E+05
DBT	P11182	Lipoamide acyltransferase component of branched-chain alpha-keto acid dehydrogenase complex, mitochondrial	2.15E+05	5.91E+04	2.33E+05	2.83E+04	0.00E+00	0.00E+00
DCAF7	P61962	DDB1- and CUL4-associated factor 7	0.00E+00	4.11E+04	0.00E+00	0.00E+00	4.43E+04	4.06E+04
DCTN1	E7EX90	Dynactin subunit 1	1.75E+06	9.17E+05	9.63E+05	2.00E+05	1.11E+05	7.30E+04
DCTN4	Q9UJW0	Dynactin subunit 4	9.61E+04	1.36E+05	1.42E+04	8.69E+04	0.00E+00	7.61E+04
DDAH1	O94760	N(G),N(G)-dimethylarginine dimethylaminohydrolase 1	2.35E+06	1.61E+06	1.47E+06	0.00E+00	5.99E+03	0.00E+00
DDB1	Q16531	DNA damage-binding protein 1	2.70E+05	2.31E+05	1.77E+05	4.73E+04	2.08E+05	3.03E+04
DDRGK1	Q96HY6	DDRGK domain-containing protein 1	1.16E+05	1.35E+05	2.78E+05	6.24E+04	0.00E+00	0.00E+00
DDX1	Q92499	ATP-dependent RNA helicase DDX1	1.04E+06	2.86E+05	4.08E+05	6.27E+05	1.17E+05	2.08E+05
DDX17	A0A1X7SBZ2	Probable ATP-dependent RNA helicase DDX17	6.86E+05	4.62E+05	2.04E+05	4.66E+06	3.31E+06	5.33E+06
DDX18	Q9NVP1	ATP-dependent RNA helicase DDX18	0.00E+00	1.65E+05	3.29E+04	3.80E+05	3.50E+05	6.34E+05
DDX23	Q9BUQ8	Probable ATP-dependent RNA helicase DDX23	1.15E+05	4.37E+04	1.19E+05	2.50E+05	1.12E+05	3.95E+05
DDX24	G3V529	ATP-dependent RNA helicase DDX24	4.97E+03	6.48E+04	5.53E+04	1.12E+06	8.19E+05	5.77E+05
DDX27	Q96GQ7	Probable ATP-dependent RNA helicase DDX27	1.89E+04	5.44E+04	0.00E+00	7.14E+04	1.47E+05	4.24E+05
DDX39A	O00148	ATP-dependent RNA helicase DDX39A	3.08E+04	0.00E+00	0.00E+00	0.00E+00	2.43E+04	0.00E+00
DDX39B	Q13838-2	Spliceosome RNA helicase DDX39B	1.11E+06	2.44E+05	4.00E+05	3.40E+05	3.77E+05	3.40E+05

DDX3X	A0A0D9SFB3	ATP-dependent RNA helicase DDX3X	2.50E+05	1.71E+05	1.39E+05	4.53E+05	2.58E+05	3.99E+05
DDX3Y	O15523	ATP-dependent RNA helicase DDX3Y	0.00E+00	3.12E+04	0.00E+00	1.44E+05	6.16E+04	9.29E+04
DDX41	J3KNN5	Probable ATP-dependent RNA helicase DDX41	2.09E+03	1.51E+03	7.05E+03	9.90E+04	1.30E+05	1.69E+05
DDX46	A0A0C4DG89	Probable ATP-dependent RNA helicase DDX46	4.09E+04	3.70E+04	0.00E+00	9.02E+05	1.18E+05	7.90E+05
DDX5	J3KTA4	Probable ATP-dependent RNA helicase DDX5	5.78E+05	1.74E+05	0.00E+00	6.45E+05	9.56E+05	1.08E+06
DDX58	A2A376	Probable ATP-dependent RNA helicase DDX58	2.09E+04	7.29E+04	2.51E+04	1.79E+05	3.07E+04	1.28E+05
DDX6	P26196	Probable ATP-dependent RNA helicase DDX6	3.96E+04	0.00E+00	0.00E+00	1.98E+04	0.00E+00	8.89E+04
DEK	P35659	Protein DEK	4.93E+03	6.54E+04	0.00E+00	4.39E+05	2.23E+04	5.13E+05
DES	P17661	Desmin	2.12E+05	0.00E+00	3.33E+04	6.80E+05	0.00E+00	0.00E+00
DHX15	O43143	Pre-mRNA-splicing factor ATP-dependent RNA helicase DHX15	5.68E+05	4.11E+05	4.27E+05	4.02E+05	6.70E+05	1.33E+06
DHX30	H7BXY3	Putative ATP-dependent RNA helicase DHX30	7.14E+04	1.04E+05	1.57E+05	6.75E+04	8.48E+04	3.33E+05
DHX36	E7EWK3	ATP-dependent RNA helicase DHX36	5.35E+04	0.00E+00	8.01E+04	4.48E+04	2.47E+04	7.61E+04
DHX38	Q92620	Pre-mRNA-splicing factor ATP-dependent RNA helicase PRP16	5.94E+04	1.75E+04	4.31E+04	1.74E+05	1.42E+05	4.18E+05
DHX9	Q08211	ATP-dependent RNA helicase A	1.23E+07	8.48E+06	7.86E+06	5.75E+06	3.95E+06	7.94E+06
DIP2B	Q9P265	Disco-interacting protein 2 homolog B	2.53E+05	1.27E+04	1.81E+05	1.97E+04	0.00E+00	0.00E+00
DIS3	Q9Y2L1	Exosome complex exonuclease RRP44	1.34E+05	6.87E+04	0.00E+00	2.90E+05	5.53E+05	1.23E+06
DKC1	O60832	H/ACA ribonucleoprotein complex subunit 4	3.93E+05	1.27E+05	2.42E+05	4.94E+04	8.07E+04	6.36E+04
DKFZp566H1924	Q9UFM8	Neuroplastin	6.22E+05	1.96E+05	1.14E+06	1.88E+04	2.05E+04	0.00E+00
DKK3	F6SYF8	Dickkopf-related protein 3	4.62E+05	3.94E+05	3.30E+05	0.00E+00	1.35E+04	2.76E+04
DLAT	P10515	Dihydrolipoyllysine-residue acetyltransferase component of pyruvate dehydrogenase complex, mitochondrial;Acetyltransferase component of pyruvate dehydrogenase complex	2.22E+06	1.92E+06	2.25E+06	1.26E+04	0.00E+00	0.00E+00
DLD	P09622	Dihydrolipoyl dehydrogenase, mitochondrial;Dihydrolipoyl dehydrogenase	2.69E+06	2.74E+06	2.72E+06	1.40E+04	0.00E+00	0.00E+00
DLST	P36957	Dihydrolipoyllysine-residue succinyltransferase component of 2-oxoglutarate dehydrogenase complex, mitochondrial	3.21E+06	2.58E+06	1.97E+06	9.58E+04	2.44E+05	0.00E+00
DNAJA1	P31689	DnaJ homolog subfamily A member 1	6.15E+05	1.13E+05	6.42E+05	3.14E+05	2.67E+05	1.17E+06
DNAJA2	O60884	DnaJ homolog subfamily A member 2	2.60E+05	3.35E+05	4.08E+05	2.88E+05	2.87E+05	3.60E+05
DNAJA3	Q96EY1	DnaJ homolog subfamily A member 3, mitochondrial	6.55E+03	8.47E+03	1.03E+05	1.96E+05	2.47E+05	4.12E+05
DNAJB11	Q9UBS4	DnaJ homolog subfamily B member 11	9.33E+05	6.59E+05	3.20E+05	9.85E+03	1.90E+04	1.65E+04
DNAJB2	C9JRD2	DnaJ homolog subfamily B member 2	1.56E+05	1.80E+05	1.64E+05	2.42E+05	1.66E+05	0.00E+00
DNAJB4	Q9UDY4	DnaJ homolog subfamily B member 4	3.44E+05	0.00E+00	3.96E+03	1.29E+05	5.86E+04	5.89E+04
DNAJB6	A0A0J9YX62	DnaJ homolog subfamily B member 6	1.57E+05	7.87E+04	6.24E+04	4.10E+05	9.43E+05	2.84E+05

DNAJC10	Q8IXB1	DnaJ homolog subfamily C member 10	7.21E+04	7.73E+04	8.68E+03	0.00E+00	2.01E+04	3.03E+03
DNAJC13	O75165	DnaJ homolog subfamily C member 13	1.24E+05	7.82E+04	9.92E+04	1.38E+04	0.00E+00	0.00E+00
DNAJC3	Q13217	DnaJ homolog subfamily C member 3	2.44E+05	1.03E+04	0.00E+00	2.04E+04	0.00E+00	0.00E+00
DNAJC7	Q99615	DnaJ homolog subfamily C member 7	1.05E+06	0.00E+00	0.00E+00	5.47E+04	4.78E+04	2.50E+04
DNPEP	E7ETB3	Aspartyl aminopeptidase	7.18E+04	1.03E+04	5.11E+04	8.22E+03	0.00E+00	9.24E+03
DOCK4	H0Y599	Dedicator of cytokinesis protein 4	5.18E+04	0.00E+00	0.00E+00	1.57E+05	6.38E+04	2.07E+05
DOCK9	A0A0A0MSY4	Dedicator of cytokinesis protein 9	1.73E+05	0.00E+00	1.22E+05	1.79E+05	0.00E+00	2.24E+05
DPYSL2	A0A1C7CYX9	Dihydropyrimidinase-related protein 2	5.31E+07	4.05E+07	3.38E+07	6.61E+06	8.23E+06	7.81E+06
DPYSL3	Q14195	Dihydropyrimidinase-related protein 3	5.66E+05	5.33E+05	4.54E+05	5.08E+04	7.48E+04	1.06E+05
DPYSL4	O14531	Dihydropyrimidinase-related protein 4	2.39E+06	6.63E+05	7.64E+05	1.45E+05	2.65E+05	2.32E+05
DRG2	A8MZF9	Developmentally-regulated GTP-binding protein 2	2.60E+04	0.00E+00	2.07E+04	4.94E+04	1.91E+04	0.00E+00
DST	E9PHM6	Dystonin	9.17E+05	5.39E+05	6.28E+05	1.24E+05	1.61E+05	2.20E+05
DSTN	F6RFD5	Destrin	5.09E+04	5.52E+04	1.34E+05	1.23E+05	2.51E+05	3.85E+05
DUSP3	P51452	Dual specificity protein phosphatase 3	1.95E+05	1.22E+05	1.85E+05	0.00E+00	6.15E+04	0.00E+00
DYNC1H1	Q14204	Cytoplasmic dynein 1 heavy chain 1	0.00E+00	1.02E+07	1.08E+07	6.35E+06	6.48E+06	7.04E+06
DYNC1I2	Q13409	Cytoplasmic dynein 1 intermediate chain 2	7.51E+05	2.21E+05	2.19E+05	4.16E+04	9.22E+04	1.42E+05
DYNC1LI1	Q9Y6G9	Cytoplasmic dynein 1 light intermediate chain 1	5.19E+05	2.23E+05	2.54E+05	9.41E+04	1.61E+05	2.90E+05
DYNC1LI2	O43237	Cytoplasmic dynein 1 light intermediate chain 2	1.74E+06	5.46E+05	5.18E+05	8.06E+05	1.65E+05	7.21E+05
ECHDC1	Q9NTX5-6	Ethylmalonyl-CoA decarboxylase	4.34E+05	2.33E+04	3.43E+04	0.00E+00	4.97E+04	3.39E+04
ECHS1	P30084	Enoyl-CoA hydratase, mitochondrial	5.12E+06	1.93E+06	4.36E+06	2.96E+04	0.00E+00	0.00E+00
EEA1	Q15075	Early endosome antigen 1	1.39E+05	1.86E+05	1.16E+05	1.58E+04	9.05E+03	0.00E+00
EEF1A1P5	Q5VTE0	Putative elongation factor 1-alpha-like 3;Elongation factor 1-alpha 1;Elongation factor 1-alpha	7.56E+06	3.01E+06	3.28E+06	7.61E+06	3.77E+06	3.74E+06
EEF1A2	Q05639	Elongation factor 1-alpha 2	8.92E+05	3.02E+05	3.74E+05	1.28E+05	2.11E+05	9.17E+04
EEF1B2	P24534	Elongation factor 1-beta	3.39E+05	1.01E+05	3.92E+05	8.61E+03	0.00E+00	0.00E+00
EEF2	P13639	Elongation factor 2	4.16E+05	1.29E+05	3.53E+05	8.20E+04	2.63E+04	2.76E+04
EFEMP1	Q12805-2	EGF-containing fibulin-like extracellular matrix protein 1	1.07E+05	0.00E+00	3.34E+04	9.37E+04	6.31E+04	8.77E+04
EFTUD2	Q15029-2	116 kDa U5 small nuclear ribonucleoprotein component	1.91E+06	1.29E+06	1.07E+06	4.33E+05	5.13E+05	7.23E+05
EHD1	A0A024R571	EH domain-containing protein 1	3.57E+06	3.42E+05	2.33E+05	1.22E+05	1.75E+05	1.57E+05
EHD2	Q9NZN4	EH domain-containing protein 2	1.09E+06	5.83E+05	5.47E+05	1.14E+06	3.85E+05	5.88E+05
EHD3	Q9NZN3	EH domain-containing protein 3	3.72E+05	2.37E+05	2.17E+04	1.27E+05	1.60E+05	2.20E+05
EHD4	Q9H223	EH domain-containing protein 4	1.91E+04	4.11E+04	0.00E+00	1.30E+05	2.10E+05	2.15E+05
EIF2A	F8WAE5	Eukaryotic translation initiation factor 2A;Eukaryotic translation initiation factor 2A, N-terminally processed	6.22E+04	5.12E+03	0.00E+00	2.18E+05	0.00E+00	1.16E+05

EIF2AK2	P19525	Interferon-induced, double-stranded RNA-activated protein kinase	2.07E+05	2.16E+05	5.72E+04	2.61E+05	1.20E+05	1.31E+05
EIF2B4	A0A087WTA5	Translation initiation factor eIF-2B subunit delta	4.90E+04	0.00E+00	2.77E+05	1.58E+04	3.17E+04	0.00E+00
EIF2S1	P05198	Eukaryotic translation initiation factor 2 subunit 1	2.55E+05	1.02E+04	1.11E+05	2.17E+04	5.79E+04	1.01E+05
EIF3A	Q14152-2	Eukaryotic translation initiation factor 3 subunit A	6.81E+04	2.41E+04	0.00E+00	1.47E+04	0.00E+00	0.00E+00
EIF3B	P55884	Eukaryotic translation initiation factor 3 subunit B	7.35E+02	8.60E+03	3.69E+04	3.64E+04	0.00E+00	0.00E+00
EIF3C	Q99613-2	Eukaryotic translation initiation factor 3 subunit C;Eukaryotic translation initiation factor 3 subunit C-like protein	2.43E+05	1.33E+05	0.00E+00	8.61E+04	3.42E+03	8.11E+04
EIF3G	K7EL20	Eukaryotic translation initiation factor 3 subunit G	3.39E+04	2.36E+04	0.00E+00	4.46E+04	0.00E+00	0.00E+00
EIF3I	Q13347	Eukaryotic translation initiation factor 3 subunit I	7.37E+04	3.71E+04	0.00E+00	3.62E+04	2.84E+04	6.78E+04
EIF4A1	P60842	Eukaryotic initiation factor 4A-I	5.56E+05	3.76E+05	1.49E+06	2.36E+05	3.74E+05	2.11E+05
EIF4A2	Q14240	Eukaryotic initiation factor 4A-II;Eukaryotic initiation factor 4A-II, N-terminally processed	2.29E+05	1.95E+04	0.00E+00	0.00E+00	7.48E+04	8.84E+04
EIF4A3	P38919	Eukaryotic initiation factor 4A-III;Eukaryotic initiation factor 4A-III, N-terminally processed	1.10E+06	1.27E+06	6.75E+05	2.09E+04	3.23E+05	2.21E+05
EIF4E	D6RBW1	Eukaryotic translation initiation factor 4E	3.14E+04	3.86E+04	0.00E+00	1.57E+04	0.00E+00	3.27E+04
EIF4G1	E7EX73	Eukaryotic translation initiation factor 4 gamma 1	3.32E+04	1.39E+05	0.00E+00	6.02E+04	6.03E+04	1.37E+04
EIF4H	Q15056-2	Eukaryotic translation initiation factor 4H	4.17E+05	3.18E+05	5.37E+04	1.53E+06	9.57E+04	2.83E+05
EIF5	P55010	Eukaryotic translation initiation factor 5	6.58E+04	6.01E+03	0.00E+00	3.19E+04	0.00E+00	0.00E+00
EIF5A	I3L397	Eukaryotic translation initiation factor 5A;Eukaryotic translation initiation factor 5A-1;Eukaryotic translation initiation factor 5A-2;Eukaryotic translation initiation factor 5A-1-like	1.73E+05	2.17E+05	1.95E+04	3.92E+04	0.00E+00	0.00E+00
EIF6	P56537	Eukaryotic translation initiation factor 6	4.97E+04	2.99E+04	0.00E+00	0.00E+00	1.81E+05	3.11E+04
ELAVL1	Q15717	ELAV-like protein 1	1.44E+06	1.33E+06	8.99E+05	4.78E+04	2.05E+05	9.80E+04
ELMO1	Q92556	Engulfment and cell motility protein 1	9.16E+04	8.31E+04	1.07E+04	1.19E+05	7.04E+04	6.18E+04
ELMO2	Q5JVZ5	Engulfment and cell motility protein 2	6.01E+04	0.00E+00	0.00E+00	2.22E+04	2.35E+04	2.90E+04
EMC2	Q15006	ER membrane protein complex subunit 2	3.37E+05	7.28E+04	3.03E+04	0.00E+00	2.45E+04	2.00E+04
EMD	P50402	Emerin	6.98E+05	3.81E+05	8.31E+05	9.10E+05	8.16E+05	8.08E+05
ENDOD1	O94919	Endonuclease domain-containing 1 protein	2.00E+06	3.16E+06	8.93E+05	2.25E+05	1.27E+05	1.82E+05
ENO1	P06733	Alpha-enolase	1.61E+07	1.12E+07	1.15E+07	3.04E+05	1.38E+05	9.54E+04
ENO2	P09104	Gamma-enolase;Enolase	1.05E+07	5.84E+06	9.65E+06	0.00E+00	2.03E+04	5.51E+03
EPB41L2	O43491-4	Band 4.1-like protein 2	2.69E+06	1.24E+06	1.03E+05	9.65E+05	1.08E+06	6.69E+05
EPM2AIP1	Q7L775	EPM2A-interacting protein 1	7.45E+05	3.14E+04	5.86E+04	3.35E+05	5.65E+04	2.15E+05
EPRS	P07814	Bifunctional glutamate/proline--tRNA ligase;Glutamate--tRNA ligase;Proline--tRNA ligase	1.05E+06	1.05E+05	5.73E+05	1.91E+05	0.00E+00	4.31E+04

EPS15L1	M0R165	Epidermal growth factor receptor substrate 15-like 1	5.54E+05	2.95E+05	9.80E+04	0.00E+00	1.23E+04	2.12E+04
EPS8	Q12929	Epidermal growth factor receptor kinase substrate 8	3.15E+04	3.95E+04	2.05E+03	8.60E+04	1.55E+04	4.02E+04
ERC1	G8JLD3	ELKS/Rab6-interacting/CAST family member 1	0.00E+00	5.02E+03	0.00E+00	6.87E+04	1.42E+05	8.99E+04
ERCC3	P19447	TFIIH basal transcription factor complex helicase XPB subunit	2.03E+04	0.00E+00	4.41E+03	0.00E+00	1.41E+04	0.00E+00
ERLIN1	O75477	Erlin-1	8.34E+04	2.12E+05	3.87E+05	6.79E+04	7.92E+04	0.00E+00
ERLIN2	O94905	Erlin-2	2.28E+06	3.20E+06	2.29E+06	3.77E+05	1.27E+06	2.91E+05
ESAM	Q96AP7	Endothelial cell-selective adhesion molecule	4.59E+04	1.02E+05	3.02E+05	2.65E+05	0.00E+00	1.02E+05
ESYT1	Q9BSJ8-2	Extended synaptotagmin-1	1.10E+06	8.67E+05	7.97E+05	1.63E+05	9.52E+04	1.24E+05
ESYT2	H7BXI1	Extended synaptotagmin-2	4.96E+05	7.59E+05	8.55E+05	1.59E+05	7.35E+04	2.40E+05
EWSR1	A0A0D9SFL3	RNA-binding protein EWS	1.06E+05	1.32E+05	2.08E+05	5.76E+05	5.17E+05	8.67E+05
EXOC1	Q9NV70-2	Exocyst complex component 1	5.16E+04	2.47E+04	0.00E+00	2.84E+04	1.81E+04	5.34E+04
EXOC3	D6RB59	Exocyst complex component 3	3.45E+04	1.96E+04	1.23E+05	0.00E+00	3.93E+03	1.35E+04
EXOC4	Q96A65	Exocyst complex component 4	1.60E+05	5.80E+04	2.78E+05	0.00E+00	4.76E+04	0.00E+00
EXOC5	O00471	Exocyst complex component 5	1.19E+05	7.26E+04	0.00E+00	3.30E+04	1.91E+04	4.70E+03
EXOC6B	A0A0U1RRB6	Exocyst complex component 6B	1.13E+05	0.00E+00	0.00E+00	2.04E+04	0.00E+00	0.00E+00
EXOC7	B4DJ07	Exocyst complex component 7	4.64E+05	1.50E+05	4.25E+04	1.30E+05	2.81E+04	9.32E+04
EXOC8	Q8IYI6	Exocyst complex component 8	7.80E+04	5.45E+04	6.07E+04	3.01E+04	1.74E+04	0.00E+00
FAF2	Q96CS3	FAS-associated factor 2	2.49E+05	3.63E+04	1.56E+05	0.00E+00	3.98E+04	3.87E+04
FAM120A	Q9NZB2-4	Constitutive coactivator of PPAR-gamma-like protein 1	8.58E+05	1.11E+06	1.39E+06	2.99E+05	2.53E+05	4.90E+05
FAM129A	Q9BZQ8	Protein Niban	9.25E+03	0.00E+00	0.00E+00	2.84E+04	0.00E+00	0.00E+00
FAM21A	F8W7U3	WASH complex subunit FAM21C;WASH complex subunit FAM21A	7.96E+04	0.00E+00	4.01E+04	5.01E+03	0.00E+00	5.87E+03
FAM49A	Q9H0Q0	Protein FAM49A	2.59E+05	1.76E+05	8.26E+04	8.98E+04	1.67E+04	0.00E+00
FAM49B	Q9NUQ9	Protein FAM49B	1.85E+06	6.59E+05	1.18E+06	0.00E+00	1.50E+04	0.00E+00
FAM98A	E9PH82	Protein FAM98A	6.12E+05	4.46E+05	5.58E+04	9.22E+04	8.65E+04	7.40E+04
FAM98B	Q52LJ0-2	Protein FAM98B	8.01E+05	7.97E+04	6.33E+04	8.76E+05	9.14E+04	2.88E+05
FASN	P49327	Fatty acid synthase;[Acyl-carrier-protein] S-acetyltransferase;[Acyl-carrier-protein] S-malonyltransferase;3-oxoacyl-[acyl-carrier-protein] synthase;3-oxoacyl-[acyl-carrier-protein] reductase;3-hydroxyacyl-[acyl-carrier-protein] dehydratase;Enoyl-[acyl-carrier-protein] reductase;Oleoyl-[acyl-carrier-protein] hydrolase	8.42E+05	4.70E+05	4.24E+05	1.38E+05	3.07E+04	0.00E+00
FBL	P22087	rRNA 2-O-methyltransferase fibrillarin	3.53E+05	5.51E+05	4.15E+05	1.46E+06	1.69E+06	2.41E+06
FBLL1	A6NHQ2	rRNA/tRNA 2-O-methyltransferase fibrillarin-like protein 1	1.53E+05	8.43E+04	1.40E+05	1.97E+05	1.89E+06	2.45E+06

FBN1	P35555	Fibrillin-1	4.30E+05	0.00E+00	0.00E+00	8.80E+05	1.58E+05	2.51E+04
FDXR	P22570-3	NADPH:adrenodoxin oxidoreductase, mitochondrial	1.58E+05	6.94E+04	0.00E+00	1.92E+05	0.00E+00	0.00E+00
FECH	P22830	Ferrochelatase, mitochondrial	4.25E+05	1.34E+05	2.11E+06	3.74E+04	2.11E+04	1.31E+04
FERMT2	A0A0U1RRM8	Fermitin family homolog 2	2.81E+05	5.68E+04	9.95E+04	2.38E+04	8.74E+03	1.78E+04
FH	P07954-2	Fumarate hydratase, mitochondrial	3.80E+06	1.99E+06	1.79E+06	0.00E+00	1.92E+04	0.00E+00
FHL1	Q13642-1	Four and a half LIM domains protein 1	8.62E+05	1.60E+05	4.75E+05	1.22E+05	5.03E+04	0.00E+00
FHL2	A0A0A0MSG2	Four and a half LIM domains protein 2	1.01E+05	2.22E+05	1.09E+04	3.89E+04	9.49E+04	6.21E+04
FIP1L1	Q6UN15-3	Pre-mRNA 3-end-processing factor FIP1	0.00E+00	1.38E+04	9.53E+04	6.42E+05	3.75E+05	7.04E+05
FKBP15	A0A0A0MT60	Peptidyl-prolyl cis-trans isomerase;FK506-binding protein 15	0.00E+00	1.99E+04	2.33E+04	9.17E+04	2.01E+04	9.92E+04
FKBP1A	P62942	Peptidyl-prolyl cis-trans isomerase FKBP1A;Peptidyl-prolyl cis-trans isomerase	7.58E+04	6.37E+04	0.00E+00	1.62E+04	0.00E+00	0.00E+00
FKBP2	P26885	Peptidyl-prolyl cis-trans isomerase FKBP2;Peptidyl-prolyl cis-trans isomerase	2.20E+05	1.28E+05	9.31E+04	3.56E+04	0.00E+00	5.79E+04
FKBP4	Q02790	Peptidyl-prolyl cis-trans isomerase FKBP4;Peptidyl-prolyl cis-trans isomerase FKBP4, N-terminally processed	2.91E+05	2.39E+05	0.00E+00	3.96E+04	0.00E+00	1.01E+03
FKBP5	Q13451-2	Peptidyl-prolyl cis-trans isomerase FKBP5;Peptidyl-prolyl cis-trans isomerase FKBP5, N-terminally processed	2.07E+04	0.00E+00	0.00E+00	5.11E+03	0.00E+00	1.46E+04
FKBP8	Q14318	Peptidyl-prolyl cis-trans isomerase FKBP8	1.58E+05	1.32E+05	5.12E+04	4.05E+03	5.44E+04	1.32E+05
FLII	Q13045-2	Protein flightless-1 homolog	3.74E+04	1.19E+04	1.09E+05	1.07E+05	2.32E+04	8.41E+04
FLNA	P21333-2	Filamin-A	1.29E+07	3.70E+06	3.31E+06	1.32E+07	3.08E+06	4.94E+06
FLNB	O75369-2	Filamin-B	1.01E+06	1.33E+05	4.79E+05	4.40E+05	4.56E+04	5.99E+05
FLNC	Q14315-2	Filamin-C	1.85E+05	7.09E+04	4.14E+04	6.33E+05	7.68E+04	2.09E+05
FLOT1	O75955	Flotillin-1	1.46E+06	1.00E+06	1.49E+06	0.00E+00	1.41E+05	9.62E+04
FLOT2	J3QLD9	Flotillin-2	1.55E+05	5.69E+05	2.67E+05	0.00E+00	2.54E+04	0.00E+00
FMNL2	Q96PY5-3	Formin-like protein 2	4.70E+05	3.50E+05	2.80E+05	0.00E+00	1.45E+05	1.34E+05
FN1	P02751	Fibronectin;Anastellin;Ugl-Y1;Ugl-Y2;Ugl-Y3	4.17E+05	4.56E+05	1.87E+04	5.46E+06	1.09E+07	1.11E+07
FNDC3A	Q9Y2H6-2	Fibronectin type-III domain-containing protein 3A	7.22E+04	1.22E+04	0.00E+00	4.00E+04	0.00E+00	2.92E+04
FSCN1	Q16658	Fascin	2.90E+06	1.08E+06	1.93E+06	2.93E+05	9.82E+04	2.44E+04
FTH1	P02794	Ferritin heavy chain;Ferritin heavy chain, N-terminally processed;Ferritin	9.47E+06	4.49E+06	5.57E+06	2.83E+06	2.03E+06	1.87E+06
FTL	P02792	Ferritin light chain	6.47E+06	1.71E+06	4.51E+06	2.31E+05	6.99E+04	1.03E+05
FUBP1	E9PEB5	Far upstream element-binding protein 1	0.00E+00	3.79E+04	0.00E+00	9.07E+04	0.00E+00	3.40E+05
FUBP3	Q96I24	Far upstream element-binding protein 3	4.16E+05	1.02E+05	1.36E+05	1.06E+06	7.33E+05	1.42E+06
FUS	P35637	RNA-binding protein FUS	4.52E+05	0.00E+00	3.59E+05	1.26E+06	6.21E+05	9.59E+05

FXR1	B4DXZ6	Fragile X mental retardation syndrome-related protein 1	4.31E+04	1.37E+04	2.65E+04	1.43E+05	7.52E+04	5.90E+04
FXR2	P51116	Fragile X mental retardation syndrome-related protein 2	7.16E+04	0.00E+00	5.43E+04	3.90E+03	0.00E+00	5.53E+04
FYTTD1	Q96QD9-2	UAP56-interacting factor	1.79E+04	0.00E+00	3.30E+04	2.55E+05	2.05E+05	2.29E+05
G3BP1	Q13283	Ras GTPase-activating protein-binding protein 1	1.73E+05	7.07E+04	4.77E+04	3.17E+05	0.00E+00	2.78E+05
G3BP2	Q9UN86-2	Ras GTPase-activating protein-binding protein 2	3.86E+05	0.00E+00	2.44E+05	1.81E+05	0.00E+00	9.70E+04
G6PD	P11413	Glucose-6-phosphate 1-dehydrogenase	1.27E+05	3.14E+04	1.44E+05	2.85E+04	3.15E+03	5.12E+03
GAK	O14976	Cyclin-G-associated kinase	2.66E+04	0.00E+00	4.33E+04	2.08E+04	2.77E+04	5.11E+04
GALK1	P51570	Galactokinase	1.65E+05	1.75E+05	9.93E+04	2.79E+05	4.26E+05	4.41E+05
GAPDH	P04406	Glyceraldehyde-3-phosphate dehydrogenase	5.78E+07	2.74E+07	4.42E+07	1.39E+06	7.30E+05	2.89E+05
GAPVD1	F8W9S7	GTPase-activating protein and VPS9 domain-containing protein 1	8.88E+04	7.91E+03	0.00E+00	6.48E+04	7.30E+04	8.04E+04
GART	P22102	Trifunctional purine biosynthetic protein adenosine-3;Phosphoribosylamine--glycine ligase;Phosphoribosylformylglycinamidine cyclo-ligase;Phosphoribosylglycinamide formyltransferase	8.40E+04	7.12E+04	3.73E+04	0.00E+00	1.24E+04	2.70E+03
GATAD2B	A0A0U1RRM1	Transcriptional repressor p66-beta	1.20E+05	7.79E+05	5.95E+04	1.02E+05	1.81E+05	4.01E+05
GBE1	E9PGM4	1,4-alpha-glucan-branching enzyme	6.81E+04	3.25E+04	0.00E+00	2.52E+05	0.00E+00	3.24E+04
GCLM	P48507	Glutamate--cysteine ligase regulatory subunit	7.49E+03	0.00E+00	0.00E+00	0.00E+00	2.12E+04	1.32E+04
GDI1	P31150	Rab GDP dissociation inhibitor alpha	1.62E+07	7.04E+06	7.56E+06	6.69E+04	8.86E+04	0.00E+00
GDI2	P50395	Rab GDP dissociation inhibitor beta	1.57E+06	1.13E+06	4.24E+05	0.00E+00	8.77E+03	0.00E+00
GEMIN5	Q8TEQ6	Gem-associated protein 5	7.63E+03	0.00E+00	0.00E+00	2.11E+04	2.19E+05	0.00E+00
GFAP	P14136-2	Glial fibrillary acidic protein	1.41E+07	5.48E+06	9.03E+06	1.27E+06	1.68E+06	1.29E+06
GFM1	Q96RP9-2	Elongation factor G, mitochondrial	5.19E+05	1.33E+05	3.79E+04	7.52E+03	0.00E+00	0.00E+00
GIMAP1	Q8WWP7	GTPase IMAP family member 1	7.61E+04	1.29E+05	9.55E+04	0.00E+00	1.25E+04	0.00E+00
GIMAP1-GIMAP5	A0A087WTJ2	GTPase IMAP family member 5	1.73E+05	3.38E+05	2.38E+05	3.42E+05	3.67E+05	4.04E+05
GIMAP7	Q8NHV1	GTPase IMAP family member 7	1.58E+04	2.87E+04	0.00E+00	1.02E+05	5.38E+04	1.01E+05
GIMAP8	Q8ND71	GTPase IMAP family member 8	1.83E+05	0.00E+00	0.00E+00	1.10E+05	2.46E+05	1.97E+05
GIPC1	K7EM11	PDZ domain-containing protein GIPC1	9.15E+02	9.92E+03	0.00E+00	7.27E+04	5.18E+04	1.39E+05
GIT1	J3QRU8	ARF GTPase-activating protein GIT1	3.19E+05	1.73E+05	8.66E+04	2.20E+04	1.32E+05	4.78E+05
GIT2	Q14161	ARF GTPase-activating protein GIT2	4.26E+04	3.42E+04	0.00E+00	4.76E+04	0.00E+00	4.49E+04
GJA1	P17302	Gap junction alpha-1 protein	1.02E+06	2.90E+05	1.78E+05	3.37E+04	1.89E+05	5.32E+04
GLIPR2	Q9H4G4	Golgi-associated plant pathogenesis-related protein 1	9.26E+05	5.25E+05	1.52E+05	4.56E+05	4.48E+05	2.54E+05
GLRX3	O76003	Glutaredoxin-3	5.63E+04	5.06E+03	4.50E+04	9.68E+03	0.00E+00	0.00E+00
GLRX5	Q86SX6	Glutaredoxin-related protein 5, mitochondrial	6.77E+04	3.21E+05	2.08E+05	1.24E+05	4.89E+04	2.56E+05

GLS	O94925	Glutaminase kidney isoform, mitochondrial	2.14E+06	1.81E+06	1.95E+06	9.92E+05	3.29E+05	9.38E+05
GLUD1	P00367	Glutamate dehydrogenase 1, mitochondrial;Glutamate dehydrogenase 2, mitochondrial	9.23E+06	7.64E+06	7.13E+06	0.00E+00	1.02E+04	2.33E+04
GLYR1	K7EMM8	Putative oxidoreductase GLYR1	3.25E+04	4.55E+03	3.51E+04	1.05E+06	6.70E+05	6.34E+05
GNA11	P29992	Guanine nucleotide-binding protein subunit alpha-11	5.06E+05	3.92E+05	3.84E+05	3.45E+05	4.30E+04	1.41E+05
GNA13	Q14344-2	Guanine nucleotide-binding protein subunit alpha-13	7.89E+05	2.59E+05	2.44E+05	5.40E+04	1.64E+05	5.00E+04
GNAI1	P63096	Guanine nucleotide-binding protein G(i) subunit alpha-1	1.31E+06	1.16E+06	1.46E+06	6.56E+04	1.89E+05	1.38E+05
GNAI2	P04899	Guanine nucleotide-binding protein G(i) subunit alpha-2	3.62E+06	2.54E+06	2.37E+06	5.17E+05	1.03E+06	3.55E+05
GNAI3	P08754	Guanine nucleotide-binding protein G(k) subunit alpha	6.04E+05	2.94E+05	1.47E+05	1.23E+05	9.09E+04	0.00E+00
GNAL	P38405	Guanine nucleotide-binding protein G(olf) subunit alpha	6.47E+05	4.97E+05	7.34E+05	1.47E+05	2.34E+05	3.77E+05
GNAQ	P50148	Guanine nucleotide-binding protein G(q) subunit alpha	2.29E+06	1.27E+06	6.76E+05	3.11E+05	7.67E+05	7.06E+05
GNAS	P63092-3	Guanine nucleotide-binding protein G(s) subunit alpha isoforms short;Guanine nucleotide-binding protein G(s) subunit alpha isoforms XLas	1.45E+06	1.12E+06	1.05E+06	3.31E+05	4.77E+05	3.73E+05
GNB1	P62873	Guanine nucleotide-binding protein G(I)/G(S)/G(T) subunit beta-1	1.59E+07	5.42E+06	1.18E+07	5.46E+05	1.05E+05	1.51E+05
GNB2	P62879	Guanine nucleotide-binding protein G(I)/G(S)/G(T) subunit beta-2	4.47E+06	1.79E+06	3.82E+06	5.57E+04	1.70E+04	2.96E+04
GNL1	P36915	Guanine nucleotide-binding protein-like 1	6.15E+04	0.00E+00	1.24E+04	2.79E+05	9.39E+04	3.88E+04
GNL2	Q13823	Nucleolar GTP-binding protein 2	0.00E+00	8.42E+03	0.00E+00	0.00E+00	8.71E+03	2.16E+04
GOLGA3	Q08378	Golgin subfamily A member 3	1.51E+04	8.84E+02	6.71E+04	2.37E+04	0.00E+00	0.00E+00
GOLGA4	H0Y6I0	Golgin subfamily A member 4	9.06E+04	1.70E+04	2.79E+05	6.39E+04	5.71E+04	2.21E+04
GOLGB1	Q14789-4	Golgin subfamily B member 1	3.76E+04	0.00E+00	7.53E+03	1.12E+05	0.00E+00	7.97E+03
GPD2	P43304	Glycerol-3-phosphate dehydrogenase, mitochondrial	1.61E+06	1.11E+06	1.02E+06	0.00E+00	1.29E+05	0.00E+00
GPX1	A0A087WUQ6	Glutathione peroxidase;Glutathione peroxidase 1	3.93E+05	2.73E+05	1.48E+05	0.00E+00	1.71E+04	5.32E+04
GPX4	K7ERP4	Glutathione peroxidase;Phospholipid hydroperoxide glutathione peroxidase, mitochondrial	2.97E+05	6.45E+05	0.00E+00	0.00E+00	1.05E+05	1.47E+05
GRHPR	Q9UBQ7	Glyoxylate reductase/hydroxypyruvate reductase	8.48E+05	7.72E+05	5.18E+05	2.00E+04	1.91E+04	1.21E+04
GRIPAP1	Q4V328	GRIP1-associated protein 1	2.30E+05	3.67E+05	0.00E+00	6.34E+05	5.75E+05	0.00E+00
GSK3B	P49841	Glycogen synthase kinase-3 beta	7.28E+04	8.98E+03	1.12E+03	7.89E+04	1.24E+04	6.92E+04
GSR	P00390-5	Glutathione reductase, mitochondrial	5.29E+05	1.68E+05	3.00E+05	2.29E+04	3.21E+04	3.55E+04
GSTK1	Q9Y2Q3-4	Glutathione S-transferase kappa 1	4.06E+05	6.90E+05	1.84E+05	9.42E+04	1.08E+05	1.18E+03
GSTM2	E9PHN6	Glutathione S-transferase Mu 2	7.65E+05	2.27E+05	1.48E+05	0.00E+00	1.60E+04	0.00E+00
GSTM3	P21266	Glutathione S-transferase Mu 3	3.65E+05	1.19E+05	2.39E+05	1.54E+05	4.97E+04	3.59E+04

GTF2I	P78347-2	General transcription factor II-I	1.87E+05	1.83E+05	5.21E+04	3.50E+06	2.19E+06	3.08E+06
GTF3C1	Q12789-3	General transcription factor 3C polypeptide 1	2.28E+04	2.03E+04	0.00E+00	3.01E+05	2.25E+05	3.22E+05
GTF3C3	Q9Y5Q9	General transcription factor 3C polypeptide 3	3.59E+04	0.00E+00	5.87E+03	3.97E+05	4.64E+05	1.57E+06
GTF3C4	Q9UKN8	General transcription factor 3C polypeptide 4	1.60E+05	1.20E+05	5.14E+04	0.00E+00	1.41E+04	5.77E+04
GTPBP1	O00178	GTP-binding protein 1	0.00E+00	6.72E+03	1.46E+04	1.10E+05	4.63E+04	1.01E+05
GUK1	B1ANH0	Guanylate kinase	2.79E+05	1.46E+05	1.38E+05	0.00E+00	1.57E+04	0.00E+00
GYS1	P13807-2	Glycogen [starch] synthase, muscle	2.55E+05	6.70E+04	1.21E+05	1.21E+05	7.22E+04	6.85E+04
H1FX	Q92522	Histone H1x	0.00E+00	5.96E+04	0.00E+00	1.03E+06	3.12E+06	1.96E+06
H2AFV	Q71UI9	Histone H2A.V;Histone H2A.Z;Histone H2A	9.30E+05	2.29E+05	1.64E+06	6.09E+06	3.03E+06	1.94E+06
H2AFY	O75367-2	Core histone macro-H2A.1;Histone H2A	2.65E+06	1.12E+06	1.68E+06	4.30E+07	4.35E+07	3.83E+07
H3F3B	K7EK07	Histone H3;Histone H3.3;Histone H3.2;Histone H3.1t;Histone H3.1	1.77E+04	5.68E+02	9.36E+03	9.02E+06	4.00E+06	4.70E+06
HACD3	Q9P035	Very-long-chain (3R)-3-hydroxyacyl-CoA dehydratase 3	3.82E+06	4.33E+05	1.28E+06	2.66E+05	2.78E+05	1.78E+05
HADHA	P40939	Trifunctional enzyme subunit alpha, mitochondrial;Long-chain enoyl-CoA hydratase;Long chain 3-hydroxyacyl-CoA dehydrogenase	7.47E+06	4.55E+06	4.64E+06	6.63E+05	2.07E+05	2.43E+05
HADHB	P55084-2	Trifunctional enzyme subunit beta, mitochondrial;3-ketoacyl-CoA thiolase	2.71E+06	2.45E+06	1.85E+06	3.49E+05	2.52E+05	1.43E+05
HBS1L	B7Z524	HBS1-like protein	2.18E+04	3.72E+04	0.00E+00	8.54E+04	3.11E+04	1.35E+05
HDAC2	Q92769-3	Histone deacetylase 2	5.44E+04	7.46E+04	1.78E+05	5.55E+05	6.41E+05	7.33E+05
HDGFRP3	A0A024R216	Hepatoma-derived growth factor-related protein 3	9.35E+04	4.05E+04	2.64E+04	9.91E+05	1.01E+06	1.77E+06
HDLBP	A0A024R4E5	Vigilin	1.31E+05	1.67E+04	6.46E+04	2.73E+05	1.45E+05	2.89E+05
HEATR1	Q9H583	HEAT repeat-containing protein 1;HEAT repeat-containing protein 1, N-terminally processed	5.09E+04	3.20E+04	0.00E+00	7.69E+04	7.28E+05	4.92E+05
HERC4	Q5GLZ8-6	Probable E3 ubiquitin-protein ligase HERC4	6.32E+05	0.00E+00	0.00E+00	0.00E+00	3.86E+04	2.51E+03
HGS	O14964	Hepatocyte growth factor-regulated tyrosine kinase substrate	1.14E+05	7.08E+04	1.99E+05	4.61E+04	0.00E+00	5.86E+04
HIP1R	O75146	Huntingtin-interacting protein 1-related protein	1.94E+05	8.04E+04	5.66E+04	2.99E+04	8.93E+03	1.40E+04
HIST1H2BN	U3KQK0	Histone H2B;Histone H2B type 1-L;Histone H2B type 1-M;Histone H2B type 1-N;Histone H2B type 1-H;Histone H2B type 2-F;Histone H2B type 1-C/E/F/G/I;Histone H2B type 1-D;Histone H2B type F-S;Histone H2B type 1-K;Histone H2B type 1-A	2.67E+06	1.23E+06	5.13E+05	3.29E+07	4.69E+07	3.72E+07
HIST1H4A	P62805	Histone H4	1.48E+07	1.19E+07	4.99E+06	2.30E+08	2.15E+08	2.59E+08
HIST2H2AB	Q8IUE6	Histone H2A type 2-B;Histone H2AX;Histone H2A type 1-A	1.16E+05	0.00E+00	1.23E+05	2.28E+06	9.41E+05	1.75E+06
HK1	P19367-4	Hexokinase-1	8.66E+06	7.19E+06	6.54E+06	0.00E+00	3.71E+05	3.37E+05

HLA-A	P30453	HLA class I histocompatibility antigen, A-34 alpha chain;HLA class I histocompatibility antigen, A-25 alpha chain;HLA class I histocompatibility antigen, A-66 alpha chain;HLA class I histocompatibility antigen, A-26 alpha chain;HLA class I histocompatibility antigen, A-33 alpha chain;HLA class I histocompatibility antigen, A-32 alpha chain;HLA class I histocompatibility antigen, A-31 alpha chain;HLA class I histocompatibility antigen, A-74 alpha chain;HLA class I histocompatibility antigen, A-43 alpha chain;HLA class I histocompatibility antigen, A-29 alpha chain	3.63E+05	0.00E+00	0.00E+00	5.83E+04	0.00E+00	0.00E+00
HMGA1	P17096	High mobility group protein HMG-I/HMG-Y	9.59E+04	1.65E+05	5.66E+04	1.92E+06	3.04E+06	4.18E+06
HMGB1	Q5T7C4	High mobility group protein B1;Putative high mobility group protein B1-like 1	5.70E+04	2.95E+05	0.00E+00	4.84E+04	0.00E+00	1.07E+05
HMOX2	A0A087WT44	Heme oxygenase 2	1.22E+06	2.06E+05	8.77E+05	3.65E+04	0.00E+00	1.07E+04
HNRNPA0	Q13151	Heterogeneous nuclear ribonucleoprotein A0	1.52E+06	7.06E+05	4.34E+05	1.33E+06	1.33E+06	1.92E+06
HNRNPA1	P09651-2	Heterogeneous nuclear ribonucleoprotein A1;Heterogeneous nuclear ribonucleoprotein A1, N-terminally processed;Heterogeneous nuclear ribonucleoprotein A1-like 2	3.87E+06	1.41E+06	1.91E+06	2.81E+06	1.73E+06	2.44E+06
HNRNPA2B1	P22626	Heterogeneous nuclear ribonucleoproteins A2/B1	1.02E+07	7.73E+06	6.23E+06	1.09E+07	8.02E+06	1.30E+07
HNRNPA3	P51991	Heterogeneous nuclear ribonucleoprotein A3	9.05E+06	1.08E+07	8.84E+06	5.83E+06	4.70E+06	4.49E+06
HNRNPAB	D6R9P3	Heterogeneous nuclear ribonucleoprotein A/B	3.54E+05	2.12E+06	1.23E+05	2.41E+05	4.75E+04	9.59E+05
HNRNPC	G3V4C1	Heterogeneous nuclear ribonucleoproteins C1/C2	4.96E+06	5.72E+06	8.01E+05	1.04E+07	4.20E+06	4.60E+06
HNRNPD	Q14103-3	Heterogeneous nuclear ribonucleoprotein D0	7.45E+06	8.14E+06	3.86E+06	4.56E+06	8.14E+06	1.34E+07
HNRNPDL	A0A087WUK2	Heterogeneous nuclear ribonucleoprotein D-like	5.21E+06	0.00E+00	3.68E+06	6.80E+05	6.62E+05	1.22E+06
HNRNPF	P52597	Heterogeneous nuclear ribonucleoprotein F;Heterogeneous nuclear ribonucleoprotein F, N-terminally processed	1.37E+06	4.52E+05	9.61E+05	1.42E+06	1.51E+06	1.27E+06
HNRNPH1	G8JLB6	Heterogeneous nuclear ribonucleoprotein H;Heterogeneous nuclear ribonucleoprotein H, N-terminally processed	5.93E+06	4.43E+06	4.00E+06	5.63E+06	9.45E+06	1.03E+07
HNRNPH2	P55795	Heterogeneous nuclear ribonucleoprotein H2	2.74E+06	8.24E+05	1.72E+06	5.17E+06	6.48E+06	6.99E+06
HNRNPH3	P31942	Heterogeneous nuclear ribonucleoprotein H3	2.48E+06	1.04E+06	2.18E+06	6.52E+06	8.83E+06	8.74E+06
HNRNPL	P14866	Heterogeneous nuclear ribonucleoprotein L	1.10E+07	7.33E+06	7.37E+06	1.92E+05	5.09E+05	7.87E+05
HNRNPLL	C9IYN3	Heterogeneous nuclear ribonucleoprotein L-like	4.38E+05	0.00E+00	1.44E+05	3.23E+05	4.39E+05	7.89E+05
HNRNPM	A0A087X0X3	Heterogeneous nuclear ribonucleoprotein M	1.86E+06	1.80E+06	1.46E+06	6.10E+06	7.71E+06	1.17E+07
HNRNPU	Q00839	Heterogeneous nuclear ribonucleoprotein U	7.68E+06	5.02E+06	3.23E+06	3.44E+07	0.00E+00	3.13E+07

HNRNPUL1	Q9BUJ2-2	Heterogeneous nuclear ribonucleoprotein U-like protein 1	1.79E+06	6.83E+05	4.72E+05	3.26E+06	2.56E+06	2.46E+06
HNRNPUL2	Q1KMD3	Heterogeneous nuclear ribonucleoprotein U-like protein 2	2.03E+06	8.23E+05	8.24E+05	5.77E+06	6.07E+06	1.02E+07
HOOK3	Q86VS8	Protein Hook homolog 3	1.65E+04	0.00E+00	6.26E+04	5.05E+04	2.94E+04	6.98E+04
HP1BP3	Q5SSJ5	Heterochromatin protein 1-binding protein 3	1.47E+06	5.42E+05	7.49E+05	1.12E+07	8.96E+06	6.38E+06
HRAS	P01112	GTPase HRas;GTPase HRas, N-terminally processed	3.36E+05	3.88E+05	2.36E+05	2.15E+04	0.00E+00	0.00E+00
HSD17B10	Q99714	3-hydroxyacyl-CoA dehydrogenase type-2	2.22E+06	5.24E+05	1.39E+06	9.51E+04	0.00E+00	8.52E+04
HSD17B4	P51659	Peroxisomal multifunctional enzyme type 2;(3R)-hydroxyacyl-CoA dehydrogenase;Enoyl-CoA hydratase 2	2.58E+06	1.36E+06	5.60E+05	2.37E+03	0.00E+00	0.00E+00
HSP90AA1	P07900	Heat shock protein HSP 90-alpha	1.38E+07	8.14E+06	9.51E+06	4.97E+06	2.86E+06	4.24E+06
HSP90AB1	P08238	Heat shock protein HSP 90-beta	0.00E+00	1.21E+06	1.54E+06	1.18E+06	5.35E+05	9.20E+05
HSP90B1	P14625	Endoplasmin	8.66E+06	3.83E+06	5.94E+06	4.58E+05	7.54E+04	7.14E+04
HSPA12B	Q5JX83	Heat shock 70 kDa protein 12B	3.44E+05	2.09E+05	5.22E+05	2.77E+05	1.27E+04	2.72E+05
HSPA1B	A0A0G2JIW1	Heat shock 70 kDa protein 1B;Heat shock 70 kDa protein 1A	3.33E+06	1.84E+06	2.68E+06	2.20E+06	1.30E+06	1.99E+06
HSPA2	P54652	Heat shock-related 70 kDa protein 2	3.76E+06	2.04E+06	1.30E+06	2.82E+06	2.49E+06	2.03E+06
HSPA4	P34932	Heat shock 70 kDa protein 4	1.52E+06	0.00E+00	1.06E+06	1.69E+05	1.38E+05	3.79E+05
HSPA4L	O95757	Heat shock 70 kDa protein 4L	7.38E+05	3.20E+05	5.45E+05	3.24E+05	0.00E+00	2.35E+05
HSPA5	P11021	78 kDa glucose-regulated protein	1.27E+07	8.24E+06	7.73E+06	1.44E+06	1.35E+06	1.60E+06
HSPA8	P11142	Heat shock cognate 71 kDa protein	1.88E+07	1.19E+07	1.90E+07	7.68E+06	8.46E+06	8.62E+06
HSPA9	P38646	Stress-70 protein, mitochondrial	7.39E+06	6.57E+06	5.23E+06	2.68E+06	1.20E+06	2.48E+06
HSPB1	P04792	Heat shock protein beta-1	2.12E+06	1.64E+06	8.55E+05	3.01E+06	2.78E+06	3.42E+06
HSPD1	P10809	60 kDa heat shock protein, mitochondrial	8.43E+06	7.95E+06	1.11E+07	6.25E+04	5.57E+04	3.49E+04
HSPG2	P98160	Basement membrane-specific heparan sulfate proteoglycan core protein;Endorepellin;LG3 peptide	1.62E+05	1.64E+05	4.39E+04	2.29E+07	2.84E+07	1.60E+07
HSPH1	Q92598-2	Heat shock protein 105 kDa	2.54E+06	1.51E+06	1.67E+06	4.60E+05	7.16E+05	9.92E+05
HTATSF1	O43719	HIV Tat-specific factor 1	2.19E+05	5.00E+04	0.00E+00	5.38E+05	2.61E+05	4.59E+05
HTRA1	Q92743	Serine protease HTRA1	3.95E+03	0.00E+00	0.00E+00	0.00E+00	7.41E+03	1.08E+05
HTT	P42858	Huntingtin	5.59E+04	0.00E+00	1.18E+05	5.76E+04	0.00E+00	0.00E+00
HUWE1	Q7Z6Z7-2	E3 ubiquitin-protein ligase HUWE1	5.81E+05	2.06E+05	1.86E+05	2.30E+05	4.23E+04	5.76E+03
HYOU1	Q9Y4L1	Hypoxia up-regulated protein 1	3.14E+06	1.28E+06	1.74E+06	2.02E+05	1.15E+05	2.35E+05
IARS2	Q9NSE4	Isoleucine--tRNA ligase, mitochondrial	6.73E+05	6.21E+05	6.28E+05	9.61E+04	7.78E+04	2.89E+05
IDH2	P48735	Isocitrate dehydrogenase [NADP], mitochondrial	5.05E+06	2.03E+06	4.48E+06	2.07E+06	6.60E+05	2.19E+06
IDH3A	P50213-2	Isocitrate dehydrogenase [NAD] subunit alpha, mitochondrial	3.06E+06	1.51E+06	3.29E+06	1.75E+05	1.72E+05	2.02E+05

IDH3B	A0A087WZN1	Isocitrate dehydrogenase [NAD] subunit, mitochondrial;Isocitrate dehydrogenase [NAD] subunit beta, mitochondrial	1.70E+06	1.05E+06	2.07E+06	0.00E+00	2.21E+04	6.13E+04
IDH3G	P51553	Isocitrate dehydrogenase [NAD] subunit gamma, mitochondrial;Isocitrate dehydrogenase [NAD] subunit, mitochondrial	1.60E+06	9.29E+05	9.38E+05	0.00E+00	1.78E+04	2.10E+04
IFI16	Q16666	Gamma-interferon-inducible protein 16	2.55E+04	0.00E+00	0.00E+00	1.53E+06	6.61E+05	8.64E+05
IFIT1	P09914-2	Interferon-induced protein with tetratricopeptide repeats 1	1.70E+05	2.06E+05	4.06E+04	6.95E+04	2.48E+05	3.03E+05
IFIT3	O14879	Interferon-induced protein with tetratricopeptide repeats 3	2.95E+04	3.18E+04	0.00E+00	2.38E+05	2.62E+05	2.60E+05
IFIT5	Q13325-2	Interferon-induced protein with tetratricopeptide repeats 5	8.58E+02	0.00E+00	5.38E+04	0.00E+00	4.72E+04	5.41E+04
IGBP1	P78318	Immunoglobulin-binding protein 1	1.44E+05	7.01E+04	0.00E+00	1.22E+03	2.32E+03	3.52E+04
IGHA1	A0A286YEY1	Ig alpha-1 chain C region;Ig alpha-2 chain C region	3.50E+04	5.46E+04	0.00E+00	0.00E+00	2.17E+04	0.00E+00
IGKC	P01834	Ig kappa chain C region	5.21E+05	9.40E+05	4.24E+05	4.22E+05	7.51E+04	2.93E+05
IK	Q13123	Protein Red	0.00E+00	2.29E+04	0.00E+00	0.00E+00	1.13E+04	4.52E+04
IKBKAP	F5H2T0	Elongator complex protein 1	6.82E+04	0.00E+00	2.53E+04	1.90E+04	0.00E+00	0.00E+00
IKBKG	A0A087X0G7	NF-kappa-B essential modulator	3.07E+04	0.00E+00	0.00E+00	7.77E+04	1.24E+04	9.40E+04
ILF2	B4DY09	Interleukin enhancer-binding factor 2	1.83E+06	2.08E+06	8.01E+05	2.46E+05	6.44E+05	8.10E+05
ILF3	Q12906-7	Interleukin enhancer-binding factor 3	3.56E+06	2.84E+06	1.99E+06	6.81E+05	3.24E+05	1.23E+06
ILK	A0A0A0MTH3	Integrin-linked protein kinase	1.34E+05	1.22E+05	5.44E+04	5.28E+05	1.92E+05	8.18E+05
IMPDH2	H0Y4R1	Inosine-5-monophosphate dehydrogenase 2	0.00E+00	8.65E+04	1.79E+04	1.09E+05	0.00E+00	8.42E+03
INF2	Q27J81-2	Inverted formin-2	1.12E+06	8.44E+05	6.47E+05	6.29E+05	4.24E+05	5.37E+05
INPP4A	Q96PE3-2	Type I inositol 3,4-bisphosphate 4-phosphatase	1.57E+04	0.00E+00	1.28E+04	5.87E+03	0.00E+00	0.00E+00
INTS1	Q8N201	Integrator complex subunit 1	0.00E+00	1.86E+04	3.84E+04	2.85E+05	2.14E+05	2.82E+04
INTS3	Q68E01-2	Integrator complex subunit 3	1.09E+04	0.00E+00	0.00E+00	1.06E+04	2.69E+04	2.47E+04
IPO7	O95373	Importin-7	7.06E+04	0.00E+00	5.98E+05	4.43E+04	0.00E+00	3.68E+03
IPO9	Q96P70	Importin-9	7.64E+04	2.12E+04	2.18E+04	7.33E+03	0.00E+00	1.15E+04
IQGAP1	P46940	Ras GTPase-activating-like protein IQGAP1	7.76E+05	3.31E+05	2.03E+05	3.18E+05	8.92E+04	3.83E+05
IRF9	Q00978	Interferon regulatory factor 9	5.96E+04	0.00E+00	0.00E+00	1.14E+05	9.23E+04	4.40E+04
ISY1	Q9ULR0	Pre-mRNA-splicing factor ISY1 homolog	0.00E+00	1.64E+04	1.72E+04	2.02E+05	1.95E+05	4.88E+04
ITCH	Q96J02-3	E3 ubiquitin-protein ligase Itchy homolog	4.63E+04	1.09E+04	0.00E+00	7.97E+04	1.15E+04	1.20E+04
ITGA6	P23229-4	Integrin alpha-6;Integrin alpha-6 heavy chain;Integrin alpha-6 light chain;Processed integrin alpha-6	9.90E+05	8.77E+05	6.34E+05	5.98E+04	0.00E+00	1.80E+04
ITPR3	Q14573	Inositol 1,4,5-trisphosphate receptor type 3	1.36E+04	2.39E+04	0.00E+00	1.28E+04	0.00E+00	0.00E+00
IVD	A0A0A0MT83	Isovaleryl-CoA dehydrogenase, mitochondrial	9.09E+04	8.24E+03	4.32E+04	8.94E+03	0.00E+00	7.95E+03

IWS1	Q96ST2-2	Protein IWS1 homolog	2.59E+04	0.00E+00	0.00E+00	8.32E+04	4.88E+03	3.46E+04
JMJD6	B2WTI3	Bifunctional arginine demethylase and lysyl-hydroxylase JMJD6	3.64E+04	0.00E+00	0.00E+00	1.02E+05	3.93E+04	9.42E+04
KANK3	Q6NY19-2	KN motif and ankyrin repeat domain-containing protein 3	1.77E+05	1.81E+05	1.07E+05	1.10E+06	5.43E+05	8.55E+05
KARS	Q15046	Lysine--tRNA ligase	2.17E+05	8.58E+04	1.80E+05	8.91E+03	1.72E+04	1.43E+05
KCTD12	Q96CX2	BTB/POZ domain-containing protein KCTD12	2.57E+05	1.39E+05	2.38E+05	2.93E+04	4.57E+04	1.11E+05
KDM3B	Q7LBC6	Lysine-specific demethylase 3B	1.65E+04	0.00E+00	4.07E+04	1.36E+05	6.80E+04	1.26E+05
KHDRBS1	Q07666-3	KH domain-containing, RNA-binding, signal transduction-associated protein 1	5.38E+05	1.28E+05	4.96E+04	1.92E+06	5.80E+05	1.63E+06
KHSRP	A0A087WTP3	Far upstream element-binding protein 2	4.67E+05	2.13E+04	8.42E+04	7.40E+05	0.00E+00	5.01E+05
KIAA0020	Q15397	Pumilio domain-containing protein KIAA0020	0.00E+00	5.46E+03	0.00E+00	0.00E+00	4.72E+03	9.57E+03
KIAA1598	A0MZ66-4	Shootin-1	2.22E+05	8.70E+04	7.95E+04	1.87E+05	1.05E+05	1.38E+05
KIF13B	Q9NQT8	Kinesin-like protein KIF13B	1.73E+04	0.00E+00	0.00E+00	6.30E+03	1.58E+04	1.28E+04
KIF1A	Q12756	Kinesin-like protein KIF1A;Kinesin-like protein	1.18E+05	1.17E+04	0.00E+00	8.49E+04	0.00E+00	9.32E+04
KIF2A	O00139-2	Kinesin-like protein KIF2A	1.09E+05	1.25E+05	1.23E+05	5.86E+05	2.76E+05	4.58E+05
KIF5B	P33176	Kinesin-1 heavy chain	4.53E+05	3.20E+05	1.13E+05	5.14E+05	1.61E+05	3.56E+05
KLC1	Q07866	Kinesin light chain 1	3.84E+05	3.36E+04	1.51E+05	1.93E+05	0.00E+00	7.03E+04
KLC2	Q9H0B6	Kinesin light chain 2	9.02E+04	0.00E+00	5.56E+04	2.42E+04	0.00E+00	4.73E+04
KLC4	Q9NSK0	Kinesin light chain 4	7.70E+04	0.00E+00	0.00E+00	3.99E+04	0.00E+00	5.14E+04
KPNA1	P52294	Importin subunit alpha-5;Importin subunit alpha-5, N-terminally processed	6.72E+05	3.44E+05	2.94E+05	4.54E+05	2.23E+05	5.83E+05
KPNA3	O00505	Importin subunit alpha-4	0.00E+00	1.33E+05	3.31E+04	4.79E+04	7.34E+04	0.00E+00
KPNA4	O00629	Importin subunit alpha-3	2.01E+05	4.64E+04	1.22E+05	1.52E+05	1.15E+05	3.23E+05
KPNA6	O60684	Importin subunit alpha-7;Importin subunit alpha-6	2.76E+05	1.73E+05	3.83E+04	6.36E+05	5.26E+05	4.20E+05
KPNB1	Q14974	Importin subunit beta-1	2.29E+06	9.48E+05	1.46E+06	1.96E+05	1.33E+05	1.23E+05
KRI1	Q8N9T8	Protein KRI1 homolog	0.00E+00	6.69E+03	9.33E+03	5.53E+04	3.07E+05	1.54E+05
KTN1	Q86UP2-4	Kinectin	5.34E+06	1.96E+06	1.65E+06	1.69E+05	0.00E+00	2.69E+04
LAMA4	A0A0A0MTC7	Laminin subunit alpha-4	6.57E+04	5.53E+04	0.00E+00	1.68E+06	1.77E+06	1.12E+06
LAMA5	O15230	Laminin subunit alpha-5	2.19E+05	1.37E+05	3.62E+03	1.60E+07	1.64E+07	1.20E+07
LAMB1	P07942	Laminin subunit beta-1	0.00E+00	1.17E+04	1.14E+04	7.17E+05	7.63E+05	6.28E+05
LAMB2	P55268	Laminin subunit beta-2	2.17E+06	2.01E+06	1.04E+06	4.54E+07	4.74E+07	3.88E+07
LAMC1	P11047	Laminin subunit gamma-1	2.02E+06	2.24E+06	1.32E+06	3.19E+07	3.90E+07	2.37E+07
LANCL2	Q9NS86	LanC-like protein 2	6.77E+05	3.30E+05	6.22E+05	7.14E+04	0.00E+00	2.90E+04
LARP1	Q6PKG0-3	La-related protein 1	5.00E+04	1.38E+04	7.64E+04	4.69E+04	1.61E+03	8.05E+04
LARS	Q9P2J5	Leucine--tRNA ligase, cytoplasmic	4.61E+05	1.89E+05	1.55E+05	1.09E+05	2.17E+04	1.58E+05

LAS1L	Q9Y4W2-2	Ribosomal biogenesis protein LAS1L	1.45E+05	1.42E+05	0.00E+00	3.23E+04	1.08E+05	1.75E+05
LASP1	Q14847-3	LIM and SH3 domain protein 1	8.41E+04	6.40E+04	1.04E+05	0.00E+00	2.86E+05	4.73E+05
LDHB	P07195	L-lactate dehydrogenase B chain;L-lactate dehydrogenase	9.14E+06	3.54E+06	2.22E+06	1.55E+05	9.54E+04	1.05E+05
LEMD2	Q8NC56	LEM domain-containing protein 2	1.73E+05	1.21E+04	4.86E+04	1.23E+05	1.15E+05	3.06E+05
LEMD3	Q9Y2U8	Inner nuclear membrane protein Man1	1.69E+05	1.47E+05	1.69E+05	4.36E+04	2.07E+04	8.20E+04
LGALS1	P09382	Galectin-1	1.90E+06	2.11E+06	1.99E+06	0.00E+00	8.84E+04	5.31E+04
LGALS9	O00182-3	Galectin-9	0.00E+00	2.08E+04	0.00E+00	2.85E+05	2.11E+05	1.58E+05
LIG3	P49916-4	DNA ligase 3	4.18E+04	5.28E+04	3.80E+04	3.55E+05	5.03E+05	5.71E+05
LIMA1	Q9UHB6	LIM domain and actin-binding protein 1	1.40E+04	1.01E+04	1.38E+04	3.39E+04	0.00E+00	1.86E+04
LIMCH1	Q9UPQ0	LIM and calponin homology domains-containing protein 1	2.18E+05	3.54E+05	7.00E+04	3.55E+05	1.81E+05	4.41E+05
LIMS1	A0A0J9YXC7	LIM and senescent cell antigen-like-containing domain protein 1	4.08E+05	1.60E+05	3.28E+04	0.00E+00	8.51E+04	1.13E+05
LLGL1	A0A087WW77	Lethal(2) giant larvae protein homolog 1	6.11E+05	2.25E+05	2.35E+05	3.11E+05	2.70E+04	1.27E+05
LMCD1	Q9NZU5-2	LIM and cysteine-rich domains protein 1	7.22E+04	0.00E+00	0.00E+00	1.52E+05	0.00E+00	6.49E+04
LMNA	P02545	Prelamin-A/C;Lamin-A/C	3.83E+07	3.00E+07	1.85E+07	8.65E+06	7.85E+06	9.75E+06
LMNB1	P20700	Lamin-B1	3.65E+06	2.73E+06	1.27E+06	3.33E+05	4.25E+05	3.43E+05
LMNB2	Q03252	Lamin-B2	1.23E+07	8.76E+06	6.16E+06	1.45E+06	8.41E+05	7.43E+05
LONP1	K7EJE8	Lon protease homolog, mitochondrial	3.91E+05	1.85E+05	2.69E+05	1.43E+06	3.53E+05	9.52E+05
LPP	A0A087WZF1	Lipoma-preferred partner	1.13E+05	3.02E+04	3.80E+04	3.08E+05	3.83E+04	1.75E+05
LRPPRC	P42704	Leucine-rich PPR motif-containing protein, mitochondrial	1.31E+06	1.47E+06	2.35E+06	4.82E+05	1.58E+05	5.05E+05
LRRC47	Q8N1G4	Leucine-rich repeat-containing protein 47	2.37E+05	1.17E+05	8.84E+04	1.10E+05	1.06E+05	1.36E+05
LRSAM1	Q6UWE0	E3 ubiquitin-protein ligase LRSAM1	3.45E+04	1.79E+04	0.00E+00	2.93E+04	0.00E+00	1.76E+04
LTA4H	P09960-2	Leukotriene A-4 hydrolase	2.57E+05	1.49E+05	1.39E+05	0.00E+00	2.78E+04	0.00E+00
LTN1	S4R3T2	E3 ubiquitin-protein ligase listerin	1.42E+04	0.00E+00	1.34E+04	6.72E+03	0.00E+00	0.00E+00
LUC7L	B8ZZ10	Putative RNA-binding protein Luc7-like 1	0.00E+00	1.73E+03	0.00E+00	5.54E+04	1.21E+05	1.92E+05
LUC7L2	Q9Y383	Putative RNA-binding protein Luc7-like 2	6.27E+05	2.24E+05	3.06E+05	1.01E+06	9.53E+05	2.42E+06
LUC7L3	O95232	Luc7-like protein 3	9.94E+04	4.57E+04	5.94E+03	3.07E+05	1.35E+05	2.48E+05
LUZP1	Q86V48-2	Leucine zipper protein 1	0.00E+00	2.12E+04	2.17E+04	1.82E+05	1.55E+05	1.78E+05
MACF1	H3BPE1	Microtubule-actin cross-linking factor 1, isoforms 1/2/3/5	7.43E+06	3.48E+06	1.04E+06	3.04E+06	2.91E+05	4.61E+05
MAD1L1	Q9Y6D9	Mitotic spindle assembly checkpoint protein MAD1	2.42E+04	0.00E+00	0.00E+00	8.50E+04	6.85E+04	2.26E+04
MAGOH	P61326	Protein mago nashi homolog;Protein mago nashi homolog 2	1.16E+05	1.44E+05	0.00E+00	1.60E+04	0.00E+00	7.45E+04

MAP1A	E9PGC8	Microtubule-associated protein 1A;MAP1A heavy chain;MAP1 light chain LC2	4.78E+06	2.38E+06	2.81E+06	5.51E+05	3.56E+05	7.10E+05
MAP1B	P46821	Microtubule-associated protein 1B;MAP1B heavy chain;MAP1 light chain LC1	1.96E+07	1.28E+07	1.11E+07	5.24E+06	4.55E+06	5.98E+06
MAP1S	Q66K74-2	Microtubule-associated protein 1S;MAP1S heavy chain;MAP1S light chain	4.00E+04	0.00E+00	8.15E+04	0.00E+00	3.68E+03	0.00E+00
MAP2	P11137-3	Microtubule-associated protein 2	1.18E+06	8.16E+05	4.87E+05	2.85E+05	1.00E+06	1.49E+06
MAP2K1	Q02750	Dual specificity mitogen-activated protein kinase kinase 1	9.87E+05	9.99E+04	7.84E+05	4.52E+05	9.48E+05	5.94E+05
MAP2K2	P36507	Dual specificity mitogen-activated protein kinase kinase 2	2.70E+05	2.14E+04	3.89E+05	2.29E+05	3.77E+05	2.09E+05
MAP2K4	P45985	Dual specificity mitogen-activated protein kinase kinase 4	3.28E+04	3.33E+04	2.47E+05	3.43E+04	1.33E+04	0.00E+00
MAP4	E7EVA0	Microtubule-associated protein;Microtubule-associated protein 4	3.70E+05	2.42E+05	2.92E+05	8.61E+04	4.46E+03	1.62E+05
MAP7D1	Q3KQU3-2	MAP7 domain-containing protein 1	6.90E+04	0.00E+00	4.23E+03	5.30E+04	1.44E+05	5.32E+04
MAPK10	A0A286YFD7	Mitogen-activated protein kinase 10;Mitogen-activated protein kinase 8;Mitogen-activated protein kinase 9	4.12E+04	2.27E+04	7.22E+04	1.30E+04	0.00E+00	4.07E+04
MAPRE1	Q15691	Microtubule-associated protein RP/EB family member 1	1.70E+05	1.11E+05	1.06E+05	7.50E+04	5.91E+04	1.59E+05
MAPRE2	Q15555-4	Microtubule-associated protein RP/EB family member 2	1.27E+06	3.82E+05	5.81E+05	7.47E+05	4.01E+05	3.22E+05
MAPRE3	C9JB30	Microtubule-associated protein RP/EB family member 3	4.82E+05	6.20E+04	1.40E+05	1.03E+04	0.00E+00	0.00E+00
MARCKS	P29966	Myristoylated alanine-rich C-kinase substrate	6.45E+05	1.01E+06	1.65E+06	0.00E+00	1.79E+04	1.71E+04
MARK2	Q7KZI7	Serine/threonine-protein kinase MARK2;MAP/microtubule affinity-regulating kinase 3	0.00E+00	5.37E+04	8.78E+04	7.39E+04	5.72E+04	5.89E+04
MARS	P56192	Methionine--tRNA ligase, cytoplasmic	5.00E+05	9.52E+04	3.76E+05	1.06E+05	9.97E+03	6.90E+04
MAT2A	P31153	S-adenosylmethionine synthase isoform type-2	1.95E+05	1.54E+05	7.35E+04	8.86E+04	1.29E+05	4.00E+04
MATR3	P43243	Matrin-3	8.27E+06	3.55E+06	5.67E+06	1.38E+07	1.11E+07	1.38E+07
MCAM	P43121	Cell surface glycoprotein MUC18	7.48E+05	4.26E+05	5.07E+05	7.66E+03	1.00E+04	2.35E+04
MCC	D6REY2	Colorectal mutant cancer protein	0.00E+00	1.03E+04	0.00E+00	0.00E+00	1.21E+04	0.00E+00
MCCC1	Q96RQ3	Methylcrotonoyl-CoA carboxylase subunit alpha, mitochondrial	1.88E+05	1.16E+05	3.62E+05	9.74E+04	0.00E+00	1.37E+05
MCCC2	Q9HCC0-2	Methylcrotonoyl-CoA carboxylase beta chain, mitochondrial	3.43E+05	1.42E+05	2.87E+05	3.07E+04	0.00E+00	2.75E+03
MDH1	P40925	Malate dehydrogenase, cytoplasmic;Malate dehydrogenase	1.44E+07	4.01E+06	9.39E+06	4.37E+04	7.39E+04	9.96E+03
MDH2	P40926	Malate dehydrogenase, mitochondrial;Malate	2.38E+07	1.47E+07	1.51E+07	3.50E+04	1.14E+04	0.00E+00

		dehydrogenase						
MECP2	P51608	Methyl-CpG-binding protein 2	1.85E+06	9.30E+05	2.49E+05	1.14E+07	1.75E+07	1.46E+07
MFAP1	P55081	Microfibrillar-associated protein 1	8.36E+04	8.19E+04	0.00E+00	3.54E+04	0.00E+00	1.03E+03
MFF	A0A0A0MS29	Mitochondrial fission factor	4.26E+04	0.00E+00	0.00E+00	0.00E+00	1.60E+03	7.88E+03
MFN2	O95140	Mitofusin-2	1.95E+05	8.13E+04	2.83E+05	1.17E+05	2.31E+05	4.24E+05
MGMT	P16455	Methylated-DNA--protein-cysteine methyltransferase	1.05E+05	0.00E+00	7.46E+04	7.68E+04	3.23E+04	4.62E+04
MGST3	O14880	Microsomal glutathione S-transferase 3	4.33E+06	2.18E+06	3.78E+06	2.38E+05	1.07E+04	1.20E+05
MIA3	Q5JRA6	Melanoma inhibitory activity protein 3	3.12E+05	9.13E+04	2.69E+05	1.33E+04	3.60E+04	3.03E+04
MIF	P14174	Macrophage migration inhibitory factor	5.90E+05	4.93E+05	1.29E+05	2.20E+04	0.00E+00	8.57E+02
MINK1	Q8N4C8-5	Misshapen-like kinase 1	0.00E+00	2.43E+04	0.00E+00	1.69E+05	1.09E+05	1.42E+05
MMRN2	Q9H8L6	Multimerin-2	3.39E+05	1.60E+05	1.76E+05	3.26E+05	3.96E+05	5.04E+05
MOGS	Q13724-2	Mannosyl-oligosaccharide glucosidase	4.53E+05	1.46E+05	3.46E+05	9.19E+03	0.00E+00	1.50E+04
MORF4L1	H0YMJ0	Mortality factor 4-like protein 1	2.44E+04	4.91E+03	1.24E+04	7.00E+04	1.15E+05	9.13E+04
MPHOSPH10	O00566	U3 small nucleolar ribonucleoprotein protein MPP10	0.00E+00	1.43E+04	0.00E+00	0.00E+00	8.64E+04	1.03E+05
MPHOSPH8	Q99549	M-phase phosphoprotein 8	0.00E+00	4.67E+04	0.00E+00	1.45E+05	1.85E+05	1.42E+05
MPP1	Q00013-2	55 kDa erythrocyte membrane protein	1.28E+05	0.00E+00	1.31E+05	6.13E+04	6.06E+04	1.39E+05
MPRIP	Q6WCQ1	Myosin phosphatase Rho-interacting protein	1.95E+05	1.66E+05	2.13E+04	4.00E+05	3.91E+05	4.75E+05
MRE11A	F8W7U8	Double-strand break repair protein MRE11A	2.47E+05	1.89E+04	7.30E+04	4.56E+05	2.81E+05	1.64E+05
MRPS22	G5E9W7	28S ribosomal protein S22, mitochondrial	0.00E+00	1.81E+04	0.00E+00	3.55E+03	0.00E+00	5.88E+04
MSH2	P43246-2	DNA mismatch repair protein Msh2	3.16E+05	1.55E+04	6.81E+04	6.67E+05	1.04E+06	1.02E+06
MSH6	P52701	DNA mismatch repair protein Msh6	2.44E+05	4.45E+04	0.00E+00	1.60E+06	7.70E+05	1.17E+06
MSI2	B4DHE8	RNA-binding protein Musashi homolog 2	9.63E+04	1.31E+05	0.00E+00	1.41E+05	1.16E+05	1.46E+05
MSN	P26038	Moesin	2.41E+05	1.67E+05	1.17E+05	4.18E+04	0.00E+00	3.82E+04
MTA1	E7ESY4	Metastasis-associated protein MTA1	4.20E+04	0.00E+00	0.00E+00	2.94E+05	1.70E+05	2.36E+05
MTA2	O94776	Metastasis-associated protein MTA2	1.59E+05	0.00E+00	2.34E+04	3.64E+05	8.37E+04	5.90E+05
MTCH1	H0Y8C3	Mitochondrial carrier homolog 1	1.47E+05	1.59E+05	2.32E+05	0.00E+00	9.42E+03	0.00E+00
MTCH2	Q9Y6C9	Mitochondrial carrier homolog 2	2.38E+06	1.19E+06	1.73E+06	0.00E+00	2.23E+04	0.00E+00
MTDH	Q86UE4	Protein LYRIC	1.19E+06	9.02E+05	4.02E+05	3.22E+04	0.00E+00	5.22E+04
MTFR1L	E9PPF9	Mitochondrial fission regulator 1-like	2.96E+04	2.02E+04	0.00E+00	0.00E+00	5.48E+03	0.00E+00
MTHFD1	P11586	C-1-tetrahydrofolate synthase, cytoplasmic;Methylenetetrahydrofolate dehydrogenase;Methenyltetrahydrofolate cyclohydrolase;Formyltetrahydrofolate synthetase;C-1-tetrahydrofolate synthase, cytoplasmic, N-terminally processed	8.98E+05	4.98E+05	9.52E+05	7.39E+05	4.17E+05	8.02E+05

MTMR2	C9JEX3	Myotubularin-related protein 2	1.46E+05	0.00E+00	1.12E+05	0.00E+00	9.21E+03	1.28E+04
MUT	P22033	Methylmalonyl-CoA mutase, mitochondrial	1.90E+05	1.08E+05	2.03E+05	1.25E+04	0.00E+00	0.00E+00
MVP	Q14764	Major vault protein	1.52E+06	1.08E+06	6.80E+05	0.00E+00	1.68E+04	6.68E+03
MX1	P20591	Interferon-induced GTP-binding protein Mx1;Interferon-induced GTP-binding protein Mx1, N-terminally processed	3.16E+05	0.00E+00	1.53E+05	7.39E+05	1.33E+05	1.87E+05
MYADM	C9JJV6	Myeloid-associated differentiation marker	9.19E+05	2.80E+05	3.94E+05	9.37E+05	7.74E+05	7.77E+05
MYBBP1A	Q9BQG0	Myb-binding protein 1A	1.05E+05	1.64E+04	8.16E+03	5.83E+05	2.77E+05	4.32E+05
MYCBP2	O75592-2	E3 ubiquitin-protein ligase MYCBP2	9.92E+04	2.72E+04	4.90E+04	5.27E+04	3.92E+04	3.91E+04
MYH10	P35580	Myosin-10	4.34E+06	3.09E+06	2.70E+06	2.84E+06	3.10E+06	5.20E+06
MYH14	Q7Z406	Myosin-14	4.49E+05	2.66E+05	2.03E+05	6.93E+05	2.83E+05	5.45E+05
MYH9	P35579	Myosin-9	2.47E+07	1.97E+07	1.64E+07	3.73E+07	3.79E+07	3.24E+07
MYL1	P05976-2	Myosin light chain 1/3, skeletal muscle isoform;Myosin light chain 3	3.36E+05	0.00E+00	1.43E+05	0.00E+00	3.39E+05	0.00E+00
MYL12A	J3QRS3	Myosin regulatory light chain 12A;Myosin regulatory light chain 12B	8.54E+06	5.03E+06	9.29E+06	1.12E+07	8.99E+06	7.88E+06
MYL6	F8VPF3	Myosin light polypeptide 6	4.70E+06	1.89E+06	1.93E+06	6.71E+05	1.71E+06	1.07E+06
MYL9	P24844	Myosin regulatory light polypeptide 9	9.95E+05	5.41E+05	7.23E+05	4.73E+06	2.32E+06	1.53E+06
MYLK	Q15746-11	Myosin light chain kinase, smooth muscle;Myosin light chain kinase, smooth muscle, deglutamylated form	7.37E+05	3.36E+05	4.24E+05	9.16E+05	8.47E+05	4.32E+05
MYO18A	A0A0D9SFK2	Unconventional myosin-XVIIIa	5.25E+05	4.46E+05	4.36E+05	1.52E+05	9.98E+04	1.91E+05
MYO1B	E9PDF6	Unconventional myosin-Ib	5.06E+05	4.25E+05	3.85E+05	1.55E+06	1.09E+06	1.17E+06
MYO1C	F5H6E2	Unconventional myosin-Ic	1.34E+06	1.11E+06	1.87E+05	2.00E+06	2.05E+06	1.27E+06
MYO1D	O94832	Unconventional myosin-Id	1.03E+06	3.77E+05	5.08E+05	5.98E+05	3.70E+05	1.80E+05
MYO1E	Q12965	Unconventional myosin-Ie	1.15E+05	1.54E+04	0.00E+00	6.18E+04	0.00E+00	5.48E+04
MYO5A	A0A087WY00	Unconventional myosin-Va	1.67E+06	1.36E+06	1.67E+06	1.28E+06	9.98E+05	1.16E+06
MYO6	A0A0A0MRM8	Unconventional myosin-VI	3.00E+05	2.98E+05	1.56E+05	2.45E+05	3.85E+05	1.64E+05
NACA	F8VZJ2	Nascent polypeptide-associated complex subunit alpha;Nascent polypeptide-associated complex subunit alpha, muscle-specific form	6.44E+05	4.26E+05	1.25E+05	3.28E+05	5.48E+04	7.97E+05
NAGK	H7C3G9	N-acetyl-D-glucosamine kinase	3.37E+04	0.00E+00	1.04E+04	6.88E+03	0.00E+00	0.00E+00
NAMPT	P43490	Nicotinamide phosphoribosyltransferase	5.68E+05	8.92E+03	1.68E+05	1.10E+05	6.00E+04	3.21E+05
NAP1L1	P55209	Nucleosome assembly protein 1-like 1	9.54E+05	9.31E+05	5.02E+05	4.46E+05	3.13E+05	5.24E+05
NAPA	P54920	Alpha-soluble NSF attachment protein	2.69E+06	2.02E+06	1.15E+06	2.92E+04	0.00E+00	4.46E+03
NAPG	Q99747	Gamma-soluble NSF attachment protein	2.23E+06	1.38E+06	8.43E+05	3.65E+03	6.04E+04	4.84E+04
NAPRT	G5E977	Nicotinate phosphoribosyltransferase	2.75E+04	0.00E+00	0.00E+00	0.00E+00	7.96E+04	0.00E+00

NAT10	Q9H0A0	N-acetyltransferase 10	1.68E+05	1.08E+04	2.00E+04	1.24E+05	7.20E+04	2.38E+05
NBAS	H0Y5G7	Neuroblastoma-amplified sequence	1.10E+04	3.52E+03	0.00E+00	2.80E+04	2.36E+03	0.00E+00
NBN	O60934	Nibrin	0.00E+00	1.75E+04	0.00E+00	1.68E+05	7.61E+04	1.34E+05
NCBP1	Q09161	Nuclear cap-binding protein subunit 1	1.75E+05	6.04E+04	9.12E+03	1.66E+05	2.29E+04	7.25E+04
NCL	P19338	Nucleolin	1.26E+06	2.42E+06	1.34E+06	3.51E+06	1.84E+06	1.98E+06
NCOA7	Q8NI08-7	Nuclear receptor coactivator 7	1.16E+04	0.00E+00	5.74E+04	1.72E+04	0.00E+00	2.00E+04
NDUFA10	A0A087WXC5	NADH dehydrogenase [ubiquinone] 1 alpha subcomplex subunit 10, mitochondrial	1.81E+06	7.76E+05	1.11E+06	5.95E+04	7.01E+04	1.03E+05
NDUFA12	Q9UI09	NADH dehydrogenase [ubiquinone] 1 alpha subcomplex subunit 12	1.82E+06	1.21E+06	1.06E+06	0.00E+00	1.00E+04	6.66E+04
NDUFA4	O00483	Cytochrome c oxidase subunit NDUFA4	1.21E+06	1.29E+06	1.61E+05	1.76E+05	0.00E+00	0.00E+00
NDUFA9	Q16795	NADH dehydrogenase [ubiquinone] 1 alpha subcomplex subunit 9, mitochondrial	4.47E+06	2.56E+06	4.16E+06	0.00E+00	1.80E+04	1.30E+04
NDUFB10	O96000	NADH dehydrogenase [ubiquinone] 1 beta subcomplex subunit 10	1.95E+05	5.12E+05	2.04E+05	3.33E+04	1.39E+04	1.28E+04
NDUFS1	P28331-4	NADH-ubiquinone oxidoreductase 75 kDa subunit, mitochondrial	1.11E+07	6.90E+06	1.16E+07	1.02E+06	6.62E+05	8.66E+05
NDUFS2	O75306-2	NADH dehydrogenase [ubiquinone] iron-sulfur protein 2, mitochondrial	5.21E+06	3.00E+06	3.69E+06	0.00E+00	4.10E+04	5.19E+04
NDUFS3	O75489	NADH dehydrogenase [ubiquinone] iron-sulfur protein 3, mitochondrial	3.71E+06	2.04E+06	5.55E+06	0.00E+00	7.54E+04	1.52E+05
NDUFS5	O43920	NADH dehydrogenase [ubiquinone] iron-sulfur protein 5	9.77E+05	4.59E+05	1.50E+05	1.11E+04	0.00E+00	0.00E+00
NDUFS7	O75251	NADH dehydrogenase [ubiquinone] iron-sulfur protein 7, mitochondrial	8.90E+05	5.72E+05	2.14E+05	2.15E+04	1.90E+04	8.01E+04
NDUFS8	E9PPW7	NADH dehydrogenase [ubiquinone] iron-sulfur protein 8, mitochondrial	9.05E+05	8.46E+05	4.14E+05	2.24E+04	0.00E+00	1.75E+04
NDUFV1	G3V0I5	NADH dehydrogenase [ubiquinone] flavoprotein 1, mitochondrial	2.18E+06	1.40E+06	1.54E+06	4.20E+04	2.31E+04	1.68E+04
NEDD4L	A0A1B0GVY1	E3 ubiquitin-protein ligase NEDD4-like	1.72E+04	0.00E+00	0.00E+00	1.05E+05	0.00E+00	1.04E+05
NEFL	P07196	Neurofilament light polypeptide	3.21E+07	2.66E+07	6.68E+06	8.16E+06	2.89E+07	5.61E+06
NEK9	Q8TD19	Serine/threonine-protein kinase Nek9	1.53E+04	0.00E+00	0.00E+00	3.13E+04	5.79E+04	9.94E+04
NELFA	A0A0C4DFX9	Negative elongation factor A	8.92E+04	0.00E+00	0.00E+00	6.64E+04	3.81E+04	0.00E+00
NELFE	P18615	Negative elongation factor E	1.01E+04	0.00E+00	0.00E+00	1.12E+05	6.47E+04	2.99E+04
NES	P48681	Nestin	9.61E+05	1.45E+06	7.17E+05	9.19E+05	3.53E+05	1.35E+06
NFS1	Q9Y697-2	Cysteine desulfurase, mitochondrial	1.68E+05	8.55E+04	2.21E+05	2.19E+04	1.28E+04	3.27E+04
NFXL1	Q6ZNB6-2	NF-X1-type zinc finger protein NFXL1	3.18E+04	0.00E+00	0.00E+00	4.57E+04	1.25E+04	4.79E+04
NHP2	J3QSY4	H/ACA ribonucleoprotein complex subunit 2	1.34E+05	8.86E+04	0.00E+00	2.45E+05	1.60E+05	3.06E+05

NHP2L1	B1AHD1	NHP2-like protein 1;NHP2-like protein 1, N-terminally processed	5.11E+05	6.99E+05	4.23E+05	3.43E+05	7.11E+05	4.24E+05
NID1	P14543-2	Nidogen-1	5.60E+05	2.84E+05	2.08E+05	1.32E+07	1.63E+07	1.06E+07
NID2	Q14112-2	Nidogen-2	3.07E+05	1.76E+05	4.86E+04	9.49E+06	8.96E+06	7.51E+06
NIPBL	Q6KC79-2	Nipped-B-like protein	1.83E+04	0.00E+00	0.00E+00	2.56E+05	1.18E+05	2.80E+05
NIPSNAP1	Q9BPW8	Protein NipSnap homolog 1	8.25E+05	7.74E+05	6.53E+05	1.02E+04	0.00E+00	0.00E+00
NMRAL1	Q9HBL8	NmrA-like family domain-containing protein 1	4.30E+04	0.00E+00	2.70E+04	3.38E+04	8.97E+04	4.96E+04
NMT1	P30419-2	Glycylpeptide N-tetradecanoyltransferase 1;Glycylpeptide N-tetradecanoyltransferase;Glycylpeptide N-tetradecanoyltransferase 2	3.39E+04	3.21E+04	3.94E+04	7.76E+03	0.00E+00	0.00E+00
NOC2L	Q9Y3T9	Nucleolar complex protein 2 homolog	0.00E+00	3.73E+04	0.00E+00	0.00E+00	6.19E+04	1.26E+05
NOL4L	A0A087X0N3		1.46E+03	0.00E+00	0.00E+00	5.50E+04	2.88E+04	3.34E+04
NOL6	A0A0A0MRW6	Nucleolar protein 6	4.82E+04	0.00E+00	0.00E+00	2.43E+04	1.07E+05	1.56E+05
NOL9	Q5SY16	Polynucleotide 5-hydroxyl-kinase NOL9	1.81E+05	2.12E+04	9.22E+03	1.19E+05	3.20E+05	2.71E+05
NONO	Q15233	Non-POU domain-containing octamer-binding protein	8.82E+05	5.62E+05	1.11E+05	1.52E+06	1.36E+06	1.03E+06
NOP14	P78316	Nucleolar protein 14	0.00E+00	1.04E+04	0.00E+00	5.56E+04	4.29E+04	2.70E+04
NOP2	P46087-2	Probable 28S rRNA (cytosine(4447)-C(5))-methyltransferase	8.20E+04	6.08E+04	0.00E+00	1.96E+05	1.88E+05	2.69E+05
NOP56	O00567	Nucleolar protein 56	1.65E+06	6.59E+05	7.05E+05	4.37E+06	4.98E+06	5.76E+06
NOP58	Q9Y2X3	Nucleolar protein 58	1.23E+06	8.36E+05	4.84E+05	3.80E+06	5.38E+06	7.17E+06
NOP9	Q86U38-2	Nucleolar protein 9	3.94E+04	0.00E+00	0.00E+00	4.48E+04	3.49E+04	1.30E+05
NOS3	P29474	Nitric oxide synthase, endothelial	1.10E+05	5.25E+04	9.34E+04	1.08E+05	5.53E+04	0.00E+00
NOSIP	M0R3B2	Nitric oxide synthase-interacting protein	2.03E+04	0.00E+00	0.00E+00	1.57E+05	5.37E+04	2.16E+05
NOVA2	Q9UNW9	RNA-binding protein Nova-2	4.40E+05	2.11E+05	4.70E+05	4.66E+05	8.31E+05	1.29E+06
NPLOC4	Q8TAT6	Nuclear protein localization protein 4 homolog	2.39E+04	8.16E+04	0.00E+00	4.34E+04	0.00E+00	4.66E+04
NPM1	P06748-2	Nucleophosmin	2.52E+06	8.98E+05	2.03E+06	3.88E+06	3.19E+06	3.65E+06
NR3C1	P04150-9	Glucocorticoid receptor	1.45E+05	2.05E+04	2.12E+03	4.92E+05	5.34E+05	5.69E+05
NSF	I3L0N3	Vesicle-fusing ATPase	8.95E+06	5.64E+06	9.85E+06	7.09E+06	1.10E+07	9.11E+06
NSFL1C	Q9UNZ2	NSFL1 cofactor p47	1.42E+05	1.74E+05	1.36E+05	1.84E+05	1.41E+05	2.60E+05
NSUN2	Q08J23-2	tRNA (cytosine(34)-C(5))-methyltransferase	1.47E+05	9.26E+04	1.20E+05	5.55E+05	2.70E+05	4.80E+05
NT5E	P21589-2	5-nucleotidase	1.25E+05	1.56E+05	6.51E+04	0.00E+00	4.70E+04	0.00E+00
NUCKS1	Q9H1E3	Nuclear ubiquitous casein and cyclin-dependent kinase substrate 1	2.57E+04	0.00E+00	3.57E+04	9.73E+04	2.55E+05	2.87E+05
NUDC	Q9Y266	Nuclear migration protein nudC	1.60E+05	1.55E+05	1.40E+05	2.13E+03	0.00E+00	0.00E+00
NUDCD2	Q8WVJ2	NudC domain-containing protein 2	1.39E+05	0.00E+00	8.56E+03	5.16E+04	3.58E+04	5.32E+04

NUDT5	A6NFX8	ADP-sugar pyrophosphatase	1.33E+05	6.55E+04	0.00E+00	1.32E+03	0.00E+00	4.47E+04
NUDT9	H7C386	ADP-ribose pyrophosphatase, mitochondrial	3.27E+04	0.00E+00	2.96E+04	2.73E+04	0.00E+00	0.00E+00
NUMA1	Q14980-2	Nuclear mitotic apparatus protein 1	4.58E+06	2.68E+06	6.60E+05	1.17E+07	1.39E+07	1.26E+07
NUMBL	M0QYC2	Numb-like protein	7.89E+03	5.80E+04	1.77E+05	0.00E+00	7.10E+03	4.60E+04
NUP107	P57740	Nuclear pore complex protein Nup107	1.82E+05	8.77E+04	2.13E+05	2.94E+04	0.00E+00	0.00E+00
NUP133	Q8WUM0	Nuclear pore complex protein Nup133	5.64E+05	2.37E+05	2.03E+05	1.26E+05	3.78E+04	9.21E+03
NUP153	P49790-2	Nuclear pore complex protein Nup153	5.77E+04	1.36E+04	0.00E+00	4.22E+04	0.00E+00	1.08E+05
NUP155	O75694	Nuclear pore complex protein Nup155	1.01E+06	6.01E+05	4.50E+05	3.11E+04	1.88E+04	1.53E+04
NUP205	Q92621	Nuclear pore complex protein Nup205	5.54E+05	2.48E+05	3.03E+05	0.00E+00	2.56E+04	2.09E+04
NUP214	P35658-2	Nuclear pore complex protein Nup214	1.68E+05	7.68E+04	2.10E+04	8.47E+04	3.53E+04	4.95E+04
NUP54	Q7Z3B4	Nucleoporin p54	2.20E+05	2.93E+05	3.07E+05	2.56E+04	1.64E+04	5.08E+04
NUP88	J3KMX1	Nuclear pore complex protein Nup88	1.88E+05	1.42E+05	0.00E+00	7.12E+04	5.21E+04	3.35E+04
NUP93	H3BVG0	Nuclear pore complex protein Nup93	6.16E+05	5.19E+05	6.03E+05	5.16E+04	7.09E+03	0.00E+00
NUP98	P52948-6	Nuclear pore complex protein Nup98-Nup96;Nuclear pore complex protein Nup98;Nuclear pore complex protein Nup96	3.90E+05	1.32E+05	2.45E+05	6.23E+04	0.00E+00	2.40E+04
NXF1	E9PIN3	Nuclear RNA export factor 1	3.80E+04	6.30E+04	3.35E+04	0.00E+00	4.89E+04	6.83E+04
OAS2	A0A087X0V5	2-5-oligoadenylate synthase 2	2.55E+04	5.72E+04	0.00E+00	0.00E+00	6.28E+04	1.63E+05
OAT	P04181	Ornithine aminotransferase, mitochondrial;Ornithine aminotransferase, hepatic form;Ornithine aminotransferase, renal form	4.55E+05	8.63E+04	3.17E+05	6.09E+04	0.00E+00	5.02E+03
OCLN	A0A0G2JMZ8	Occludin	2.17E+05	0.00E+00	1.32E+04	6.17E+05	1.41E+05	3.79E+05
OGDH	Q02218	2-oxoglutarate dehydrogenase, mitochondrial	2.69E+06	1.85E+06	4.23E+06	4.22E+04	1.32E+04	0.00E+00
OGDHL	Q9ULD0	2-oxoglutarate dehydrogenase-like, mitochondrial	1.05E+06	3.72E+05	7.59E+05	6.22E+04	0.00E+00	0.00E+00
OGFR	Q9NZT2-2	Opioid growth factor receptor	3.58E+04	2.36E+04	2.92E+04	7.47E+04	6.49E+04	8.72E+04
OGT	O15294-3	UDP-N-acetylglucosamine--peptide N-acetylglucosaminyltransferase 110 kDa subunit	3.55E+05	9.33E+04	2.35E+05	4.53E+05	8.04E+05	1.35E+06
OLA1	J3KQ32	Obg-like ATPase 1	3.07E+05	8.21E+04	1.90E+05	2.46E+04	9.07E+03	1.14E+04
OPA1	O60313	Dynamin-like 120 kDa protein, mitochondrial;Dynamin-like 120 kDa protein, form S1	4.38E+06	2.00E+06	3.00E+06	6.00E+04	7.08E+04	1.01E+05
OPTN	Q96CV9-2	Optineurin	2.34E+04	9.13E+04	2.62E+04	3.48E+05	3.77E+05	3.47E+05
OSBPL8	Q9BZF1-3	Oxysterol-binding protein-related protein 8;Oxysterol-binding protein	1.24E+05	6.37E+04	6.71E+04	4.14E+05	9.78E+04	1.32E+05
OSTF1	Q92882	Osteoclast-stimulating factor 1	1.29E+05	3.25E+04	7.93E+04	6.02E+04	0.00E+00	0.00E+00
OTUB1	F5GYN4	Ubiquitin thioesterase OTUB1	1.64E+06	7.23E+05	6.73E+05	3.66E+05	4.73E+05	2.56E+05
OXCT1	P55809	Succinyl-CoA:3-ketoacid coenzyme A transferase 1, mitochondrial	2.11E+06	1.17E+06	1.52E+06	5.92E+04	0.00E+00	4.32E+04

OXSR1	O95747	Serine/threonine-protein kinase OSR1	1.25E+05	6.91E+04	7.82E+04	7.71E+04	1.24E+05	2.02E+05
P4HB	P07237	Protein disulfide-isomerase	3.60E+06	1.59E+06	1.92E+06	0.00E+00	1.15E+04	0.00E+00
PA2G4	Q9UQ80-2	Proliferation-associated protein 2G4	4.76E+04	0.00E+00	4.91E+04	1.66E+04	0.00E+00	1.83E+04
PABPC1	P11940	Polyadenylate-binding protein 1;Polyadenylate-binding protein;Polyadenylate-binding protein 3	8.52E+05	4.39E+05	5.85E+05	3.58E+05	1.88E+05	4.73E+05
PABPN1	B4DEH8	Polyadenylate-binding protein 2	1.51E+05	9.44E+04	4.40E+04	1.57E+04	3.69E+04	4.83E+04
PACS1	Q6VY07	Phosphofurin acidic cluster sorting protein 1	9.41E+04	7.07E+04	0.00E+00	1.04E+05	1.35E+05	1.56E+05
PACSIN2	Q9UNF0-2	Protein kinase C and casein kinase substrate in neurons protein 2	0.00E+00	3.89E+03	1.51E+04	6.89E+03	0.00E+00	0.00E+00
PAF1	Q8N7H5-3	RNA polymerase II-associated factor 1 homolog	6.83E+04	4.30E+04	8.42E+04	4.80E+05	4.95E+05	4.98E+05
PAFAH1B1	P43034	Platelet-activating factor acetylhydrolase IB subunit alpha	8.78E+05	3.20E+05	1.89E+05	1.46E+05	4.60E+04	1.32E+05
PAK1	E9PM17	Non-specific serine/threonine protein kinase;Serine/threonine-protein kinase PAK 1	7.31E+04	3.60E+04	0.00E+00	6.58E+04	0.00E+00	4.35E+04
PALD1	Q9ULE6	Paladin	5.98E+03	0.00E+00	0.00E+00	5.25E+04	7.03E+04	8.38E+04
PALLD	Q8WX93-7	Palladin	2.73E+04	0.00E+00	0.00E+00	1.71E+04	0.00E+00	0.00E+00
PALMD	S4R313	Palmdelphin	2.53E+04	0.00E+00	2.08E+04	6.95E+03	0.00E+00	4.25E+04
PANK4	A0A0G2JR38	Pantothenate kinase 4	5.42E+04	7.01E+04	4.70E+04	7.11E+04	6.16E+04	6.94E+04
PARP1	P09874	Poly [ADP-ribose] polymerase 1	2.10E+06	1.00E+06	2.55E+05	1.61E+07	4.42E+06	8.85E+06
PARVA	J3KNQ4	Alpha-parvin	5.34E+04	7.65E+04	5.58E+04	8.33E+04	1.56E+04	0.00E+00
PARVB	B0QYP8	Beta-parvin	2.84E+04	1.12E+04	1.26E+04	4.15E+04	9.35E+04	1.36E+05
PBRM1	Q86U86-5	Protein polybromo-1	1.67E+04	2.21E+04	0.00E+00	1.59E+05	3.46E+05	4.25E+05
PC	P11498	Pyruvate carboxylase, mitochondrial	1.05E+06	3.63E+05	4.92E+05	2.86E+06	6.21E+05	2.32E+06
PCBP1	Q15365	Poly(rC)-binding protein 1	1.60E+06	1.86E+06	1.33E+06	3.72E+06	3.15E+06	3.56E+06
PCBP2	Q15366-3	Poly(rC)-binding protein 2	2.12E+05	2.72E+05	0.00E+00	1.14E+06	8.35E+05	5.99E+05
PCBP4	C9JZY3	Poly(rC)-binding protein 4	2.90E+04	4.57E+04	0.00E+00	0.00E+00	7.04E+04	4.24E+04
PCDH1	Q08174	Protocadherin-1	7.03E+04	7.76E+04	0.00E+00	3.58E+04	4.30E+04	1.52E+04
PCIF1	A0A087WWZ2	Phosphorylated CTD-interacting factor 1	4.76E+04	0.00E+00	0.00E+00	2.25E+05	7.86E+04	0.00E+00
PCNA	P12004	Proliferating cell nuclear antigen	6.15E+04	0.00E+00	0.00E+00	1.83E+03	0.00E+00	0.00E+00
PCSK1N	Q9UHG2	ProSAAS;KEP;Big SAAS;Little SAAS;Big PEN-LEN;PEN;Little LEN;Big LEN	7.40E+05	4.51E+05	1.20E+06	1.59E+04	3.58E+04	1.80E+06
PCYT1A	C9JEJ2	Choline-phosphate cytidylyltransferase A	5.24E+05	1.78E+05	1.03E+05	2.80E+05	2.83E+05	2.68E+05
PDCD11	Q14690	Protein RRP5 homolog	5.42E+04	0.00E+00	0.00E+00	1.87E+05	1.82E+05	2.40E+05
PDCD6IP	Q8WUM4	Programmed cell death 6-interacting protein	8.10E+05	3.13E+05	4.24E+05	2.40E+05	5.84E+04	2.21E+05
PDDC1	Q8NB37	Parkinson disease 7 domain-containing protein 1	1.13E+03	1.69E+04	6.66E+04	1.05E+04	0.00E+00	0.00E+00
PDE2A	O00408-2	cGMP-dependent 3,5-cyclic phosphodiesterase	6.15E+05	3.12E+04	1.13E+05	6.56E+05	8.19E+04	7.94E+04
PDHA1	P08559	Pyruvate dehydrogenase E1 component subunit alpha,	2.30E+06	2.71E+06	1.06E+06	1.88E+04	0.00E+00	1.32E+05

		somatic form, mitochondrial						
PDIA3	P30101	Protein disulfide-isomerase A3	6.88E+06	3.18E+06	2.07E+06	1.32E+05	2.09E+04	0.00E+00
PDLIM2	Q96JY6	PDZ and LIM domain protein 2	4.99E+04	0.00E+00	6.09E+04	0.00E+00	6.75E+03	1.56E+04
PDLIM7	Q9NR12	PDZ and LIM domain protein 7	1.39E+05	0.00E+00	0.00E+00	5.13E+05	3.35E+05	3.30E+05
PDS5A	Q29RF7	Sister chromatid cohesion protein PDS5 homolog A	1.05E+05	0.00E+00	0.00E+00	6.44E+05	9.14E+04	1.62E+05
PDS5B	Q9NTI5-2	Sister chromatid cohesion protein PDS5 homolog B	5.22E+05	2.07E+05	2.89E+05	3.87E+05	1.80E+05	3.85E+05
PDXK	O00764	Pyridoxal kinase	7.03E+05	1.24E+05	6.39E+05	3.63E+05	2.15E+05	1.40E+05
PEA15	Q15121-2	Astrocytic phosphoprotein PEA-15	3.13E+05	3.57E+04	8.12E+04	7.58E+03	0.00E+00	0.00E+00
PEBP1	P30086	Phosphatidylethanolamine-binding protein 1;Hippocampal cholinergic neurostimulating peptide	1.33E+07	7.13E+06	6.86E+06	8.46E+04	8.83E+04	8.32E+03
PECAM1	A0A075B738	Platelet endothelial cell adhesion molecule	1.95E+05	2.44E+05	1.77E+05	3.37E+03	0.00E+00	0.00E+00
PELP1	C9JFV4	Proline-, glutamic acid- and leucine-rich protein 1	2.68E+05	2.66E+05	2.15E+05	3.67E+05	3.93E+05	4.88E+05
PES1	B3KXD6	Pescadillo homolog	0.00E+00	1.52E+04	1.40E+04	5.49E+04	1.17E+05	1.72E+05
PEX5	B4E0T2	Peroxisomal targeting signal 1 receptor	8.28E+04	7.35E+03	2.78E+04	1.00E+05	1.03E+05	9.15E+04
PFDN2	Q9UHV9	Prefoldin subunit 2	9.31E+04	4.79E+04	0.00E+00	1.12E+05	6.34E+03	3.71E+04
PFKL	P17858	ATP-dependent 6-phosphofructokinase, liver type	2.08E+06	4.21E+05	0.00E+00	1.69E+06	6.14E+05	1.03E+06
PFKM	P08237-3	ATP-dependent 6-phosphofructokinase, muscle type	1.73E+06	6.32E+05	1.21E+06	3.17E+05	3.39E+05	4.42E+05
PFKP	Q01813	ATP-dependent 6-phosphofructokinase, platelet type	4.11E+06	2.82E+06	6.82E+06	3.64E+06	4.09E+06	6.63E+06
PFN2	C9J712	Profilin;Profilin-2	1.80E+06	6.34E+05	6.28E+05	9.23E+05	3.30E+05	6.24E+05
PGAM1	P18669	Phosphoglycerate mutase 1	6.78E+06	4.05E+06	4.84E+06	1.60E+05	1.20E+05	7.82E+04
PGK1	P00558-2	Phosphoglycerate kinase 1	4.61E+06	2.36E+06	4.51E+06	1.99E+04	0.00E+00	0.00E+00
PGRMC1	O00264	Membrane-associated progesterone receptor component 1	3.98E+06	2.07E+06	1.43E+06	3.87E+04	2.05E+04	6.55E+04
PGRMC2	O15173-2	Membrane-associated progesterone receptor component 2	9.72E+05	1.17E+06	5.87E+05	2.73E+04	0.00E+00	0.00E+00
PHB	P35232	Prohibitin	2.49E+06	2.06E+06	8.91E+05	0.00E+00	7.53E+04	0.00E+00
PHB2	J3KPX7	Prohibitin-2	2.94E+06	1.99E+06	2.27E+06	0.00E+00	2.61E+04	0.00E+00
PHGDH	A0A286YF22	D-3-phosphoglycerate dehydrogenase	1.68E+06	5.50E+05	6.58E+05	5.17E+05	4.87E+05	1.21E+05
PHLDB1	Q86UU1-3	Pleckstrin homology-like domain family B member 1	0.00E+00	1.74E+04	0.00E+00	1.22E+05	1.94E+05	2.25E+05
PI4KA	P42356	Phosphatidylinositol 4-kinase alpha	3.72E+05	3.61E+04	9.65E+04	1.44E+05	4.54E+04	1.45E+05
PICALM	Q13492-4	Phosphatidylinositol-binding clathrin assembly protein	1.64E+05	3.51E+04	2.30E+05	1.37E+05	5.64E+04	1.20E+05
PIK3C3	A8MYT4	Phosphatidylinositol 3-kinase;Phosphatidylinositol 3-kinase catalytic subunit type 3	6.67E+04	0.00E+00	3.48E+03	4.39E+04	2.93E+04	6.45E+04
PIK3R4	Q99570	Phosphoinositide 3-kinase regulatory subunit 4	5.43E+04	0.00E+00	0.00E+00	0.00E+00	8.28E+03	0.00E+00
PIN1	Q13526	Peptidyl-prolyl cis-trans isomerase NIMA-interacting 1	1.07E+06	6.64E+05	8.24E+05	3.91E+05	3.63E+05	5.24E+05
PIP4K2A	P48426-2	Phosphatidylinositol 5-phosphate 4-kinase type-2 alpha	1.37E+06	9.31E+05	7.91E+05	1.41E+05	9.40E+04	8.28E+04

PIP4K2B	P78356	Phosphatidylinositol 5-phosphate 4-kinase type-2 beta	8.37E+05	5.51E+05	3.13E+05	5.88E+05	3.30E+05	4.69E+05
PIP4K2C	Q8TBX8-3	Phosphatidylinositol 5-phosphate 4-kinase type-2 gamma	1.05E+05	1.28E+05	1.14E+05	4.65E+04	0.00E+00	0.00E+00
PITHD1	Q9GZP4-2	PITH domain-containing protein 1	2.40E+05	1.22E+05	2.20E+05	3.19E+04	1.99E+03	0.00E+00
PKM	P14618	Pyruvate kinase PKM;Pyruvate kinase	1.75E+07	7.29E+06	7.21E+06	7.30E+04	3.41E+05	9.80E+04
PKN1	Q16512-3	Serine/threonine-protein kinase N1	6.38E+03	0.00E+00	0.00E+00	1.93E+04	0.00E+00	8.36E+03
PLAA	Q9Y263	Phospholipase A-2-activating protein	1.26E+05	0.00E+00	4.11E+04	9.04E+04	3.27E+04	1.68E+05
PLAT	P00750-3	Tissue-type plasminogen activator;Tissue-type plasminogen activator chain A;Tissue-type plasminogen activator chain B	2.13E+05	4.85E+04	0.00E+00	1.44E+05	9.59E+04	8.75E+04
PLCB1	Q9NQ66	1-phosphatidylinositol 4,5-bisphosphate phosphodiesterase beta-1;Phosphoinositide phospholipase C	1.28E+06	5.39E+05	5.58E+05	0.00E+00	5.46E+03	0.00E+00
PLRG1	O43660-2	Pleiotropic regulator 1	3.71E+04	0.00E+00	0.00E+00	1.58E+05	0.00E+00	9.91E+04
PML	P29590	Protein PML	3.50E+05	5.02E+05	8.99E+04	3.41E+05	2.55E+05	1.04E+05
PMP2	P02689	Myelin P2 protein	7.18E+05	6.93E+05	2.84E+05	3.80E+04	0.00E+00	0.00E+00
PMVK	Q15126	Phosphomevalonate kinase	1.41E+05	2.27E+04	6.38E+04	9.58E+04	2.28E+05	2.10E+05
PNN	Q9H307	Pinin	3.06E+05	6.60E+04	0.00E+00	1.72E+05	6.88E+05	9.22E+05
PNPLA6	Q8IY17-2	Neuropathy target esterase	3.30E+05	7.17E+04	2.18E+05	6.43E+04	1.76E+04	2.82E+04
PNPT1	Q8TCS8	Polyribonucleotide nucleotidyltransferase 1, mitochondrial	3.92E+04	0.00E+00	3.58E+04	1.66E+05	1.10E+04	7.09E+04
POGZ	Q7Z3K3-5	Pogo transposable element with ZNF domain	4.71E+04	7.77E+04	0.00E+00	9.09E+05	7.09E+05	7.63E+05
POLDIP2	B4DEM9	Polymerase delta-interacting protein 2	0.00E+00	3.54E+04	0.00E+00	5.32E+04	0.00E+00	5.55E+03
POLR1C	D6RDJ3	DNA-directed RNA polymerases I and III subunit RPAC1	0.00E+00	4.56E+04	0.00E+00	4.53E+04	2.20E+05	1.96E+05
POLR2A	P24928	DNA-directed RNA polymerase II subunit RPB1	6.70E+05	4.83E+04	5.85E+05	5.18E+05	3.68E+05	9.43E+05
POLR2B	C9J2Y9	DNA-directed RNA polymerase;DNA-directed RNA polymerase II subunit RPB2	2.97E+05	4.61E+04	2.37E+05	5.30E+05	2.68E+05	4.86E+05
POLR2C	P19387	DNA-directed RNA polymerase II subunit RPB3	8.86E+04	1.14E+04	8.98E+04	2.05E+05	2.32E+05	1.36E+05
POLR2H	C9JLU1	DNA-directed RNA polymerases I, II, and III subunit RPABC3	2.71E+04	1.90E+04	0.00E+00	1.15E+04	1.33E+05	1.31E+05
PON2	A0A0J9YYG4	Serum paraoxonase/arylesterase 2	1.46E+06	6.71E+05	3.91E+05	0.00E+00	2.14E+04	3.06E+05
POR	E7EMD0	NADPH--cytochrome P450 reductase	1.99E+05	1.99E+05	6.58E+04	0.00E+00	5.73E+04	4.37E+04
PPFIA3	O75145	Liprin-alpha-3	1.85E+05	1.21E+05	2.69E+05	1.94E+05	3.90E+05	5.75E+05
PPFIBP1	Q86W92-3	Liprin-beta-1	0.00E+00	7.70E+04	3.23E+04	1.45E+05	3.43E+05	3.11E+05
PPIA	P62937	Peptidyl-prolyl cis-trans isomerase A;Peptidyl-prolyl cis-trans isomerase A, N-terminally processed;Peptidyl-prolyl cis-trans isomerase	7.16E+06	6.44E+06	4.20E+06	1.30E+06	4.74E+04	4.15E+05
PPIB	P23284	Peptidyl-prolyl cis-trans isomerase B	4.62E+06	1.04E+06	1.14E+06	9.02E+04	5.70E+04	3.50E+05

PPIG	C9JN15	Peptidyl-prolyl cis-trans isomerase;Peptidyl-prolyl cis-trans isomerase G	2.49E+04	6.33E+04	0.00E+00	4.62E+05	1.51E+05	3.07E+05
PPM1G	O15355	Protein phosphatase 1G	2.35E+04	0.00E+00	0.00E+00	1.24E+04	0.00E+00	3.79E+04
PPP1CB	P62140	Serine/threonine-protein phosphatase PP1-beta catalytic subunit;Serine/threonine-protein phosphatase;Serine/threonine-protein phosphatase PP1-alpha catalytic subunit	1.21E+06	4.64E+05	9.25E+05	2.43E+05	4.85E+05	5.67E+05
PPP1R12A	O14974-4	Protein phosphatase 1 regulatory subunit 12A	1.32E+05	4.55E+04	0.00E+00	1.88E+05	3.19E+04	1.60E+05
PPP1R7	C9JD73	Protein phosphatase 1 regulatory subunit 7	1.02E+06	3.95E+05	7.62E+05	6.85E+04	5.45E+04	1.86E+04
PPP1R9B	D3DTX6	Neurabin-2	7.06E+04	0.00E+00	6.15E+04	3.11E+04	0.00E+00	1.88E+05
PPP2R1A	P30153	Serine/threonine-protein phosphatase 2A 65 kDa regulatory subunit A alpha isoform	3.20E+06	1.71E+06	4.17E+06	9.40E+04	2.97E+04	3.65E+04
PPP2R2A	P63151	Serine/threonine-protein phosphatase 2A 55 kDa regulatory subunit B alpha isoform	5.63E+05	3.52E+05	4.43E+05	2.38E+05	1.80E+05	2.44E+05
PPP2R4	A6PVN8	Serine/threonine-protein phosphatase 2A activator	3.32E+05	1.69E+05	7.40E+04	2.48E+04	0.00E+00	0.00E+00
PPP2R5D	E9PFR3	Serine/threonine-protein phosphatase 2A 56 kDa regulatory subunit delta isoform	3.25E+05	3.20E+04	7.70E+04	0.00E+00	1.42E+05	9.87E+04
PPP2R5E	Q16537-3	Serine/threonine-protein phosphatase 2A 56 kDa regulatory subunit epsilon isoform	3.35E+05	3.52E+04	1.17E+05	2.01E+03	3.23E+03	0.00E+00
PPP3CA	Q08209	Serine/threonine-protein phosphatase 2B catalytic subunit alpha isoform;Serine/threonine-protein phosphatase	3.89E+06	1.62E+06	1.87E+06	1.39E+05	1.86E+04	9.19E+04
PPP6C	O00743-2	Serine/threonine-protein phosphatase 6 catalytic subunit;Serine/threonine-protein phosphatase 6 catalytic subunit, N-terminally processed;Serine/threonine-protein phosphatase	4.09E+04	1.73E+04	1.37E+05	2.64E+04	5.43E+04	5.85E+04
PPP6R3	H0YEN2	Serine/threonine-protein phosphatase 6 regulatory subunit 3	0.00E+00	1.91E+04	0.00E+00	1.41E+04	4.26E+04	9.47E+04
PPT1	A0A2C9F2P4	Palmitoyl-protein thioesterase 1	1.49E+06	9.32E+05	9.76E+05	7.86E+05	7.80E+05	8.34E+05
PRAF2	O60831	PRA1 family protein 2	9.33E+04	0.00E+00	0.00E+00	0.00E+00	1.26E+04	0.00E+00
PRCC	Q92733	Proline-rich protein PRCC	1.76E+04	0.00E+00	1.76E+04	1.13E+05	5.56E+04	8.08E+04
PRDX1	Q06830	Peroxiredoxin-1	3.48E+06	1.57E+06	1.12E+06	1.58E+05	3.11E+04	7.58E+04
PRDX4	Q13162	Peroxiredoxin-4	2.95E+05	1.22E+05	0.00E+00	0.00E+00	2.30E+04	0.00E+00
PREB	B5MC98	Prolactin regulatory element-binding protein	2.37E+05	1.74E+05	4.68E+04	2.79E+04	3.61E+04	5.13E+04
PREX1	Q8TCU6-2	Phosphatidylinositol 3,4,5-trisphosphate-dependent Rac exchanger 1 protein	2.06E+04	9.50E+05	6.54E+05	5.72E+04	0.00E+00	0.00E+00
PRKAA1	Q13131	5-AMP-activated protein kinase catalytic subunit alpha-1	3.56E+05	5.07E+04	1.13E+04	5.72E+04	6.89E+04	8.58E+04
PRKACB	P22694-2	cAMP-dependent protein kinase catalytic subunit beta	3.14E+05	1.00E+05	9.31E+04	1.60E+05	9.09E+04	6.20E+04
PRKAR1A	P10644	cAMP-dependent protein kinase type I-alpha regulatory	6.49E+05	5.64E+05	5.73E+05	1.59E+05	2.66E+05	1.78E+05

		subunit;cAMP-dependent protein kinase type I-alpha regulatory subunit, N-terminally processed						
PRKAR2A	P13861-2	cAMP-dependent protein kinase type II-alpha regulatory subunit	1.26E+05	9.36E+04	1.92E+05	4.66E+04	0.00E+00	0.00E+00
PRKAR2B	P31323	cAMP-dependent protein kinase type II-beta regulatory subunit	8.64E+05	4.78E+05	1.44E+06	1.09E+05	4.48E+04	6.27E+04
PRKCA	P17252	Protein kinase C alpha type	1.56E+05	8.93E+04	5.31E+04	7.30E+04	1.34E+05	1.83E+05
PRKDC	P78527	DNA-dependent protein kinase catalytic subunit	2.37E+06	1.14E+06	1.07E+06	1.82E+06	3.22E+05	6.44E+05
PRKRA	O75569-3	Interferon-inducible double-stranded RNA-dependent protein kinase activator A	3.76E+05	6.45E+04	1.87E+05	1.37E+05	3.46E+04	1.12E+05
PRMT5	O14744-5	Protein arginine N-methyltransferase 5;Protein arginine N-methyltransferase 5, N-terminally processed	2.96E+05	1.26E+05	7.50E+04	0.00E+00	1.25E+04	0.00E+00
PROSC	O94903	Proline synthase co-transcribed bacterial homolog protein	1.60E+05	1.01E+05	8.20E+04	3.13E+04	0.00E+00	0.00E+00
PRPF19	Q9UMS4	Pre-mRNA-processing factor 19	1.81E+05	1.30E+04	2.09E+04	2.93E+04	0.00E+00	0.00E+00
PRPF31	E7EVX8	U4/U6 small nuclear ribonucleoprotein Prp31	7.18E+04	3.74E+03	3.00E+04	4.98E+05	2.24E+05	3.25E+05
PRPF38A	Q8NAV1	Pre-mRNA-splicing factor 38A	4.90E+03	3.27E+04	3.40E+05	2.58E+04	6.40E+04	8.55E+04
PRPF4	O43172-2	U4/U6 small nuclear ribonucleoprotein Prp4	1.84E+05	2.46E+04	1.15E+05	3.71E+04	0.00E+00	7.55E+04
PRPF40A	O75400-2	Pre-mRNA-processing factor 40 homolog A	6.52E+05	1.45E+05	3.37E+05	1.19E+05	1.66E+05	1.49E+05
PRPF4B	Q13523	Serine/threonine-protein kinase PRP4 homolog	3.26E+04	6.68E+03	0.00E+00	1.97E+05	5.31E+04	2.76E+05
PRPF6	O94906	Pre-mRNA-processing factor 6	3.64E+05	1.75E+05	1.17E+04	3.20E+05	7.08E+05	6.66E+05
PRPF8	Q6P2Q9	Pre-mRNA-processing-splicing factor 8	4.33E+06	1.42E+06	1.35E+06	5.25E+05	6.23E+05	5.27E+05
PSIP1	O75475	PC4 and SFRS1-interacting protein	8.38E+05	5.06E+05	2.65E+05	4.84E+06	3.74E+06	3.94E+06
PSMA4	H0YKT8	Proteasome subunit beta type;Proteasome subunit alpha type;Proteasome subunit alpha type-4	1.64E+05	4.66E+04	2.98E+04	1.15E+04	0.00E+00	0.00E+00
PSMA5	P28066-2	Proteasome subunit alpha type-5	1.57E+05	1.07E+05	7.75E+04	0.00E+00	1.00E+05	5.57E+04
PSMA7	O14818	Proteasome subunit alpha type-7;Proteasome subunit alpha type-7-like	5.39E+05	1.39E+05	3.02E+05	4.07E+04	3.71E+04	4.02E+04
PSMB1	P20618	Proteasome subunit beta type-1	1.80E+05	6.38E+04	2.20E+05	0.00E+00	2.23E+04	0.00E+00
PSMB2	P49721	Proteasome subunit beta type-2	2.69E+05	2.87E+04	2.95E+04	0.00E+00	3.24E+04	0.00E+00
PSMB5	P28074	Proteasome subunit beta type-5	1.45E+05	1.29E+04	2.88E+04	0.00E+00	1.98E+04	0.00E+00
PSMC1	P62191	26S protease regulatory subunit 4	5.43E+05	2.49E+05	3.40E+05	0.00E+00	2.18E+05	1.81E+05
PSMC2	P35998	26S protease regulatory subunit 7	5.29E+05	2.16E+05	1.75E+05	2.31E+05	1.88E+05	4.40E+05
PSMC3	E9PM69	26S protease regulatory subunit 6A	1.00E+06	1.91E+05	8.01E+05	2.00E+05	2.67E+05	4.93E+05
PSMC5	P62195-2	26S protease regulatory subunit 8	5.61E+05	1.73E+05	3.89E+05	0.00E+00	2.08E+05	2.42E+05
PSMC6	A0A087X2I1	26S protease regulatory subunit 10B	5.23E+05	2.20E+05	2.71E+05	1.29E+05	1.32E+05	4.65E+04
PSMD1	Q99460-2	26S proteasome non-ATPase regulatory subunit 1	1.48E+06	1.05E+06	2.11E+06	3.12E+04	4.51E+04	9.07E+04

PSMD11	O00231	26S proteasome non-ATPase regulatory subunit 11	6.42E+05	3.84E+05	6.41E+05	2.67E+04	8.40E+04	3.36E+04
PSMD12	O00232	26S proteasome non-ATPase regulatory subunit 12	3.94E+05	8.26E+04	2.27E+05	0.00E+00	8.22E+04	9.68E+04
PSMD13	Q9UNM6	26S proteasome non-ATPase regulatory subunit 13	3.12E+05	8.46E+04	5.12E+05	0.00E+00	1.75E+04	3.82E+04
PSMD2	Q13200	26S proteasome non-ATPase regulatory subunit 2	4.88E+05	1.53E+05	3.03E+05	7.78E+04	2.37E+05	2.12E+05
PSMD3	O43242	26S proteasome non-ATPase regulatory subunit 3	1.58E+06	4.76E+05	6.92E+05	5.23E+05	5.14E+05	9.97E+05
PSMD4	P55036	26S proteasome non-ATPase regulatory subunit 4	1.64E+05	1.36E+05	3.65E+02	1.14E+04	5.64E+03	8.94E+04
PSMD5	Q16401	26S proteasome non-ATPase regulatory subunit 5	2.63E+05	0.00E+00	2.08E+05	5.07E+03	0.00E+00	8.31E+03
PSMD6	Q15008	26S proteasome non-ATPase regulatory subunit 6	6.98E+05	2.89E+05	7.34E+05	0.00E+00	1.57E+05	9.48E+04
PSMD7	P51665	26S proteasome non-ATPase regulatory subunit 7	2.70E+05	3.74E+04	3.64E+05	1.09E+05	0.00E+00	1.43E+05
PSPC1	Q8WXF1	Paraspeckle component 1	9.88E+04	1.15E+05	1.23E+05	1.52E+05	0.00E+00	2.99E+04
PTBP1	A6NLN1	Polypyrimidine tract-binding protein 1	3.44E+05	1.29E+05	1.80E+05	1.05E+06	8.25E+03	1.81E+05
PTBP2	Q9UKA9-5	Polypyrimidine tract-binding protein 2	1.64E+05	0.00E+00	1.36E+05	5.24E+04	3.87E+04	1.33E+05
PTGES3	A0A087WYT3	Prostaglandin E synthase 3	1.39E+06	6.92E+05	6.15E+04	7.19E+05	5.58E+05	6.55E+05
PTGES3L-AARSD1	C9J5N1	Alanyl-tRNA editing protein Aarsd1	2.72E+04	0.00E+00	1.60E+04	2.57E+04	0.00E+00	0.00E+00
PTK2	E9PEI4	Focal adhesion kinase 1	7.70E+04	3.53E+04	4.05E+04	2.44E+03	3.06E+04	3.77E+04
PTPN1	B4DSN5	Tyrosine-protein phosphatase non-receptor type;Tyrosine-protein phosphatase non-receptor type 1	6.79E+04	0.00E+00	0.00E+00	1.57E+04	8.77E+03	0.00E+00
PTPN11	Q06124-2	Tyrosine-protein phosphatase non-receptor type 11	4.67E+05	4.49E+04	1.17E+05	3.58E+04	0.00E+00	0.00E+00
PTPN23	Q9H3S7	Tyrosine-protein phosphatase non-receptor type 23	6.96E+04	4.64E+04	6.52E+04	4.72E+04	2.68E+04	1.24E+05
PTRF	Q6NZI2	Polymerase I and transcript release factor	5.48E+06	4.47E+06	2.81E+06	6.25E+06	4.68E+06	4.96E+06
PUF60	Q9UHX1	Poly(U)-binding-splicing factor PUF60	4.03E+05	8.46E+04	2.30E+05	1.61E+05	2.41E+05	3.46E+05
PURA	Q00577	Transcriptional activator protein Pur-alpha	6.64E+06	5.07E+06	4.15E+06	3.09E+06	3.16E+06	4.79E+06
PURB	Q96QR8	Transcriptional activator protein Pur-beta	8.29E+04	5.74E+04	1.80E+05	1.07E+04	3.97E+04	1.03E+05
PXDN	Q92626	Peroxidasin homolog	0.00E+00	8.83E+05	0.00E+00	0.00E+00	4.66E+04	0.00E+00
PXN	F5GZ78	Paxillin	0.00E+00	2.76E+04	0.00E+00	7.25E+04	8.76E+03	7.24E+03
PYGB	P11216	Glycogen phosphorylase, brain form	6.02E+06	1.66E+06	2.12E+06	9.57E+05	7.74E+05	9.91E+05
PYGM	P11217	Glycogen phosphorylase, muscle form	2.49E+06	2.03E+05	8.68E+05	8.53E+05	2.79E+05	4.48E+05
QARS	P47897	Glutamine--tRNA ligase	5.53E+05	8.53E+04	8.46E+03	4.91E+04	1.28E+04	4.26E+04
QDPR	P09417	Dihydropteridine reductase	9.07E+06	3.94E+06	6.94E+06	5.71E+04	4.42E+04	7.72E+03
QKI	Q96PU8-5	Protein quaking	1.18E+06	6.65E+04	1.69E+05	8.30E+05	4.46E+05	1.24E+06
QRICH1	Q2TAL8	Glutamine-rich protein 1	4.52E+04	0.00E+00	0.00E+00	1.98E+05	3.58E+05	5.41E+05
RAB10	P61026	Ras-related protein Rab-10	3.18E+05	3.25E+05	3.11E+05	1.24E+04	5.76E+04	8.73E+04
RAB11B	Q15907-2	Ras-related protein Rab-11B;Ras-related protein Rab-11A	3.77E+05	1.52E+05	3.49E+05	1.44E+04	0.00E+00	0.00E+00
RAB11FIP5	A0A1B0GTL5	Rab11 family-interacting protein 5	7.20E+04	6.67E+03	6.87E+04	1.40E+05	1.26E+05	1.93E+05
RAB12	Q6IQ22	Ras-related protein Rab-12	2.03E+05	9.02E+04	8.24E+04	0.00E+00	8.40E+03	2.23E+04

RAB14	P61106	Ras-related protein Rab-14	1.13E+06	7.97E+05	9.33E+05	0.00E+00	5.14E+04	0.00E+00
RAB1B	E9PLD0	Ras-related protein Rab-1B;Putative Ras-related protein Rab-1C	2.49E+06	6.90E+05	3.29E+06	1.41E+05	1.61E+05	0.00E+00
RAB2A	P61019	Ras-related protein Rab-2A	2.70E+06	2.81E+06	1.02E+06	0.00E+00	3.02E+04	0.00E+00
RAB30	H0YDK7	Ras-related protein Rab-30	0.00E+00	3.10E+04	1.72E+04	2.65E+04	3.75E+04	0.00E+00
RAB35	Q15286	Ras-related protein Rab-35	2.66E+05	1.43E+05	3.90E+04	3.41E+04	2.61E+04	3.48E+04
RAB39B	Q96DA2	Ras-related protein Rab-39B	1.18E+05	0.00E+00	0.00E+00	6.35E+03	0.00E+00	0.00E+00
RAB3C	Q96E17	Ras-related protein Rab-3C	1.07E+06	9.79E+05	1.10E+06	2.14E+05	3.17E+05	3.87E+05
RAB3GAP1	Q15042-4	Rab3 GTPase-activating protein catalytic subunit	4.70E+03	2.41E+04	3.46E+04	6.04E+04	0.00E+00	1.61E+04
RAB3GAP2	Q9H2M9	Rab3 GTPase-activating protein non-catalytic subunit	3.84E+04	4.29E+04	4.30E+04	1.42E+05	8.33E+04	4.41E+05
RAB5C	P51148	Ras-related protein Rab-5C	1.14E+06	1.01E+06	8.24E+05	3.28E+04	1.78E+04	0.00E+00
RAB6A	P20340-2	Ras-related protein Rab-6A	6.25E+05	3.79E+05	6.02E+04	1.12E+05	0.00E+00	3.36E+05
RAB7A	P51149	Ras-related protein Rab-7a	7.89E+05	6.94E+05	6.11E+05	0.00E+00	7.13E+03	3.12E+03
RAB9A	P51151	Ras-related protein Rab-9A	1.06E+04	5.13E+04	0.00E+00	0.00E+00	2.30E+04	0.00E+00
RABEP1	Q15276	Rab GTPase-binding effector protein 1	2.45E+05	0.00E+00	2.69E+04	4.64E+04	0.00E+00	0.00E+00
RABGAP1	Q9Y3P9	Rab GTPase-activating protein 1	1.64E+05	5.10E+03	1.29E+05	4.72E+04	3.65E+04	0.00E+00
RABL6	H0Y4Z8	Rab-like protein 6	9.63E+04	8.62E+03	1.69E+04	3.96E+03	0.00E+00	9.67E+03
RAC1	P63000	Ras-related C3 botulinum toxin substrate 1;Ras-related C3 botulinum toxin substrate 3;Ras-related C3 botulinum toxin substrate 2	1.27E+06	9.32E+05	5.92E+05	9.45E+05	4.08E+05	5.75E+05
RAD21	O60216	Double-strand-break repair protein rad21 homolog	1.71E+05	6.51E+04	1.05E+05	3.81E+05	7.01E+05	9.32E+05
RAD23B	P54727	UV excision repair protein RAD23 homolog B	1.70E+05	0.00E+00	1.34E+05	8.33E+04	3.80E+04	6.91E+04
RAD50	Q92878	DNA repair protein RAD50	5.82E+05	4.93E+05	1.69E+05	2.46E+06	2.61E+06	1.59E+06
RAI14	Q9P0K7-4	Ankycorbin	0.00E+00	1.03E+04	0.00E+00	1.36E+05	0.00E+00	8.08E+04
RALA	H7C3P7	Ras-related protein Ral-A	2.07E+06	1.79E+06	4.25E+05	2.17E+05	2.95E+05	6.83E+05
RALY	Q5QPM1	RNA-binding protein Raly	3.60E+05	6.17E+05	3.52E+03	8.62E+05	8.28E+05	8.67E+05
RALYL	Q86SE5	RNA-binding Raly-like protein	0.00E+00	2.44E+04	2.95E+04	3.73E+04	0.00E+00	0.00E+00
RAN	F5H018	GTP-binding nuclear protein Ran	3.93E+05	1.79E+05	5.90E+04	3.11E+04	1.06E+05	2.31E+05
RANBP1	C9JJ34	Ran-specific GTPase-activating protein	3.61E+05	2.00E+05	9.76E+04	7.18E+05	2.40E+05	3.82E+05
RANBP2	P49792	E3 SUMO-protein ligase RanBP2	1.33E+06	8.24E+05	5.64E+05	1.66E+06	1.31E+06	1.98E+06
RANBP9	Q96S59	Ran-binding protein 9	7.51E+04	6.50E+04	5.73E+04	6.19E+04	1.20E+05	1.26E+05
RANGAP1	P46060	Ran GTPase-activating protein 1	3.38E+05	2.26E+05	1.34E+05	1.75E+05	1.36E+05	3.66E+05
RAP1A	P62834	Ras-related protein Rap-1A	2.66E+06	1.93E+06	1.18E+06	7.85E+04	2.12E+05	3.90E+04
RAP1B	P61224	Ras-related protein Rap-1b;Ras-related protein Rap-1b-like protein	7.76E+05	2.12E+05	1.58E+05	1.86E+04	0.00E+00	2.40E+04
RAP1GDS1	P52306-4	Rap1 GTPase-GDP dissociation stimulator 1	1.01E+06	6.27E+05	8.04E+05	5.65E+04	7.20E+04	5.73E+04

RAP2A	P10114	Ras-related protein Rap-2a;Ras-related protein Rap-2c	5.08E+05	3.74E+05	1.48E+05	4.75E+04	2.82E+05	2.32E+05
RAPGEF2	Q9Y4G8	Rap guanine nucleotide exchange factor 2	3.24E+04	3.84E+04	0.00E+00	2.31E+04	0.00E+00	0.00E+00
RAPH1	C9K0J5	Ras-associated and pleckstrin homology domains-containing protein 1	1.84E+04	0.00E+00	0.00E+00	0.00E+00	2.70E+04	3.65E+04
RARS	P54136	Arginine--tRNA ligase, cytoplasmic	3.37E+05	6.05E+04	5.01E+04	7.04E+04	0.00E+00	2.17E+04
RASAL2	Q9UJF2-2	Ras GTPase-activating protein nGAP	0.00E+00	2.71E+04	5.44E+04	1.42E+04	4.59E+04	5.75E+04
RASIP1	Q5U651	Ras-interacting protein 1	3.36E+04	5.21E+04	4.70E+04	7.00E+05	8.04E+05	6.95E+05
RASSF2	P50749	Ras association domain-containing protein 2;Ras association domain-containing protein 4	6.40E+03	0.00E+00	0.00E+00	5.56E+04	0.00E+00	0.00E+00
RBBP4	Q09028-4	Histone-binding protein RBBP4	1.89E+04	8.22E+03	0.00E+00	0.00E+00	2.53E+04	0.00E+00
RBBP7	E9PC52	Histone-binding protein RBBP7	4.45E+05	5.01E+05	2.03E+05	5.73E+05	8.47E+05	9.17E+05
RBFOX2	O43251-6	RNA binding protein fox-1 homolog 2;RNA binding protein fox-1 homolog	1.82E+05	0.00E+00	0.00E+00	1.28E+05	3.14E+05	3.05E+05
RBM10	A0A0A0MR66	RNA-binding protein 10	6.91E+04	1.49E+04	0.00E+00	4.80E+04	2.34E+04	1.18E+05
RBM14	Q96PK6	RNA-binding protein 14	7.99E+05	1.37E+05	2.74E+04	1.54E+06	1.07E+06	1.85E+06
RBM15	A0A087WWP4	Putative RNA-binding protein 15	1.79E+04	0.00E+00	0.00E+00	2.23E+05	7.53E+04	2.01E+05
RBM17	Q96I25	Splicing factor 45	7.62E+04	6.21E+04	0.00E+00	4.24E+04	1.10E+05	2.15E+05
RBM22	Q9NW64	Pre-mRNA-splicing factor RBM22	1.27E+05	4.70E+04	1.15E+04	2.20E+05	2.96E+04	1.63E+05
RBM25	P49756	RNA-binding protein 25	1.01E+05	0.00E+00	0.00E+00	4.34E+04	1.20E+04	1.21E+05
RBM26	A0A087X0H9	RNA-binding protein 26	3.17E+04	0.00E+00	0.00E+00	1.13E+05	2.63E+03	6.98E+04
RBM39	Q14498-3	RNA-binding protein 39	6.78E+05	2.81E+05	4.93E+05	1.50E+06	1.11E+06	2.02E+06
RBM4B	Q9BQ04	RNA-binding protein 4B	1.24E+05	2.31E+04	0.00E+00	2.34E+05	6.79E+05	7.74E+05
RBM8A	Q9Y5S9	RNA-binding protein 8A	4.62E+05	2.54E+05	9.18E+04	1.54E+05	3.19E+05	3.85E+05
RBMX	P38159	RNA-binding motif protein, X chromosome;RNA-binding motif protein, X chromosome, N-terminally processed;RNA binding motif protein, X-linked-like-1	2.33E+05	1.97E+05	6.23E+04	1.30E+06	1.19E+06	1.63E+06
RCC1	P18754	Regulator of chromosome condensation	6.90E+04	4.43E+04	9.53E+04	1.89E+05	8.84E+04	2.56E+05
RCN2	Q14257	Reticulocalbin-2	6.71E+05	3.90E+05	6.83E+05	1.16E+04	2.04E+04	4.21E+04
RDH11	Q8TC12	Retinol dehydrogenase 11	4.97E+05	2.62E+05	7.32E+05	8.76E+04	0.00E+00	0.00E+00
RDX	P35241	Radixin	9.88E+05	7.55E+05	5.11E+05	2.29E+04	2.06E+04	3.12E+04
RELA	Q2TAM5	Transcription factor p65	3.45E+04	1.48E+04	3.83E+04	1.24E+05	5.94E+04	5.67E+04
RER1	Q5T092	Protein RER1	1.56E+05	1.26E+05	0.00E+00	0.00E+00	6.45E+03	7.07E+03
RFC1	P35251-2	Replication factor C subunit 1	1.78E+03	1.49E+04	2.80E+04	5.38E+05	5.37E+05	4.39E+05
RFC3	P40938	Replication factor C subunit 3	4.02E+04	0.00E+00	0.00E+00	5.14E+04	9.42E+04	9.69E+04
RFC4	P35249	Replication factor C subunit 4	5.98E+04	3.95E+04	0.00E+00	1.53E+05	2.36E+05	1.44E+05
RFC5	P40937	Replication factor C subunit 5	3.23E+04	1.96E+04	0.00E+00	2.26E+04	5.06E+04	2.88E+04

RFTN1	Q14699	Raftlin	5.08E+04	1.39E+04	0.00E+00	2.04E+03	0.00E+00	0.00E+00
RHOB	P62745	Rho-related GTP-binding protein RhoB	1.50E+06	9.47E+05	4.06E+05	0.00E+00	1.56E+03	1.23E+05
RHOG	P84095	Rho-related GTP-binding protein RhoG	1.26E+06	3.17E+05	4.42E+05	4.31E+05	4.98E+05	4.06E+05
RHOT1	H7BXZ6	Mitochondrial Rho GTPase;Mitochondrial Rho GTPase 1	5.22E+05	4.90E+05	2.38E+05	1.83E+05	1.54E+05	1.77E+05
RHOT2	Q8IXI1	Mitochondrial Rho GTPase 2	8.85E+04	1.04E+05	3.61E+04	3.77E+04	4.39E+04	4.54E+04
RIF1	Q5UIP0-2	Telomere-associated protein RIF1	1.09E+05	0.00E+00	0.00E+00	1.51E+05	3.90E+04	1.12E+05
RING1	Q06587	E3 ubiquitin-protein ligase RING1	1.05E+05	0.00E+00	1.57E+04	9.84E+03	3.27E+04	9.40E+04
RMDN3	Q96TC7	Regulator of microtubule dynamics protein 3	8.81E+04	5.39E+03	4.90E+04	3.12E+04	1.41E+04	6.59E+04
RNF114	A0A096LNV3	E3 ubiquitin-protein ligase RNF114	1.17E+04	0.00E+00	0.00E+00	5.32E+03	0.00E+00	3.29E+03
RNF20	Q5VTR2	E3 ubiquitin-protein ligase BRE1A	1.52E+04	3.88E+04	2.03E+04	1.80E+05	6.48E+04	1.79E+05
RNF40	H3BP71	E3 ubiquitin-protein ligase BRE1B	1.78E+04	0.00E+00	1.51E+04	3.40E+04	0.00E+00	2.18E+04
RNH1	P13489	Ribonuclease inhibitor	1.79E+06	9.34E+05	1.39E+06	1.93E+05	2.14E+05	3.13E+05
RNPS1	H3BV80	RNA-binding protein with serine-rich domain 1	7.32E+05	2.61E+05	2.65E+03	8.88E+05	1.18E+06	7.42E+05
RPA1	P27694	Replication protein A 70 kDa DNA-binding subunit;Replication protein A 70 kDa DNA-binding subunit, N-terminally processed	3.00E+04	0.00E+00	5.49E+04	4.80E+05	4.05E+05	4.68E+05
RPL10	F8W7C6	60S ribosomal protein L10	2.28E+05	1.56E+05	9.78E+04	2.82E+05	2.54E+05	1.63E+05
RPL10A	P62906	60S ribosomal protein L10a	4.46E+05	1.83E+05	0.00E+00	3.84E+05	9.05E+04	2.29E+05
RPL12	P30050	60S ribosomal protein L12	1.11E+06	7.75E+05	3.54E+05	3.60E+05	6.25E+05	5.33E+05
RPL13	P26373	60S ribosomal protein L13	9.31E+04	4.58E+04	1.39E+04	1.78E+06	4.16E+05	1.07E+06
RPL13A	M0QYS1	60S ribosomal protein L13a;Putative 60S ribosomal protein L13a protein RPL13AP3	1.91E+05	0.00E+00	3.17E+04	1.77E+06	8.58E+05	1.45E+06
RPL14	E7EPB3	60S ribosomal protein L14	6.68E+04	7.89E+04	7.53E+04	1.20E+06	3.83E+05	6.16E+05
RPL15	E7EQV9	Ribosomal protein L15;60S ribosomal protein L15	1.34E+05	1.18E+05	4.96E+04	8.47E+05	4.30E+05	4.47E+05
RPL17	A0A087WXM6	60S ribosomal protein L17	1.23E+05	6.43E+04	1.26E+05	4.30E+05	7.95E+05	7.32E+05
RPL18	G3V203	60S ribosomal protein L18	2.81E+05	1.49E+05	1.97E+05	2.56E+06	1.64E+06	2.46E+06
RPL18A	M0R3D6	60S ribosomal protein L18a	3.17E+05	2.73E+05	7.03E+04	8.94E+05	8.89E+05	1.06E+06
RPL21	P46778	60S ribosomal protein L21	4.52E+05	1.05E+05	7.44E+04	7.98E+05	3.00E+05	3.52E+05
RPL22	P35268	60S ribosomal protein L22	2.36E+05	1.47E+05	4.66E+05	1.36E+05	3.10E+04	3.39E+05
RPL23	C9JD32	60S ribosomal protein L23	8.08E+05	1.83E+05	5.61E+05	1.68E+06	1.88E+06	1.52E+06
RPL24	C9JXB8	60S ribosomal protein L24	0.00E+00	1.21E+05	0.00E+00	2.51E+05	1.31E+05	1.92E+05
RPL27	P61353	60S ribosomal protein L27	1.45E+05	8.16E+04	0.00E+00	1.04E+05	1.21E+05	1.11E+05
RPL3	P39023	60S ribosomal protein L3	3.06E+05	2.34E+05	1.49E+05	5.19E+05	2.71E+05	5.71E+05
RPL30	E5RI99	60S ribosomal protein L30	3.77E+05	4.61E+05	1.17E+05	1.09E+05	7.58E+04	8.82E+04
RPL31	H7C2W9	60S ribosomal protein L31	3.54E+04	5.20E+04	0.00E+00	6.10E+05	3.51E+05	5.18E+05

RPL32	D3YTB1	60S ribosomal protein L32	2.66E+05	1.84E+05	1.64E+05	5.14E+04	1.35E+05	2.88E+05
RPL36	Q9Y3U8	60S ribosomal protein L36	8.16E+04	8.27E+04	3.02E+04	1.47E+05	3.97E+05	3.71E+05
RPL38	J3KT73	60S ribosomal protein L38	7.08E+04	0.00E+00	7.69E+04	2.50E+04	0.00E+00	0.00E+00
RPL4	P36578	60S ribosomal protein L4	4.39E+05	5.46E+05	3.10E+05	9.90E+05	1.38E+06	1.27E+06
RPL5	P46777	60S ribosomal protein L5	1.60E+06	7.84E+05	1.18E+06	8.72E+04	0.00E+00	2.12E+05
RPL6	Q02878	60S ribosomal protein L6	1.89E+05	1.31E+05	1.17E+05	9.63E+05	5.10E+05	1.40E+06
RPL7	A8MUD9	60S ribosomal protein L7	2.98E+05	8.02E+04	8.20E+04	1.83E+06	1.46E+06	1.38E+06
RPL7A	P62424	60S ribosomal protein L7a	2.02E+05	4.15E+04	7.90E+04	5.55E+05	1.14E+06	7.32E+05
RPL9	D6RAN4	60S ribosomal protein L9	1.28E+05	2.06E+05	3.13E+05	1.77E+05	2.34E+05	2.79E+05
RPLP0	P05388	60S acidic ribosomal protein P0;60S acidic ribosomal protein P0-like	2.48E+06	1.77E+06	1.30E+06	2.57E+04	2.27E+04	1.20E+05
RPLP2	P05387	60S acidic ribosomal protein P2	2.60E+06	1.14E+06	1.57E+06	0.00E+00	2.46E+04	0.00E+00
RPN1	P04843	Dolichyl-diphosphooligosaccharide--protein glycosyltransferase subunit 1	3.64E+06	1.58E+06	2.32E+06	9.44E+03	0.00E+00	3.57E+04
RPN2	P04844-2	Dolichyl-diphosphooligosaccharide--protein glycosyltransferase subunit 2	9.15E+05	5.88E+05	4.74E+05	3.61E+04	1.49E+04	5.25E+04
RPRD1A	A0A0C4DGQ6	Regulation of nuclear pre-mRNA domain-containing protein 1A	4.85E+04	0.00E+00	6.98E+04	5.20E+05	6.20E+03	3.60E+05
RPRD1B	Q9NQG5	Regulation of nuclear pre-mRNA domain-containing protein 1B	2.48E+04	0.00E+00	2.78E+04	3.71E+04	1.94E+04	3.66E+04
RPRD2	Q5VT52-5	Regulation of nuclear pre-mRNA domain-containing protein 2	1.47E+04	0.00E+00	0.00E+00	4.46E+04	2.24E+04	8.50E+04
RPS11	M0QZC5	40S ribosomal protein S11	1.19E+05	0.00E+00	0.00E+00	4.17E+05	6.12E+05	4.34E+05
RPS14	P62263	40S ribosomal protein S14	4.40E+05	4.17E+05	3.92E+03	4.11E+05	1.28E+06	5.19E+05
RPS15A	P62244	40S ribosomal protein S15a	3.61E+05	1.00E+05	0.00E+00	5.85E+04	0.00E+00	0.00E+00
RPS16	M0R3H0	40S ribosomal protein S16	1.91E+04	1.08E+05	8.68E+04	2.81E+05	0.00E+00	2.34E+05
RPS17	P08708	40S ribosomal protein S17	1.84E+05	2.03E+04	0.00E+00	4.25E+05	1.85E+05	2.06E+05
RPS18	P62269	40S ribosomal protein S18	9.15E+04	4.98E+04	0.00E+00	1.66E+05	1.01E+05	1.79E+05
RPS19	P39019	40S ribosomal protein S19	7.70E+04	3.73E+04	4.24E+04	4.94E+04	3.58E+04	1.19E+05
RPS2	H0YEN5	40S ribosomal protein S2	1.18E+05	8.46E+04	6.75E+04	3.48E+04	1.04E+04	3.85E+05
RPS20	P60866	40S ribosomal protein S20	0.00E+00	3.41E+04	7.40E+04	2.20E+05	9.22E+04	3.33E+05
RPS24	E7ETK0	40S ribosomal protein S24	1.03E+05	0.00E+00	7.34E+04	7.79E+05	3.24E+05	4.11E+05
RPS26	P62854	40S ribosomal protein S26;Putative 40S ribosomal protein S26-like 1	2.18E+04	1.29E+04	8.61E+03	4.31E+04	6.35E+04	9.68E+04
RPS27A	P62979	Ubiquitin-40S ribosomal protein S27a;Ubiquitin;40S ribosomal protein S27a;Polyubiquitin-C;Ubiquitin;Ubiquitin-60S ribosomal protein	2.28E+05	5.53E+05	0.00E+00	6.76E+05	1.07E+06	2.34E+05

		L40;Ubiquitin;60S ribosomal protein L40;Polyubiquitin-B;Ubiquitin						
RPS3	E9PPU1	40S ribosomal protein S3	1.09E+06	3.57E+05	5.44E+05	1.70E+05	3.03E+05	1.65E+05
RPS3A	D6RG13	40S ribosomal protein S3a	1.88E+05	9.46E+03	2.79E+05	2.82E+04	9.13E+03	8.95E+03
RPS4X	P62701	40S ribosomal protein S4, X isoform	1.77E+05	0.00E+00	1.02E+05	8.77E+05	7.47E+05	7.84E+05
RPS6	P62753	40S ribosomal protein S6	4.20E+04	7.57E+04	0.00E+00	8.22E+05	1.20E+05	6.94E+05
RPS7	P62081	40S ribosomal protein S7	1.26E+05	4.07E+04	0.00E+00	1.97E+04	3.92E+04	2.87E+04
RPS8	P62241	40S ribosomal protein S8	3.97E+05	2.35E+05	2.30E+04	4.03E+05	1.46E+06	3.66E+05
RPSA	C9J9K3	40S ribosomal protein SA	1.16E+06	3.39E+05	2.76E+05	3.79E+04	1.70E+04	1.34E+05
RRAGA	Q7L523	Ras-related GTP-binding protein A;Ras-related GTP-binding protein B	6.47E+04	0.00E+00	7.62E+04	0.00E+00	2.52E+04	0.00E+00
RRAGC	Q9HB90	Ras-related GTP-binding protein C;Ras-related GTP-binding protein D	1.38E+05	8.00E+04	0.00E+00	5.20E+04	6.45E+04	4.21E+04
RRAS	P10301	Ras-related protein R-Ras	4.09E+05	5.74E+05	6.79E+05	5.51E+04	1.26E+05	8.50E+04
RRBP1	A0A0A0MRV0	Ribosome-binding protein 1	9.38E+05	3.24E+05	3.61E+05	2.74E+04	2.15E+04	6.35E+04
RRP1	P56182	Ribosomal RNA processing protein 1 homolog A	6.64E+04	4.64E+04	0.00E+00	3.45E+05	5.52E+05	4.45E+05
RRP12	Q5JTH9	RRP12-like protein	0.00E+00	1.42E+03	2.66E+04	1.15E+06	8.48E+05	1.10E+06
RRP9	O43818	U3 small nucleolar RNA-interacting protein 2	0.00E+00	8.12E+03	0.00E+00	0.00E+00	2.43E+04	9.79E+03
RSL1D1	J3QSV6	Ribosomal L1 domain-containing protein 1	0.00E+00	4.09E+03	0.00E+00	1.83E+05	5.58E+05	2.93E+05
RSRC2	Q7L4I2-2	Arginine/serine-rich coiled-coil protein 2	2.52E+04	7.31E+03	0.00E+00	3.94E+04	5.91E+03	4.79E+04
RSU1	Q15404	Ras suppressor protein 1	4.07E+05	4.10E+05	2.84E+05	1.49E+04	0.00E+00	0.00E+00
RTCA	O00442-2	RNA 3-terminal phosphate cyclase	5.15E+04	7.67E+04	5.17E+04	0.00E+00	1.23E+05	0.00E+00
RTCB	Q9Y3I0	tRNA-splicing ligase RtcB homolog	1.02E+06	5.79E+05	5.42E+04	7.80E+05	2.04E+04	6.83E+04
RTFDC1	A0A0A0MQR2	Protein RTF2 homolog	1.39E+04	0.00E+00	0.00E+00	2.33E+05	4.15E+04	2.56E+05
RTN1	Q16799	Reticulon-1;Reticulon	1.66E+05	6.75E+04	7.66E+04	3.03E+04	1.19E+03	8.80E+03
RTN3	O95197-7	Reticulon-3	1.44E+06	8.95E+05	7.04E+05	2.23E+04	3.51E+04	1.81E+04
RUVBL1	Q9Y265	RuvB-like 1	8.34E+05	6.45E+05	3.26E+05	2.65E+05	2.84E+05	4.09E+05
RUVBL2	Q9Y230	RuvB-like 2	9.48E+05	3.04E+05	2.57E+05	2.09E+05	2.34E+05	2.64E+05
SACM1L	Q9NTJ5	Phosphatidylinositide phosphatase SAC1	7.37E+05	4.43E+05	1.71E+05	2.08E+04	1.87E+04	2.01E+04
SACS	Q9NZJ4	Sacsin	3.33E+04	4.84E+04	0.00E+00	1.00E+04	2.82E+04	0.00E+00
SAE1	M0QZS6	SUMO-activating enzyme subunit 1;SUMO-activating enzyme subunit 1, N-terminally processed	1.45E+05	3.91E+04	8.66E+04	9.99E+04	6.00E+03	0.00E+00
SAFB	Q15424	Scaffold attachment factor B1	1.28E+05	1.00E+05	3.08E+04	3.62E+05	2.04E+05	3.93E+05
SAMHD1	Q9Y3Z3	Deoxynucleoside triphosphate triphosphohydrolase SAMHD1	4.37E+05	2.16E+05	3.07E+05	2.03E+06	1.62E+06	1.29E+06
SAMM50	Q9Y512	Sorting and assembly machinery component 50 homolog	3.34E+05	1.15E+05	2.02E+05	3.78E+04	0.00E+00	3.61E+04

SAP18	H7BZW6	Histone deacetylase complex subunit SAP18	7.65E+04	3.56E+04	9.14E+04	4.87E+04	8.55E+04	1.74E+05
SAP30BP	J3KS14	SAP30-binding protein	0.00E+00	2.95E+04	0.00E+00	7.75E+04	5.33E+04	1.52E+05
SAR1A	Q9NR31	GTP-binding protein SAR1a	2.64E+05	5.35E+03	2.28E+04	9.10E+04	8.41E+04	5.07E+04
SAR1B	D6RD69	GTP-binding protein SAR1b	9.88E+04	9.76E+03	5.00E+04	0.00E+00	1.34E+04	0.00E+00
SARS2	M0QWZ7	Serine--tRNA ligase, mitochondrial	4.54E+05	3.63E+05	1.31E+05	2.83E+04	0.00E+00	3.79E+04
SART1	O43290	U4/U6.U5 tri-snRNP-associated protein 1	1.14E+04	0.00E+00	1.36E+05	6.11E+05	4.12E+05	4.37E+05
SART3	Q15020-4	Squamous cell carcinoma antigen recognized by T-cells 3	1.68E+05	2.04E+04	0.00E+00	3.68E+05	1.09E+05	3.56E+05
SBF1	G5E933	Myotubularin-related protein 5	2.67E+05	5.56E+04	1.38E+05	1.80E+05	1.07E+05	1.78E+05
SCAF4	O95104-2	Splicing factor, arginine/serine-rich 15	5.20E+04	0.00E+00	6.16E+04	0.00E+00	6.14E+03	1.24E+05
SCFD1	Q8WVM8-2	Sec1 family domain-containing protein 1	2.51E+05	1.67E+05	6.53E+04	1.33E+04	0.00E+00	0.00E+00
SCP2	P22307-4	Non-specific lipid-transfer protein	8.55E+04	5.58E+04	6.80E+04	1.94E+04	0.00E+00	0.00E+00
SCYL1	E9PS17	N-terminal kinase-like protein	9.77E+03	0.00E+00	0.00E+00	4.53E+04	0.00E+00	0.00E+00
SDCBP	O00560	Syntenin-1	3.73E+05	1.81E+05	1.88E+05	1.79E+05	1.41E+05	1.06E+05
SDHB	P21912	Succinate dehydrogenase [ubiquinone] iron-sulfur subunit, mitochondrial	1.26E+06	9.86E+05	2.25E+05	3.77E+04	4.25E+04	1.34E+04
SDPR	O95810	Serum deprivation-response protein	2.67E+06	1.74E+06	1.68E+06	4.28E+06	1.93E+06	3.49E+06
SEC13	P55735-2	Protein SEC13 homolog	6.84E+04	1.41E+05	2.50E+04	2.83E+05	1.83E+05	3.29E+05
SEC14L2	O76054-5	SEC14-like protein 2;SEC14-like protein 3	6.47E+04	0.00E+00	5.59E+04	1.55E+04	0.00E+00	0.00E+00
SEC22B	O75396	Vesicle-trafficking protein SEC22b	3.88E+05	3.89E+05	6.33E+04	1.99E+05	7.52E+04	1.05E+04
SEC23A	F5H365	Protein transport protein Sec23A	1.59E+05	4.92E+04	1.77E+05	1.84E+05	2.58E+04	9.25E+04
SEC24C	G5EA31	Protein transport protein Sec24C	1.84E+05	1.12E+05	2.60E+05	2.59E+05	2.08E+05	2.88E+05
SEC31A	O94979-6	Protein transport protein Sec31A	9.71E+04	8.33E+03	2.26E+05	1.05E+05	7.37E+04	2.47E+05
SEL1L	Q9UBV2	Protein sel-1 homolog 1	8.05E+05	1.48E+05	1.72E+05	0.00E+00	7.44E+03	1.35E+04
SENP3	Q9H4L4	Sentrin-specific protease 3	4.46E+04	4.77E+04	0.00E+00	3.47E+04	1.84E+05	1.80E+05
Sep-02	Q15019-2	Septin-2	2.39E+06	1.18E+06	1.07E+06	2.34E+05	2.92E+05	2.76E+05
Sep-07	E7ES33	Septin-7	8.57E+06	5.36E+06	4.77E+06	1.19E+06	1.04E+06	1.05E+06
Sep-08	A6NMH6	Septin-8	3.09E+06	2.37E+06	2.81E+06	3.24E+05	1.85E+05	0.00E+00
Sep-09	Q9UHD8	Septin-9	2.94E+05	2.05E+05	2.09E+05	8.44E+04	2.50E+04	2.09E+05
Sep-10	E7EW69	Septin-10	2.86E+05	1.79E+05	1.75E+04	1.06E+05	4.04E+04	6.01E+04
Sep-11	D6RGI3	Septin-11	2.40E+06	7.22E+05	2.63E+06	1.42E+05	3.99E+03	6.51E+04
SERBP1	Q8NC51-4	Plasminogen activator inhibitor 1 RNA-binding protein	1.32E+04	0.00E+00	0.00E+00	9.74E+03	9.97E+03	0.00E+00
SERPINH1	P50454	Serpin H1	1.17E+05	4.89E+04	2.21E+05	8.35E+04	1.19E+05	1.15E+05
SET	A0A0C4DFV9	Protein SET;Protein SETSIP	1.75E+05	3.61E+04	2.87E+05	2.71E+05	0.00E+00	1.35E+05
SF1	Q15637-4	Splicing factor 1	5.65E+04	0.00E+00	0.00E+00	1.09E+05	6.13E+04	9.83E+04
SF3A1	Q15459	Splicing factor 3A subunit 1	2.69E+05	2.02E+05	2.72E+05	6.19E+04	9.82E+04	2.09E+05

SF3A3	Q12874	Splicing factor 3A subunit 3	5.48E+05	5.45E+05	5.67E+05	8.52E+03	1.07E+04	1.06E+05
SF3B1	O75533	Splicing factor 3B subunit 1	1.02E+06	8.56E+05	2.65E+05	1.35E+05	8.89E+04	3.72E+05
SF3B2	E9PPJ0	Splicing factor 3B subunit 2	7.09E+05	2.66E+05	2.05E+05	2.34E+05	1.63E+05	4.13E+05
SF3B3	Q15393	Splicing factor 3B subunit 3	2.70E+06	1.61E+06	7.92E+05	3.15E+05	5.28E+05	5.71E+05
SF3B4	Q15427	Splicing factor 3B subunit 4	3.45E+05	3.07E+05	5.43E+05	8.59E+04	1.26E+05	1.59E+05
SFPQ	P23246	Splicing factor, proline- and glutamine-rich	1.89E+06	7.23E+05	2.50E+05	1.09E+05	3.14E+05	7.98E+05
SGTA	O43765	Small glutamine-rich tetratricopeptide repeat-containing protein alpha	2.08E+05	2.02E+05	3.03E+05	5.34E+04	0.00E+00	0.00E+00
SGTB	D6RFW1	Small glutamine-rich tetratricopeptide repeat-containing protein beta	2.42E+05	3.05E+05	3.94E+05	2.34E+04	0.00E+00	6.23E+04
SH3GL1	Q99961	Endophilin-A2	3.19E+05	1.51E+05	9.11E+04	1.09E+04	0.00E+00	1.59E+04
SH3GLB1	A0A087WW40	Endophilin-B1	1.46E+05	3.01E+03	5.11E+04	9.58E+03	7.67E+03	1.13E+04
SH3GLB2	B7ZC38	Endophilin-B2	2.65E+05	9.06E+04	2.65E+05	0.00E+00	5.41E+04	7.69E+04
SH3KBP1	Q5JPT2	SH3 domain-containing kinase-binding protein 1	0.00E+00	5.17E+04	2.96E+03	2.44E+04	0.00E+00	3.04E+04
SHANK3	A0A0U1RR93	SH3 and multiple ankyrin repeat domains protein 3	2.09E+04	0.00E+00	7.19E+04	1.50E+05	9.49E+04	1.01E+05
SHROOM4	Q9ULL8-2	Protein Shroom4	0.00E+00	3.86E+03	0.00E+00	6.17E+04	5.26E+04	7.52E+04
SIAE	Q9HAT2	Sialate O-acetylesterase	2.56E+04	0.00E+00	0.00E+00	7.73E+04	3.06E+04	2.15E+04
SIN3A	Q96ST3	Paired amphipathic helix protein Sin3a	4.19E+04	0.00E+00	0.00E+00	8.13E+04	2.49E+05	3.50E+05
SIRT5	Q9NXA8-4	NAD-dependent protein deacylase sirtuin-5, mitochondrial	4.51E+04	5.13E+04	1.80E+05	1.56E+05	6.12E+04	3.27E+04
SKIV2L2	P42285	Superkiller viralicidic activity 2-like 2	3.78E+04	1.86E+04	7.02E+03	3.44E+04	0.00E+00	4.47E+04
SKP1	P63208	S-phase kinase-associated protein 1	5.73E+05	3.44E+05	6.08E+05	1.20E+05	1.30E+05	1.86E+05
SLC12A2	P55011	Solute carrier family 12 member 2	1.16E+06	7.05E+05	3.22E+05	1.81E+05	8.56E+04	2.45E+05
SLC12A7	A0A0G2JNW7	Solute carrier family 12 member 7	2.04E+04	3.36E+04	1.85E+04	3.52E+03	0.00E+00	3.65E+04
SLC25A1	P53007	Tricarboxylate transport protein, mitochondrial	2.62E+05	2.35E+04	3.19E+04	0.00E+00	5.50E+04	0.00E+00
SLC25A11	I3L1P8	Mitochondrial 2-oxoglutarate/malate carrier protein	2.79E+06	1.82E+06	6.29E+05	1.53E+05	1.54E+05	3.01E+05
SLC25A12	O75746	Calcium-binding mitochondrial carrier protein Aralar1	5.49E+06	2.60E+06	4.25E+06	3.75E+05	4.46E+05	2.97E+05
SLC25A13	Q9UJS0-2	Calcium-binding mitochondrial carrier protein Aralar2	3.17E+05	2.41E+05	2.71E+05	5.30E+04	0.00E+00	0.00E+00
SLC25A22	Q9H936	Mitochondrial glutamate carrier 1	6.73E+05	1.01E+06	1.35E+06	2.78E+04	3.89E+04	2.73E+05
SLC25A3	Q00325-2	Phosphate carrier protein, mitochondrial	6.81E+06	4.32E+06	7.33E+06	5.43E+05	7.35E+05	5.86E+05
SLC25A4	P12235	ADP/ATP translocase 1	1.09E+06	8.23E+05	5.06E+05	0.00E+00	3.04E+04	0.00E+00
SLC25A5	P05141	ADP/ATP translocase 2;ADP/ATP translocase 2, N-terminally processed	1.01E+06	8.31E+05	8.69E+05	2.60E+05	3.82E+05	1.60E+05
SLC25A6	P12236	ADP/ATP translocase 3;ADP/ATP translocase 3, N-terminally processed	9.14E+06	6.10E+06	7.54E+06	1.16E+06	7.68E+05	7.29E+05
SLC27A1	Q6PCB7	Long-chain fatty acid transport protein 1	2.18E+05	4.42E+03	8.95E+03	1.20E+05	4.76E+04	1.78E+04

SLC2A1	P11166	Solute carrier family 2, facilitated glucose transporter member 1	4.65E+06	3.59E+06	2.02E+06	1.60E+05	3.44E+05	5.58E+05
SLC3A2	F5GZS6	4F2 cell-surface antigen heavy chain	3.22E+06	2.63E+06	2.80E+06	6.64E+04	0.00E+00	1.28E+04
SLC9A1	P19634	Sodium/hydrogen exchanger 1	5.63E+05	1.46E+05	5.36E+05	3.57E+04	0.00E+00	0.00E+00
SLC9A3R1	O14745	Na(+)/H(+) exchange regulatory cofactor NHE-RF1	3.85E+05	3.68E+05	4.94E+05	0.00E+00	7.66E+03	0.00E+00
SLC9A3R2	Q15599	Na(+)/H(+) exchange regulatory cofactor NHE-RF2	4.44E+05	5.07E+05	2.07E+05	3.80E+04	0.00E+00	1.07E+05
SLK	Q9H2G2-2	STE20-like serine/threonine-protein kinase	1.46E+05	5.63E+04	2.40E+04	2.66E+05	1.06E+05	2.44E+05
SMARCA2	P51531	Probable global transcription activator SNF2L2	2.75E+05	1.79E+05	1.83E+05	3.51E+05	4.47E+05	7.08E+05
SMARCA4	Q9HBD4	Transcription activator BRG1	9.83E+04	2.59E+04	3.41E+04	1.31E+05	1.46E+05	1.36E+05
SMARCA5	O60264	SWI/SNF-related matrix-associated actin-dependent regulator of chromatin subfamily A member 5	4.12E+05	1.65E+05	0.00E+00	1.45E+06	1.57E+06	1.66E+06
SMARCB1	G5E975	SWI/SNF-related matrix-associated actin-dependent regulator of chromatin subfamily B member 1	9.90E+04	0.00E+00	1.03E+04	1.71E+05	1.26E+05	2.09E+05
SMARCC2	F8VXC8	SWI/SNF complex subunit SMARCC2	6.21E+05	0.00E+00	3.77E+05	7.87E+05	5.26E+05	6.99E+05
SMARCD1	Q96GM5-2	SWI/SNF-related matrix-associated actin-dependent regulator of chromatin subfamily D member 1	3.60E+04	0.00E+00	4.98E+04	1.12E+05	5.50E+04	2.49E+04
SMARCD3	Q6STE5-2	SWI/SNF-related matrix-associated actin-dependent regulator of chromatin subfamily D member 3;SWI/SNF-related matrix-associated actin-dependent regulator of chromatin subfamily D member 2	1.53E+04	0.00E+00	0.00E+00	6.43E+04	0.00E+00	8.96E+04
SMARCE1	J3QKS7	SWI/SNF-related matrix-associated actin-dependent regulator of chromatin subfamily E member 1	2.58E+05	4.54E+04	5.83E+04	3.47E+04	2.06E+05	8.34E+04
SMC1A	G8JLG1	Structural maintenance of chromosomes protein;Structural maintenance of chromosomes protein 1A	9.05E+05	4.88E+05	1.27E+05	2.40E+06	2.14E+06	2.72E+06
SMC3	Q9UQE7	Structural maintenance of chromosomes protein 3	1.05E+06	6.47E+05	3.84E+05	2.86E+06	2.39E+06	4.08E+06
SMCHD1	A6NHR9	Structural maintenance of chromosomes flexible hinge domain-containing protein 1	6.84E+04	0.00E+00	0.00E+00	3.18E+05	2.02E+05	3.42E+05
SMEK1	Q6IN85-4	Serine/threonine-protein phosphatase 4 regulatory subunit 3A	2.63E+04	0.00E+00	2.06E+04	0.00E+00	1.92E+04	2.50E+05
SMTN	A0A087WVP4	Smoothelin	3.13E+04	0.00E+00	0.00E+00	3.57E+05	6.09E+04	9.64E+04
SMU1	Q2TAY7	WD40 repeat-containing protein SMU1;WD40 repeat-containing protein SMU1, N-terminally processed	7.52E+04	5.59E+04	1.76E+04	8.46E+02	1.12E+04	0.00E+00
SND1	Q7KZF4	Staphylococcal nuclease domain-containing protein 1	1.12E+06	4.84E+05	4.45E+05	1.24E+05	1.81E+04	8.73E+04
SNRNP200	O75643	U5 small nuclear ribonucleoprotein 200 kDa helicase	1.45E+06	0.00E+00	7.82E+05	3.03E+05	4.13E+05	1.07E+06
SNRNP40	Q96DI7	U5 small nuclear ribonucleoprotein 40 kDa protein	3.15E+05	7.57E+04	6.93E+05	4.99E+04	0.00E+00	7.40E+04
SNRNP70	P08621-2	U1 small nuclear ribonucleoprotein 70 kDa	1.13E+06	1.30E+06	6.44E+05	1.51E+05	2.73E+05	5.22E+05
SNRPA1	P09661	U2 small nuclear ribonucleoprotein A	0.00E+00	1.53E+05	1.21E+05	8.75E+04	7.30E+04	1.59E+05

SNRPD2	P62316	Small nuclear ribonucleoprotein Sm D2	3.80E+04	1.47E+05	5.19E+04	9.25E+02	6.33E+04	2.46E+05
SNRPD3	P62318	Small nuclear ribonucleoprotein Sm D3	1.20E+05	3.17E+05	0.00E+00	0.00E+00	2.36E+04	0.00E+00
SNRPE	P62304	Small nuclear ribonucleoprotein E	1.14E+05	2.14E+05	1.02E+05	6.91E+04	0.00E+00	0.00E+00
SNRPF	P62306	Small nuclear ribonucleoprotein F	5.57E+05	4.90E+05	9.44E+04	1.28E+04	6.68E+04	2.76E+04
SNRPN	J3QLE5	Small nuclear ribonucleoprotein-associated proteins B and B;Small nuclear ribonucleoprotein-associated protein N	0.00E+00	1.48E+05	8.72E+03	0.00E+00	2.27E+04	5.05E+04
SNX1	Q13596	Sorting nexin-1	3.86E+05	2.99E+05	6.01E+05	3.45E+05	4.67E+04	1.02E+05
SNX17	Q15036	Sorting nexin-17	3.96E+04	0.00E+00	1.56E+05	2.90E+04	0.00E+00	1.12E+05
SNX2	O60749-2	Sorting nexin-2	6.57E+04	6.76E+04	5.25E+04	8.06E+04	0.00E+00	0.00E+00
SNX27	Q96L92	Sorting nexin-27	3.06E+05	8.68E+04	1.01E+05	2.74E+05	2.04E+05	3.86E+05
SNX3	O60493-4	Sorting nexin-3	5.40E+05	2.02E+05	3.39E+05	3.41E+05	6.50E+05	8.84E+05
SNX4	O95219	Sorting nexin-4	9.01E+04	7.15E+04	1.34E+05	9.99E+04	5.85E+04	1.50E+05
SNX9	Q9Y5X1	Sorting nexin-9	4.13E+04	8.17E+03	0.00E+00	1.67E+05	8.97E+04	2.03E+05
SOGA3	E9PJP2	Protein SOGA3	1.31E+05	2.29E+05	0.00E+00	4.55E+04	4.41E+04	1.78E+03
SON	P18583-2	Protein SON	4.64E+05	6.82E+05	7.27E+05	7.79E+05	6.19E+05	2.09E+06
SORBS2	O94875-10	Sorbin and SH3 domain-containing protein 2	1.71E+05	0.00E+00	0.00E+00	6.07E+05	1.52E+03	3.77E+05
SORBS3	O60504	Vinexin	2.98E+05	3.64E+05	0.00E+00	6.34E+05	4.24E+05	5.75E+05
SORT1	Q99523-2	Sortilin	6.18E+05	9.93E+04	1.83E+05	2.24E+04	2.29E+04	2.51E+04
SPG20	Q8N0X7	Spartin	4.14E+04	1.52E+05	1.97E+05	1.53E+05	1.89E+05	0.00E+00
SPTB	P11277	Spectrin beta chain, erythrocytic	1.28E+06	2.26E+06	9.45E+05	0.00E+00	5.13E+04	3.10E+03
SPTBN1	Q01082	Spectrin beta chain, non-erythrocytic 1	5.10E+07	3.75E+07	3.30E+07	9.93E+06	1.02E+07	1.33E+07
SPTBN2	O15020-2	Spectrin beta chain, non-erythrocytic 2	8.74E+05	1.11E+06	1.52E+06	1.97E+04	1.16E+05	2.73E+05
SPTBN4	C9JY79	Spectrin beta chain, non-erythrocytic 4	1.22E+05	7.72E+04	0.00E+00	1.53E+05	2.94E+05	4.03E+05
SQRDL	Q9Y6N5	Sulfide:quinone oxidoreductase, mitochondrial	8.55E+05	1.34E+06	1.77E+05	0.00E+00	3.39E+03	4.65E+03
SQSTM1	E9PFW8	Sequestosome-1	7.02E+04	6.05E+04	0.00E+00	0.00E+00	3.33E+04	1.51E+05
SRC	P12931	Proto-oncogene tyrosine-protein kinase Src	1.50E+05	0.00E+00	4.67E+04	1.87E+05	0.00E+00	3.26E+04
SRGAP2	A0A075B7B5	SLIT-ROBO Rho GTPase-activating protein 2;SLIT-ROBO Rho GTPase-activating protein 2B	2.02E+04	2.06E+04	5.66E+04	9.99E+04	7.58E+04	1.08E+05
SRP14	P37108	Signal recognition particle 14 kDa protein	1.92E+05	1.30E+05	1.35E+05	1.14E+05	7.43E+04	1.09E+05
SRP54	P61011	Signal recognition particle 54 kDa protein	3.10E+03	0.00E+00	0.00E+00	5.11E+03	0.00E+00	4.43E+03
SRP9	P49458	Signal recognition particle 9 kDa protein	1.89E+05	0.00E+00	0.00E+00	0.00E+00	1.62E+04	2.83E+04
SRPK2	P78362-2	SRSF protein kinase 2;SRSF protein kinase 2 N-terminal;SRSF protein kinase 2 C-terminal	2.03E+05	5.01E+04	1.04E+05	4.80E+04	1.12E+05	2.31E+05
SRPRB	Q9Y5M8	Signal recognition particle receptor subunit beta	3.60E+05	1.25E+05	2.07E+05	0.00E+00	9.69E+04	1.61E+04
SRRM1	A9Z1X7	Serine/arginine repetitive matrix protein 1	2.19E+04	0.00E+00	6.20E+04	6.64E+05	3.66E+05	4.16E+05

SRRM2	Q9UQ35	Serine/arginine repetitive matrix protein 2	1.80E+05	1.17E+04	1.33E+05	8.82E+05	5.70E+05	1.58E+06
SRRT	Q9BXP5-5	Serrate RNA effector molecule homolog	2.22E+04	0.00E+00	0.00E+00	7.82E+04	0.00E+00	7.15E+04
SRSF1	J3KTL2	Serine/arginine-rich splicing factor 1	3.95E+06	3.42E+06	1.21E+06	4.34E+06	3.90E+06	9.25E+06
SRSF10	Q5JRI1	Serine/arginine-rich splicing factor 10	5.19E+05	1.88E+05	1.87E+05	8.65E+05	9.54E+05	1.52E+06
SRSF3	P84103	Serine/arginine-rich splicing factor 3	1.62E+06	4.21E+05	9.02E+05	2.10E+06	3.77E+06	5.84E+06
SRSF4	A0A0D9SEM4	Serine/arginine-rich splicing factor 4	1.03E+06	1.31E+06	3.97E+05	3.14E+05	7.43E+05	9.89E+05
SRSF5	Q13243	Serine/arginine-rich splicing factor 5	4.12E+05	3.10E+05	0.00E+00	1.94E+05	3.38E+05	4.75E+05
SRSF6	Q13247	Serine/arginine-rich splicing factor 6	2.77E+05	0.00E+00	4.99E+03	2.21E+05	1.81E+05	4.16E+05
SRSF7	A0A0B4J1Z1	Serine/arginine-rich splicing factor 7	3.42E+05	1.16E+05	2.23E+05	4.52E+04	4.71E+05	6.14E+05
SRSF9	Q13242	Serine/arginine-rich splicing factor 9	2.67E+05	1.43E+05	1.06E+05	6.18E+05	1.29E+06	2.31E+06
SSB	P05455	Lupus La protein	1.68E+05	6.19E+04	1.26E+05	5.21E+05	1.34E+05	3.33E+05
SSBP1	Q04837	Single-stranded DNA-binding protein, mitochondrial	1.42E+06	8.56E+05	1.37E+06	2.22E+05	5.08E+05	6.96E+05
SSFA2	E9PHV5	Sperm-specific antigen 2	2.31E+04	5.98E+03	0.00E+00	6.84E+03	2.98E+04	2.21E+04
SSRP1	Q08945	FACT complex subunit SSRP1	6.30E+04	1.17E+05	3.16E+04	2.97E+04	1.86E+04	1.35E+05
ST13	P50502	Hsc70-interacting protein;Putative protein FAM10A4;Putative protein FAM10A5	8.39E+05	5.80E+05	1.33E+05	5.02E+04	0.00E+00	1.09E+05
STAM	Q92783	Signal transducing adapter molecule 1	1.04E+05	7.71E+04	1.41E+05	8.33E+04	1.30E+05	5.25E+04
STAT1	P42224	Signal transducer and activator of transcription 1-alpha/beta;Signal transducer and activator of transcription	3.85E+05	2.86E+05	1.33E+05	8.04E+05	1.16E+06	1.33E+06
STAT3	P40763-3	Signal transducer and activator of transcription 3;Signal transducer and activator of transcription	4.27E+05	1.28E+05	4.44E+05	1.25E+06	5.76E+05	9.76E+05
STAT5B	P51692	Signal transducer and activator of transcription 5B;Signal transducer and activator of transcription;Signal transducer and activator of transcription 5A	2.30E+04	0.00E+00	0.00E+00	6.47E+03	8.47E+04	2.07E+05
STAT6	A0A1W2PNW1	Signal transducer and activator of transcription 6	3.01E+04	4.70E+04	0.00E+00	0.00E+00	7.11E+04	1.47E+05
STIM1	G0XQ39	Stromal interaction molecule 1	8.00E+04	6.69E+04	2.08E+05	3.67E+03	0.00E+00	0.00E+00
STK39	Q9UEW8-2	STE20/SPS1-related proline-alanine-rich protein kinase	1.36E+06	6.68E+05	5.41E+05	1.14E+05	5.56E+05	3.39E+05
STOM	P27105	Erythrocyte band 7 integral membrane protein	1.28E+06	8.88E+05	4.75E+05	1.84E+05	1.45E+05	1.43E+05
STRAP	Q9Y3F4-2	Serine-threonine kinase receptor-associated protein	5.27E+05	6.75E+05	7.04E+05	4.00E+05	3.27E+05	5.54E+05
STRN	O43815	Striatin	4.26E+05	7.63E+04	2.27E+05	2.09E+04	0.00E+00	8.25E+04
STRN3	Q13033-2	Striatin-3	3.18E+04	1.65E+04	3.63E+04	4.38E+04	3.53E+04	9.84E+04
STUB1	H3BUD0	E3 ubiquitin-protein ligase CHIP	3.73E+04	2.17E+04	0.00E+00	2.41E+04	0.00E+00	3.50E+04
STX12	Q86Y82	Syntaxin-12	3.93E+05	3.24E+05	5.94E+05	7.90E+04	0.00E+00	5.93E+04
STX5	H7C3X5	Syntaxin-5	1.10E+05	4.93E+04	4.68E+04	1.52E+04	0.00E+00	3.64E+04
STXBP1	P61764-2	Syntaxin-binding protein 1	2.03E+07	9.09E+06	2.89E+07	3.50E+06	2.00E+06	2.92E+06

STXBP3	O00186	Syntaxin-binding protein 3	3.51E+05	1.51E+05	1.54E+05	3.55E+04	0.00E+00	0.00E+00
SUB1	P53999	Activated RNA polymerase II transcriptional coactivator p15	1.25E+05	7.52E+04	1.17E+04	4.35E+05	4.41E+05	3.49E+05
SUCLA2	Q9P2R7-2	Succinyl-CoA ligase [ADP-forming] subunit beta, mitochondrial	2.03E+06	1.84E+06	3.70E+06	4.69E+05	2.62E+05	4.01E+05
SUCLG1	P53597	Succinyl-CoA ligase [ADP/GDP-forming] subunit alpha, mitochondrial	1.97E+06	7.41E+05	2.82E+06	0.00E+00	7.50E+04	4.22E+04
SUCLG2	Q96I99	Succinyl-CoA ligase [GDP-forming] subunit beta, mitochondrial	9.39E+04	0.00E+00	0.00E+00	6.77E+04	5.93E+03	1.81E+04
SUGP2	M0R2Z9	SURP and G-patch domain-containing protein 2	5.12E+05	3.63E+05	6.34E+05	1.04E+06	1.97E+06	2.42E+06
SUGT1	Q9Y2Z0-2	Suppressor of G2 allele of SKP1 homolog	1.74E+05	3.27E+04	0.00E+00	1.89E+05	9.19E+04	2.58E+04
SULT1A1	H3BRY5	Sulfotransferase;Sulfotransferase 1A1;Sulfotransferase 1A2	3.92E+04	0.00E+00	0.00E+00	2.11E+04	1.21E+05	9.01E+04
SUMF2	J3KQJ1	Sulfatase-modifying factor 2	9.08E+04	2.14E+05	2.86E+04	1.91E+04	0.00E+00	1.14E+04
SUN1	H0Y742	SUN domain-containing protein 1	3.53E+05	8.13E+04	2.70E+05	1.02E+05	3.62E+05	2.08E+05
SUN2	Q9UH99	SUN domain-containing protein 2	3.62E+06	9.60E+05	1.28E+06	1.03E+06	1.03E+06	1.76E+06
SUPT16H	Q9Y5B9	FACT complex subunit SPT16	3.05E+05	1.03E+05	8.12E+04	7.57E+05	8.54E+05	1.34E+06
SUPT6H	Q7KZ85	Transcription elongation factor SPT6	1.84E+04	0.00E+00	0.00E+00	5.52E+05	9.40E+03	4.93E+04
SVIL	O95425-2	Supervillin	2.29E+05	0.00E+00	8.96E+03	3.71E+04	4.85E+04	8.31E+04
SYMPK	A0A087WUE9	Symplekin	3.12E+04	0.00E+00	3.56E+04	1.57E+05	0.00E+00	1.09E+05
SYNCRIP	O60506-2	Heterogeneous nuclear ribonucleoprotein Q	2.92E+06	4.25E+06	2.03E+06	1.86E+05	3.33E+05	8.06E+05
SYNE1	Q8NF91	Nesprin-1	1.11E+06	1.09E+06	1.73E+05	1.09E+05	2.29E+05	3.50E+05
SYNE2	G3V5X4	Nesprin-2	5.56E+04	4.19E+04	1.68E+04	1.64E+05	2.09E+05	3.44E+05
SYNE3	Q6ZMZ3-2	Nesprin-3	1.89E+05	6.32E+04	6.30E+04	0.00E+00	4.83E+04	7.12E+04
SYNJ1	J3KPK1	Synaptojanin-1	5.47E+05	4.11E+05	6.58E+05	4.97E+04	5.93E+04	0.00E+00
SYNM	O15061	Synemin	9.68E+04	1.62E+05	2.02E+05	1.40E+06	1.49E+06	7.63E+05
SYNPO	Q8N3V7-3	Synaptopodin	0.00E+00	1.08E+04	8.88E+04	4.60E+04	7.00E+04	1.77E+05
TACC1	O75410-7	Transforming acidic coiled-coil-containing protein 1	5.91E+04	6.81E+04	8.10E+04	4.99E+04	0.00E+00	5.01E+04
TAF15	A0A075B7D9	TATA-binding protein-associated factor 2N	4.64E+05	1.45E+05	2.20E+05	1.17E+05	4.65E+04	2.76E+04
TAGLN	Q01995	Transgelin	9.26E+04	0.00E+00	1.19E+05	9.46E+04	1.09E+04	4.78E+05
TAGLN2	X6RJP6	Transgelin-2	9.58E+04	6.72E+03	0.00E+00	3.53E+05	0.00E+00	2.22E+05
TAGLN3	Q9UI15	Transgelin-3	3.46E+05	4.32E+05	1.31E+05	3.83E+04	4.45E+04	1.71E+04
TAOK1	Q7L7X3	Serine/threonine-protein kinase TAO1	2.01E+04	3.08E+03	2.85E+04	0.00E+00	5.62E+03	4.66E+04
TAOK3	Q9H2K8	Serine/threonine-protein kinase TAO3	0.00E+00	1.38E+04	0.00E+00	8.22E+03	0.00E+00	0.00E+00
TBC1D15	Q8TC07-2	TBC1 domain family member 15	3.51E+05	7.74E+04	5.43E+04	2.67E+04	0.00E+00	7.50E+04
TBC1D4	O60343-2	TBC1 domain family member 4	7.26E+04	2.20E+04	9.75E+04	1.04E+05	4.32E+04	1.43E+05

TBC1D9B	Q66K14-2	TBC1 domain family member 9B	1.08E+05	0.00E+00	5.60E+04	4.20E+04	8.59E+04	7.76E+04
TBCB	Q99426-2	Tubulin-folding cofactor B	5.89E+05	2.63E+05	1.65E+05	6.30E+05	1.35E+05	2.99E+05
TBCD	J3KR97	Tubulin-specific chaperone D	1.86E+05	1.69E+04	2.88E+04	2.13E+05	3.05E+04	9.30E+04
TBK1	Q9UHD2	Serine/threonine-protein kinase TBK1	1.43E+05	4.69E+04	5.82E+03	2.37E+05	2.75E+05	3.38E+05
TBL1XR1	Q9BZK7	F-box-like/WD repeat-containing protein TBL1XR1	1.18E+04	1.64E+04	1.87E+04	3.47E+05	5.13E+05	5.42E+05
TBL2	E9PF19	Transducin beta-like protein 2	1.30E+05	7.56E+04	0.00E+00	3.70E+04	3.42E+04	5.00E+04
TBL3	Q12788	Transducin beta-like protein 3	6.87E+03	5.70E+04	1.04E+04	1.96E+05	1.92E+05	3.61E+05
TCEB1	E5RHG8	Transcription elongation factor B polypeptide 1	2.72E+05	1.48E+05	0.00E+00	1.62E+05	8.50E+04	1.24E+05
TCEB2	B8ZZU8	Transcription elongation factor B polypeptide 2	4.27E+04	7.87E+04	1.90E+05	0.00E+00	1.41E+04	0.00E+00
TCP1	P17987	T-complex protein 1 subunit alpha	1.18E+06	5.11E+05	8.36E+05	1.26E+05	1.05E+05	6.99E+04
TECR	Q9NZ01	Very-long-chain enoyl-CoA reductase	9.05E+05	8.56E+05	3.46E+05	5.74E+04	1.56E+04	4.25E+04
TERF2IP	Q9NYB0	Telomeric repeat-binding factor 2-interacting protein 1	3.19E+05	4.62E+03	1.42E+05	1.02E+06	3.08E+05	4.70E+05
TEX10	Q9NXF1	Testis-expressed sequence 10 protein	4.82E+05	2.35E+05	2.13E+05	1.81E+05	2.94E+05	1.67E+05
TF	P02787	Serotransferrin	3.15E+06	1.99E+06	1.50E+06	8.88E+04	0.00E+00	0.00E+00
TFAM	Q00059	Transcription factor A, mitochondrial	7.51E+04	1.11E+05	0.00E+00	4.12E+05	5.90E+05	1.04E+06
TFG	Q92734-4	Protein TFG	8.76E+03	9.77E+03	4.93E+03	2.42E+04	0.00E+00	5.37E+04
TGFB1I1	O43294-2	Transforming growth factor beta-1-induced transcript 1 protein	4.06E+05	3.61E+05	2.16E+05	5.77E+04	1.38E+05	6.56E+04
TGM2	P21980	Protein-glutamine gamma-glutamyltransferase 2	1.42E+06	4.84E+05	5.17E+05	3.20E+06	4.39E+06	5.00E+06
THOC2	Q8NI27	THO complex subunit 2	2.05E+05	1.46E+03	1.27E+05	1.39E+03	0.00E+00	1.61E+04
THOC6	Q86W42-3	THO complex subunit 6 homolog	8.25E+04	1.93E+04	9.60E+04	4.26E+03	3.03E+04	5.44E+03
THRAP3	Q9Y2W1	Thyroid hormone receptor-associated protein 3	1.01E+06	4.66E+05	6.52E+05	8.32E+05	3.09E+05	1.01E+06
TIAL1	Q01085	Nucleolysin TIAR;Nucleolysin TIA-1 isoform p40	7.35E+04	1.09E+04	4.75E+04	1.59E+05	9.94E+04	1.49E+05
TINAGL1	Q9GZM7	Tubulointerstitial nephritis antigen-like	2.35E+05	5.72E+04	8.31E+04	1.11E+07	7.18E+06	7.14E+06
TJP1	G3V1L9	Tight junction protein ZO-1	1.34E+06	2.45E+06	6.40E+05	5.62E+06	4.61E+06	6.04E+06
TJP2	A0A1B0GTW1	Tight junction protein ZO-2	1.47E+05	5.64E+04	0.00E+00	2.64E+05	3.92E+04	1.48E+05
TKT	P29401	Transketolase	3.50E+06	2.09E+06	5.36E+05	1.76E+05	3.33E+04	0.00E+00
TLN1	Q9Y490	Talin-1	8.81E+06	0.00E+00	4.43E+06	3.60E+06	1.22E+06	3.53E+06
TLN2	Q9Y4G6	Talin-2	2.63E+05	1.99E+05	4.29E+05	1.23E+05	1.24E+05	1.02E+05
TMCC3	G3V207	Transmembrane and coiled-coil domains protein 3	5.08E+04	5.72E+06	1.38E+06	0.00E+00	1.81E+06	7.89E+03
TMED9	Q9BVK6	Transmembrane emp24 domain-containing protein 9	5.11E+05	3.76E+04	2.88E+04	0.00E+00	1.77E+04	0.00E+00
TMEM43	Q9BTV4	Transmembrane protein 43	4.16E+05	1.46E+04	9.56E+04	3.46E+02	1.10E+05	0.00E+00
TMOD2	Q9NZR1	Tropomodulin-2	3.04E+05	3.95E+05	3.93E+05	2.46E+04	1.56E+05	2.51E+05
TMOD3	Q9NYL9	Tropomodulin-3	5.20E+04	7.02E+04	2.65E+04	7.00E+04	7.45E+03	7.94E+04
TMPO	P42166	Lamina-associated polypeptide 2, isoform	1.78E+06	1.45E+06	1.90E+06	7.38E+05	3.27E+05	1.29E+06

		alpha;Thymopoietin;Thymopentin						
TMX1	Q9H3N1	Thioredoxin-related transmembrane protein 1	4.99E+05	1.30E+05	2.23E+05	2.31E+04	0.00E+00	3.93E+04
TNC	J3QSU6	Tenascin	2.04E+05	9.30E+04	0.00E+00	1.61E+05	0.00E+00	0.00E+00
TNKS1BP1	Q9C0C2	182 kDa tankyrase-1-binding protein	0.00E+00	7.08E+04	1.82E+04	1.51E+05	9.85E+04	1.57E+05
TNPO1	Q92973-2	Transportin-1	7.28E+05	2.61E+05	1.69E+04	3.49E+04	1.04E+05	1.29E+05
TNPO2	O14787-2	Transportin-2	3.36E+05	2.13E+05	1.79E+05	0.00E+00	1.22E+04	5.75E+04
TNPO3	E9PFH4	Transportin-3	7.28E+04	0.00E+00	0.00E+00	0.00E+00	8.14E+03	2.14E+04
TNS2	Q63HR2-6	Tensin-2	1.70E+05	1.72E+05	7.05E+03	5.37E+05	0.00E+00	5.27E+05
TOLLIP	Q9H0E2	Toll-interacting protein	1.73E+05	5.15E+04	2.50E+05	1.32E+04	2.67E+04	4.13E+04
TOM1	O60784-4	Target of Myb protein 1	2.06E+04	9.21E+04	4.32E+04	2.22E+04	1.65E+04	7.73E+04
TOMM70A	O94826	Mitochondrial import receptor subunit TOM70	3.87E+06	2.24E+06	1.89E+06	9.85E+04	1.56E+05	1.29E+05
TOP1	P11387	DNA topoisomerase 1	3.04E+05	4.08E+05	6.46E+04	2.14E+05	2.70E+05	6.99E+05
TOP2B	Q02880-2	DNA topoisomerase 2-beta;DNA topoisomerase 2	2.56E+05	2.34E+05	1.76E+05	6.59E+05	1.30E+06	6.39E+05
TP53BP1	A6NNK5	Tumor suppressor p53-binding protein 1	1.20E+06	7.35E+05	3.15E+05	9.26E+05	1.50E+06	1.81E+06
TP53RK	Q96S44	TP53-regulating kinase	2.26E+04	0.00E+00	4.36E+04	1.18E+04	1.44E+05	1.33E+05
TPI1	P60174-1	Triosephosphate isomerase	1.05E+07	4.89E+06	8.24E+06	1.11E+04	3.41E+04	9.62E+03
TPM1	F5H7S3	Tropomyosin alpha-1 chain	4.64E+05	8.38E+05	5.53E+05	1.96E+05	7.08E+04	5.27E+04
TPM4	P67936	Tropomyosin alpha-4 chain	8.97E+05	9.25E+05	5.65E+05	7.57E+04	0.00E+00	2.51E+05
TPP1	O14773-2	Tripeptidyl-peptidase 1	2.06E+06	4.07E+05	2.41E+06	7.06E+05	6.53E+05	2.56E+05
TPR	P12270	Nucleoprotein TPR	8.20E+05	8.32E+05	9.28E+04	4.96E+04	5.39E+04	1.75E+05
TRA2A	Q13595-2	Transformer-2 protein homolog alpha	9.86E+05	3.35E+05	5.99E+05	9.32E+05	4.03E+05	8.76E+05
TRA2B	P62995-3	Transformer-2 protein homolog beta	1.34E+06	6.76E+05	6.85E+05	7.01E+05	6.61E+05	1.40E+06
TRAP1	Q12931-2	Heat shock protein 75 kDa, mitochondrial	9.76E+05	4.82E+05	1.48E+06	9.11E+04	5.94E+04	1.31E+05
TRIM25	Q14258	E3 ubiquitin/ISG15 ligase TRIM25	6.48E+03	1.21E+04	3.76E+04	1.68E+05	1.31E+05	2.02E+05
TRIM28	Q13263	Transcription intermediary factor 1-beta	0.00E+00	6.57E+05	1.03E+06	3.73E+06	2.73E+06	5.19E+06
TRIM3	A0A1W2PPI7	Tripartite motif-containing protein 3	3.77E+04	0.00E+00	0.00E+00	2.08E+05	2.77E+05	1.68E+05
TRIM33	Q9UPN9-2	E3 ubiquitin-protein ligase TRIM33	1.15E+05	1.39E+04	7.71E+04	1.94E+05	1.97E+05	2.37E+05
TRIO	E7EWP2	Triple functional domain protein	4.95E+04	4.97E+04	1.38E+05	1.11E+05	6.75E+04	5.39E+04
TRIOBP	Q9H2D6-5	TRIO and F-actin-binding protein	2.05E+04	0.00E+00	0.00E+00	8.68E+04	5.85E+04	1.62E+05
TRIP12	Q14669-4	E3 ubiquitin-protein ligase TRIP12	3.69E+03	1.53E+04	1.65E+04	3.91E+05	3.74E+05	4.06E+05
TSC2	H3BMQ0	Tuberin	8.52E+03	0.00E+00	0.00E+00	2.98E+03	0.00E+00	0.00E+00
TSC22D1	Q15714-2	TSC22 domain family protein 1	1.52E+04	0.00E+00	1.40E+05	1.82E+04	1.15E+04	2.76E+04
TSFM	P43897	Elongation factor Ts, mitochondrial;Elongation factor Ts	6.88E+04	1.25E+05	0.00E+00	8.77E+04	8.95E+04	2.44E+05
TSG101	F5H442	Tumor susceptibility gene 101 protein	0.00E+00	6.20E+04	0.00E+00	2.02E+04	0.00E+00	2.73E+04

TSSC1	A8MUM1	Protein TSSC1	1.71E+05	9.63E+04	5.59E+04	5.91E+04	0.00E+00	6.95E+04
TTC37	Q6PGP7	Tetratricopeptide repeat protein 37	1.69E+05	0.00E+00	1.78E+04	2.12E+05	8.06E+04	4.21E+04
TTLL12	Q14166	Tubulin--tyrosine ligase-like protein 12	5.25E+04	2.18E+04	2.28E+04	1.75E+05	2.05E+04	2.68E+04
TUBA1C	Q9BQE3	Tubulin alpha-1C chain	2.10E+05	0.00E+00	4.77E+04	4.63E+04	2.62E+05	3.42E+04
TUBA4A	P68366-2	Tubulin alpha-4A chain	1.71E+06	3.13E+06	4.03E+06	4.59E+05	1.12E+06	1.14E+06
TUBA8	C9J2C0	Tubulin alpha-8 chain	6.42E+04	8.85E+04	2.16E+05	0.00E+00	2.05E+05	1.63E+05
TUBB	P07437	Tubulin beta chain	6.34E+06	4.07E+06	5.97E+06	1.22E+07	1.63E+07	1.23E+07
TUBB2A	Q13885	Tubulin beta-2A chain	0.00E+00	9.56E+07	1.05E+08	1.39E+08	1.55E+08	1.54E+08
TUBB3	Q13509	Tubulin beta-3 chain	1.11E+07	5.76E+06	8.96E+06	1.07E+07	1.44E+07	1.32E+07
TUBB4A	P04350	Tubulin beta-4A chain	6.72E+07	2.68E+07	3.21E+07	4.83E+07	4.37E+07	3.86E+07
TUBB6	Q9BUF5	Tubulin beta-6 chain	0.00E+00	1.28E+05	2.71E+04	6.19E+05	6.78E+05	5.31E+05
TUBB8	Q3ZCM7	Tubulin beta-8 chain	9.70E+06	4.79E+06	5.27E+06	4.48E+06	4.63E+06	1.12E+06
TUFM	P49411	Elongation factor Tu, mitochondrial	9.16E+05	1.22E+06	6.59E+05	7.33E+06	6.53E+06	8.97E+06
TXN	P10599	Thioredoxin	1.42E+05	1.64E+04	2.37E+05	1.36E+04	0.00E+00	0.00E+00
TXNL1	K7ER96	Thioredoxin-like protein 1	2.24E+05	1.96E+05	4.48E+05	0.00E+00	5.69E+03	0.00E+00
TXNRD2	A0A096LPD9	Thioredoxin reductase 2, mitochondrial	4.53E+04	4.53E+04	2.01E+05	0.00E+00	2.01E+04	6.21E+04
U2AF1	P0DN76	Splicing factor U2AF 35 kDa subunit	2.08E+05	4.46E+04	3.94E+04	7.89E+04	1.02E+05	1.60E+05
U2AF2	K7ENG2	Splicing factor U2AF 65 kDa subunit	1.14E+05	1.71E+04	4.31E+04	5.99E+05	3.54E+05	5.41E+05
U2SURP	O15042-2	U2 snRNP-associated SURP motif-containing protein	4.60E+05	1.44E+05	6.52E+04	3.77E+05	1.78E+05	2.63E+05
UACA	H0YNH8	Uveal autoantigen with coiled-coil domains and ankyrin repeats	2.22E+04	0.00E+00	2.45E+04	3.64E+05	1.68E+05	2.33E+05
UBA1	P22314-2	Ubiquitin-like modifier-activating enzyme 1	2.74E+06	2.48E+06	2.62E+06	7.25E+05	2.25E+05	1.66E+05
UBA2	Q9UBT2	SUMO-activating enzyme subunit 2	3.04E+05	1.22E+05	5.65E+05	7.95E+05	5.09E+05	6.06E+05
UBA5	E7EQ61	Ubiquitin-like modifier-activating enzyme 5	3.84E+04	0.00E+00	0.00E+00	7.08E+04	0.00E+00	1.55E+05
UBA6	A0AVT1	Ubiquitin-like modifier-activating enzyme 6	1.27E+05	3.27E+04	4.14E+04	1.83E+05	0.00E+00	1.91E+04
UBAP2L	F8W726	Ubiquitin-associated protein 2-like	2.32E+03	0.00E+00	1.99E+04	1.82E+05	4.46E+04	1.77E+05
UBE2N	P61088	Ubiquitin-conjugating enzyme E2 N	6.09E+04	1.98E+05	3.89E+04	2.74E+04	0.00E+00	0.00E+00
UBE3C	Q15386	Ubiquitin-protein ligase E3C	5.50E+04	0.00E+00	7.60E+04	6.75E+03	0.00E+00	4.91E+04
UBE4A	B7Z7P0	Ubiquitin conjugation factor E4 A	1.82E+04	0.00E+00	0.00E+00	2.77E+04	0.00E+00	0.00E+00
UBQLN2	Q9UHD9	Ubiquilin-2	5.25E+04	2.59E+04	1.93E+05	7.97E+04	2.72E+04	0.00E+00
UBR4	Q5T4S7-3	E3 ubiquitin-protein ligase UBR4	9.28E+04	1.47E+05	3.73E+05	7.07E+04	6.91E+04	7.50E+04
UBR5	E7EMW7	E3 ubiquitin-protein ligase UBR5	5.72E+04	1.50E+04	0.00E+00	1.04E+05	1.23E+05	1.97E+05
UBR7	H0YJY4	Putative E3 ubiquitin-protein ligase UBR7	7.97E+03	0.00E+00	0.00E+00	1.08E+04	9.14E+03	5.31E+04
UBTF	E9PKP7	Nucleolar transcription factor 1	1.98E+05	5.70E+04	7.13E+04	1.54E+06	5.31E+05	6.67E+05
UBXN6	Q9BZV1	UBX domain-containing protein 6	2.55E+05	3.64E+04	2.14E+05	2.78E+04	4.24E+04	8.48E+04

UCHL1	P09936	Ubiquitin carboxyl-terminal hydrolase isozyme L1;Ubiquitin carboxyl-terminal hydrolase	6.39E+06	3.50E+06	5.88E+06	7.46E+04	0.00E+00	2.42E+04
UCHL5	Q5LJA5	Ubiquitin carboxyl-terminal hydrolase;Ubiquitin carboxyl-terminal hydrolase isozyme L5	4.22E+02	1.33E+04	1.25E+05	1.14E+05	6.24E+04	2.34E+05
UFL1	O94874	E3 UFM1-protein ligase 1	2.83E+05	1.73E+05	1.55E+05	2.33E+05	1.00E+04	6.41E+03
UPF1	Q92900-2	Regulator of nonsense transcripts 1	2.96E+05	1.21E+05	1.74E+05	8.33E+04	8.05E+04	1.46E+05
UQCRB	P14927	Cytochrome b-c1 complex subunit 7	7.48E+05	9.19E+05	5.32E+05	0.00E+00	3.51E+03	1.32E+04
UQCRC1	P31930	Cytochrome b-c1 complex subunit 1, mitochondrial	0.00E+00	5.69E+06	1.06E+07	1.37E+04	0.00E+00	4.34E+04
UQCRC2	P22695	Cytochrome b-c1 complex subunit 2, mitochondrial	2.29E+07	1.18E+07	1.63E+07	2.08E+05	1.68E+05	1.48E+05
UQCRFS1	P47985	Cytochrome b-c1 complex subunit Rieske, mitochondrial;Cytochrome b-c1 complex subunit 11;Putative cytochrome b-c1 complex subunit Rieske-like protein 1	1.68E+06	1.07E+06	1.08E+06	6.97E+03	0.00E+00	3.98E+03
USO1	O60763	General vesicular transport factor p115	2.87E+05	1.52E+05	2.90E+05	2.58E+05	2.00E+04	3.92E+04
USP10	Q14694	Ubiquitin carboxyl-terminal hydrolase 10	2.71E+04	0.00E+00	0.00E+00	1.09E+04	0.00E+00	8.07E+03
USP39	A0A087X1B2	U4/U6.U5 tri-snRNP-associated protein 2	1.11E+05	2.39E+04	1.11E+05	1.77E+03	0.00E+00	2.79E+04
USP47	Q96K76-2	Ubiquitin carboxyl-terminal hydrolase 47	3.01E+04	0.00E+00	0.00E+00	2.78E+04	0.00E+00	3.46E+03
USP48	Q86UV5	Ubiquitin carboxyl-terminal hydrolase 48	1.38E+05	1.43E+05	0.00E+00	1.06E+06	8.94E+05	1.16E+06
USP5	P45974-2	Ubiquitin carboxyl-terminal hydrolase 5	3.04E+06	6.05E+05	1.64E+06	0.00E+00	2.73E+06	3.41E+06
USP9X	Q93008-1	Probable ubiquitin carboxyl-terminal hydrolase FAF-X	2.27E+05	0.00E+00	1.10E+04	4.43E+04	0.00E+00	3.31E+04
UTP15	Q8TED0	U3 small nucleolar RNA-associated protein 15 homolog	2.34E+04	1.40E+06	0.00E+00	7.31E+05	1.07E+05	2.60E+05
UTP20	O75691	Small subunit processome component 20 homolog	1.51E+04	2.20E+04	1.21E+04	3.99E+04	2.61E+04	4.99E+04
UTP6	Q9NYH9	U3 small nucleolar RNA-associated protein 6 homolog	4.55E+03	4.25E+04	0.00E+00	0.00E+00	1.98E+05	4.80E+03
UTRN	P46939	Utrophin	6.91E+05	9.57E+05	3.99E+05	2.31E+06	2.68E+06	2.62E+06
VAPB	O95292	Vesicle-associated membrane protein-associated protein B/C	9.40E+05	1.03E+06	2.18E+05	2.20E+04	6.39E+03	0.00E+00
VARS	A0A140T936	Valine--tRNA ligase	4.39E+04	4.55E+04	1.68E+04	1.61E+04	1.68E+04	1.99E+04
VAT1	Q99536	Synaptic vesicle membrane protein VAT-1 homolog	1.51E+06	7.53E+05	6.53E+05	3.12E+04	0.00E+00	2.80E+04
VCL	P18206-2	Vinculin	5.28E+06	2.66E+06	1.56E+06	1.52E+06	2.79E+05	2.43E+06
VCP	P55072	Transitional endoplasmic reticulum ATPase	5.97E+06	2.42E+06	2.79E+06	1.31E+05	5.84E+04	2.37E+05
VCPIP1	Q96JH7	Deubiquitinating protein VCIP135	5.06E+04	4.39E+04	0.00E+00	2.16E+05	3.56E+05	2.97E+05
VDAC1	P21796	Voltage-dependent anion-selective channel protein 1	1.75E+07	1.34E+07	1.98E+07	8.29E+05	9.65E+05	9.10E+05
VDAC2	A0A0A0MR02	Voltage-dependent anion-selective channel protein 2	9.63E+06	7.49E+06	6.84E+06	2.08E+06	1.01E+06	1.60E+06
VPS11	B7Z879	Vacuolar protein sorting-associated protein 11 homolog	4.96E+04	1.19E+04	1.25E+04	1.41E+04	0.00E+00	7.39E+04
VPS16	Q9H269-2	Vacuolar protein sorting-associated protein 16 homolog	0.00E+00	1.01E+04	0.00E+00	7.67E+04	0.00E+00	0.00E+00
VPS26A	O75436	Vacuolar protein sorting-associated protein 26A	2.57E+05	7.59E+04	3.47E+04	6.29E+04	0.00E+00	4.54E+04

VPS29	F8VXU5	Vacuolar protein sorting-associated protein 29	4.68E+05	2.24E+05	7.44E+05	0.00E+00	3.36E+03	0.00E+00
VPS33B	Q9H267	Vacuolar protein sorting-associated protein 33B	5.93E+05	2.01E+05	6.94E+04	2.76E+04	2.86E+04	6.56E+04
VPS35	Q96QK1	Vacuolar protein sorting-associated protein 35	1.52E+06	3.19E+05	1.17E+06	2.29E+05	1.47E+05	4.31E+04
VPS39	Q96JC1-2	Vam6/Vps39-like protein	7.69E+04	0.00E+00	0.00E+00	4.48E+04	0.00E+00	9.81E+03
VPS45	Q9NRW7-2	Vacuolar protein sorting-associated protein 45	9.27E+04	4.53E+04	3.95E+04	0.00E+00	1.71E+04	0.00E+00
VPS4A	I3L4J1	Vacuolar protein sorting-associated protein 4A	5.16E+04	0.00E+00	0.00E+00	2.47E+05	2.97E+05	1.49E+05
VPS4B	O75351	Vacuolar protein sorting-associated protein 4B	4.89E+04	0.00E+00	0.00E+00	3.09E+04	1.22E+05	1.00E+05
VPS52	Q8N1B4-2	Vacuolar protein sorting-associated protein 52 homolog	9.27E+04	4.70E+04	3.13E+04	2.51E+04	6.95E+03	5.81E+04
VWA1	Q6PCB0	von Willebrand factor A domain-containing protein 1	5.28E+04	0.00E+00	0.00E+00	1.07E+06	1.82E+06	8.24E+05
VWF	P04275	von Willebrand factor;von Willebrand antigen 2	3.47E+06	0.00E+00	1.57E+06	6.55E+05	4.10E+05	6.24E+05
WASF2	Q9Y6W5	Wiskott-Aldrich syndrome protein family member 2	1.79E+05	0.00E+00	1.39E+05	8.20E+04	1.96E+04	5.75E+04
WDFY1	Q8IWB7	WD repeat and FYVE domain-containing protein 1	1.68E+05	6.79E+04	2.30E+05	1.01E+04	0.00E+00	0.00E+00
WDR1	O75083	WD repeat-containing protein 1	4.25E+05	3.37E+05	4.10E+05	2.22E+04	0.00E+00	5.22E+04
WDR18	U3KQC1	WD repeat-containing protein 18	0.00E+00	5.62E+04	3.05E+04	0.00E+00	1.17E+05	1.55E+04
WDR26	Q9H7D7	WD repeat-containing protein 26	6.38E+04	1.70E+04	7.11E+04	0.00E+00	5.65E+04	2.98E+04
WDR37	C9JGR9	WD repeat-containing protein 37	3.59E+04	1.21E+04	1.17E+05	1.92E+05	1.85E+05	0.00E+00
WDR43	Q15061	WD repeat-containing protein 43	2.80E+04	0.00E+00	0.00E+00	9.85E+03	3.14E+03	0.00E+00
WDR44	Q5JSH3-2	WD repeat-containing protein 44	1.98E+05	4.41E+04	1.46E+05	3.65E+03	0.00E+00	2.43E+04
WDR48	Q8TAF3-3	WD repeat-containing protein 48	9.69E+04	7.71E+03	1.28E+04	3.65E+04	2.07E+04	5.47E+04
WDR5	P61964	WD repeat-containing protein 5	0.00E+00	4.41E+04	0.00E+00	2.63E+05	0.00E+00	3.14E+05
WDR61	H0YN81	WD repeat-containing protein 61;WD repeat-containing protein 61, N-terminally processed	1.97E+05	3.59E+03	2.66E+05	9.86E+04	6.44E+04	9.29E+03
WDR77	Q9BQA1	Methylosome protein 50	6.28E+04	1.90E+04	0.00E+00	9.39E+03	4.08E+04	4.77E+04
WDR82	Q6UXN9	WD repeat-containing protein 82	1.08E+05	1.02E+04	5.29E+04	3.13E+04	2.27E+05	2.13E+05
WNK1	F5GWT4	Serine/threonine-protein kinase WNK1	1.25E+05	2.58E+04	7.87E+02	9.74E+04	6.80E+04	1.84E+05
XAB2	Q9HCS7	Pre-mRNA-splicing factor SYF1	1.10E+05	1.52E+05	8.90E+04	2.06E+05	2.49E+05	2.87E+05
XPO1	O14980	Exportin-1	4.88E+05	1.02E+05	3.04E+05	6.09E+05	1.18E+05	1.52E+05
XRCC5	P13010	X-ray repair cross-complementing protein 5	2.18E+06	1.03E+06	5.03E+05	2.23E+06	8.64E+05	1.44E+06
XRCC6	P12956	X-ray repair cross-complementing protein 6	2.74E+06	1.09E+06	1.51E+06	4.37E+06	1.12E+06	3.11E+06
YARS	P54577	Tyrosine--tRNA ligase, cytoplasmic;Tyrosine--tRNA ligase, cytoplasmic, N-terminally processed;Tyrosine--tRNA ligase	2.21E+05	1.94E+05	1.57E+05	0.00E+00	9.68E+03	0.00E+00
YBX1	H0Y449	Nuclease-sensitive element-binding protein 1	3.44E+05	3.69E+05	1.50E+05	1.58E+05	2.37E+05	4.48E+05
YLPM1	P49750	YLP motif-containing protein 1	1.01E+05	1.07E+04	4.33E+04	7.75E+05	3.14E+05	7.65E+05
YTHDF2	Q9Y5A9	YTH domain-containing family protein 2	1.67E+04	0.00E+00	0.00E+00	5.25E+04	1.07E+04	4.31E+04

YWHAB	P31946-2	14-3-3 protein beta/alpha;14-3-3 protein beta/alpha, N-terminally processed	4.17E+06	2.84E+06	2.01E+06	7.29E+05	6.99E+05	1.33E+06
YWHAE	P62258	14-3-3 protein epsilon	5.89E+06	4.89E+06	3.37E+06	3.82E+05	3.59E+05	2.46E+05
YWHAG	P61981	14-3-3 protein gamma;14-3-3 protein gamma, N-terminally processed	8.08E+06	5.35E+06	9.10E+06	2.25E+06	2.06E+06	4.45E+06
YWHAH	Q04917	14-3-3 protein eta	1.80E+06	1.42E+06	1.59E+06	5.46E+05	1.61E+05	7.40E+05
YWHAQ	P27348	14-3-3 protein theta	3.18E+06	1.73E+06	1.02E+06	4.41E+05	2.84E+05	3.35E+05
YWHAZ	P63104	14-3-3 protein zeta/delta	1.12E+07	6.68E+06	7.33E+06	2.09E+06	2.27E+06	1.90E+06
YY1	P25490	Transcriptional repressor protein YY1	4.75E+04	6.83E+03	0.00E+00	1.95E+05	3.89E+05	4.15E+05
ZC3H11A	E9PQ61	Zinc finger CCCH domain-containing protein 11A	4.53E+04	1.30E+04	2.34E+04	1.60E+05	3.50E+05	3.08E+05
ZC3H14	Q6PJT7	Zinc finger CCCH domain-containing protein 14	1.41E+05	0.00E+00	0.00E+00	3.09E+05	3.93E+04	2.19E+05
ZC3H18	E7ERS3	Zinc finger CCCH domain-containing protein 18	2.81E+04	5.80E+04	5.11E+04	2.32E+05	2.05E+05	4.71E+05
ZC3HAV1	Q7Z2W4	Zinc finger CCCH-type antiviral protein 1	3.09E+04	3.29E+04	0.00E+00	1.30E+05	2.07E+05	1.10E+05
ZFR	Q96KR1	Zinc finger RNA-binding protein	7.94E+05	5.10E+05	4.12E+04	4.21E+05	2.94E+05	7.33E+05
ZMYM2	Q9UBW7	Zinc finger MYM-type protein 2	1.05E+04	0.00E+00	0.00E+00	1.40E+05	2.99E+04	1.83E+05
ZNF326	Q5BKZ1	DBIRD complex subunit ZNF326	8.73E+04	7.52E+03	1.05E+05	6.69E+05	7.03E+05	8.77E+05
ZNF512	G3XAG1	Zinc finger protein 512	9.29E+04	4.26E+04	1.05E+05	1.29E+06	6.58E+05	5.18E+05
ZNF638	Q14966-3	Zinc finger protein 638	1.07E+05	1.54E+04	1.65E+05	1.11E+06	1.09E+06	1.35E+06
ZW10	O43264	Centromere/kinetochore protein zw10 homolog	5.96E+04	1.87E+04	8.84E+04	0.00E+00	2.23E+04	5.07E+03
ZYX	Q15942-2	Zyxin	1.07E+05	0.00E+00	0.00E+00	8.75E+04	0.00E+00	0.00E+00
