# Supplementary material for: Global proteomic analysis of extracellular matrix in mouse and human brain highlights relevance to cerebrovascular disease
Source: J Cereb Blood Flow Metab. 2021 Mar 17;41(9):2423–38. doi: 10.1177/0271678X211004307 (PMC8392779; doi:10.1177/0271678X211004307)
Supplement: sj-pdf-7-jcb-10.1177_0271678X211004307 - Supplemental material for Global proteomic analysis of extracellular matrix in mouse and human brain highlights relevance to cerebrovascular disease [file sj-pdf-7-jcb-10.1177_0271678X211004307.pdf]

Supplementary Tables S4, S5.

Table S4. ECM proteins present in MatrisomeDB, which are identified in ECM-enriched and cellular fractions of mouse (top) and human (bottom) HpH-fractionated samples. ECM proteins are quantified in either both fractions in at least 2 biological replicates or exclusively in the ECM-enriched or cellular fraction.

Identified mouse ECM proteins

Category Matrisome DB	protein	fraction	mean cellular	mean ECM	fc	gene	Protein.names	Unique peptides	cellular 1	cellular 2	cellular 3	ECM1	ECM2	ECM3
ECM-affiliated Proteins	A0A0N4SW89	cellular	94104	-	-	Anxa4	Annexin;Annexin A4	6	79836	74445	128030	0	0	0
Proteoglycans	Q5DTP6	cellular	29591	-	-	Lppr3	Lipid phosphate phosphatase-related protein type 3	2	28231	30787	29754	0	0	0
ECM Regulators	Q8VCK2	cellular	249050	-	-	Ctsd	Cathepsin D	2	374080	275710	97359	0	0	0
ECM Glycoproteins	Q5NCU3	cellular	68042	-	-	Sparc	SPARC	2	17025	0	119060	0	0	0
ECM-affiliated Proteins	Q922A2	cellular	141889	-	-	Anxa7	Annexin;Annexin A7	3	86368	205500	133800	0	0	0
ECM-affiliated Proteins	A0A3B0INZ4	cellular	104708	-	-	C1qc	Complement C1q subcomponent subunit C	2	67558	79747	166820	0	0	0
ECM-affiliated Proteins	Q3UBX7	cellular	47660	-	-	C1qa	Complement C1q subcomponent subunit A	3	57111	61215	24653	0	0	0
ECM-affiliated Proteins	Q3US82	cellular	200717	-	-	C1qb	Complement C1q subcomponent subunit B	2	186770	187180	228200	0	0	0
ECM Regulators	A4FUT9	cellular	1926533	-	-	Adam22	Disintegrin and metalloproteinase domain-containing protein 22	13	1989300	2291400	1498900	0	0	0
ECM Glycoproteins	A8C1T7	cellular	229868	-	-	Creld1	Cysteine-rich with EGF-like domain protein 1	5	27773	460190	201640	0	0	0

ECM Regulators	B0V2N1	cellular	666063	-	-	Ptprs	Receptor-type tyrosine-protein phosphatase S	12	893530	680650	424010	0	12424	0
ECM-affiliated Proteins	B1AQR8	cellular	58125	-	-	Lgals9	Galectin;Galectin-9	2	23655	94803	55916	0	0	0
Secreted Factors	Q61191	cellular	49020	-	-	Hcfc1	Host cell factor 1	3	24142	22819	100100	0	0	0
ECM-affiliated Proteins	B1AY86	cellular	12705	-	-	Plxdc2	Plexin domain-containing protein 2	2	2249.9	23161	0	0	0	0
Proteoglycans	B2RRU7	cellular	79529	-	-	Hapln2	Hyaluronan and proteoglycan link protein 2	3	61287	142170	35131	0	0	0
ECM-affiliated Proteins	B2RWT9	cellular	135812	-	-	Plxnb1	Plexin-B1	7	155660	172860	78916	0	0	0
ECM-affiliated Proteins	B2RXS4	cellular	87574	-	-	Plxnb2	Plexin-B2	5	124670	76233	61819	0	0	0
ECM-affiliated Proteins	B9EJ29	cellular	574133	-	-	Plxna4	Plexin-A4	8	617370	704000	401030	0	0	0
Proteoglycans	E9QMK3	cellular	161467	-	-	Vcan	Versican core protein	9	141350	219520	123530	0	0	0
Proteoglycans	E9PZ16	cellular	777627	-	-	Hspg2	Basement membrane-specific heparan sulfate proteoglycan core protein;Endorepellin;LG3 peptide	67	519350	1193300	620230	0	0	0
ECM Regulators	Q3TTT2	cellular	34292	-	-	P4ha1	Prolyl 4-hydroxylase subunit alpha-1	2	20717	68831	13327	0	0	0
Proteoglycans	Q6PAN0	cellular	139555	-	-	Spock2	Testican-2	2	159490	0	119620	0	28241	0
ECM-affiliated Proteins	Q99JX6	cellular	1239600	-	-	Anxa6	Annexin;Annexin A6	13	1399700	1483200	835900	0	0	0
ECM Glycoproteins	F8WJ51	cellular	57282	-	-	Ntng1	Netrin-G1	2	77875	42224	51746	0	0	0
ECM-affiliated Proteins	Q8BJC1	cellular	20999	-	-	Sema4d	Semaphorin-4D	2	21479	28774	12745	0	0	0

ECM Regulators	O35598	cellular	400537	-	-	Adam10	Disintegrin and metalloproteinase domain-containing protein 10	8	302660	479090	419860	0	0	0
ECM-affiliated Proteins	Q8C1X9	cellular	24912	-	-	Anxa3	Annexin;Annexin A3	4	36077	15061	23597	0	0	0
ECM Regulators	Q3TVS6	cellular	258153	-	-	Ctsb	Cathepsin B;Cathepsin B light chain;Cathepsin B heavy chain	6	360660	225170	188630	0	0	0
ECM Glycoproteins	Q3TDU5	cellular	18994	-	-	Mfge8	Lactadherin	2	25361	0	12627	0	0	0
Secreted Factors	Q3UKQ5	cellular	212099	-	-	M6pr	Cation-dependent mannose-6-phosphate receptor	2	81908	236400	317990	0	0	0
ECM-affiliated Proteins	P48036	cellular	1061507	-	-	Anxa5	Annexin A5	7	1166100	698120	1320300	0	0	0
Secreted Factors	Q3UY00	cellular	172990	-	-	S100b	Protein S100;Protein S100-B	2	225850	178790	114330	0	0	0
Secreted Factors	P61329	cellular	53750	-	-	Fgf12	Fibroblast growth factor 12	3	65952	70829	24470	0	0	0
ECM-affiliated Proteins	P70206	cellular	47665	-	-	Plxna1	Plexin-A1	3	37392	31385	74218	0	0	0
ECM-affiliated Proteins	P70207	cellular	201952	-	-	Plxna2	Plexin-A2	3	13130	13986	578740	0	0	0
ECM Glycoproteins	Q3UYK7	cellular	270067	-	-	Sparcl1	SPARC-like protein 1	9	279740	220670	309790	0	0	0
ECM-affiliated Proteins	P97384	cellular	59260	-	-	Anxa11	Annexin A11;Annexin	8	33249	36392	108140	0	0	0
ECM Regulators	Q3TZE2	cellular	199732	-	-	Adam11	Disintegrin and metalloproteinase domain-containing protein 11	5	280040	278060	41095	0	0	0
ECM Glycoproteins	Q3U0Z1	cellular	8952	-	-	Coch	Cochlin	2	0	13394	4509.8	0	0	0
ECM-affiliated Proteins	Q9D0F3	cellular	298860	-	-	Lman1	Protein ERGIC-53	5	71129	367930	457520	0	0	0

ECM-affiliated Proteins	Q6ZQ83	cellular	66864	-	-	Plxnd1	Plexin-D1	2	60872	72855	0	0	0	0
Proteoglycans	Q61361	cellular	998133	-	-	Bcan	Brevican core protein	12	666600	1665000	662800	0	0	0
ECM-affiliated Proteins	Q68FM6	cellular	65200	-	-	Elfn2	Protein phosphatase 1 regulatory subunit 29	2	52479	92572	50549	0	0	0
ECM-affiliated Proteins	Q71M36	cellular	1555350	-	-	Cspg5	Chondroitin sulfate proteoglycan 5	7	1674500	0	1436200	0	0	0
Proteoglycans	Q80WM4	cellular	392383	-	-	Hapln4	Hyaluronan and proteoglycan link protein 4	4	438730	430320	308100	47395	0	0
ECM-affiliated Proteins	Q8VED9	cellular	116138	-	-	Lgalsl	Galectin-related protein	2	150090	82187	0	0	0	0
ECM-affiliated Proteins	Q8VHY0	cellular	538950	-	-	Cspg4	Chondroitin sulfate proteoglycan 4	13	618170	698130	300550	0	0	0
ECM-affiliated Proteins	Q9QUR8	cellular	215923	-	-	Sema7a	Semaphorin-7A	8	214200	235150	198420	0	0	0
ECM-affiliated Proteins	Q9QZC2	cellular	41683	-	-	Plxnc1	Plexin-C1	4	57191	26175	0	0	0	0
ECM Regulators	Q9R118	cellular	12490	-	-	Htra1	Serine protease HTRA1	3	13073	11908	0	0	0	17255
ECM Regulators	Q9R1V7	cellular	908273	-	-	Adam23	Disintegrin and metalloproteinase domain-containing protein 23	8	1001500	924020	799300	0	0	0
Collagens	A2AJY2	ECM	-	144933	-	Col15a1	Collagen alpha-1(XV) chain;Restin	4	0	0	0	72650	189080	173070
ECM Glycoproteins	A2ATM9	ECM	-	98145	-	Lamc3	Laminin subunit gamma-3	4	0	0	0	7955.6	197040	89439
ECM Glycoproteins	Q3UGU1	ECM	-	48598	-	Ltbp4	Latent-transforming growth factor beta-binding protein 4	2	5758.3	0	0	0	21795	75401
ECM Glycoproteins	B7ZN28	ECM	-	21703	-	Papln	Papilin	5	0	0	0	12835	48479	3794.1

Collagens	P39061	ECM	-	1165870	-	Col18a1	Collagen alpha-1(XVIII) chain;Endostatin	8	0	0	45671	891540	1637700	968370
Collagens	P02463	ECM	-	480907	-	Col4a1	Collagen alpha-1(IV) chain;Arresten	2	0	12682	0	285130	651490	506100
Collagens	P11087	ECM	-	4709967	-	Col1a1	Collagen alpha-1(I) chain	3	0	0	0	4009200	4069600	6051100
Proteoglycans	Q7TMW3	ECM	-	38459	-	Bgn	Biglycan	2	0	0	0	8789.1	85793	20795
Proteoglycans	Q3UKR1	ECM	-	51761	-	Dcn	Decorin	2	0	0	0	60701	14675	79906
Collagens	Q3TX57	ECM	-	586640	-	Col1a2	Collagen alpha-2(I) chain	6	0	0	0	577420	413370	769130
ECM Glycoproteins	Q3USG5	ECM	-	44009	-	Emilin1	EMILIN-1	5	0	0	0	34871	39933	57224
ECM Glycoproteins	Q3UPR9	ECM	-	5801	-	Sbspon	Somatomedin-B and thrombospondin type-1 domain-containing protein	3	0	16499	0	0	4723	6878.6
ECM Glycoproteins	Q61789	ECM	-	78840	-	Lama3	Laminin subunit alpha-3	5	0	0	0	35208	148760	52551
ECM Glycoproteins	H3BJ97	both	36946	5281833	142.96	Tinagl1	Tubulointerstitial nephritis antigen-like	9	16329	32925	61583	5066900	5992800	4785800
ECM Glycoproteins	Q61001	both	108403	11271833	103.98	Lama5	Laminin subunit alpha-5	37	15705	218520	90984	9310000	14817000	9688500
ECM Glycoproteins	Q60675	both	52132	4352133	83.48	Lama2	Laminin subunit alpha-2	43	26672	55546	74177	5255400	7801000	0
Collagens	B2RQQ8	both	112686	8654000	76.8	Col4a2	Collagen alpha-2(IV) chain;Canstatin	7	0	69943	155430	7659600	11294000	7008400
ECM Glycoproteins	Q8R2Z5	both	107114	2493967	23.28	Vwa1	von Willebrand factor A domain-containing protein 1	8	5472.1	126700	189170	1922300	3378100	2181500
Collagens	Q02788	both	21499	478250	22.24	Col6a2	Collagen alpha-2(VI) chain	4	7173.5	0	35825	435110	483520	516120
ECM Regulators	Q8BJD1	both	41457	916447	22.11	Itih5	Inter-alpha-trypsin inhibitor heavy chain H5	13	0	23678	59236	534230	1253600	961510
ECM Glycoproteins	Q3UHL7	both	94378	1378000	14.6	Lamb1	Laminin subunit beta-1	14	90102	71172	121860	781700	2191500	1160800
ECM	Q8R5G0	both	384850	522723	13.58	Nid2	Nidogen-2	20	196390	413590	544570	497970	562430	507770

Glycoproteins				3								0	0	0
ECM Glycoproteins	F8VQJ3	both	2458333	31721667	12.9	Lamc1	Laminin subunit gamma-1	37	1803900	3023900	2547200	29602000	39765000	25798000
Collagens	Q04857	both	100555	1264700	12.58	Col6a1	Collagen alpha-1(VI) chain	8	29975	205140	66550	1284700	1286600	1222800
ECM Glycoproteins	Q61292	both	2737433	31704667	11.58	Lamb2	Laminin subunit beta-2	39	2137400	3244200	2830700	29616000	36384000	29114000
ECM Glycoproteins	P97927	both	179650	1974967	10.99	Lama4	Laminin subunit alpha-4	20	210330	208680	119940	3052000	0	2872900
ECM Glycoproteins	P10493	both	1266713	10351600	8.17	Nid1	Nidogen-1	25	937940	1588200	1274000	9139500	13321000	8594300
ECM Glycoproteins	P19137	both	498740	3315867	6.65	Lama1	Laminin subunit alpha-1	36	344780	687580	463860	1985200	5118000	2844400
Collagens	J3QQ16	both	391547	2056667	5.25	Col6a3	collagen, type VI, alpha 3	36	236410	419860	518370	2268200	2127300	1774500
ECM Regulators	Q7M754	both	66860	295723	4.42	Gm5409	predicted pseudogene 5409	2	0	9980.8	123740	403050	239100	245020
ECM Regulators	P21981	both	194477	600477	3.09	Tgm2	Protein-glutamine gamma-glutamyltransferase 2	9	209930	254530	118970	869090	464550	467790
ECM Glycoproteins	M0QWP1	both	3190967	7758167	2.43	Agrn	Agrin;Agrin N-terminal 110 kDa subunit;Agrin C-terminal 110 kDa subunit;Agrin C-terminal 90 kDa fragment;Agrin C-terminal 22 kDa fragment	36	2039500	4220100	3313300	4950100	8207400	10117000
ECM-affiliated Proteins	Q8BNB0	both	38268	61587	1.61	Gpc5	Glypican-5;Secreted glypican-5	4	22154	69552	23097	32453	142400	9906.8
ECM-affiliated Proteins	O35988	both	41864	57483	1.37	Sdc4	Syndecan-4;Syndecan	3	39506	44222	0	62100	52906	57442
Collagens	E9PX70	both	212160	214950	1.01	Col12a1	Collagen alpha-1(XII) chain	13	113030	327590	195860	184800	188470	271580
ECM Glycoproteins	P29788	both	33870	28489	0.84	Vtn	Vitronectin	2	0	38236	29503	31781	18115	35570

ECM Glycoproteins	E9Q557	both	71698	42926	0.6	Dsp	Desmoplakin	5	116300	0	27097	115530	0	13247
ECM-affiliated Proteins	P51655	both	49053	26348	0.54	Gpc4	Glypican-4;Secreted glypican-4	2	0	57803	40303	55707	0	23338
ECM Glycoproteins	Q2I0J8	both	338003	144829	0.43	Vwf	von Willebrand factor;von Willebrand antigen 2	14	291270	282710	440030	48727	224790	160970
ECM-affiliated Proteins	Q3U379	both	23494	9153	0.39	Gpc1	Glypican-1;Secreted glypican-1	5	19449	27540	0	12318	15142	0
ECM Glycoproteins	A6H6E2	both	134912	50714	0.38	Mmrn2	Multimerin-2	4	124750	183620	96366	25323	79894	46926
Proteoglycans	Q543S0	both	71051	25333	0.36	Prelp	Prolargin	5	40624	109440	63088	60749	0	15249
ECM Regulators	Q3TWG9	both	453673	107901	0.24	Serpinh1	Serpin H1	9	319750	548760	492510	85301	218430	19972
ECM-affiliated Proteins	C0HKD9	both	426787	81873	0.19	Mfap1	Microfibrillar-associated protein 1	9	274920	635890	369550	15142	133370	97106
Proteoglycans	Q9QUP5	both	204695	20698	0.1	Hapln1	Hyaluronan and proteoglycan link protein 1	5	270070	246770	97245	33347	0	28746
ECM Glycoproteins	A0A1Y7VJW9	both	260477	17437	0.07	Fbln5	Fibulin-5	6	201690	388140	191600	20205	32107	0
Secreted Factors	Q9Z315	both	1336033	84079	0.06	Sart1	U4/U6.U5 tri-snRNP-associated protein 1	19	857800	1713900	1436400	92122	109870	50244
Proteoglycans	A0A0R4IZX5	both	305207	12289	0.04	Ncan	Neurocan core protein	4	373290	247490	294840	25792	11076	0
ECM-affiliated Proteins	Q9CZI7	both	465053	8052	0.02	Anxa2	Annexin;Annexin A2	13	202970	317880	874310	17436	6718.7	0
ECM Glycoproteins	Q8K406	both	695647	16263	0.02	Lgi3	Leucine-rich repeat LGI family member 3	7	755710	1040400	290830	35238	0	13551
ECM Glycoproteins	Q8BYI9	both	4633467	84889	0.02	Tnr	Tenascin-R	26	4851300	5890300	3158800	77963	90740	85963
ECM Glycoprotei	A0A0G2JGB7	both	1310950	11350	0.01	Lgi1	Leucine-rich glioma-inactivated protein 1	7	1490600	1586700	855550	24658	0	9393.5

ns														

Identified human ECM proteins

Category MatrisomeDB	fraction	mean cellular	mean ECM	fc	protein	gene	Protein.names	Unique peptides	cellular1	cellular 2	cellular 3	ECM1	ECM2	ECM3
ECM Glycoproteins	cellular	473407	-	-	A0A0D9SFU4	LGI1	Leucine-rich glioma-inactivated protein 1	6	670880	267510	481830	0	0	7.34E+03
Proteoglycans	cellular	76044	-	-	D6RC59	HAPLN1	Hyaluronan and proteoglycan link protein 1	2	15176	63705	149250	0	0	0.00E+00
ECM-affiliated Proteins	cellular	50161	-	-	E5RK69	ANXA6	Annexin	2	30985	43593	75905	0	0	0.00E+00
ECM Regulators	cellular	189605	-	-	E7EWD3	ADAM23	Disintegrin and metalloproteinase domain-containing protein 23	7	297890	44234	226690	0	0	0.00E+00
ECM Glycoproteins	cellular	37485	-	-	E9PRU1	EFEMP2	EGF-containing fibulin-like extracellular matrix protein 2	2	0	26857	48113	0	0	0.00E+00
ECM Regulators	cellular	109018	-	-	P34949	MPI	Mannose-6-phosphate isomerase	5	101180	45865	180010	0	0	0.00E+00
ECM Regulators	cellular	148337	-	-	F8WAD8	ADAM22	Disintegrin and metalloproteinase domain-containing protein 22	8	221090	102780	121140	0	0	0.00E+00
ECM Glycoproteins	cellular	85924	-	-	H0YJW4	COCH	Cochlin	3	165600	74161	18012	32717	0	0.00E+00
ECM Regulators	cellular	33498	-	-	H7C2S8	PLOD3	Procollagen-lysine,2-oxoglutarate 5-dioxygenase 3	2	44917	22080	0	0	0	0.00E+00
ECM Glycoproteins	cellular	148564	-	-	J3QSU6	TNC	Tenascin	8	204120	93009	0	161390	0	0.00E+00
Proteoglycans	cellular	101559	-	-	O14594	NCAN	Neurocan core protein	3	73991	24157	206530	0	95957	0.00E+00

ECM Regulators	cellular	423673	-	-	O14672	ADAM10	Disintegrin and metalloproteinase domain-containing protein 10	10	869270	278900	122850	2476.3	0	0.00E+00
ECM-affiliated Proteins	cellular	42484	-	-	O15031	PLXNB2	Plexin-B2	4	13984	51912	61557	0	0	0.00E+00
ECM-affiliated Proteins	cellular	62886	-	-	O43157	PLXNB1	Plexin-B1	4	102220	33931	52506	0	0	0.00E+00
Secreted Factors	cellular	1832100	-	-	P04271	S100B	Protein S100-B	3	2750100	1308200	1438000	0	0	0.00E+00
Secreted Factors	cellular	61070	-	-	P05230	FGF1	Fibroblast growth factor 1	2	83018	0	39123	0	0	0.00E+00
ECM Regulators	cellular	122384	-	-	P07858	CTSB	Cathepsin B;Cathepsin B light chain;Cathepsin B heavy chain	3	174440	19142	173570	0	0	0.00E+00
ECM Glycoproteins	cellular	7675	-	-	P07996-2	THBS1	Thrombospondin-1	2	0	1352.7	13998	0	0	0.00E+00
ECM-affiliated Proteins	cellular	377290	-	-	P09525	ANXA4	Annexin A4;Annexin	9	510000	313460	308410	0	0	0.00E+00
ECM-affiliated Proteins	cellular	134240	-	-	P12429	ANXA3	Annexin A3;Annexin	7	162040	156240	84440	0	0	0.00E+00
Secreted Factors	cellular	368690	-	-	P23297	S100A1	Protein S100-A1;Protein S100	3	358480	379830	367760	0	0	0.00E+00
ECM Regulators	cellular	53653	-	-	P30740	SERPINB1	Leukocyte elastase inhibitor	4	100960	37239	22759	0	0	0.00E+00
ECM-affiliated Proteins	cellular	331250	-	-	P49257	LMAN1	Protein ERGIC-53	6	366690	295810	0	0	0	4.77E+04
ECM-affiliated Proteins	cellular	573910	-	-	P50995	ANXA11	Annexin A11	8	537740	558390	625600	0	0	0.00E+00
ECM Glycoproteins	cellular	1517780	-	-	Q14515-2	SPARCL1	SPARC-like protein 1	18	1916600	910540	1726200	0	0	0.00E+00
Secreted Factors	cellular	54491	-	-	Q5JVF3-3	PCID2	PCI domain-containing protein 2	2	72458	0	36524	0	0	2.48E+04

ECM-affiliated Proteins	cellular	73236	-	-	Q5KU26	COLEC12	Collectin-12	5	84790	65825	69093	0	0	0.00E+00
ECM-affiliated Proteins	cellular	71807	-	-	Q6UVK1	CSPG4	Chondroitin sulfate proteoglycan 4	4	41127	158660	15635	0	0	0.00E+00
ECM-affiliated Proteins	cellular	40120	-	-	Q86T13	CLEC14A	C-type lectin domain family 14 member A	4	5742.6	31977	82640	0	0	0.00E+00
Secreted Factors	cellular	83258	-	-	Q96FN4	CPNE2	Copine-2	2	136440	13896	99439	0	0	0.00E+00
Proteoglycans	cellular	700557	-	-	Q96GW7	BCAN	Brevican core protein	11	977420	602360	521890	0	51131	0.00E+00
Secreted Factors	cellular	92813	-	-	Q99584	S100A13	Protein S100-A13	2	84696	100790	92952	0	0	0.00E+00
ECM-affiliated Proteins	cellular	245667	-	-	Q9HCM2	PLXNA4	Plexin-A4	6	382250	115730	239020	0	0	0.00E+00
ECM-affiliated Proteins	ECMdepl	32391	-	-	Q9UIW2	PLXNA1	Plexin-A1	4	5689.6	71469	20014	0	0	0.00E+00
Collagens	ECM	-	6603300	-	A0A087WTA8	COL1A2	Collagen alpha-2(I) chain	4	0	0	0	13249000	3551600	3.01E+06
Collagens	ECM	-	67759	-	F5H3Q5	COL4A6	Collagen alpha-6(IV) chain	2	0	0	0	55276	80242	0.00E+00
Collagens	ECM	-	430947	-	A0A087X0K0	COL15A1	Collagen alpha-1(XV) chain;Restin;Restin-2;Restin-3;Restin-4	5	0	0	0	262340	582940	4.48E+05
ECM Glycoproteins	ECM	-	145240	-	A0A0A0MSA0	LAMA3	Laminin subunit alpha-3	10	0	0	0	304940	45124	8.57E+04
Secreted Factors	ECM	-	55696	-	A0A0A6YYC7	ZFP91-CNTF	E3 ubiquitin-protein ligase ZFP91	2	0	0	0	108070	0	3.32E+03
Secreted Factors	ECM	-	4022	-	A0A140T9I6	EGFL8	Epidermal growth factor-like protein 8	2	0	0	0	2176.9	0	5.87E+03
ECM Glycoproteins	ECM	-	767245	-	A0A140T9S5	VWA7	von Willebrand factor A domain-containing protein 7	2	393320	0	0	0	1136100	3.98E+05
ECM Glycoproteins	ECM	-	29501	-	F5GZ78	PXN	Paxillin	4	0	27567	0	72497	8764	7.24E+03

Secreted Factors	ECM	-	120735	-	A6NEM2	HCFC1	Host cell factor 1	4	0	0	26277	61775	113850	1.87E+05
ECM Glycoproteins	ECM	-	23298	-	C9JD84	LTBP1	Latent-transforming growth factor beta-binding protein 1	2	0	0	0	44186	12645	1.31E+04
ECM Glycoproteins	ECM	-	730970	-	P15502-9	ELN	Elastin	6	0	0	0	1019600	0	4.42E+05
ECM Glycoproteins	ECM	-	21627	-	H0YBU5	MATN2	Matrilin-2	2	0	0	0	15127	32098	1.77E+04
ECM Glycoproteins	ECM	-	1238667	-	Q6PCB0	VWA1	von Willebrand factor A domain-containing protein 1	7	52754	0	0	1069000	1822700	8.24E+05
ECM-affiliated Proteins	ECM	-	217910	-	O00182-3	LGALS9	Galectin-9	3	0	20755	0	284650	211250	1.58E+05
Secreted Factors	ECM	-	126040	-	O60934	NBN	Nibrin	3	0	17518	0	167830	76061	1.34E+05
ECM Glycoproteins	ECM	-	109385	-	O95428-6	PAPLN	Papilin	5	0	0	0	54395	115070	1.59E+05
Collagens	ECM	-	2268623	-	P02452	COL1A1	Collagen alpha-1(I) chain	3	0	0	0	4786500	1409700	6.10E+05
Collagens	ECM	-	2491133	-	P02462-2	COL4A1	Collagen alpha-1(IV) chain;Arresten	7	0	0	0	2774000	2905300	1.79E+06
Proteoglycans	ECM	-	375420	-	P07585	DCN	Decorin	4	1507.7	0	0	484440	266400	0.00E+00
ECM Glycoproteins	ECM	-	354515	-	P35555	FBN1	Fibrillin-1	15	430230	0	0	880310	158150	2.51E+04
ECM Regulators	ECM	-	317600	-	P35625	TIMP3	Metalloproteinase inhibitor 3	3	0	0	0	425640	0	2.10E+05
Secreted Factors	ECM	-	84936	-	Q13188	STK3	Serine/threonine-protein kinase 3;Serine/threonine-protein kinase 3 36kDa subunit;Serine/threonine-protein kinase 3 20kDa subunit;Serine/threonine-protein kinase	4	0	0	0	88619	81254	0.00E+00

							4;Serine/threonine-protein kinase 4 37kDa subunit;Serine/threonine-protein kinase 4 18kDa subunit						
ECM Glycoproteins	ECM	-	35144	-	Q15582	TGFBI	Transforming growth factor-beta-induced protein ig-h3	2	0	0	0	20761	49528	0.00E+00
Secreted Factors	ECM	-	100742	-	Q5UIP0-2	RIF1	Telomere-associated protein RIF1	8	109210	0	0	151350	38976	1.12E+05
ECM Glycoproteins	ECM	-	149223	-	Q8IVN8	SBSPON	Somatomedin-B and thrombospondin type-1 domain-containing protein	2	0	0	0	279820	83074	8.48E+04
ECM Regulators	ECM	-	57481	-	Q92743	HTRA1	Serine protease HTRA1	2	3953	0	0	0	7411.5	1.08E+05
Secreted Factors	ECM	-	26471	-	Q9Y5Z7	HCFC2	Host cell factor 2	2	0	0	0	13035	44666	2.17E+04
ECM Glycoproteins	ECM	-	967467	-	Q9Y6C2	EMILIN1	EMILIN-1	11	3749.8	0	0	706790	1383900	8.12E+05
ECM Glycoproteins	ECM	-	822823	-	Q9Y6N6	LAMC3	Laminin subunit gamma-3	7	0	0	0	1059900	1086600	3.22E+05
Collagens	both	26806	20233000	754.78	P08572	COL4A2	Collagen alpha-2(IV) chain;Canstatin	11	25177	19880	35362	22779000	25260000	1.27E+07
ECM Glycoproteins	both	7635	1816567	237.92	P25391	LAMA1	Laminin subunit alpha-1	22	0	10425	4845.6	1295700	2557900	1.60E+06
Proteoglycans	both	123256	22413000	181.84	P98160	HSPG2	Basement membrane-specific heparan sulfate proteoglycan core protein;Endorepellin;LG3 peptide	72	162220	163610	43938	22855000	28351000	1.60E+07
ECM Glycoproteins	both	119963	14827000	123.6	O15230	LAMA5	Laminin subunit alpha-5	51	218990	137280	3619.6	16047000	16387000	1.20E+07
Collagens	both	25697	2049733	79.77	P39060-2	COL18A1	Collagen alpha-1(XVIII) chain;Endostatin	11	32552	27294	17245	2196500	1856000	2.10E+06
ECM Glycoproteins	both	125011	8472267	67.77	Q9GZM7	TINAGL1	Tubulointerstitial nephritis antigen-like	14	234640	57248	83144	11098000	7176200	7.14E+06

ECM Glycoproteins	both	11582	702673	60.67	P07942	LAMB1	Laminin subunit beta-1	12	0	11744	11421	717200	762550	6.28E+05
ECM Glycoproteins	both	176958	8653033	48.9	Q14112-2	NID2	Nidogen-2	29	306730	175570	48575	9491700	8957700	7.51E+06
ECM Glycoproteins	both	350620	13351000	38.08	P14543-2	NID1	Nidogen-1	23	559660	284300	207900	13160000	16284000	1.06E+07
ECM Glycoproteins	both	297235	9137300	30.74	P02751	FN1	Fibronectin;Anastellin;Ugl-Y1;Ugl-Y2;Ugl-Y3	37	416910	456110	18686	5460900	10897000	1.11E+07
ECM Glycoproteins	both	1740100	43886000	25.22	P55268	LAMB2	Laminin subunit beta-2	44	2174500	2009700	1036100	45397000	47414000	3.88E+07
ECM Glycoproteins	both	60504	1522300	25.16	A0A0A0MTC7	LAMA4	Laminin subunit alpha-4	19	65733	55274	0	1683400	1766100	1.12E+06
ECM Glycoproteins	both	585730	12930833	22.08	A0A087WYF1	LAMA2	Laminin subunit alpha-2	59	895790	614050	247350	14711000	14613000	9.47E+06
ECM Glycoproteins	both	1860333	31533667	16.95	P11047	LAMC1	Laminin subunit gamma-1	38	2017500	2242500	1321000	31923000	38998000	2.37E+07
ECM Glycoproteins	both	908897	7158567	7.88	C9JEU5	FGG	Fibrinogen gamma chain	15	912850	905930	907910	3794800	11637000	6.04E+06
Collagens	both	1230970	9499633	7.72	E7ENL6	COL6A3	Collagen alpha-3(VI) chain	50	2247000	214940	0	11913000	11479000	5.11E+06
ECM Glycoproteins	both	68076	449285	6.6	A0A140T9C0	TNXB	Tenascin-X	22	109510	26642	0	385330	883370	7.92E+04
Collagens	both	150258	922960	6.14	D6RGG3	COL12A1	Collagen alpha-1(XII) chain	20	339160	86285	25330	1183900	893470	6.92E+05
ECM Regulators	both	807530	4198000	5.2	P21980	TGM2	Protein-glutamine gamma-glutamyltransferase 2	15	1422000	483800	516790	3202300	4390600	5.00E+06
Secreted Factors	both	49287	221430	4.49	Q15773	MLF2	Myeloid leukemia factor 2	2	90694	7879.7	0	191050	216290	2.57E+05
ECM Regulators	both	85030	348857	4.1	G5E9D8	ITIH5	Inter-alpha-trypsin inhibitor heavy chain H5	10	105020	65040	0	484530	371480	1.91E+05

ECM Glycoproteins	both	24246	94333	3.89	P15924	DSP	Desmoplakin	11	0	18268	30223	115490	66470	1.01E+05
Collagens	both	922310	2971967	3.22	A0A087X0S5	COL6A1	Collagen alpha-1(VI) chain	13	2299200	291350	176380	4368200	3130100	1.42E+06
ECM Glycoproteins	both	3859400	12071200	3.13	O00468-6	AGRN	Agrin;Agrin N-terminal 110 kDa subunit;Agrin C-terminal 110 kDa subunit;Agrin C-terminal 90 kDa fragment;Agrin C-terminal 22 kDa fragment	45	4965400	4866500	1746300	14433000	12727000	9.05E+06
Secreted Factors	both	635717	1931490	3.04	P11498	PC	Pyruvate carboxylase, mitochondrial	21	1051700	363170	492280	2855300	621070	2.32E+06
ECM Glycoproteins	both	766283	2124560	2.77	P02671	FGA	Fibrinogen alpha chain;Fibrinopeptide A;Fibrinogen alpha chain	11	577760	1473200	247890	568480	4379000	1.43E+06
ECM Glycoproteins	both	225330	408360	1.81	Q9H8L6	MMRN2	Multimerin-2	5	339350	160310	176330	325740	395510	5.04E+05
Proteoglycans	both	226096	390398	1.73	P21810	BGN	Biglycan	7	629400	35199	13689	918580	197720	5.49E+04
ECM-affiliated Proteins	both	551327	779673	1.41	P04083	ANXA1	Annexin A1;Annexin	8	711690	409850	532440	1407100	165170	7.67E+05
ECM Glycoproteins	both	70376	81508	1.16	Q12805-2	EFEMP1	EGF-containing fibulin-like extracellular matrix protein 1	5	107350	0	33401	93660	63116	8.77E+04
Secreted Factors	both	95772	92414	0.96	P05109	S100A8	Protein S100-A8;Protein S100-A8, N-terminally processed	2	93751	70716	122850	67994	95368	1.14E+05
ECM Regulators	both	130662	109025	0.83	P00750-3	PLAT	Tissue-type plasminogen activator;Tissue-type plasminogen activator chain A;Tissue-type plasminogen activator chain B	4	212870	48453	0	143730	95875	8.75E+04
ECM Regulators	both	128923	105615	0.82	P50454	SERPINH1	Serpin H1	7	116660	48879	221230	83486	118860	1.15E+05
ECM	both	585210	368180	0.63	P40123	CAP2	Adenylyl cyclase-	9	1148600	493480	113550	392590	193110	5.19E+0

Regulators							associated protein 2;Adenylyl cyclase-associated protein						5
Proteoglycans	both	866390	545763	0.63	Q9GZV7	HAPLN2	Hyaluronan and proteoglycan link protein 2	6	1413100	860090	325980	617850	513140	5.06E+05
ECM Regulators	both	2509400	1094113	0.44	A0A1B0GW44	CTSD	Cathepsin D;Cathepsin D light chain;Cathepsin D heavy chain	7	3735500	1484300	2308400	1594100	1377200	3.11E+05
Collagens	both	597840	166825	0.28	Q05707-2	COL14A1	Collagen alpha-1(XIV) chain	16	859100	336580	0	298640	175730	2.61E+04
ECM Glycoproteins	both	277091	66300	0.24	Q8N145	LGI3	Leucine-rich repeat LGI family member 3	7	446820	296730	87722	88286	87554	2.31E+04
ECM Glycoproteins	both	2520200	563017	0.22	P04275	VWF	von Willebrand factor;von Willebrand antigen 2	46	3467500	0	1572900	654940	410430	6.24E+05
Proteoglycans	both	321084	61287	0.19	P51888	PRELP	Prolargin	7	611980	30188	0	59710	67017	5.71E+04
ECM Glycoproteins	both	82742	12133	0.15	P55081	MFAP1	Microfibrillar-associated protein 1	5	83602	81882	0	35366	0	1.03E+03
Secreted Factors	both	9079700	1011980	0.11	Q03252	LMNB2	Lamin-B2	25	12311000	8763300	6164800	1452600	840660	7.43E+05
ECM Glycoproteins	both	3259967	308200	0.09	Q92752	TNR	Tenascin-R	20	4094400	3689800	1995700	361400	418230	1.45E+05
Proteoglycans	both	1265550	81204	0.06	E9PF17	VCAN	Versican core protein	10	0	1356700	1174400	131620	68749	4.32E+04
ECM Glycoproteins	both	152305	2585	0.02	O43854-2	EDIL3	EGF-like repeat and discoidin I-like domain-containing protein 3	2	306050	67127	83737	2098.5	0	5.66E+03
ECM-affiliated Proteins	both	1514107	31383	0.02	P08758	ANXA5	Annexin A5;Annexin	10	2149000	1478600	914720	0	44318	4.98E+04
ECM-affiliated Proteins	both	2001633	47170	0.02	P09382	LGALS1	Galectin-1	5	1903400	2107000	1994500	0	88439	5.31E+04
Secreted Factors	both	404233	7616	0.02	P14174	MIF	Macrophage migration inhibitory factor	3	590130	493140	129430	21990	0	8.57E+02
ECM-affiliated	both	289137	6155	0.02	P20073-2	ANXA7	Annexin A7	6	327620	259600	280190	0	6109.5	1.24E+04

Proteins															
ECM-affiliated Proteins	both	2756933	26578	0.01	P07355-2	ANXA2	Annexin A2;Annexin	5	3871500	2009500	2389800	0	24509	5.52E+04
ECM-affiliated Proteins	both	6370433	36020	0.01	P08133	ANXA6	Annexin A6;Annexin	9	8807300	5777500	4526500	55295	37926	1.48E+04

Table S5. Proteins used for differential expression analysis comparing ECM-enriched and cellular fractions in mouse and human. The Table lists all proteins depicted on volcano plots, including those quantified in at least 2 biological replicates in both fractions and those quantified exclusively in the ECM-enriched fraction. The differential expression was done using empirical Bayes method for two group comparison implemented in eb.fit function of limma package. The plotted FDR-adjusted for multiple testing P values of the moderated t-test are shown in q.mod column; log2 fold changes are in log2fc column. Main ECM proteins (MatrisomeDB) are marked by bold; yellow colour indicates key basement membrane proteins.

Mouse proteins quantified with HpH fractionation

log2fc	q.mod	Significant	ECM mean	cellular mean	gene	description	Unique peptides	cellular1	cellular2	cellular3	ECM1	ECM2	ECM3	ECM marker
-3.13657	1.35E-05	TRUE	94721.33	783000	Nfasc		3	970730	715240	663030	127340	107280	49544	FALSE
-2.12882	4.78E-05	TRUE	330926.7	1439433	Ncam1	Neural cell adhesion molecule 1	5	1312400	1816700	1189200	433170	272640	286970	FALSE
2.996351	0.000124	TRUE	549540	47751.33	Lrrc7	Leucine-rich repeat-containing protein 7	13	101350	41904	0	752440	565900	330280	FALSE
-5.18034	1.15E-07	TRUE	374550	13536333	Atp1a3	Sodium/potassium-transporting ATPase subunit alpha-3	25	14179000	12580000	13850000	349820	426680	347150	FALSE
-2.59337	0.000115	TRUE	467753.3	2851900	NA		2	4560200	2140500	1855000	256670	661210	485380	FALSE
-0.55912	0.020986	FALSE	521146.7	776810	Zfml	Zinc finger protein 638	25	600300	771800	958330	482970	606750	473720	FALSE
1.849506	0.002671	TRUE	187104.7	31183.33	Shank2	SH3 and multiple ankyrin repeat domains protein 2	14	0	35263	58287	292940	190030	78344	FALSE
1.430606	0.001102	TRUE	373696.7	92163.33	Iqsec1	IQ motif and SEC7 domain-containing protein 1	11	0	117110	159380	421950	411440	287700	FALSE
1.514732	0.00033	TRUE	7165200	2550233	Thy1	Thy-1 membrane glycoprotein	5	3046800	1821400	2782500	8219900	6155700	7120000	FALSE
-1.33137	0.000418	TRUE	644670	1611200	Actn4	Alpha-actinin-4	14	1449900	1603500	1780200	774010	551240	608760	FALSE
-2.05618	0.00049	TRUE	179910	694296.7	Atp6v1b2	V-type proton ATPase subunit B, brain isoform	20	708640	439010	935240	311410	120750	107570	FALSE
1.220872	0.004246	TRUE	1882080	748883.3	Myl6	Myosin light polypeptide 6	5	608700	663870	974080	1711000	988740	2946500	FALSE
-1.25629	0.000689	TRUE	183630	430906.7	Sorbs1	Sorbin and SH3 domain-containing protein 1	16	461310	418620	412790	236100	155260	159530	FALSE

-0.43272	0.083291	FALSE	68847.67	100100.3	Syt7	Synaptotagmin-7	8	220910	79391	0	135500	71043	0	FALSE
2.531059	1.11E-05	TRUE	10040733	1717033	Rbmxl1	RNA binding motif protein, X-linked-like-1;RNA-binding motif protein, X chromosome;RNA-binding motif protein, X chromosome, N-terminally processed	6	1463200	1717900	1970000	12547000	9794400	7780800	FALSE
2.84902	7.44E-06	TRUE	1905000	264556.7	Dlg2	Disks large homolog 2	15	329810	257180	206680	2392500	1799100	1523400	FALSE
3.140395	3.96E-06	TRUE	13357333	1560433	Myh11		2	1116500	2070800	1494000	13520000	12230000	14322000	FALSE
0.456203	0.045227	FALSE	132706.7	92352.67	Tjp2	Tight junction protein ZO-2	11	76094	99374	101590	136410	181660	80050	FALSE
-1.78418	7.18E-05	TRUE	1152097	3928700	Actn1		2	3583600	4077200	4125300	1384500	949090	1122700	FALSE
-2.2264	0.000355	TRUE	49053	338590	Sh3gl2	Endophilin-A1	8	451890	341760	222120	49228	97931	0	FALSE
-2.67619	8.06E-06	TRUE	324320	2039867	Map1a	Microtubule-associated protein 1A;MAP1A heavy chain;MAP1 light chain LC2	47	2077300	2252400	1789900	239300	390040	343620	FALSE
-0.24228	0.08969	FALSE	31954	37736.33	Pkp4	Plakophilin-4	10	54638	0	58571	0	51115	44747	FALSE
-1.6744	0.000258	TRUE	338383.3	1039667	Srcin1	SRC kinase signaling inhibitor 1	29	1032700	1136700	949600	441230	218720	355200	FALSE
0.40055	0.039862	FALSE	467996.7	350106.7	Baiap2	Brain-specific angiogenesis inhibitor 1-associated protein 2	13	327000	345370	377950	575370	446310	382310	FALSE
-1.77162	0.000191	TRUE	901850	2975967	Map1b	Microtubule-associated protein 1B;MAP1B heavy chain;MAP1 light chain LC1	54	3409200	2955400	2563300	857210	1235900	612440	FALSE
2.268522	3.01E-05	TRUE	121053.3	25697.67	Cgnl1	Cingulin-like protein 1	11	19325	34271	23497	133620	126790	102750	FALSE
4.873234	1.77E-07	TRUE	2.1E+08	7288633	Hist2h4	Histone H4	10	5425900	8897600	7542400	1.97E+08	2.24E+08	2.07E+08	FALSE
0.123467	0.115864	FALSE	1139047	1073180	Zfr	Zinc finger RNA-binding protein	29	672440	1315400	1231700	1161800	1371400	883940	FALSE

1.226231	0.041851	TRUE	2793367	2871133	H2afv	Histone H2A;Histone H2A.V;Histone H2A.Z	3	628240	347360	7637800	2396900	3203600	2779600	FALSE
-0.84753	0.007012	FALSE	149196.7	262946.7	9430020K01Rik	Junctional protein associated with coronary artery disease	12	219520	314980	254340	204640	124010	118940	FALSE
2.279861	0.000187	TRUE	253933.3	50598	Dlgap4	Disks large-associated protein 4	11	44653	73353	33788	408720	188980	164100	FALSE
0.205804	0.084742	FALSE	1081550	926246.7	Tjp1	Tight junction protein ZO-1	27	882300	995210	901230	860250	1292700	1091700	FALSE
-0.2828	0.070542	FALSE	24150.67	44096	Cep170b	Centrosomal protein of 170 kDa protein B	3	45922	45181	41185	37444	35008	0	FALSE
-0.39273	0.066575	FALSE	94428.67	119075.7			9	79767	159000	118460	50822	137350	95114	FALSE
-0.10436	0.117824	FALSE	1668200	1816100	Hnrnpdl		2	1350400	1732000	2365900	1979600	1318400	1706600	FALSE
0.726403	0.010324	FALSE	278800	171990	Pogz	Pogo transposable element with ZNF domain	14	152400	135900	227670	248960	315880	271560	FALSE
-1.55966	0.001102	TRUE	234536.7	667873.3	Ylpm1	YLP motif-containing protein 1	28	458080	687720	857820	299420	276990	127200	FALSE
3.91003	4.08E-05	TRUE	2847167	235860.3	Glyr1	Putative oxidoreductase GLYR1	13	68761	420730	218090	2177700	3713200	2650600	FALSE
-0.21781	0.084742	FALSE	1042593	1187633	Myo5a	Unconventional myosin-Va	45	1319300	1159100	1084500	1329800	775280	1022700	FALSE
0.339913	0.059292	FALSE	59167.67	31029.67	Whsc1l1	Histone-lysine N-methyltransferase;Histone-lysine N-methyltransferase NSD3	7	47799	45290	0	52292	66350	58861	FALSE
2.626973	8.06E-06	TRUE	1457967	238356.7	Ntm	Neurotrimin	5	300850	208250	205970	1693300	1335300	1345300	FALSE
2.518816	0.00032	TRUE	414426.7	70823.33	Dlgap1	Disks large-associated protein 1	4	77866	101200	33404	692200	209670	341410	FALSE
-2.57501	8.06E-06	TRUE	150380	880663.3	Ctnna2	Catenin alpha-2	22	867820	925380	848790	191040	123040	137060	FALSE
-0.08676	0.122604	FALSE	129526.7	204940	Nrxn1	Neurexin-1	12	201070	210310	203440	171460	217120	0	FALSE
-0.58117	0.090138	FALSE	7203197	4721300	Macf1	Microtubule-actin cross-linking factor 1	43	4605700	6336200	3222000	1596100	921490	19092000	FALSE
0.783739	0.018599	FALSE	211313.3	77986.67	**Col12a1**	**Collagen alpha-1(XII) chain**	13	0	124040	109920	187440	142620	303880	TRUE
-0.21172	0.117129	FALSE	30176.33	40782	Fmnl2	Formin-like protein 2	6	26746	95600	0	57194	0	33335	FALSE

5.967815	1.77E-07	TRUE	18874000	321943.3	**Hspg2**	**Basement membrane-specific heparan sulfate proteoglycan core protein;Endorepellin;LG3 peptide**	67	204560	489630	271640	17404000	20723000	18495000	TRUE
-5.02466	8.84E-07	TRUE	103206	3131067	SEPT7	Septin-7	20	3974400	2492800	2926000	63584	166780	79254	FALSE
-3.35897	1.20E-05	TRUE	64433.67	668453.3	Utrn		35	375950	855750	773660	92930	56414	43957	FALSE
-2.60261	0.000957	TRUE	64573.67	331796.7	Synj1	Synaptojanin-1	14	545790	249780	199820	69410	108110	16201	FALSE
-0.37046	0.054243	FALSE	130268.7	169746.7	Clasp2	CLIP-associating protein 2	14	164420	218970	125850	136180	155750	98876	FALSE
0.004459	0.14674	FALSE	119212	118905	Mark2	Serine/threonine-protein kinase MARK2	5	152060	76145	128510	164580	82656	110400	FALSE
-1.81486	0.000276	TRUE	55531.33	298016.7	Cald1		11	255880	378150	260020	0	81983	84611	FALSE
2.671881	1.35E-05	TRUE	3327800	521223.3	Syngap1	Ras/Rap GTPase-activating protein SynGAP	29	676390	483720	403560	4363900	3063700	2555800	FALSE
-2.12901	5.77E-05	TRUE	133621.7	570766.7	Psd3	PH and SEC7 domain-containing protein 3	12	568370	670840	473090	130650	177030	93185	FALSE
-0.62223	0.013846	FALSE	170473.3	262920	Farp1	FERM, RhoGEF and pleckstrin domain-containing protein 1	11	300010	217690	271060	196570	166830	148020	FALSE
4.94944	2.15E-07	TRUE	32066667	1061670	**Lamc1**	**Laminin subunit gamma-1**	37	740110	1152300	1292600	29542000	36508000	30150000	TRUE
2.43834	8.01E-06	TRUE	2679133	494730	Supt16	FACT complex subunit SPT16	23	463300	467430	553460	2710700	2434000	2892700	FALSE
4.510553	2.49E-06	TRUE	477033.3	20922.33	Trip12	E3 ubiquitin-protein ligase TRIP12	16	11941	28268	22558	313420	715680	402000	FALSE
2.441209	7.18E-05	TRUE	536040	66490.67	Zc3h18	Zinc finger CCCH domain-containing protein 18	5	78022	0	121450	547420	636900	423800	FALSE
-1.68698	0.000694	TRUE	146985.3	442346.7	Ppp1r9a		17	570680	377630	378730	233780	120070	87106	FALSE
3.587398	1.70E-06	TRUE	2336400	200600	**Col6a3**		36	138960	262410	200430	2524100	2229700	2255400	TRUE
0.659554	0.012811	FALSE	479690	299290	Myh14	Myosin-14	26	334740	279900	283230	520880	359220	558970	FALSE
-0.18563	0.101281	FALSE	274890	306846.7	Mta1	Metastasis-associated protein MTA1	16	319450	209750	391340	183980	396090	244600	FALSE

-3.02146	5.80E-06	TRUE	1026513	8660933	Dpysl2	Dihydropyrimidinase-related protein 2	21	12161000	6304200	7517600	1109200	980970	989370	FALSE
-0.36979	0.087497	FALSE	157970	263420	Purb	Transcriptional activator protein Pur-beta	7	256850	349830	183580	105280	0	368630	FALSE
-2.13826	3.76E-05	TRUE	125143.3	559390	Cntnap1	Contactin-associated protein 1	20	682330	585460	410380	117980	111880	145570	FALSE
-1.04055	0.003471	TRUE	142626.7	296553.3	Gnaz	Guanine nucleotide-binding protein G(z) subunit alpha	10	400540	223940	265180	168760	105220	153900	FALSE
-1.0457	0.015984	TRUE	131000	440473.3	rps14	40S ribosomal protein S14	4	204700	484320	632400	236240	0	156760	FALSE
-2.1414	0.000104	TRUE	277276.7	1169587	Bsn	Protein bassoon	59	1460800	989960	1058000	402500	257290	172040	FALSE
2.092505	4.52E-05	TRUE	31377667	7531733	Gfap	Glial fibrillary acidic protein	24	5312000	7986800	9296400	28847000	30422000	34864000	FALSE
-1.72904	0.000729	TRUE	604760	1882067	Gap43	Neuromodulin	9	2460100	1822000	1364100	982250	462980	369050	FALSE
0.219361	0.098832	FALSE	7205333	5540133	Nefm	Neurofilament medium polypeptide	38	5330400	5002100	6287900	11558000	3479400	6578600	FALSE
4.48041	1.52E-06	TRUE	9886967	482323.3	**Nid1**	**Nidogen-1**	25	248940	490510	707520	9062900	10100000	10498000	TRUE
-3.76049	1.79E-05	TRUE	81749.67	1202040	Slc3a2	4F2 cell-surface antigen heavy chain	12	561080	2053500	991540	119290	73890	52069	FALSE
-0.52553	0.029389	FALSE	1352797	1915233	Cntn1	Contactin-1	29	2167400	2072400	1505900	1779400	1294300	984690	FALSE
-2.46754	0.000159	TRUE	107400	926010	Mdh1	Malate dehydrogenase, cytoplasmic	7	548730	1000100	1229200	132630	189570	0	FALSE
-1.85035	0.000194	TRUE	2209533	7834900	Gnao1	Guanine nucleotide-binding protein G(o) subunit alpha	18	10159000	7241600	6104100	3090900	1595500	1942200	FALSE
4.565603	6.33E-07	TRUE	3253533	140075.7	**Lama1**	**Laminin subunit alpha-1**	36	91777	173940	154510	2500900	3912100	3347600	TRUE
-2.41142	0.000144	TRUE	385546.7	1913433	Map2	Microtubule-associated protein 2	36	2674600	1551800	1513900	318720	630130	207790	FALSE
0.114452	0.117901	FALSE	3674667	3523333	Lmnb2	Lamin-B2	24	2433100	2911600	5225300	3671600	4526700	2825700	FALSE
2.071302	4.54E-05	TRUE	683566.7	166993.3	Tgm2	Protein-glutamine gamma-glutamyltransferase 2	9	120460	165470	215050	719410	637300	693990	TRUE
-3.3497	0.00024	TRUE	58121.33	668200	Ppib	Peptidyl-prolyl cis-trans isomerase B;Peptidyl-prolyl cis-trans	8	851440	564570	588590	145950	28414	0	FALSE

						isomerase							
-0.44475	0.094692	FALSE	1254470	1366880	Pura	Transcriptional activator protein Pur-alpha	7	487240	1576400	2037000	266530	2602200	894680	FALSE
8.237935	1.85E-08	TRUE	99837333	336793.3	Hist1h1e	Histone H1.4	3	268030	298990	443360	87279000	98433000	1.14E+08	FALSE
-0.04669	0.134882	FALSE	4548067	4412067	Ina	Alpha-internexin	7	4206100	4727200	4302900	6731800	4098200	2814200	FALSE
-5.0081	1.05E-06	TRUE	46310.33	2207767	Atp6v1a	V-type proton ATPase catalytic subunit A	28	2539800	1811600	2271900	83917	55014	0	FALSE
-6.29946	1.15E-07	TRUE	22995.33	1820200	Pkm	Pyruvate kinase PKM	25	2477800	1585500	1397300	28829	16409	23748	FALSE
-2.74726	0.001694	TRUE	1514630	6812500	Plp1	Myelin proteolipid protein	6	8313200	7476700	4647600	222590	2778000	1543300	FALSE
-0.7504	0.034478	FALSE	50249	142294	Ppfia3	Liprin-alpha-3	14	247390	80903	98589	86424	0	64323	FALSE
-3.93754	3.32E-06	TRUE	200486.7	4641667	Stx1b	Syntaxin-1B	15	5647900	4431000	3846100	331980	269480	0	FALSE
-3.1217	8.15E-06	TRUE	540613.3	4847467	Ywhaz	14-3-3 protein zeta/delta	12	6426200	5101500	3014700	448450	486630	686760	FALSE
-1.94335	0.00081	TRUE	31693	200490	Hpca	Neuron-specific calcium-binding protein hippocalcin	4	144410	329060	128000	0	50238	44841	FALSE
5.477832	2.46E-07	TRUE	3978900	92030.33	**Lama4**	**Laminin subunit alpha-4**	20	63881	78930	133280	3024700	4894100	4017900	TRUE
1.627017	0.00046	TRUE	2488567	760630	Tubb5	Tubulin beta-5 chain	5	645500	800900	835490	1707100	3792800	1965800	FALSE
1.093914	0.003678	TRUE	6821700	3316767	Hnrnpul2	Heterogeneous nuclear ribonucleoprotein U-like protein 2	27	2001800	3551400	4397100	8099400	7015100	5350600	FALSE
-6.7259	1.15E-07	TRUE	60161.67	6331400	Ckb	Creatine kinase B-type	16	8353500	5722400	4918300	81599	45529	53357	FALSE
-4.64544	3.50E-05	TRUE	30128.67	858350	Rab2a	Ras-related protein Rab-2A	3	670810	830340	1073900	75343	15043	0	FALSE
0.833216	0.029389	FALSE	289536.7	163909.3	Bcas1	Breast carcinoma-amplified sequence 1 homolog	6	222220	197370	72138	450410	272310	145890	FALSE
1.046445	0.003678	TRUE	451903.3	221916.7	Hdgfrp3	Hepatoma-derived growth factor-related protein 3	3	145750	261610	258390	585250	393680	376780	FALSE
-0.94385	0.019434	FALSE	60469.67	165520	Serpinh1	Serpin H1	9	105850	164510	226200	129270	0	52139	TRUE

2.244724	0.000303	TRUE	452576.7	108412.3	Lsamp	Limbic system-associated membrane protein	6	121470	43917	159850	381050	473080	503600	FALSE
-2.0402	9.41E-05	TRUE	3299100	12922667	Cnp	2,3-cyclic-nucleotide 3-phosphodiesterase	23	11269000	14034000	13465000	4854100	2290300	2752900	FALSE
-0.89944	0.014187	FALSE	369910	669930	Arf3	ADP-ribosylation factor 1;ADP-ribosylation factor 3;ADP-ribosylation factor 2	4	978060	574640	457090	247070	270570	592090	FALSE
-0.3789	0.05439	FALSE	170653.3	220660	Lbr	Lamin-B receptor	10	204040	286390	171550	223990	124560	163410	FALSE
1.307113	0.001642	TRUE	458286.7	172313.3	Ccdc177	Coiled-coil domain-containing protein 177	9	185320	162040	169580	366590	713150	295120	FALSE
0.037689	0.136194	FALSE	173596.7	162880	Agap2	Arf-GAP with GTPase, ANK repeat and PH domain-containing protein 2	9	153560	153900	181180	109630	200870	210290	FALSE
-6.15336	1.77E-07	TRUE	89903.67	5905267	Atp2b2	Calcium-transporting ATPase;Plasma membrane calcium-transporting ATPase 2	3	6261200	6271300	5183300	143850	68995	56866	FALSE
-4.05114	4.91E-07	TRUE	107735.3	1795367	Gnaq	Guanine nucleotide-binding protein G(q) subunit alpha	7	2158700	1740900	1486500	118660	113470	91076	FALSE
-5.12182	1.19E-06	TRUE	1372243	42558667	Atp1a2	Sodium/potassium-transporting ATPase subunit alpha-2	25	47388000	38201000	42087000	2155800	1332800	628130	FALSE
-2.81564	6.54E-05	TRUE	60413.33	643370	Camkv	CaM kinase-like vesicle-associated protein	17	861170	427290	641650	68150	113090	0	FALSE
2.300592	6.54E-05	TRUE	1092487	215380	**Lamb1**	**Laminin subunit beta-1**	14	198470	169770	277900	688160	1516200	1073100	TRUE
1.022674	0.026929	TRUE	120960.7	70210.67	Rrp12	RRP12-like protein	8	79831	111310	19491	67662	165560	129660	FALSE
-2.44427	0.000827	TRUE	319529.3	1346700	Ina		2	1383100	1230800	1426200	466700	77958	413930	FALSE
-1.3902	0.001045	TRUE	192546.7	523016.7	Anpep	Aminopeptidase N	18	318550	574130	676370	153640	227330	196670	FALSE
-0.89647	0.007993	FALSE	306693.3	539933.3	Synpo	Synaptopodin	14	552330	476520	590950	434730	301210	184140	FALSE
-2.16615	3.28E-05	TRUE	633670	2812800	Mog	Myelin-oligodendrocyte glycoprotein	8	2773300	3257800	2407300	750390	473320	677300	FALSE

1.256958	0.00192	TRUE	487826.7	208816.7	Des	Desmin	8	171730	152170	302550	350810	513140	599530	FALSE
1.068484	0.004907	TRUE	493533.3	251366.7	Erh	Enhancer of rudimentary homolog	3	158090	203990	392020	414070	585590	480940	FALSE
-3.09489	2.51E-06	TRUE	81092.33	701193.3	Rab3c	Ras-related protein Rab-3C	6	866110	579590	657880	88894	78558	75825	FALSE
-3.03135	0.000134	TRUE	1150680	7916400	Atp1b1	Sodium/potassium-transporting ATPase subunit beta-1	13	9339300	5363600	9046300	1988100	1076500	387440	FALSE
-1.69488	7.85E-05	TRUE	194386.7	627106.7	Ctnna1	Catenin alpha-1	16	574970	633080	673270	181320	224120	177720	FALSE
0.685414	0.013065	FALSE	146070	90185.67	Clu	Clusterin;Clusterin;Clusterin beta chain;Clusterin alpha chain	8	70874	102530	97153	122730	188690	126790	FALSE
-4.57446	1.11E-06	TRUE	138529.3	3101267	Atp2a2	Sarcoplasmic/endoplasmic reticulum calcium ATPase 2	26	3048300	3611300	2644200	147260	192350	75978	FALSE
-0.17109	0.092766	FALSE	915356.7	1031493	Myh10	Myosin-10	42	894280	1194200	1006000	1045700	795560	904810	FALSE
-0.76716	0.005355	FALSE	104824	177923.3	Mprip		3	176000	192100	165670	91332	108880	114260	FALSE
7.759585	1.15E-07	TRUE	5824100	18372.67	**Lama2**	**Laminin subunit alpha-2**	43	0	21154	33964	5513600	5524300	6434400	TRUE
-1.74851	0.000829	TRUE	329623.3	996203.3	Elavl3	ELAV-like protein 3	8	872310	1022000	1094300	391190	145320	452360	FALSE
8.883952	1.80E-06	TRUE	9366833	35843.67	**Lama5**	**Laminin subunit alpha-5**	37	5145	84820	17566	8313000	10703000	9084500	TRUE
-2.99222	5.47E-06	TRUE	343123.3	2750767	Tmpo	Lamina-associated polypeptide 2, isoforms alpha/zeta	6	2223100	2457200	3572000	405180	354660	269530	FALSE
4.64375	1.77E-07	TRUE	28307000	1140940	**Lamb2**	**Laminin subunit beta-2**	39	956020	1175600	1291200	28990000	28263000	27668000	TRUE
-2.46706	1.20E-05	TRUE	3511267	19163333	Sptbn1	Spectrin beta chain, non-erythrocytic 1	112	22083000	18265000	17142000	4428500	2765900	3339400	FALSE
3.123713	5.02E-06	TRUE	798426.7	92990.67	Hist2h2ab	Histone H2A type 2-B;Histone H2AX	2	64732	114120	100120	642230	801150	951900	FALSE
-3.50252	1.73E-05	TRUE	68443.67	1115117	Vcl	Vinculin	24	868950	1065800	1410600	0	67501	137830	FALSE
-2.03535	0.000544	TRUE	787270	2887533	Sptbn2		71	3948300	2536200	2178100	1414000	499470	448340	FALSE
3.442604	5.02E-06	TRUE	2742933	251046.7	Znf512	Zinc finger protein 512	16	267810	312930	172400	1832300	3505600	2890900	FALSE

0.380617	0.049934	FALSE	173226.7	137230	Fam120a	Constitutive coactivator of PPAR-gamma-like protein 1	18	91900	155590	164200	162740	174430	182510	FALSE
1.654317	0.002091	TRUE	605403.3	175540	Opcml		2	224140	105190	197290	1061100	301230	453880	FALSE
-2.18937	3.76E-05	TRUE	460446.7	2043633	Atp6v0a1	V-type proton ATPase subunit a;V-type proton ATPase 116 kDa subunit a isoform 1	18	2319100	1955400	1856400	628890	397850	354600	FALSE
-2.35553	0.001742	TRUE	87791.33	535763.3	Crmp1	Dihydropyrimidinase-related protein 1	15	803700	396760	406830	44754	0	218620	FALSE
0.642169	0.02684	FALSE	286763.3	182066.7	Smchd1	Structural maintenance of chromosomes flexible hinge domain-containing protein 1	18	118410	230930	196860	275910	398070	186310	FALSE
2.352562	7.43E-05	TRUE	291583.3	58708.33	Ahdc1	AT-hook DNA-binding motif-containing protein 1	10	34639	62219	79267	278310	382830	213610	FALSE
-1.2679	0.01247	TRUE	380860	1018480	Tpm2		2	392210	788630	1874600	607190	210450	324940	FALSE
0.272085	0.065688	FALSE	5351900	4385700	Plec	Plectin	109	4075200	4454900	4627000	6555700	4737400	4762600	FALSE
-1.42457	0.00032	TRUE	405876.7	1095830	Palm	Paralemmin-1	3	1332200	929990	1025300	465790	357900	393940	FALSE
1.654636	0.000446	TRUE	4951400	1687467	Tubb2a	Tubulin beta-2A chain	3	2564600	1460900	1036900	4959100	4776300	5118800	FALSE
0.753363	0.010266	FALSE	651603.3	379260	Gja1	Gap junction protein;Gap junction alpha-1 protein	10	362350	448000	327430	829560	660720	464530	FALSE
0.046334	0.132275	FALSE	186740	180073.3	Myo1b	Unconventional myosin-Ib	15	156660	198490	185070	164290	222840	173090	FALSE
-0.42074	0.05439	FALSE	350750	463833.3	Map6	Microtubule-associated protein 6	11	568270	310250	512980	419110	215320	417820	FALSE
-2.84211	1.12E-05	TRUE	237116.7	1657200	Tln1	Talin-1	56	1291400	1832600	1847600	151390	287320	272640	FALSE
-1.33119	0.0009	TRUE	1219803	3039767	Nefh	Neurofilament heavy polypeptide	21	2279300	3567600	3272400	1630800	1080900	947710	FALSE
0.458163	0.0552	FALSE	52058	53824.33	Cnksr2	Connector enhancer of kinase suppressor of ras 2	9	66924	46468	48081	106030	0	50144	FALSE
-2.00229	0.003471	TRUE	164290.3	668080	Erlin2	Erlin-2	12	215520	769320	1019400	287610	65291	139970	FALSE

0.483359	0.034696	FALSE	236346.7	166726.7	Anks1b	Ankyrin repeat and sterile alpha motif domain-containing protein 1B	6	133560	202610	164010	177710	217270	314060	FALSE
-1.96168	0.000417	TRUE	128874.3	442493.3	Ncoa5	Nuclear receptor coactivator 5	3	431370	458100	438010	105820	217030	63773	FALSE
-4.25826	4.48E-06	TRUE	76387	1462643	Tnr	Tenascin-R	26	1665700	1931100	791130	52733	118140	58288	TRUE
-3.6167	2.52E-05	TRUE	37868	413563.3	Pfkp	ATP-dependent 6-phosphofructokinase;ATP-dependent 6-phosphofructokinase, platelet type	13	547300	387880	305510	50231	14235	49138	FALSE
-0.93643	0.005011	FALSE	102214.7	192996.7	Scai	Protein SCAI	9	178290	241060	159640	120370	116430	69844	FALSE
-0.18874	0.097019	FALSE	63677.33	109377.3	Zbtb20	Zinc finger and BTB domain-containing protein 20	10	110660	128920	88552	0	84222	106810	FALSE
-0.59209	0.01646	FALSE	556163.3	836290	Hspa12a	Heat shock 70 kDa protein 12A	22	978770	796310	733790	617830	601000	449660	FALSE
-3.0648	1.07E-05	TRUE	165585.7	1339733	Add1	Alpha-adducin	22	1655800	1336200	1027200	219210	99257	178290	FALSE
-1.45358	0.000359	TRUE	7107833	19560333	Matr3	Matrin-3	15	15631000	18960000	24090000	6752400	8557300	6013800	FALSE
-0.47549	0.05061	FALSE	109950	219783.3	Ablim1	Actin-binding LIM protein 1	12	174650	229170	255530	218100	0	111750	FALSE
5.267588	4.26E-07	TRUE	5678133	156117.3	**Nid2**	**Nidogen-2**	20	87482	166770	214100	4683100	6286200	6065100	TRUE
1.347523	0.000844	TRUE	7330633	2955700	Myh9	Myosin-9	56	1966500	3186300	3714300	7048800	8660800	6282300	FALSE
-4.23133	1.00E-05	TRUE	286416.7	7579333	Atp1a1	Sodium/potassium-transporting ATPase subunit alpha-1	21	7150700	10002000	5585300	605210	254040	0	FALSE
-3.42005	1.49E-06	TRUE	127383.3	1364367	Sfxn3	Sideroflexin-3	10	1400500	1572700	1119900	134330	143410	104410	FALSE
-3.29011	3.96E-06	TRUE	118898.3	1123167	Idh3b	Isocitrate dehydrogenase [NAD] subunit, mitochondrial	14	1244400	1005800	1119300	128100	151440	77155	FALSE
4.486555	4.26E-07	TRUE	63556667	2910933	LOC665622	Histone H2B;Histone H2B type 1-P;Histone H2B type 1-K;Histone H2B type 1-	3	3032000	2016400	3684400	56249000	64811000	69610000	FALSE

						C/E/G;Histone H2B type 2-B;Histone H2B type 1-H;Histone H2B type 1-B;Histone H2B type 1-M;Histone H2B type 1-F/J/L								
-0.4921	0.031384	FALSE	163990	225876.7	Tor1aip1	Torsin-1A-interacting protein 1	9	200400	264260	212970	144840	219810	127320	FALSE
0.205681	0.092087	FALSE	601860	541200	Bcam	Basal cell adhesion molecule	10	372660	489440	761500	532280	708140	565160	FALSE
3.561124	1.00E-05	TRUE	2126600	205963.3	Myl9	Myosin regulatory light polypeptide 9	3	116250	138210	363430	2048900	2113700	2217200	FALSE
-0.34196	0.084014	FALSE	112961.7	199006.7	Sun1	SUN domain-containing protein 1	15	137720	230050	229250	0	242690	96195	FALSE
-3.54958	8.28E-07	TRUE	425110	4992400	Hspa5	78 kDa glucose-regulated protein	24	4925700	4406800	5644700	431230	455840	388260	FALSE
-0.05516	0.130327	FALSE	1562933	1639667	Tubb3	Tubulin beta-3 chain	10	2013900	1454200	1450900	1420300	1691300	1577200	FALSE
-3.71331	9.57E-06	TRUE	53130.33	1034350	Sv2a	Synaptic vesicle glycoprotein 2A	9	1312800	1029600	760650	100440	0	58951	FALSE
0.712445	0.019951	FALSE	142467.7	80711.67	No66	Bifunctional lysine-specific demethylase and histidyl-hydroxylase NO66	5	94785	70548	76802	105720	93773	227910	FALSE
-0.84298	0.003674	FALSE	137213.3	245886.7	Dclk1	Serine/threonine-protein kinase DCLK1	18	256390	261370	219900	145290	120440	145910	FALSE
-2.22737	6.51E-05	TRUE	95606	476093.3	Pclo	Protein piccolo	42	711040	411130	306110	99089	97868	89861	FALSE
0.225521	0.08969	FALSE	928476.7	791200	Khdrbs3	KH domain-containing, RNA-binding, signal transduction-associated protein 3	4	595610	1050400	727590	987260	631870	1166300	FALSE
0.307006	0.061986	FALSE	114723.3	90491.67	Myo1c	Unconventional myosin-Ic	15	83129	96747	91599	94368	153520	96282	FALSE
0.090253	0.132275	FALSE	147012.7	81721	Khdrbs2	KH domain-containing, RNA-binding, signal transduction-associated protein 2	4	0	91173	153990	91287	267590	82161	FALSE
-1.69583	0.00056	TRUE	54211.33	262476.7	Ncdn	Neurochondrin	16	334110	229990	223330	0	97846	64788	FALSE
2.662454	6.06E-06	TRUE	1195400	189380	Homer1	Homer protein homolog 1	13	224140	162430	181570	1378900	1065100	1142200	FALSE

-0.93499	0.045362	FALSE	243006.7	499070	Dst	Dystonin	25	743200	436370	317640	94790	634230	0	FALSE
-3.49248	5.81E-06	TRUE	339076.7	3782200	Ank2	Ankyrin-2	3	4995100	3805800	2545700	485820	238730	292680	FALSE
-2.81199	6.51E-05	TRUE	73053.33	712173.3	Ank3		9	803120	700020	633380	152250	66910	0	FALSE
-1.88536	7.43E-05	TRUE	257390	944643.3	Gnas	Guanine nucleotide-binding protein G(s) subunit alpha isoforms short;Guanine nucleotide-binding protein G(s) subunit alpha isoforms XLas	7	1118500	924070	791360	313100	259200	199870	FALSE
-	-	-	152811.3	0	**Col15a1**	**Collagen alpha-1(XV) chain;Restin**	4	0	0	0	93704	157420	207310	TRUE
-	-	-	88053.33	0	**Lamc3**	**Laminin subunit gamma-3**	4	0	0	0	0	111900	152260	TRUE
-	-	-	5922567	0	**Col4a2**	**Collagen alpha-2(IV) chain;Canstatin**	7	0	0	0	6293800	5958400	5515500	TRUE
-	-	-	985473.3	0	**Col18a1**	**Collagen alpha-1(XVIII) chain;Endostatin**	8	0	0	0	768520	1160600	1027300	TRUE
-	-	-	56686	0	Gpc5	Glypican-5;Secreted glypican-5	4	0	0	0	41908	128150	0	TRUE
-	-	-	60717	0	Sdc4	Syndecan-4;Syndecan	3	0	0	0	78857	56195	47099	TRUE
-	-	-	384390	0	**Col4a1**	**Collagen alpha-1(IV) chain;Arresten**	2	0	0	0	0	600610	552560	TRUE
-	-	-	4430233	0	**Col1a1**	**Collagen alpha-1(I) chain**	3	0	0	0	3816800	3450900	6023000	TRUE
-	-	-	581703.3	0	**Col1a2**	**Collagen alpha-2(I) chain**	6	0	0	0	389210	647450	708450	TRUE
-	-	-	325780	0	**Col6a2**	**Collagen alpha-2(VI) chain**	4	0	0	0	368650	0	608690	TRUE
-	-	-	46927.33	0	Emilin1	EMILIN-1	5	0	0	0	46782	57261	36739	TRUE
-	-	-	755953.3	0	Itih5	Inter-alpha-trypsin inhibitor heavy chain H5	13	0	0	0	462500	1161200	644160	TRUE

Human proteins quantified with HpH fractionation

protein	log2fc	q.mod	Significant	ECM mean	cellular mean	gene	description	Unique peptides	cellular1	cellular2	cellular3	ECM1	ECM2	ECM3	ECM marker
A0A024R4E5	1.407561	0.010745966	TRUE	6170	1505.13	HDLBP	Vigilin	8	2386.9	0	2128.5	8360	4649.6	5500.4	FALSE
A0A024R571	-2.43218	0.000349195	TRUE	7655	39908	EHD1	EH domain-containing protein 1	7	48195	32926	38603	5202.9	10876	6886.1	FALSE
A0A087WTJ2	0.924247	0.035473216	FALSE	9873.233	3496.6	GIMAP1-GIMAP5	GTPase IMAP family member 5	5	4554.8	5935	0	9501.2	10363	9755.5	FALSE
A0A087WUK2	-2.07962	0.000623524	TRUE	18401.67	76694.7	HNRNPDL	Heterogeneous nuclear ribonucleoprotein D-like	10	64005	91947	74132	13889	17365	23951	FALSE
A0A087WW77	0.050143	0.298497235	FALSE	3947.133	5663.97	LLGL1	Lethal(2) giant larvae protein homolog 1	10	7247.4	5663.6	4080.9	7495.6	0	4345.8	FALSE
A0A087WXM8	1.3289	0.047314512	TRUE	41483.33	8728.67	BCAM	Basal cell adhesion molecule	11	10558	15628	0	83885	23573	16992	FALSE
A0A087WY00	0.40879	0.137071549	FALSE	34405.67	26330.7	MYO5A	Unconventional myosin-Va	36	23032	22361	33599	35536	31425	36256	FALSE
A0A087WYF1	4.863386	2.13E-05	TRUE	307660	12500.6	**LAMA2**	**Laminin subunit alpha-2**	59	11113	21694	4694.9	350890	333590	238500	TRUE
A0A087WZF1	0.977519	0.094652195	FALSE	4751	1552.13	LPP	Lipoma-preferred partner	6	3489	1167.4	0	8950.9	2521.8	2780.3	FALSE
A0A087X0S5	2.539641	0.00099924	TRUE	71606.33	13253.5	**COL6A1**	**Collagen alpha-1(VI) chain**	13	22374	10635	6751.4	87466	84754	42599	TRUE
A0A087X165	-0.12777	0.286230994	FALSE	8184.533	7751.07	SRCIN1	SRC kinase signaling inhibitor 1	15	5398.1	4772.1	13083	4207	3686.6	16660	FALSE
A0A087X2E3	-0.60592	0.132681342	FALSE	32115	50143	SYN2	Synapsin-2	17	24264	48698	77467	17227	31964	47154	FALSE
A0A0A0MQZ2	0.838976	0.150727095	FALSE	5524.133	3635.07	SYNGAP1	Ras/Rap GTPase-activating protein SynGAP	11	1847.9	0	9057.3	0	4399.4	12173	FALSE
A0A0A0MS47	0.469433	0.178134763	FALSE	6741.5	7130.47	OPALIN	Opalin	3	10683	4389.2	6319.2	0	5982.5	14242	FALSE
A0A0A0MSG2	-0.10551	0.289270937	FALSE	1779.567	1995.77	FHL2	Four and a half LIM domains protein 2	2	1873.2	4114.1	0	0	3353.1	1985.6	FALSE

A0A0A0MTC7	4.253107	5.41E-05	TRUE	49922	1707.07	LAMA4	**Laminin subunit alpha-4**	19	1899.5	3221.7	0	40590	74437	34739	TRUE
A0A0A0MTH3	1.987298	0.002599772	TRUE	12382.3	2005.97	ILK	Integrin-linked protein kinase	7	2766.7	3251.2	0	14796	7822.9	14528	FALSE
A0A0D9SFK2	-0.98034	0.026996657	FALSE	4679.367	8745.4	MYO18A	Unconventional myosin-XVIIIa	17	8659.2	8347.8	9229.2	5912.2	2716.1	5409.8	FALSE
A0A0J9YYF7	-0.42587	0.184457481	FALSE	11956	14031	DAAM2	Disheveled-associated activator of morphogenesis 2	6	10943	18084	13066	16814	14755	4299	FALSE
A0A0J9YYG4	-2.58671	0.002150284	TRUE	1827.1	16167.6	PON2	Serum paraoxonase/arylesterase 2	11	22822	17254	8426.7	0	1580.3	3901	FALSE
A0A0X1KG75	-0.11159	0.289943219	FALSE	2231.5	1524.1	COBLL1	Cordon-bleu protein-like 1	7	1298.8	0	3273.5	988.6	1800.3	3905.6	FALSE
A0A1B0GTW1	1.250326	0.020326677	TRUE	4416.7	1188.47	TJP2	Tight junction protein ZO-2	14	1971.4	1594	0	5287.7	2690.3	5272.1	FALSE
A0A1C7CYX9	-1.5938	0.001843452	TRUE	210826.7	640193	DPYSL2	Dihydropyrimidinase-related protein 2	28	582640	771840	566100	201540	193220	237720	FALSE
A0A286YF22	-0.37025	0.18054923	FALSE	13254.43	16647.7	PHGDH	D-3-phosphoglycerate dehydrogenase	11	22853	12161	14929	18752	13319	7692.3	FALSE
A0MZ66-4	-0.23782	0.220382689	FALSE	3929.867	3085.73	KIAA1598	Shootin-1	9	4695.8	4561.4	0	3859.5	3725.6	4204.5	FALSE
A6NMH6	-2.86962	0.000214547	TRUE	4599.3	49905.3	SEPT8	Septin-8	10	47102	51010	51604	7922.4	5875.5	0	FALSE
B1AKY9	-4.68698	2.43E-05	TRUE	2859.933	108599	ATP1A2	Sodium/potassium-transporting ATPase subunit alpha-2	19	75548	136510	113740	3009.3	5570.5	0	FALSE
B4DKF8	0.62446	0.092859107	FALSE	6049.867	4225.67	PSD3	PH and SEC7 domain-containing protein 3	7	3346.1	2658.2	6672.7	6306.5	5157.7	6685.4	FALSE
B4DUR8	-1.89299	0.002613834	TRUE	3560.9	19601.7	CCT3	T-complex protein 1 subunit gamma	13	23663	19037	16105	6508.3	4174.4	0	FALSE
B7Z2R7	-0.06534	0.294172073	FALSE	3272.367	2255.8	ACBD5	Acyl-CoA-binding domain-containing protein 5	4	3873.2	2894.2	0	2940.4	2613.9	4262.8	FALSE
C9IYN3	1.217468	0.015884796	TRUE	12147.67	3432	HNRNPLL	Heterogeneous nuclear ribonucleoprotein L-like	12	5496.7	0	4799.3	10864	10192	15387	FALSE

C9J712	0.103632	0.281690689	FALSE	17151.33	15631	PFN2	Profilin;Profilin-2	3	19720	10803	16370	19780	10171	21503	FALSE
C9JEJ2	1.03157	0.034113315	TRUE	7074.567	5365.5	PCYT1A	Choline-phosphate cytidylyltransferase A	8	6097.5	3507.6	6491.4	0	11363	9860.7	FALSE
C9JEU5	3.102522	0.000110325	TRUE	177570	18825.7	FGG	Fibrinogen gamma chain	15	19797	17775	18905	106930	289700	136080	TRUE
D6RGG3	2.978309	0.00109275	TRUE	27439.67	2384.47	**COL12A1**	**Collagen alpha-1(XII) chain**	20	5132.5	2020.9	0	37976	29986	14357	TRUE
D6RGI3	-2.81795	0.003255738	TRUE	2831.033	32368	SEPT11	Septin-11	9	34941	11117	51046	6063.3	0	2429.8	FALSE
E7ENL6	3.648602	0.001775853	TRUE	258586.7	19539	**COL6A3**	**Collagen alpha-3(VI) chain**	50	51454	7163.1	0	277240	357830	140690	TRUE
E7ES33	-1.58663	0.001814107	TRUE	39360	118937	SEPT7	Septin-7	20	140160	112690	103960	42959	37392	37729	FALSE
E7ESP9	-0.77267	0.085298362	FALSE	299453.3	454570	NEFM	Neurofilament medium polypeptide	40	519430	531850	312430	195280	538470	164610	FALSE
E7EWK3	0.225077	0.246805362	FALSE	1421.39	763.653	DHX36	ATP-dependent RNA helicase DHX36	4	948.16	0	1342.8	1515.7	761.87	1986.6	FALSE
E9PCN2	-0.68548	0.090629595	FALSE	814.3333	2047.8	GUCY1B3	Guanylate cyclase soluble subunit beta-1	10	2876.7	1453.1	1813.6	1221.7	0	1221.3	FALSE
E9PD68	-1.1003	0.030259132	TRUE	10877.27	25017.3	CRMP1	Dihydropyrimidinase-related protein 1	17	22421	13038	39593	7624.8	10982	14025	FALSE
E9PDG8	-3.35252	0.000110214	TRUE	1126.333	17731.3	SNAP91	Clathrin coat assembly protein AP180	6	14119	15307	23768	0	1720.5	1658.5	FALSE
E9PEZ7	-0.68028	0.189558427	FALSE	1054.417	2370.68	ABI2	Abl interactor 2	3	6282.8	829.24	0	0	2269.1	894.15	FALSE
E9PF17	-1.94365	0.009498115	TRUE	7740.9	23429	VCAN	Versican core protein	10	21786	28644	19857	7699.2	13416	2107.5	TRUE
E9PFH7	-0.98008	0.043266637	FALSE	2566.1	5776.9	MAPK8IP3	C-Jun-amino-terminal kinase-interacting protein 3	6	3681	3336.7	10313	3029.5	2290.8	2378	FALSE
E9PGC8	-1.90885	0.000616438	TRUE	14670.67	54964	MAP1A	Microtubule-associated protein 1A;MAP1A heavy chain;MAP1 light chain LC2	37	59311	53849	51732	14870	12999	16143	FALSE

E9PHM6	-1.14644	0.009498115	TRUE	5546.433	12407.6	DST	Dystonin	33	14098	9982.9	13142	5570.4	5389.6	5679.3	FALSE
E9PR44	-0.29719	0.187690496	FALSE	112380	143006	CRYAB	Alpha-crystallin B chain	11	189450	141480	98089	108070	116750	112320	FALSE
F2Z357	1.604356	0.004311612	TRUE	21554.67	7585.97	RAP1GAP	Rap1 GTPase-activating protein 1	11	11991	5175.7	5591.2	25058	18106	21500	FALSE
F5GYN4	0.243723	0.221307119	FALSE	10533.33	13522.3	OTUB1	Ubiquitin thioesterase OTUB1	8	16690	11329	12548	15303	16297	0	FALSE
F5H5G1	0.22736	0.231529867	FALSE	2661.333	3491.4	LSAMP	Limbic system-associated membrane protein	6	3771.2	4210.6	2492.4	4132.2	0	3851.8	FALSE
F8VPF3	-0.2196	0.246302174	FALSE	31460	39247.7	MYL6	Myosin light polypeptide 6	2	67301	23612	26830	22094	45309	26977	FALSE
F8VZJ2	-0.08559	0.299047766	FALSE	11646.93	5332.97	NACA	Nascent polypeptide-associated complex subunit alpha;Nascent polypeptide-associated complex subunit alpha, muscle-specific form	3	7963.4	8035.5	0	9886.8	1869	23185	FALSE
F8W1K5	-1.51567	0.011322243	TRUE	2202.3	8781.4	CNPY2	Protein canopy homolog 2	3	7658	7995.2	10691	0	2002.8	4604.1	FALSE
G3V1A4	-3.04363	0.000387587	TRUE	8985	107255	CFL1	Cofilin-1	7	94504	83880	143380	18110	0	8845	FALSE
G3V1L9	2.487229	0.000616438	TRUE	146200	28840.3	TJP1	Tight junction protein ZO-1	34	23970	47161	15390	161990	124290	152320	FALSE
G3XAG1	2.447708	0.002357315	TRUE	22483	2471.27	ZNF512	Zinc finger protein 512	12	0	3046.2	4367.6	37314	11085	19050	FALSE
G5E9D8	0.601483	0.133091354	FALSE	7002.967	3244.37	ITIH5	Inter-alpha-trypsin inhibitor heavy chain H5	10	6849.3	2883.8	0	9735.7	6162.1	5111.1	TRUE
G5EA31	0.445054	0.174442531	FALSE	6823.7	3786.17	SEC24C	Protein transport protein Sec24C	6	0	3004	8354.5	7048.6	6903.2	6519.3	FALSE
H0Y742	0.729824	0.150727095	FALSE	6349.033	3359.23	SUN1	SUN domain-containing protein 1	7	2663.2	1563.5	5851	1524.9	10731	6791.2	FALSE
H3BPE1	-0.34782	0.159773393	FALSE	42677	53513	MACF1	Microtubule-actin cross-linking factor 1, isoforms 1/2/3/5	29	53354	49522	57663	52654	33616	41761	FALSE
H7BXI1	-1.19287	0.015798735	TRUE	5032.6	11821.1	ESYT2	Extended synaptotagmin-2	17	8255.5	17482	9725.9	5977.7	3492.6	5627.5	FALSE

H7C3P7	-0.6888	0.114606423	FALSE	10001.7	23634.3	RALA	Ras-related protein Ral-A	3	26631	30403	13869	0	9338.1	20667	FALSE
I3L0N3	0.170618	0.254505786	FALSE	203170	171330	NSF	Vesicle-fusing ATPase	30	140970	153920	219100	121410	305100	183000	FALSE
J3KQA0	-2.50086	0.000196483	TRUE	9887.167	55936	SYT1		2	57677	67079	43052	12541	8367.1	8753.4	FALSE
J3QRD1	-2.22068	0.001209833	TRUE	1520.933	10887.9	ALDH3A2	Fatty aldehyde dehydrogenase	5	9998.1	8243.7	14422	2481.2	0	2081.6	FALSE
J3QRS3	1.261777	0.010126029	TRUE	251763.3	109568	MYL12A	Myosin regulatory light chain 12A;Myosin regulatory light chain 12B	4	72173	101510	155020	288390	247600	219300	FALSE
J3QRU8	-0.47808	0.153822046	FALSE	4113.733	5576.43	GIT1	ARF GTPase-activating protein GIT1	8	3807.4	4704.2	8217.7	2169.3	4214.3	5957.6	FALSE
J3QSS3	0.941356	0.032021429	FALSE	5227.467	2674.6	ABCA2	ATP-binding cassette sub-family A member 2	10	3168.6	2955.5	1899.7	7190.5	3533.7	4958.2	FALSE
M0R116	-5.54541	0.001209833	TRUE	13076.5	334760	ATP1A3	Sodium/potassium-transporting ATPase subunit alpha-3	20	253810	394550	355920	1309.5	37920	0	FALSE
O00264	-3.56736	0.000326826	TRUE	1908.633	38227.7	PGRMC1	Membrane-associated progesterone receptor component 1	7	64124	31717	18842	2534.2	0	3191.7	FALSE
O00468-6	1.963572	0.000996826	TRUE	285213.3	74432.3	AGRN	Agrin;Agrin N-terminal 110 kDa subunit;Agrin C-terminal 110 kDa subunit;Agrin C-terminal 90 kDa fragment;Agrin C-terminal 22 kDa fragment	45	86488	86375	50434	339850	285000	230790	TRUE
O00560	0.489185	0.132731208	FALSE	3733.967	4047	SDCBP	Syntenin-1	5	3554.9	5040.2	3545.9	5617.6	5584.3	0	FALSE
O00764	-1.11619	0.083309932	FALSE	5916.9	11532.4	PDXK	Pyridoxal kinase	9	10839	4059.2	19699	2135.9	3211.8	12403	FALSE
O14531	-1.5948	0.004979998	TRUE	6042.1	18964.7	DPYSL4	Dihydropyrimidinase-related protein 4	11	27967	11568	17359	4780.2	8050.5	5295.6	FALSE
O14639-2	0.569722	0.146980931	FALSE	2976.567	2048.2	ABLIM1	Actin-binding LIM protein 1	9	3972.8	2171.8	0	0	3501.8	5427.9	FALSE
O14773-2	0.090418	0.289278808	FALSE	15063.33	21288	TPP1	Tripeptidyl-peptidase 1	6	20228	13705	29931	15817	29373	0	FALSE
O14880	-2.2191	0.001843452	TRUE	6620.333	43558	MGST3	Microsomal glutathione S-transferase 3	8	43759	51458	35457	6313	0	13548	FALSE

	1														
O14974-4	0.898623	0.063771181	FALSE	2603.1	1388.17	PPP1R12A	Protein phosphatase 1 regulatory subunit 12A	6	2381.2	1783.3	0	4603	0	3206.3	FALSE
O15020-2	-2.27634	0.003639492	TRUE	5686.8	22403.7	SPTBN2	Spectrin beta chain, non-erythrocytic 2	27	16127	24970	26114	1744.1	5288.3	10028	FALSE
O15061	2.236138	0.002567715	TRUE	32605.67	7923.03	SYNM	Synemin	19	14455	3511.5	5802.6	40463	36497	20857	FALSE
O15075-2	0.359789	0.174442531	FALSE	16883	12765.3	DCLK1	Serine/threonine-protein kinase DCLK1	12	11388	16255	10653	10429	17908	22312	FALSE
O15230	6.769513	2.25E-06	TRUE	361633.3	2177.9	**LAMA5**	**Laminin subunit alpha-5**	51	3629.6	2904.1	0	299070	468970	316860	TRUE
O43236-2	-1.29658	0.010745966	TRUE	5455.667	20080.3	SEPT4	Septin-4	8	20542	21405	18294	7521.6	0	8845.4	FALSE
O43237	-0.51493	0.116315859	FALSE	12263.43	17515	DYNC1LI2	Cytoplasmic dynein 1 light intermediate chain 2	14	23931	13530	15084	10838	9244.3	16708	FALSE
O43294-2	-0.30026	0.200045093	FALSE	2988.967	5492.93	TGFB1I1	Transforming growth factor beta-1-induced transcript 1 protein	6	4255.4	6166.2	6057.2	0	5349.4	3617.5	FALSE
O43491-4	0.496038	0.205650545	FALSE	24106	22992.5	EPB41L2	Band 4.1-like protein 2	18	38739	26311	3927.5	26882	32636	12800	FALSE
O43581	-0.7963	0.05135043	FALSE	2224	2564.27	SYT7	Synaptotagmin-7	6	3861.3	3831.5	0	2035.9	2126.5	2509.6	FALSE
O60268-3	0.444366	0.143574906	FALSE	5955.2	6607.73	KIAA0513	Uncharacterized protein KIAA0513	3	5674.6	6634.9	7513.7	8999.8	8865.8	0	FALSE
O60282	-1.84248	0.002599772	TRUE	1215.733	6655.57	KIF5C	Kinesin heavy chain isoform 5C	9	7610.5	4988.2	7368	1817.6	0	1829.6	FALSE
O60331-4	0.084754	0.291266836	FALSE	3477	2406.83	PIP5K1C	Phosphatidylinositol 4-phosphate 5-kinase type-1 gamma	6	1993.1	5227.4	0	2748.6	3439.2	4243.2	FALSE
O60493-4	1.393514	0.019378988	TRUE	13596.67	5304.5	SNX3	Sorting nexin-3	3	9895.1	6018.4	0	0	18177	22613	FALSE
O60504	0.708013	0.097267048	FALSE	7431.733	4591.67	SORBS3	Vinexin	9	5704.4	8070.6	0	9957.2	0	12338	FALSE
O60506-2	-2.70157	0.000110214	TRUE	8276.2	53349.3	SYNCRIP	Heterogeneous nuclear ribonucleoprotein Q	15	42746	57752	59550	8021.4	6393.2	10414	FALSE
O60716	1.1185	0.051581	FALSE	10780.	6066.0	CTNND1	Catenin delta-1	15	3026.4	2835.8	12336	13252	6491.4	12597	FALS

	05	036		13	7										E
O60825-2	0.884564	0.079162516	FALSE	2582.6	1360.07	PFKFB2	6-phosphofructo-2-kinase/fructose-2,6-bisphosphatase 2;6-phosphofructo-2-kinase;Fructose-2,6-bisphosphatase	4	2445.9	1634.3	0	0	5049.6	2698.2	FALSE
O60884	1.162412	0.01017078	TRUE	9488.933	4212.53	DNAJA2	DnaJ homolog subfamily A member 2	9	4161.2	3547.4	4929	7259.8	10392	10815	FALSE
O75145	1.257735	0.016727974	TRUE	10421.67	4340.63	PPFIA3	Liprin-alpha-3	14	2903.7	3725.8	6392.4	6325	10019	14921	FALSE
O75369-2	1.081626	0.0922531	FALSE	10223.93	8347.17	FLNB	Filamin-B	30	15461	2608.9	6971.6	8783.8	0	21888	FALSE
O75534	0.903211	0.044234496	FALSE	8014.967	2951.87	CSDE1	Cold shock domain-containing protein E1	12	3276.2	5579.4	0	8517.5	8267.4	7260	FALSE
O75582	0.869294	0.039564936	FALSE	38613	21706.7	RPS6KA5	Ribosomal protein S6 kinase alpha-5	17	20334	14475	30311	32611	49613	33615	FALSE
O75781-2	-0.01417	0.312391808	FALSE	5698.467	8486.6	PALM	Paralemmin-1	5	7179.8	8295.3	9984.7	6618.4	0	10477	FALSE
O75955	-2.34827	0.00109275	TRUE	2910.6	22754	FLOT1	Flotillin-1	12	20908	16368	30986	0	5034.2	3697.6	FALSE
O94819	-0.45707	0.130285131	FALSE	7177.833	9895.53	KBTBD11	Kelch repeat and BTB domain-containing protein 11	10	12366	7253.6	10067	8671.8	5380.6	7481.1	FALSE
O94826	-3.00951	4.89E-05	TRUE	5054.767	40443.7	TOMM70A	Mitochondrial import receptor subunit TOM70	15	47802	40253	33276	3923.1	6202.2	5039	FALSE
O94832	-0.08533	0.286667192	FALSE	10984.23	10751.3	MYO1D	Unconventional myosin-Id	21	9958.8	10056	12239	16935	9888.6	6129.1	FALSE
O94905	-1.81409	0.008469619	TRUE	15300.5	48515.3	ERLIN2	Erlin-2	12	27383	62373	55790	12375	26957	6569.5	FALSE
O94919	-2.16149	0.00215569	TRUE	5642.467	28181	ENDOD1	Endonuclease domain-containing 1 protein	8	20973	49544	14026	7666.7	4172.2	5088.5	FALSE
O95622	0.304097	0.181891656	FALSE	5750.733	4723.27	ADCY5	Adenylate cyclase type 5	7	6084.1	3997.5	4088.2	5046.5	5698.4	6507.3	FALSE
O95741	-	0.114606	FALSE	4993.7	4988.1	CPNE6	Copine-6	12	9239.7	5724.8	0	9183.8	4231.4	1566	FALS

	0.88677	423		33	7									E
O95757	-0.74413	0.091139451	FALSE	4663.9	10826.2	HSPA4L	Heat shock 70 kDa protein 4L	13	12436	8533.6	11509	9861.9	0	4129.8	FALSE
O95782-2	-5.18996	1.88E-05	TRUE	2114.127	70201.3	AP2A1	AP-2 complex subunit alpha-1	23	72354	96006	42244	795.78	3105	2441.6	FALSE
O95810	1.10169	0.015798735	TRUE	86646.33	39326	SDPR	Serum deprivation-response protein	7	36134	47064	34780	108920	57222	93797	FALSE
P00750-3	0.456853	0.144414298	FALSE	3448.667	1691.73	PLAT	Tissue-type plasminogen activator;Tissue-type plasminogen activator chain A;Tissue-type plasminogen activator chain B	4	2994.3	2080.9	0	3821.6	3606.6	2917.8	TRUE
P01834	-0.13264	0.286230994	FALSE	8035.333	10342.6	IGKC	Ig kappa chain C region	3	7348.7	23679	0	12754	0	11352	FALSE
P02671	1.85675	0.019378988	TRUE	59092	13073.3	FGA	Fibrinogen alpha chain;Fibrinopeptide A;Fibrinogen alpha chain	11	5815	20633	12772	17490	127010	32776	TRUE
P02751	4.613474	3.71E-05	TRUE	236920	6047.13	**FN1**	**Fibronectin;Anastellin;Ugl-Y1;Ugl-Y2;Ugl-Y3**	37	8013.4	10128	0	125090	288960	296710	TRUE
P02792	-3.81266	0.000387587	TRUE	3835.333	85850	FTL	Ferritin light chain	6	96271	35589	125690	7806.1	0	3699.9	FALSE
P02794	-0.2673	0.209230308	FALSE	83409.67	102669	FTH1	Ferritin heavy chain;Ferritin heavy chain, N-terminally processed;Ferritin	10	131610	62986	113410	105090	84189	60950	FALSE
P04075	-4.95626	1.04E-05	TRUE	8854.133	250517	ALDOA	Fructose-bisphosphate aldolase A;Fructose-bisphosphate aldolase	20	249810	314660	187080	4733.9	14825	7003.5	FALSE
P04083	0.726291	0.100169192	FALSE	20518.7	10800.9	ANXA1	Annexin A1;Annexin	8	11815	12262	8325.7	29889	7589.1	24078	TRUE
P04275	-1.61782	0.004157103	TRUE	14561	45878	VWF	von Willebrand factor;von Willebrand antigen 2	46	54665	55746	27223	14621	10673	18389	TRUE
P04406	-5.16569	4.53E-06	TRUE	22489.67	739723	GAPDH	Glyceraldehyde-3-phosphate dehydrogenase	15	672110	810010	737050	35894	18530	13045	FALSE
P04899	-1.8184	0.002030983	TRUE	13828.5	48365	GNAI2	Guanine nucleotide-binding protein G(i) subunit alpha-2	9	44940	64382	35773	16183	16431	8871.5	FALSE

	6														
P05023-4	-5.02539	4.46E-05	TRUE	40887	975703	ATP1A1	Sodium/potassium-transporting ATPase subunit alpha-1	24	1049400	1089900	787810	24589	85693	12379	FALSE
P05026	-4.94749	1.92E-05	TRUE	7141.233	303083	ATP1B1	Sodium/potassium-transporting ATPase subunit beta-1	10	327560	322030	259660	6317.7	15106	0	FALSE
P06733	-6.11817	1.82E-06	TRUE	3480.133	238100	ENO1	Alpha-enolase	15	211800	244530	257970	3616.2	4198.9	2625.3	FALSE
P07195	-4.05254	0.000316238	TRUE	2601.167	76499.3	LDHB	L-lactate dehydrogenase B chain;L-lactate dehydrogenase	12	119750	82902	26846	3474.4	4329.1	0	FALSE
P07196	-0.07408	0.298497235	FALSE	301906.7	289395	NEFL	Neurofilament light polypeptide	36	326260	449390	92535	149450	633410	122860	FALSE
P08133	-6.96299	2.24E-06	TRUE	564.61	105921	ANXA6	Annexin A6;Annexin	9	113970	117090	86702	942.68	751.15	0	TRUE
P08237-3	-0.55596	0.087600571	FALSE	12020.33	17961	PFKM	ATP-dependent 6-phosphofructokinase, muscle type	15	19304	13557	21022	12791	11526	11744	FALSE
P09417	-5.68611	4.53E-06	TRUE	1145.833	87593.7	QDPR	Dihydropteridine reductase	9	92764	75405	94612	2017.5	1420	0	FALSE
P09471-2	-2.2174	0.000316238	TRUE	76966	355067	GNAO1	Guanine nucleotide-binding protein G(o) subunit alpha	2	400220	328420	336560	81430	88295	61173	FALSE
P09543-2	-0.77829	0.03952053	FALSE	718040	1224233	CNP	2,3-cyclic-nucleotide 3-phosphodiesterase	29	1246300	1392400	1034000	647980	888110	618030	FALSE
P10114	-0.12823	0.283194304	FALSE	4730.967	4780.83	RAP2A	Ras-related protein Rap-2a;Ras-related protein Rap-2c	4	7724.9	6617.6	0	0	9846.9	4346	FALSE
P10301	-1.91526	0.003491025	TRUE	2149.167	9043.7	RRAS	Ras-related protein R-Ras	2	3902.8	9572.3	13656	2048.5	2642.9	1756.1	FALSE
P11021	-1.4303	0.009988166	TRUE	48880.33	144352	HSPA5	78 kDa glucose-regulated protein	24	183650	181270	68135	44255	52710	49676	FALSE
P11047	4.11202	1.03E-05	TRUE	836290	47238	LAMC1	**Laminin subunit gamma-1**	38	48400	54096	39218	889020	1052400	567450	TRUE
P11166	-	0.000616	TRUE	12033.	50294.	SLC2A1	Solute carrier family 2, facilitated	4	65135	46504	39244	10637	11771	13692	FALS

	2.03853	438		33	3		glucose transporter member 1							E
P11216	-0.97289	0.028786118	FALSE	27232.67	57063.7	PYGB	Glycogen phosphorylase, brain form	25	87614	45286	38291	25399	29306	26993	FALSE
P11217	-0.69783	0.140859617	FALSE	10362.13	22051.7	PYGM	Glycogen phosphorylase, muscle form	17	36832	5145.1	24178	12291	10382	8413.4	FALSE
P11532	0.539395	0.120518733	FALSE	5103.7	2308.33	DMD	Dystrophin	11	3822.3	3102.7	0	6347.3	5060.3	3903.5	FALSE
P11586	0.117011	0.276742917	FALSE	18486.67	16886.3	MTHFD1	C-1-tetrahydrofolate synthase, cytoplasmic;Methylenetetrahydrofolate dehydrogenase;Methenyltetrahydrofolate cyclohydrolase;Formyltetrahydrofolate synthetase;C-1-tetrahydrofolate synthase, cytoplasmic, N-terminally processed	14	12845	13906	23908	24994	11476	18990	FALSE
P12036-2	-0.28543	0.254505786	FALSE	61732.67	67097.7	NEFH	Neurofilament heavy polypeptide	15	98947	78049	24297	56744	112170	16284	FALSE
P12277	-0.39309	0.174108192	FALSE	547443.3	683493	CKB	Creatine kinase B-type	20	769880	482130	798470	794990	301710	545630	FALSE
P12814-3	-1.77801	0.003255738	TRUE	32756.67	102454	ACTN1	Alpha-actinin-1	25	94633	107520	105210	47832	16210	34228	FALSE
P13489	-1.90417	0.00075208	TRUE	7265.267	27468	RNH1	Ribonuclease inhibitor	14	31514	21516	29374	6936.9	6764.6	8094.3	FALSE
P14136-2	-1.37713	0.005022105	TRUE	58337	154660	GFAP	Glial fibrillary acidic protein	18	192370	111200	160410	58708	64973	51330	FALSE
P14543-2	5.758037	2.95E-06	TRUE	374690	7276.23	**NID1**	**Nidogen-1**	23	9493.3	8549.1	3786.3	372460	478090	273520	TRUE
P16152	-1.07887	0.039564936	TRUE	38364.67	67035.7	CBR1	Carbonyl reductase [NADPH] 1	11	66172	63386	71549	73372	21978	19744	FALSE
P16615-5	-5.3449	1.92E-05	TRUE	3086.367	166153	ATP2A2	Sarcoplasmic/endoplasmic reticulum calcium ATPase 2	26	153010	200110	145340	2381.4	6877.7	0	FALSE

	4													
P17600	-0.1216	0.2736101	FALSE	66306.33	75658.3	SYN1	Synapsin-1	17	62028	51167	113780	53183	66806	78930	FALSE
P17677	-2.77955	0.000132836	TRUE	15838	108821	GAP43	Neuromodulin	13	101640	80893	143930	12437	13840	21237	FALSE
P17858	0.912545	0.035551904	FALSE	32616.33	17363	PFKL	ATP-dependent 6-phosphofructokinase, liver type	16	15117	12962	24010	42924	22651	32274	FALSE
P17987	-1.07604	0.019291879	TRUE	6026.967	13039.7	TCP1	T-complex protein 1 subunit alpha	15	17390	8454	13275	7625.5	5353.1	5102.3	FALSE
P18206-2	-1.06372	0.088770912	FALSE	37870.6	60379.3	VCL	Vinculin	34	75659	70176	35303	40454	7756.8	65401	FALSE
P18669	-4.72285	1.78E-05	TRUE	2197.133	83272	PGAM1	Phosphoglycerate mutase 1	14	85788	74579	89449	4284	0	2307.4	FALSE
P19086	-0.3751	0.188771287	FALSE	6791.167	10020.1	GNAZ	Guanine nucleotide-binding protein G(z) subunit alpha	9	4449.7	8759.6	16851	5305.9	7422	7645.6	FALSE
P20020-5	-3.47803	1.78E-05	TRUE	15759.33	176373	ATP2B1	Plasma membrane calcium-transporting ATPase 1;Calcium-transporting ATPase	14	145040	173040	211040	15315	18311	13652	FALSE
P20336	-2.23945	0.001843452	TRUE	16747.67	69104	RAB3A	Ras-related protein Rab-3A	4	69389	78546	59377	6511	24337	19395	FALSE
P20916-3	-2.11328	0.005375085	TRUE	6972.267	33784.7	MAG	Myelin-associated glycoprotein	10	55491	34157	11706	10128	6827.4	3961.4	FALSE
P21281	-3.17035	0.001198	TRUE	3919.533	48412	ATP6V1B2	V-type proton ATPase subunit B, brain isoform	20	24418	60109	60709	0	9033.9	2724.7	FALSE
P21291	-2.82697	0.002090589	TRUE	3762.767	29708.7	CSRP1	Cysteine and glycine-rich protein 1	9	44721	35812	8593.2	5397.2	1704.9	4186.2	FALSE
P24844	3.104931	0.001918691	TRUE	76456.67	5789.17	MYL9	Myosin regulatory light polypeptide 9	3	12945	0	4422.5	128740	70034	30596	FALSE
P26232-2	-0.37332	0.174938079	FALSE	4942.5	6308.13	CTNNA2	Catenin alpha-2	16	5624.6	4599.6	8700.2	3126.3	4800.2	6901	FALSE
P27824	-3.2268	9.60E-05	TRUE	12184.8	115098	CANX	Calnexin	23	171960	87721	85614	9312.6	9552.8	17689	FALSE

	9													
P29992	0.336135	0.188771287	FALSE	5787.067	6764.43	GNA11	Guanine nucleotide-binding protein subunit alpha-11	5	8292.3	6119.9	5881.1	10726	0	6635.2	FALSE
P30086	-5.23063	8.72E-06	TRUE	2708.2	154910	PEBP1	Phosphatidylethanolamine-binding protein 1;Hippocampal cholinergic neurostimulating peptide	10	187050	160290	117390	4361.9	3762.7	0	FALSE
P31150	-6.89131	2.25E-06	TRUE	1085.2	190637	GDI1	Rab GDP dissociation inhibitor alpha	19	230990	166970	173950	1273.5	1982.1	0	FALSE
P31689	1.121471	0.019230599	TRUE	17720.33	8125.1	DNAJA1	DnaJ homolog subfamily A member 1	10	11152	5837.2	7386.1	11421	22280	19460	FALSE
P35221	1.585618	0.004307098	TRUE	55414	19174.3	CTNNA1	Catenin alpha-1	16	21853	24447	11223	48476	47786	69980	FALSE
P35579	1.075972	0.011761288	TRUE	712923.3	334873	MYH9	Myosin-9	73	339500	358540	306580	754850	816930	566990	FALSE
P35609	-1.41545	0.011322243	TRUE	21019	59911	ACTN2	Alpha-actinin-2	2	29676	64565	85492	15754	20253	27050	FALSE
P35612	-1.44828	0.015089854	TRUE	2279.733	9986.57	ADD2	Beta-adducin	18	11995	5331.7	12633	0	3208.7	3630.5	FALSE
P38405	0.057308	0.294172073	FALSE	9581.167	9160.07	GNAL	Guanine nucleotide-binding protein G(olf) subunit alpha	9	6055.1	9110.1	12315	6003.5	10028	12712	FALSE
P38606	-3.61168	8.70E-05	TRUE	8419.867	100925	ATP6V1A	V-type proton ATPase catalytic subunit A	26	134920	110130	57725	4601.1	8174.5	12484	FALSE
P40123	-0.00811	0.314149039	FALSE	9154.267	10118.3	CAP2	Adenylyl cyclase-associated protein 2;Adenylyl cyclase-associated protein	9	14740	10819	4795.9	10665	8565.9	8231.9	FALSE
P41222	2.084753	0.001792239	TRUE	41936.67	6838.87	PTGDS	Prostaglandin-H2 D-isomerase	3	7376.6	13140	0	42725	46105	36980	FALSE
P42262-2	0.772727	0.066350097	FALSE	12145.83	6931.83	GRIA2	Glutamate receptor 2	8	4624.4	9091	7080.1	7487.5	11117	17833	FALSE
P45974-2	1.131245	0.009628529	TRUE	72062.33	32718.7	USP5	Ubiquitin carboxyl-terminal hydrolase 5	18	35656	31213	31287	63341	68507	84339	FALSE
P46821	-0.81761	0.043534799	FALSE	145710	268950	MAP1B	Microtubule-associated protein 1B;MAP1B heavy chain;MAP1 light chain LC1	64	336760	306580	163510	154690	138040	144400	FALSE
P46939	2.311454	0.00075208	TRUE	72074	15938.4	UTRN	Utrophin	48	14411	24878	8526.1	69115	74471	72636	FALSE

P47755	-1.05015	0.044460765	TRUE	3878.333	12921.1	CAPZA2	F-actin-capping protein subunit alpha-2	8	18098	13830	6835.4	6512.7	0	5122.3	FALSE
P48681	0.223016	0.242632086	FALSE	23867	18694.3	NES	Nestin	29	16585	23748	15750	26100	10932	34569	FALSE
P50148	0.278188	0.246805362	FALSE	17537	18596.5	GNAQ	Guanine nucleotide-binding protein G(q) subunit alpha	7	30960	20295	4534.6	13225	21593	17793	FALSE
P50454	0.233105	0.230727988	FALSE	3244.867	1812.33	SERPINH1	Serpin H1	7	3091.8	2345.2	0	2594	4295.1	2845.5	TRUE
P50991	-1.4882	0.004397901	TRUE	5141.667	13989.7	CCT4	T-complex protein 1 subunit delta	14	11366	16209	14394	4390	7273.5	3761.5	FALSE
P51570	1.476105	0.006537294	TRUE	10674.6	2545.53	GALK1	Galactokinase	5	4021	3615.6	0	12009	10881	9133.8	FALSE
P52294	0.803665	0.066529625	FALSE	10236.43	5519.73	KPNA1	Importin subunit alpha-5;Importin subunit alpha-5, N-terminally processed	4	7155.3	5392.2	4011.7	14730	10809	5170.3	FALSE
P52907	-0.32432	0.188771287	FALSE	6817	8887.7	CAPZA1	F-actin-capping protein subunit alpha-1	6	7716.4	13199	5747.7	8269.6	5074.4	7107	FALSE
P53621	0.690255	0.10519295	FALSE	14542.33	10638.2	COPA	Coatomer subunit alpha;Xenin;Proxenin	18	20127	6013.9	5773.6	16895	11158	15574	FALSE
P54289-3	-0.72606	0.082983613	FALSE	2983.867	7617.07	CACNA2D1	Voltage-dependent calcium channel subunit alpha-2/delta-1;Voltage-dependent calcium channel subunit alpha-2-1;Voltage-dependent calcium channel subunit delta-1	19	5573.6	10578	6699.6	0	5073.4	3878.2	FALSE
P54829-2	0.371187	0.192319785	FALSE	4326.3	3385.9	PTPN5	Tyrosine-protein phosphatase non-receptor type 5;Protein-tyrosine-phosphatase	8	6208.2	0	3949.5	5443.5	0	7535.4	FALSE
P55011	-0.8229	0.092959457	FALSE	6729.1	12015.9	SLC12A2	Solute carrier family 12 member 2	15	15136	15753	5158.6	8277.2	3019.5	8890.6	FALSE
P55060-3	-0.04668	0.300481896	FALSE	2900.633	4650.7	CSE1L	Exportin-2	13	6800.1	4548.2	2603.8	5555.5	3146.4	0	FALSE
P55209	-0.7534	0.066350097	FALSE	8589.933	13751.7	NAP1L1	Nucleosome assembly protein 1-like 1	5	15202	15499	10554	10358	4661.8	10750	FALSE
P55268	4.931183	2.25E-06	TRUE	1125647	37141.3	**LAMB2**	**Laminin subunit beta-2**	44	41904	41016	28504	1130700	1299000	947240	TRUE
P60201-2	-2.64329	0.000345685	TRUE	184813.3	1127713	PLP1	Myelin proteolipid protein	5	1305000	1325500	752640	193730	250850	109860	FALSE

P60880	-3.33812	1.92E-05	TRUE	13317.67	135410	SNAP25	Synaptosomal-associated protein 25	4	163300	123860	119070	11635	14741	13577	FALSE
P61158	-2.17296	0.002839319	TRUE	2571.933	18715.1	ACTR3	Actin-related protein 3	9	20821	9652.4	25672	0	3410.1	4305.7	FALSE
P61266-2	-3.85286	9.21E-06	TRUE	12631.67	181450	STX1B	Syntaxin-1B	17	187740	160890	195720	15117	11148	11630	FALSE
P62330	1.242552	0.011761288	TRUE	10830.93	4741.8	ARF6	ADP-ribosylation factor 6	4	4193.4	3153.9	6878.1	8643.8	13591	10258	FALSE
P62873	-5.24528	1.78E-05	TRUE	6526.233	209623	GNB1	Guanine nucleotide-binding protein G(I)/G(S)/G(T) subunit beta-1	7	207590	146680	274600	12537	3102.8	3938.9	FALSE
P62937	-2.42246	0.013385473	TRUE	24976.73	90872.3	PPIA	Peptidyl-prolyl cis-trans isomerase A;Peptidyl-prolyl cis-trans isomerase A, N-terminally processed;Peptidyl-prolyl cis-trans isomerase	9	89977	136140	46500	49575	3396.2	21959	FALSE
P63010	-2.76141	0.000115979	TRUE	8894.633	59600.3	AP2B1	AP-2 complex subunit beta	18	52028	50927	75846	7340.1	7279.8	12064	FALSE
P63092-3	-0.65666	0.061307992	FALSE	14388.33	22565.3	GNAS	Guanine nucleotide-binding protein G(s) subunit alpha isoforms short;Guanine nucleotide-binding protein G(s) subunit alpha isoforms XLas	8	26108	19331	22257	11951	17519	13695	FALSE
P63261	-0.75971	0.039732888	FALSE	1006170	1698500	ACTG1	Actin, cytoplasmic 2;Actin, cytoplasmic 2, N-terminally processed;Actin, cytoplasmic 1;Actin, cytoplasmic 1, N-terminally processed	4	1964800	1558400	1572300	865190	963520	1189800	FALSE
P78347-2	5.280194	2.83E-05	TRUE	71317.33	2266.41	GTF2I	General transcription factor II-I	19	2883.6	3303.4	612.23	90860	56910	66182	FALSE
P78352-3	0.089287	0.281137702	FALSE	9408.633	8789.6	DLG4	Disks large homolog 4	13	7420.2	9406	9542.6	8357.2	8279.7	11589	FALSE
P78356	0.300239	0.188771287	FALSE	12428	10553.4	PIP4K2B	Phosphatidylinositol 5-phosphate 4-kinase type-2 beta	7	12942	12253	6465.1	11892	13178	12214	FALSE
P78357	-1.45287	0.011491177	TRUE	18716.67	52744.3	CNTNAP1	Contactin-associated protein 1	31	75676	55062	27495	26271	11682	18197	FALSE
P84074	-	0.010454	TRUE	6791.4	43127	HPCA	Neuron-specific calcium-binding	7	66805	43863	18713	15886	0	4488.4	FALS

	2.16965	987		67			protein hippocalcin							E
P84077	-0.84717	0.052883666	FALSE	19976.33	36761.3	ARF1	ADP-ribosylation factor 1;ADP-ribosylation factor 3	4	37974	51046	21264	17188	28063	14678	FALSE
P84095	-0.05579	0.294172073	FALSE	12066	13471	RHOG	Rho-related GTP-binding protein RhoG	5	21153	9105.1	10155	11311	13374	11513	FALSE
P98160	7.260183	2.24E-06	TRUE	600620	2558.17	**HSPG2**	**Basement membrane-specific heparan sulfate proteoglycan core protein;Endorepellin;LG3 peptide**	72	3891.7	3782.8	0	628870	729350	443640	TRUE
Q00013-2	-0.08873	0.286667192	FALSE	2276.467	1561.57	MPP1	55 kDa erythrocyte membrane protein	6	2188.7	0	2496	1869.1	1794.1	3166.2	FALSE
Q01064	-0.86577	0.052883666	FALSE	12272.33	24108.3	PDE1B	Calcium/calmodulin-dependent 3,5-cyclic nucleotide phosphodiesterase 1B	16	28853	11684	31788	15006	11865	9946	FALSE
Q01484	-2.03587	0.000596564	TRUE	24537.33	99154.3	ANK2	Ankyrin-2	62	111260	88803	97400	30665	19022	23925	FALSE
Q01518-2	-1.33646	0.005441376	TRUE	9491.867	23466.7	CAP1	Adenylyl cyclase-associated protein 1	12	24726	24273	21401	11519	6852.6	10104	FALSE
Q01813	0.180798	0.246805362	FALSE	94870	86715.3	PFKP	ATP-dependent 6-phosphofructokinase, platelet type	20	59984	74442	125720	88097	78923	117590	FALSE
Q02952-3	-1.55033	0.011591337	TRUE	6629	16916.3	AKAP12	A-kinase anchor protein 12	23	20698	16130	13921	2547.3	7074.7	10265	FALSE
Q03252	-2.32104	0.000588161	TRUE	33200.67	154377	LMNB2	Lamin-B2	25	164600	159690	138840	52196	22496	24910	FALSE
Q04917	-1.21496	0.011322243	TRUE	14775.67	33953.7	YWHAH	14-3-3 protein eta	9	25628	33660	42573	15487	10135	18705	FALSE
Q05469-2	0.553593	0.116315859	FALSE	14307.33	6356.47	LIPE	Hormone-sensitive lipase	8	9203.8	0	9865.6	16425	16242	10255	FALSE
Q05639	-0.03285	0.304363501	FALSE	5517.967	5858.07	EEF1A2	Elongation factor 1-alpha 2	5	5918.5	3043.3	8612.4	4506.1	8057.4	3990.4	FALSE
Q05682	-	0.188771	FALSE	14422.	16397.	CALD1	Caldesmon	10	21025	12674	15494	25042	5907.1	12319	FALS

	0.39331	287		7	7										E
Q05707-2	0.40568	0.18681444	FALSE	6031.3	4362.2	**COL14A1**	**Collagen alpha-1(XIV) chain**	16	6558.3	6528.3	0	11638	6455.9	0	TRUE
Q06830	-2.39013	0.002066332	TRUE	3979.667	33224	PRDX1	Peroxiredoxin-1	8	43408	39529	16735	7198.3	0	4740.7	FALSE
Q07002	0.155198	0.284420751	FALSE	3631.433	1670.13	CDK18	Cyclin-dependent kinase 18	4	2402.1	0	2608.3	6824.6	3018.4	1051.3	FALSE
Q07666-3	-0.47864	0.155990477	FALSE	21400	20119.3	KHDRBS1	KH domain-containing, RNA-binding, signal transduction-associated protein 1	4	39152	21206	0	29194	15639	19367	FALSE
Q07866	0.359216	0.185960666	FALSE	2548.933	2885.07	KLC1	Kinesin light chain 1	5	3428.4	2454.9	2771.9	4911.7	0	2735.1	FALSE
Q09666	-0.27499	0.193677644	FALSE	159810	188127	AHNAK	Neuroblast differentiation-associated protein AHNAK	81	180490	215150	168740	180200	106510	192720	FALSE
Q12860-2	-3.74918	4.46E-05	TRUE	7036.7	83744.7	CNTN1	Contactin-1	27	84913	85365	80956	11981	3733.7	5395.4	FALSE
Q12931-2	-2.22502	0.000254398	TRUE	3954.1	18417.3	TRAP1	Heat shock protein 75 kDa, mitochondrial	14	17873	17417	19962	4396.2	4053.9	3412.2	FALSE
Q12955	1.63461	0.007179951	TRUE	117576.7	37677	ANK3	Ankyrin-3	27	51424	21292	40315	66050	120110	166570	FALSE
Q12979	0.397177	0.189276971	FALSE	10666.37	8304.8	ABR	Active breakpoint cluster region-related protein	8	3953.8	7732.6	13228	17371	7881.2	6746.9	FALSE
Q13148	2.634241	0.000793273	TRUE	63902.33	12007.7	TARDBP	TAR DNA-binding protein 43	13	21836	8648.1	5538.9	57958	79261	54488	FALSE
Q13367	-2.37468	0.001040651	TRUE	641.93	5177.03	AP3B2	AP-3 complex subunit beta-2	8	5721.1	3352.2	6457.8	904.59	0	1021.2	FALSE
Q13509	1.017897	0.031872994	TRUE	261063.3	119260	TUBB3	Tubulin beta-3 chain	11	138250	118140	101390	132190	369450	281550	FALSE
Q13526	-0.46942	0.141989783	FALSE	11986.9	16731	PIN1	Peptidyl-prolyl cis-trans isomerase NIMA-interacting 1	6	13750	11969	24474	16668	8229.7	11063	FALSE
Q13554-2	1.917441	0.00075208	TRUE	497703.3	133300	CAMK2B	Calcium/calmodulin-dependent protein kinase type II subunit beta	10	136720	104390	158790	486440	456310	550360	FALSE
Q13555-6	2.588792	0.000354888	TRUE	35775.67	5505.93	CAMK2G	Calcium/calmodulin-dependent protein kinase type II subunit	6	5275.4	5490.7	5751.7	18351	46077	42899	FALSE

							gamma								
Q13596	-0.34093	0.189558427	FALSE	5753.367	6571.3	SNX1	Sorting nexin-1	10	6843.7	5639.4	7230.8	9216.7	2893.9	5149.5	FALSE
Q13884-2	1.519623	0.009684642	TRUE	3488.4	1861.43	SNTB1	Beta-1-syntrophin	4	2005.9	2350.3	1228.1	0	6167.4	4297.8	FALSE
Q13885	0.524007	0.100169192	FALSE	2597800	1836067	TUBB2A	Tubulin beta-2A chain	3	1866400	2289400	1352400	2688700	2904900	2199800	FALSE
Q14112-2	4.085232	0.001410726	TRUE	200953.3	25966	**NID2**	**Nidogen-2**	29	7211.9	3391.2	67295	207750	214480	180630	TRUE
Q14155-3	0.351649	0.184457481	FALSE	4375.5	3645.6	ARHGEF7	Rho guanine nucleotide exchange factor 7	2	3254.8	2119.4	5562.6	4225.5	5419.9	3481.1	FALSE
Q14195	-1.40704	0.015809232	TRUE	2477.7	9794.83	DPYSL3	Dihydropyrimidinase-related protein 3	9	6495.2	13098	9791.3	0	2610	4823.1	FALSE
Q14247	1.682156	0.018075609	TRUE	13200.33	4771.2	CTTN	Src substrate cortactin	8	6980.5	5992.8	1340.3	20636	9125.9	9839.1	FALSE
Q14315-2	1.089559	0.116315859	FALSE	8415.833	1626.87	FLNC	Filamin-C	13	2545.8	2334.8	0	19027	1582.7	4637.8	FALSE
Q14344-2	-0.29471	0.264172512	FALSE	2826.433	3556.39	GNA13	Guanine nucleotide-binding protein subunit alpha-13	6	9692	977.18	0	1693.1	4871.6	1914.6	FALSE
Q14576	-0.39398	0.184457481	FALSE	16355	22399.3	ELAVL3	ELAV-like protein 3	7	28676	27829	10693	12365	24055	12645	FALSE
Q14643-4	-4.14895	2.83E-05	TRUE	534.7133	14161.3	ITPR1	Inositol 1,4,5-trisphosphate receptor type 1	22	13424	11684	17376	954.89	0	649.25	FALSE
Q14966-3	5.652952	1.18E-05	TRUE	39388	933.277	ZNF638	Zinc finger protein 638	15	1205.3	299.43	1295.1	40368	45070	32726	FALSE
Q15019-2	-2.00896	0.000793273	TRUE	7619.767	30565.7	SEPT2	Septin-2	7	39228	25285	27184	9990.1	6539.1	6330.1	FALSE
Q15424	1.460956	0.010126029	TRUE	6846.233	2585.93	SAFB	Scaffold attachment factor B1	6	3566.9	2840.2	1350.7	6156.5	4879.9	9502.3	FALSE
Q15555-4	-0.68446	0.09270107	FALSE	10611.83	16985	MAPRE2	Microtubule-associated protein RP/EB family member 2	7	17696	9831.1	23428	16224	8442.6	7168.9	FALSE
Q15746-11	0.866861	0.029507761	FALSE	18780.33	10151.6	MYLK	Myosin light chain kinase, smooth muscle;Myosin light chain kinase, smooth muscle,	11	10531	8808.7	11115	23603	18097	14641	FALSE

							deglutamylated form								
Q16623	-4.03199	3.71E-05	TRUE	2328.667	58452.3	STX1A	Syntaxin-1A	12	42380	57657	75320	0	3825.3	3160.7	FALSE
Q16653	-1.68425	0.002599772	TRUE	61613	191190	MOG	Myelin-oligodendrocyte glycoprotein	9	196870	224350	152350	54673	88055	42111	FALSE
Q16658	-1.40451	0.017847575	TRUE	7149.5	26338.3	FSCN1	Fascin	14	33637	24908	20470	15209	6239.5	0	FALSE
Q1KMD3	2.63216	0.000104722	TRUE	164460	26792.7	HNRNPUL2	Heterogeneous nuclear ribonucleoprotein U-like protein 2	23	33273	24651	22454	145180	164930	183270	FALSE
Q4V328	0.865325	0.048455345	FALSE	17509.33	6258.47	GRIPAP1	GRIP1-associated protein 1	19	9516.6	9258.8	0	17201	13050	22277	FALSE
Q52LJ0-2	0.419242	0.230131689	FALSE	12870.37	5575	FAM98B	Protein FAM98B	5	12653	4072	0	25540	3691.6	9379.5	FALSE
Q5QPM1	1.311932	0.017977161	TRUE	21073	5818.1	RALY	RNA-binding protein Raly	5	6001.3	11453	0	20141	15975	27103	FALSE
Q5T4S7-3	-0.17919	0.251562845	FALSE	2138.233	1581.6	UBR4	E3 ubiquitin-protein ligase UBR4	17	2364.1	2380.7	0	2706.3	1656.6	2051.8	FALSE
Q5TH69	1.132063	0.043534799	TRUE	4259.233	1152.83	ARFGEF3	Brefeldin A-inhibited guanine nucleotide-exchange protein 3	11	1948	1510.5	0	7397.8	2460.5	2919.4	FALSE
Q5U651	4.463226	8.70E-05	TRUE	21758.67	783.273	RASIP1	Ras-interacting protein 1	8	526.82	1823	0	19128	25155	20993	FALSE
Q5VTE0	0.008291	0.314244806	FALSE	84243.33	62064.7	EEF1A1P5	Putative elongation factor 1-alpha-like 3;Elongation factor 1-alpha 1;Elongation factor 1-alpha	7	60574	73436	52184	173840	53494	25396	FALSE
Q63HR2-6	1.508703	0.005375085	TRUE	12278.33	2881.2	TNS2	Tensin-2	12	4044	4599.6	0	12289	11807	12739	FALSE
Q6H8Q1-8	0.205551	0.264172512	FALSE	903.6	900.197	ABLIM2	Actin-binding LIM protein 2	4	2016	684.59	0	0	1399.5	1311.3	FALSE
Q6NY19-2	1.494068	0.009638272	TRUE	19431.17	6383.27	KANK3	KN motif and ankyrin repeat domain-containing protein 3	9	4761.1	7675.8	6712.9	27642	9282.5	21369	FALSE
Q6UWR7	-2.54846	0.001412843	TRUE	5820.9	32937.7	ENPP6	Ectonucleotide pyrophosphatase/phosphodiesterase family member 6	9	35488	46370	16955	6967.4	7992.9	2502.4	FALSE
Q6WCQ1	2.116494	0.001070675	TRUE	13568.67	2078.23	MPRIP	Myosin phosphatase Rho-interacting protein	4	3025.9	3208.8	0	15158	12127	13421	FALSE
Q6ZMZ3-2	-	0.313165	FALSE	1240.5	2007.7	SYNE3	Nesprin-3	5	3061.9	1178.3	1783.1	0	1606.5	2115.2	FALS

	0.01282	593		67	7										E
Q7KZF4	-1.3559	0.011093412	TRUE	3113.7	11858.1	SND1	Staphylococcal nuclease domain-containing protein 1	14	12660	9665.2	13249	5536.3	0	3804.8	FALSE
Q7L099	1.901218	0.001746701	TRUE	22404	6194.53	RUFY3	Protein RUFY3	10	8504.2	6255.1	3824.3	21407	28312	17493	FALSE
Q7L0J3	-5.26407	1.78E-05	TRUE	1570.683	56184.7	SV2A	Synaptic vesicle glycoprotein 2A	7	36156	44909	87489	1534.8	643.85	2533.4	FALSE
Q7L775	0.280655	0.260581739	FALSE	4983.667	3550.57	EPM2AIP1	EPM2A-interacting protein 1	10	9159.9	1491.8	0	6935.5	2273.7	5741.8	FALSE
Q7Z406	1.156464	0.023833628	TRUE	13558.23	5686.7	MYH14	Myosin-14	21	7602.4	5053.2	4404.5	21331	7287.7	12056	FALSE
Q7Z4S6-2	0.37238	0.159276675	FALSE	9633.9	7758	KIF21A	Kinesin-like protein KIF21A	24	5481.3	6894.7	10898	9959.3	9473.7	9468.7	FALSE
Q86UP2-4	-4.75564	0.000254398	TRUE	1657.783	56721.7	KTN1	Kinectin	42	89444	28804	51917	4099	0	874.35	FALSE
Q86Y82	-0.83918	0.047799217	FALSE	2734.7	7426.7	STX12	Syntaxin-12	5	9095.2	6472.2	6712.7	4052.4	0	4151.7	FALSE
Q8IYB4-8	1.010959	0.031872994	TRUE	3693.8	1196.53	PEX5L	PEX5-related protein	6	1682.5	1907.1	0	4629.8	3721	2730.6	FALSE
Q8IYQ7	0.515422	0.184457481	FALSE	5681.367	4286.6	THNSL1	Threonine synthase-like 1	10	1835.6	2655.8	8368.4	8334.2	2194.1	6515.8	FALSE
Q8N145	-0.94522	0.049451941	FALSE	2040.767	3936.6	LGI3	Leucine-rich repeat LGI family member 3	7	6298.3	5511.5	0	2973.5	3148.8	0	TRUE
Q8N573-8	-0.14267	0.273145715	FALSE	6896	12125.6	OXR1	Oxidation resistance protein 1	16	11223	7526.8	17627	0	10385	10303	FALSE
Q8N5V2-3	0.904262	0.038017588	FALSE	6737.333	3461.5	NGEF	Ephexin-1	8	4323.5	2907.8	3153.2	3933.2	8557.8	7721	FALSE
Q8N6T3	0.865095	0.089135539	FALSE	2328	1188.47	ARFGAP1	ADP-ribosylation factor GTPase-activating protein 1	6	1519	2046.4	0	0	2120.5	4863.5	FALSE
Q8NE71-2	1.023505	0.031872994	TRUE	5841.3	1918.2	ABCF1	ATP-binding cassette sub-family F member 1	6	3407	2347.6	0	6877.6	6163.6	4482.7	FALSE
Q8NFW8	0.639421	0.10519295	FALSE	9875.433	4006.57	CMAS	N-acylneuraminate cytidylyltransferase	6	5938.5	0	6081.2	6301.2	9246.1	14079	FALSE
Q8WUM4	-0.86181	0.062926988	FALSE	4131	7921.8	PDCD6IP	Programmed cell death 6-interacting protein	11	8849.4	3809	11107	5444	2690.9	4258.1	FALSE

Q8WXD9	0.5671 5	0.129705 073	FALSE	3219.4	2179.4 3	CASKIN1	Caskin-1	5	2973	3565.3	0	0	5058.7	4599.5	FALS E
Q8WXF7-2	- 0.9059 4	0.030299 874	FALSE	6032.8	10922. 3	ATL1	Atlastin-1	9	9718.8	11465	11583	7322.2	3891.8	6884.4	FALS E
Q92556	0.7630 71	0.089346 494	FALSE	1907.8 67	1108.3 7	ELMO1	Engulfment and cell motility protein 1	5	1853.7	1471.4	0	3439.9	2283.7	0	FALS E
Q92598-2	- 0.5520 9	0.100979 171	FALSE	23691. 67	34429	HSPH1	Heat shock protein 105 kDa	20	32463	26697	44127	16510	24208	30357	FALS E
Q92752	- 2.4478 6	0.000663 846	TRUE	11722. 77	59533. 3	TNR	Tenascin-R	20	68511	64982	45107	12105	17085	5978.3	TRUE
Q96AP7	- 0.4772 5	0.188771 287	FALSE	2558.0 33	3955.3 7	ESAM	Endothelial cell-selective adhesion molecule	5	0	3079.7	8786.4	4708.7	0	2965.4	FALS E
Q96I24	2.9972 79	0.000752 08	TRUE	29763. 33	2941.6 7	FUBP3	Far upstream element-binding protein 3	10	6781.4	2043.6	0	31844	28283	29163	FALS E
Q96JH7	2.3759 08	0.001814 107	TRUE	7955.8	943	VCPIP1	Deubiquitinating protein VCIP135	12	1456	1373	0	4139.2	11207	8521.2	FALS E
Q96KR1	1.5068 9	0.009532 591	TRUE	15391. 33	5902.9 3	ZFR	Zinc finger RNA-binding protein	15	7451.7	7553.8	2703.3	14412	12742	19020	FALS E
Q96PU8-5	1.9263 59	0.009638 272	TRUE	24388	5118	QKI	Protein quaking	8	12098	0	3256	28240	17583	27341	FALS E
Q96PY5-3	- 0.4076 6	0.167550 679	FALSE	3777.9 67	7534.8 3	FMNL2	Formin-like protein 2	7	8521.5	5571.2	8511.8	0	4640.9	6693	FALS E
Q96QR8	0.1795 4	0.279288 539	FALSE	1591.3 07	717.83 3	PURB	Transcriptional activator protein Pur-beta	4	1046	1107.5	0	455.02	1333.1	2985.8	FALS E
Q99250-2	0.2465 81	0.262407 187	FALSE	4228.6 43	3088.7	SCN2A	Sodium channel protein type 2 subunit alpha;Sodium channel protein type 3 subunit alpha	8	5285.9	1580	2400.2	6638	998.73	5049.2	FALS E
Q99962	- 0.7575 4	0.092433 126	FALSE	9837.3 33	26570. 7	SH3GL2	Endophilin-A1	7	17210	20149	42353	0	11943	17569	FALS E
Q9BSJ8-2	- 1.9304 3	0.001427 345	TRUE	4878.1	17502	ESYT1	Extended synaptotagmin-1	23	16726	18025	17755	6517.8	5338.7	2777.8	FALS E
Q9BX66-9	1.2027 42	0.016383 709	TRUE	12891. 27	5141.1 3	SORBS1	Sorbin and SH3 domain-containing protein 1	11	5371.2	5166.8	4885.4	12173	6945.8	19555	FALS E
Q9BZF1-3	- 0.1839	0.255782 042	FALSE	4098.8	3299.8 7	OSBPL8	Oxysterol-binding protein-related protein 8;Oxysterol-	8	3151	0	6748.6	4891.7	3518.5	3886.2	FALS E

	8						binding protein								
Q9BZV1	-0.27312	0.253216271	FALSE	1269.133	2818.19	UBXN6	UBX domain-containing protein 6	7	2360.5	927.06	5167	1486.3	0	2321.1	FALSE
Q9C040-2	-0.59017	0.137625066	FALSE	4654.433	6786.67	TRIM2	Tripartite motif-containing protein 2	12	8254.7	8670.1	3435.2	7452.3	4219.2	2291.8	FALSE
Q9GZM7	5.55164	2.25E-06	TRUE	300740	6620.03	TINAGL1	Tubulointerstitial nephritis antigen-like	14	9436.1	6142.8	4281.2	375780	229430	297010	TRUE
Q9GZV7	-0.3151	0.189558427	FALSE	16994.33	22752.7	HAPLN2	Hyaluronan and proteoglycan link protein 2	6	33841	21591	12826	17874	17885	15224	TRUE
Q9H115	-1.91643	0.003325271	TRUE	20604	77257	NAPB	Beta-soluble NSF attachment protein	15	86101	103630	42040	13148	16479	32185	FALSE
Q9H2X9	0.919732	0.081150333	FALSE	7201.7	6509.4	SLC12A5	Solute carrier family 12 member 5	7	6481.4	10279	2767.8	9934.1	0	11671	FALSE
Q9H4G4	0.438403	0.193344118	FALSE	10920.63	10512.5	GLIPR2	Golgi-associated plant pathogenesis-related protein 1	5	19719	8958.5	2859.9	11716	12375	8670.9	FALSE
Q9H8L6	1.339923	0.005698319	TRUE	11535.67	4676.6	MMRN2	Multimerin-2	5	5943.1	4762.3	3324.4	10547	11906	12154	TRUE
Q9H936	-1.78119	0.024052043	TRUE	4998.267	19009.3	SLC25A22	Mitochondrial glutamate carrier 1	8	12652	18250	26126	2190.8	0	12804	FALSE
Q9NT62	1.249623	0.026888712	TRUE	6613.367	1731.17	ATG3	Ubiquitin-like-conjugating enzyme ATG3	5	2177.6	3015.9	0	5403	4020.1	10417	FALSE
Q9NXF1	0.05999	0.294172073	FALSE	5200.467	5162	TEX10	Testis-expressed sequence 10 protein	11	4381.5	3173.2	7931.3	3728.1	7254	4619.3	FALSE
Q9NZB2-4	-1.00882	0.022272301	TRUE	9716.633	19922	FAM120A	Constitutive coactivator of PPAR-gamma-like protein 1	20	13229	22216	24321	8446.7	8535.2	12168	FALSE
Q9NZN3	0.427632	0.15989524	FALSE	5511.233	2736.6	EHD3	EH domain-containing protein 3	3	4931.2	3278.6	0	5523	4213.6	6797.1	FALSE
Q9NZN4	-0.38427	0.167598281	FALSE	11618.97	14098.7	EHD2	EH domain-containing protein 2	16	13505	12906	15885	18277	9060.2	7519.7	FALSE
Q9NZR1	-0.53185	0.15611871	FALSE	3666.4	5036.83	TMOD2	Tropomodulin-2	5	0	8507.3	6603.2	0	3663.5	7335.7	FALSE
Q9NZW5	0.729574	0.081991778	FALSE	4395.767	2586.57	MPP6	MAGUK p55 subfamily member 6	3	3215.8	2927	1616.9	2413	4870.7	5903.6	FALSE
Q9P121-3	-	0.220382	FALSE	3059.6	5835.4	NTM	Neurotrimin	5	4157.4	3528.4	9820.4	0	6617.1	2561.9	FALS

	0.34845	689		67											E
Q9P1Z2-2	0.804394	0.076762626	FALSE	2005.6	1680.3	CALCOCO1	Calcium-binding and coiled-coil domain-containing protein 1	5	2133.7	1173.2	1734	3975.2	2041.6	0	FALSE
Q9UBB6-2	-1.13669	0.020251987	TRUE	17810	40500.3	NCDN	Neurochondrin	16	42542	23418	55541	13596	16198	23636	FALSE
Q9UBT2	1.920774	0.000996826	TRUE	16827.33	4526.5	UBA2	SUMO-activating enzyme subunit 2	12	6003.9	3421.2	4154.4	16825	14017	19640	FALSE
Q9UEW8-2	-0.63189	0.093513941	FALSE	8863.4	13363.3	STK39	STE20/SPS1-related proline-alanine-rich protein kinase	9	18257	10208	11625	5437.9	12763	8389.3	FALSE
Q9UH99	-0.08201	0.286230994	FALSE	29396	31404.3	SUN2	SUN domain-containing protein 2	19	32330	21714	40169	22624	27919	37645	FALSE
Q9UHD8	0.085882	0.294172073	FALSE	3964.567	3260.73	SEPT9	Septin-9	11	2874.3	1860.3	5047.6	4624.8	1138	6130.9	FALSE
Q9UHG2	-0.76101	0.137625066	FALSE	10495.03	20425	PCSK1N	ProSAAS;KEP;Big SAAS;Little SAAS;Big PEN-LEN;PEN;Little LEN;Big LEN	7	14181	18316	28778	0	5032.1	26453	FALSE
Q9UJU6	0.788909	0.103590793	FALSE	9214.033	6606.83	DBNL	Drebrin-like protein	7	2471.5	4253	13096	10781	6146.1	10715	FALSE
Q9UK22	-0.23028	0.221478363	FALSE	10154.4	12324.3	FBXO2	F-box only protein 2	9	13899	15457	7616.9	8648.5	9582.7	12232	FALSE
Q9ULU8	0.138592	0.276742917	FALSE	7829.933	7115.9	CADPS	Calcium-dependent secretion activator 1	14	4562	5262.7	11523	9361.9	10301	3826.9	FALSE
Q9UNW9	1.314864	0.022512609	TRUE	20955.67	8418.73	NOVA2	RNA-binding protein Nova-2	8	4631.3	7151.9	13473	11595	17598	33674	FALSE
Q9UNZ2	0.254178	0.221478363	FALSE	4717.3	2575.93	NSFL1C	NSFL1 cofactor p47	8	4250.6	3477.2	0	3959.6	3821.2	6371.1	FALSE
Q9UPA5	2.045953	0.011322243	TRUE	28153.67	6646.87	BSN	Protein bassoon	32	2557.1	5271.5	12112	10440	20623	53398	FALSE
Q9UPQ0	0.954632	0.036475954	FALSE	9818.633	5191.9	LIMCH1	LIM and calponin homology domains-containing protein 1	16	4980.6	7392.4	3202.7	10912	6569.9	11974	FALSE
Q9UPV7	1.124741	0.011322243	TRUE	4028.933	1828.93	KIAA1045	Protein KIAA1045	4	1705.8	1632.7	2148.3	3172.8	5014.8	3899.2	FALSE
Q9UQB8	0.044428	0.298497235	FALSE	3804.833	3749.7	BAIAP2	Brain-specific angiogenesis inhibitor 1-associated protein 2	7	3954.7	2393	4901.4	2711.4	3935.8	4767.3	FALSE
Q9Y233-2	-2.49711	0.002839319	TRUE	10450.7	60309	PDE10A	cAMP and cAMP-inhibited cGMP 3,5-cyclic phosphodiesterase 10A	23	112380	27545	41002	19086	5322.8	6943.3	FALSE

Q9Y2Q0-3	-3.6945	0.000524852	TRUE	1221.433	27539.3	ATP8A1	Phospholipid-transporting ATPase IA	22	48791	22827	11000	2263.6	0	1400.7	FALSE
Q9Y3F4-2	0.417449	0.150727095	FALSE	13274	10289.9	STRAP	Serine-threonine kinase receptor-associated protein	9	6484.6	14016	10369	16178	10830	12814	FALSE
Q9Y3I0	0.280812	0.274095479	FALSE	6829.5	9541.23	RTCB	tRNA-splicing ligase RtcB homolog	11	15725	11371	1527.7	16787	0	3701.5	FALSE
Q9Y3Z3	1.818126	0.009049607	TRUE	40543.33	14257.1	SAMHD1	Deoxynucleoside triphosphate triphosphohydrolase SAMHD1	20	10481	5135.3	27155	38101	49659	33870	FALSE
Q9Y490	-0.42896	0.139030247	FALSE	81522.33	107370	TLN1	Talin-1	61	128440	93710	99960	90541	54990	99036	FALSE
Q9Y4E6-2	-0.13454	0.262407187	FALSE	4024.533	4474.03	WDR7	WD repeat-containing protein 7	12	3733.7	3700	5988.4	4875	3243.6	3955	FALSE
Q9Y4G6	-0.05519	0.294172073	FALSE	4354.833	4593.27	TLN2	Talin-2	23	3354.5	3943.9	6481.4	3178	5559.1	4327.4	FALSE
Q9Y4L1	-2.31938	0.000430087	TRUE	6979.1	34634.3	HYOU1	Hypoxia up-regulated protein 1	22	37625	24895	41383	6913.7	4993.3	9030.3	FALSE
Q9Y5S2	1.065566	0.047314512	TRUE	4745.333	1921.23	CDC42BPB	Serine/threonine-protein kinase MRCK beta	10	1719.5	1524.2	2520	3695	1895.6	8645.4	FALSE
Q9Y6D5	1.406412	0.024516457	TRUE	3723.667	840.667	ARFGEF2	Brefeldin A-inhibited guanine nucleotide-exchange protein 2	8	1114	1408	0	5221.3	1617.6	4332.1	FALSE
Q9Y6V0-2	0.773716	0.155157913	FALSE	3591.4	911.867	PCLO	Protein piccolo	13	1312.7	1422.9	0	1249.1	1231.4	8293.7	FALSE
V9GYM8	-0.18832	0.24499215	FALSE	3054.833	3655.17	ARHGEF2	Rho guanine nucleotide exchange factor 2	8	4146.8	2130.6	4688.1	3356.2	3206.8	2601.5	FALSE
A0A087WTA8	-	-	-	186813.3	0	**COL1A2**	**Collagen alpha-2(I) chain**	4	0	0	0	263490	296950	0	TRUE
A0A087X0K0	-	-	-	13559	0	**COL15A1**	**Collagen alpha-1(XV) chain;Restin;Restin-2;Restin-3;Restin-4**	5	0	0	0	12989	12905	14783	TRUE
A0A0A0MSA0	-	-	-	3422.733	0	**LAMA3**	**Laminin subunit alpha-3**	10	0	0	0	6706.2	0	3562	TRUE
A6NEM2	-	-	-	2965.367	0	HCFC1	Host cell factor 1	4	0	0	0	0	3374.7	5521.4	TRUE
P15502-9	-	-	-	12173	0	ELN	Elastin	6	0	0	0	24014	0	12505	TRUE
P07942	-	-	-	17053	0	**LAMB1**	**Laminin subunit beta-1**	12	0	0	0	14791	20252	16116	TRUE
Q6PCB0	-	-	-	30382	0	VWA1	von Willebrand factor A domain-	7	0	0	0	28213	45055	17878	TRUE

							containing protein 1								
O00182-3	-	-	-	6289.4	0	LGALS9	Galectin-9	3	0	0	0	7738.9	6518.1	4611.2	TRUE
O95428-6	-	-	-	1782.3	0	PAPLN	Papilin	5	0	0	0	1904.1	3442.8	0	TRUE
P02452	-	-	-	58202	0	**COL1A1**	**Collagen alpha-1(I) chain**	3	0	0	0	133390	25757	15459	TRUE
P02462-2	-	-	-	59212	0	**COL4A1**	**Collagen alpha-1(IV) chain;Arresten**	7	0	0	0	28528	95482	53626	TRUE
P07585	-	-	-	7805	0	DCN	Decorin	4	0	0	0	12148	11267	0	TRUE
P25391	-	-	-	48628.67	0	**LAMA1**	**Laminin subunit alpha-1**	22	0	0	0	64366	56808	24712	TRUE
P39060-2	-	-	-	63895	0	**COL18A1**	**Collagen alpha-1(XVIII) chain;Endostatin**	11	0	0	0	80237	58766	52682	TRUE
Q9Y6C2	-	-	-	27430.33	0	EMILIN1	EMILIN-1	11	0	0	0	26006	33971	22314	TRUE
Q9Y6N6	-	-	-	15468.67	0	**LAMC3**	**Laminin subunit gamma-3**	7	0	0	0	15412	20954	10040	TRUE

Mouse proteins quantified without HpH fractionation

protein	log2fc	q.mod	Significant	ECM mean	cellular mean	gene	description	Unique peptides	cellular 1	cellular 2	cellular 3	ECM1	ECM2	ECM3	ECM marker
A0A1L1SUX8	-0.19544	0.708086	FALSE	2367033	2620800	Thy1	Thy-1 membrane glycoprotein	5	2260900	3207900	2393600	3306100	1576300	2218700	FALSE
A0A1W2P6G5	0.829308	0.173439	FALSE	621246.7	385066.7	Myl6	Myosin light polypeptide 6	5	330310	824890	0	0	841540	1022200	FALSE
A0A2I3BRL8	1.856545	0.000118	TRUE	8070467	2248367	Rbmxl1	RNA binding motif protein, X-linked-like-1;RNA-binding motif protein, X chromosome;RNA-binding motif protein, X chromosome, N-terminally processed	6	1677200	2447000	2620900	7232500	9688500	7290400	FALSE
A0A338P6J0	2.192679	5.28E-05	TRUE	211926.3	33487.33	Dlg2	Disks large homolog 2	15	81984	18478	0	83429	369720	182630	FALSE
A0A338P6K2	2.306683	2.71E-06	TRUE	5867333	1174203	Myh11		2	919510	1329600	1273500	4258700	6184200	7159100	FALSE

A1BN54	-4.91104	5.01E-19	TRUE	154919	4316600	Actn1		2	4570500	3387000	4992300	249830	93687	121240	FALSE
B2RTM0	4.612137	1.61E-17	TRUE	3.12E+08	12758000	Hist2h4	Histone H4	10	9786000	14500000	13988000	2.68E+08	3.91E+08	2.77E+08	FALSE
B9EHJ3	-2.07581	0.000118	TRUE	126576.7	788393.3	Tjp1	Tight junction protein ZO-1	27	882910	482510	999760	0	254660	125070	FALSE
F6SEU4	2.809345	4.19E-07	TRUE	1153040	114712.7	Syngap1	Ras/Rap GTPase-activating protein SynGAP	29	244150	99988	0	842150	1698600	918370	FALSE
F8VQJ3	4.159954	3.07E-15	TRUE	14420333	815643.3	**Lamc1**	**Laminin subunit gamma-1**	37	582280	868270	996380	12383000	17885000	12993000	TRUE
G3X956	2.29234	2.93E-06	TRUE	1082223	176006.7	Supt16	FACT complex subunit SPT16	23	196420	123690	207910	283070	1795300	1168300	FALSE
O08553	-5.91855	6.26E-21	TRUE	75003.33	7002300	Dpysl2	Dihydropyrimidinase-related protein 2	21	9515600	5931100	5560200	0	106540	118470	FALSE
P03995	1.277003	0.007779	TRUE	31132333	13461667	Gfap	Glial fibrillary acidic protein	24	8242000	14959000	17184000	31131000	30279000	31987000	FALSE
P06837	-1.83601	0.000637	TRUE	142983.3	837826.7	Gap43	Neuromodulin	9	422540	1354300	736640	0	257980	170970	FALSE
P08553	-0.35264	0.495518	FALSE	4769400	6004267	Nefm	Neurofilament medium polypeptide	38	3835900	7676700	6500200	7076900	3900900	3330400	FALSE
P10493	4.519724	4.56E-17	TRUE	4246133	203306	**Nid1**	**Nidogen-1**	25	204610	89048	316260	2943000	5492900	4302500	TRUE
P12960	-2.72135	5.53E-08	TRUE	165770	1108690	Cntn1	Contactin-1	29	1427200	1125800	773070	197580	173140	126590	FALSE
P18872	-2.70581	6.06E-08	TRUE	1086317	6681700	Gnao1	Guanine nucleotide-binding protein G(o) subunit alpha	18	8300900	5272900	6471300	600150	1589800	1069000	FALSE
P21619	-2.07133	2.08E-05	TRUE	1578700	6556967	Lmnb2	Lamin-B2	24	4889600	7143300	7638000	1761800	1901600	1072700	FALSE
P46660	-2.34038	2.19E-06	TRUE	913846.7	4127300	Ina	Alpha-internexin	7	4051800	4036000	4294100	1561400	634080	546060	FALSE
P60202	-4.6202	1.61E-17	TRUE	1355887	33321667	Plp1	Myelin proteolipid protein	6	22541000	40533000	36891000	1377900	1750300	939460	FALSE

	4														
P99024	-0.00335	0.994145	FALSE	1180030	1071250	Tubb5	Tubulin beta-5 chain	5	1017000	573250	1623500	616840	2244300	678950	FALSE
Q00PI9	-0.15219	0.760072	FALSE	3528033	3883400	Hnrnpul2	Heterogeneous nuclear ribonucleoprotein U-like protein 2	27	3060700	3799300	4790200	3538500	4491700	2553900	FALSE
Q3TYL9	-4.47302	6.59E-17	TRUE	827070	15261667	Cnp	2,3-cyclic-nucleotide 3-phosphodiesterase	23	14675000	16656000	14454000	264010	1193800	1023400	FALSE
Q3UHK5	-7.02637	6.38E-30	TRUE	319700	37556333	Atp1a2	Sodium/potassium-transporting ATPase subunit alpha-2	25	36980000	44091000	31598000	470470	345600	143030	FALSE
Q3V2C6	-0.30939	0.543724	FALSE	150346.7	181413.3	Des	Desmin	8	187010	192960	164270	129780	203040	118220	FALSE
Q61292	5.234415	1.00E-20	TRUE	17921333	512680	**Lamb2**	**Laminin subunit beta-2**	39	251340	714280	572420	14916000	22542000	16306000	TRUE
Q62261	-4.50048	5.17E-17	TRUE	786213.3	17635000	Sptbn1	Spectrin beta chain, non-erythrocytic 1	112	18943000	16081000	17881000	846460	885640	626540	FALSE
Q6S388	-3.24469	1.79E-10	TRUE	375010	3491267	Plec	Plectin	109	2818400	3262800	4392600	406820	470300	247910	FALSE
Q7TMM9	-1.16963	0.01463	TRUE	2907733	6693500	Tubb2a	Tubulin beta-2A chain	3	9103700	5399600	5577200	3161300	3120900	2441000	FALSE
Q80TQ3	-2.38076	1.29E-05	TRUE	255393.3	1973100	Nefh	Neurofilament heavy polypeptide	21	2208400	1954100	1756800	450030	316150	0	FALSE
Q8K310	-1.46738	0.002231	TRUE	3856700	10816767	Matr3	Matrin-3	15	7537300	11320000	13593000	3125500	4749100	3695500	FALSE
Q8VDD5	0.491418	0.339451	FALSE	4911133	3424000	Myh9	Myosin-9	56	3313800	3016300	3941900	3543900	6235900	4953600	FALSE
Q921L4	5.552269	3.45E-22	TRUE	1.15E+08	2530467	LOC665622	Histone H2B;Histone H2B type 1-P;Histone H2B type 1-K;Histone H2B type 1-C/E/G;Histone H2B	3	1644500	2739400	3207500	1E+08	1.15E+08	1.29E+08	FALSE

							type 2-B;Histone H2B type 1-H;Histone H2B type 1-B;Histone H2B type 1-M;Histone H2B type 1-F/J/L								
Q9CQ19	2.578336	2.71E-06	TRUE	875150	100793.3	Myl9	Myosin regulatory light polypeptide 9	3	109560	0	192820	775910	814640	1034900	FALSE
Q9ERD7	-0.51818	0.361202	FALSE	371370	807976.7	Tubb3	Tubulin beta-3 chain	10	898840	919550	605540	0	611510	502600	FALSE
J3QQ16	-	-	-	442896.7	0	**Col6a3**		36	0	0	0	332320	584980	411390	TRUE
B2RQQ8	-	-	-	10527933	0	**Col4a2**	**Collagen alpha-2(IV) chain;Canstatin**	7	0	0	0	8476900	15042000	8064900	TRUE
Q3UHL7	-	-	-	154548	0	**Lamb1**	**Laminin subunit beta-1**	14	0	0	0	51504	220090	192050	TRUE
E9PZ16	-	-	-	10169400	0	**Hspg2**	**Basement membrane-specific heparan sulfate proteoglycan core protein;Endorepellin;LG3 peptide**	67	0	0	0	7728200	12719000	10061000	TRUE
P39061	-	-	-	682243.3	0	**Col18a1**	**Collagen alpha-1(XVIII) chain;Endostatin**	8	0	0	0	503090	824350	719290	TRUE
H3BJ97	-	-	-	1730770	0	Tinagl1	Tubulointerstitial nephritis antigen-like	9	0	0	0	826010	2497000	1869300	TRUE
Q8R5G0	-	-	-	2405733	0	**Nid2**	**Nidogen-2**	20	0	0	0	2118800	2760900	2337500	TRUE
P11087	-	-	-	1802287	0	**Col1a1**	**Collagen alpha-1(I) chain**	3	0	0	0	697360	2157000	2552500	TRUE
P21981	-	-	-	144186.7	0	Tgm2	Protein-glutamine gamma-glutamyltransferase 2	9	0	0	0	0	105940	326620	TRUE
P97927	-	-	-	657060	0	**Lama4**	**Laminin subunit alpha-4**	20	0	0	0	334290	938880	698010	TRUE
Q04857	-	-	-	328756.7	0	**Col6a1**	**Collagen alpha-1(VI) chain**	8	0	0	0	118350	574010	293910	TRUE
Q60675	-	-	-	180704	0	**Lama2**	**Laminin subunit**	43	0	0	0	335040	344750	163860	TRUE

				7			**alpha-2**						0	0	
Q61001	-	-	-	4489600	0	**Lama5**	**Laminin subunit alpha-5**	37	0	0	0	1921600	7519600	4027600	TRUE
Q8BJD1	-	-	-	186036.7	0	Itih5	Inter-alpha-trypsin inhibitor heavy chain H5	13	0	0	0	0	398620	159490	TRUE
Q8R2Z5	-	-	-	364753.3	0	Vwa1	von Willebrand factor A domain-containing protein 1	8	0	0	0	0	661430	432830	TRUE

Human proteins quantified without HpH fractionation

protein	log2fc	q.mod	Significant	ECM mean	cellular mean	gene	description	Unique peptides	cellular r1	cellular r2	cellular r3	ECM1	ECM2	ECM3	ECM marker
A0A087WUK2	-1.42405778	0.06136665	FALSE	9550.533	36100	HNRNPDL	Heterogeneous nuclear ribonucleoprotein D-like	10	24986	46655	36659	0	20282	8369.6	FALSE
A0A087WXM8	1.791395727	0.06353742	FALSE	10846.167	4330.833	BCAM	Basal cell adhesion molecule	11	4926.1	5836.6	2229.8	24799	7739.5	0	FALSE
A0A087WY00	-1.444686899	0.15070923	FALSE	2505.967	12895.133	MYO5A	Unconventional myosin-Va	36	5067.7	7392.7	26225	0	4648.6	2869.3	FALSE
A0A087X2E3	-0.759794707	0.68086845	FALSE	5125.533	27620.333	SYN2	Synapsin-2	17	1644	11109	70108	0	3447.6	11929	FALSE
A0A1C7CYX9	-2.850832616	0.00086448	TRUE	89859	630443.333	DPYSL2	Dihydropyrimidinase-related protein 2	28	563490	573740	754100	60583	118690	90304	FALSE
C9JEU5	3.105139235	0.01741301	TRUE	107384.67	6990.167	FGG	Fibrinogen gamma chain	15	0	13908	7062.5	37349	203000	81805	TRUE
E7ES33	-3.055927944	0.00086448	TRUE	6233.567	77526.333	SEPT7	Septin-7	20	73362	83374	75843	10234	8466.7	0	FALSE
E7ESP9	-0.877093369	0.29212529	FALSE	273340	447916.667	NEFM	Neurofilament medium polypeptide	40	471950	651470	220330	180600	523850	115570	FALSE
E9PIM6	-	0.003370	TRUE	21567.3	100271.66	THY1	Thy-1 membrane	5	13953	60715	10057	18858	20899	24945	FALSE

	2.1457744 26	1		33	7		glycoprotein		0		0				
E9PR44	- 0.9637171 12	0.298333 03	FALSE	62420	91517	CRYAB	Alpha-crystallin B chain	11	10721 0	96470	70871	13714	104800	68746	FALSE
F5H7S3	- 1.0264748 29	0.218943 31	FALSE	2156.2	4754.467	TPM1	Tropomyosin alpha-1 chain	2	3954.4	10309	0	2436.5	0	4032.1	FALSE
F8VPF3	- 0.2963575 85	0.562128 4	FALSE	44088	51399.667	MYL6	Myosin light polypeptide 6	2	62061	43600	48538	40338	64792	27134	FALSE
G3V1L9	3.0893321 4	0.002374 27	TRUE	70736.3 33	9389.9	TJP1	Tight junction protein ZO-1	34	3844.7	13523	10802	83069	63515	65625	FALSE
I3L0N3	- 0.2603037	0.447435 92	FALSE	143056. 67	171300	NSF	Vesicle-fusing ATPase	30	17263 0	17242 0	16885 0	13827 0	146620	14428 0	FALSE
J3QRS3	0.9407931 11	0.030867 24	FALSE	169710	87846.333	MYL12A	Myosin regulatory light chain 12A;Myosin regulatory light chain 12B	4	88899	92772	81868	19664 0	167440	14505 0	FALSE
O00468-6	2.2117863 92	0.002695 95	TRUE	219650	46970.667	AGRN	Agrin;Agrin N-terminal 110 kDa subunit;Agrin C-terminal 110 kDa subunit;Agrin C-terminal 90 kDa fragment;Agrin C-terminal 22 kDa fragment	45	34092	56137	50683	24732 0	263870	14776 0	TRUE
O43491-4	- 1.3338715 93	0.038179 95	TRUE	6601.13 3	25706.667	EPB41L2	Band 4.1-like protein 2	18	32620	17060	27440	0	10988	8815.4	FALSE
O60506-2	- 0.7170008 51	0.218943 31	FALSE	11944.3 33	27551.333	SYNCRIP	Heterogeneous nuclear ribonucleoprotein Q	15	25102	25917	31635	0	11355	24478	FALSE
O94905	- 1.1604964 84	0.036041 04	TRUE	17650	37203.667	ERLIN2	Erlin-2	12	37516	39783	34312	13735	26724	12491	FALSE
O95810	1.2503390 82	0.057887 13	FALSE	32301.3 33	14334.867	SDPR	Serum deprivation-response protein	7	7169.6	19340	16495	42424	31258	23222	FALSE
P02794	- 0.4245699	0.471940 34	FALSE	30169.6 67	42529.667	FTH1	Ferritin heavy chain;Ferritin heavy	10	56062	21194	50333	41861	23443	25205	FALSE

	22						chain, N-terminally processed;Ferritin							
P04275	-1.124627745	0.15438781	FALSE	12179.833	21835.667	VWF	von Willebrand factor;von Willebrand antigen 2	46	24619	21817	19071	16829	16053	3657.5	TRUE
P04406	-6.585378962	0.00021823	TRUE	9572.1	780483.333	GAPDH	Glyceraldehyde-3-phosphate dehydrogenase	15	667220	672530	1001700	3345.7	15718	9652.6	FALSE
P04899	-2.728396325	0.00144617	TRUE	7962.367	50241.333	GNAI2	Guanine nucleotide-binding protein G(i) subunit alpha-2	9	39834	53540	57350	5823.7	12100	5963.4	FALSE
P07196	-0.357758541	0.66304856	FALSE	311610	328550	NEFL	Neurofilament light polypeptide	36	325800	465030	194820	198800	622720	113310	FALSE
P09471-2	-3.268749826	0.00082721	TRUE	32237.667	298040	GNAO1	Guanine nucleotide-binding protein G(o) subunit alpha	2	351230	233280	309610	34060	43552	19101	FALSE
P09543-2	-1.051443755	0.0241632	TRUE	752743.33	1550533.333	CNP	2,3-cyclic-nucleotide 3-phosphodiesterase	29	1727100	1506900	1417600	684310	904220	669700	FALSE
P11021	-4.625455452	0.00044964	TRUE	9952.367	215803.333	HSPA5	78 kDa glucose-regulated protein	24	226240	187030	234140	7376.1	17326	5155	FALSE
P11047	4.658959995	0.00021823	TRUE	538743.33	21326.667	**LAMC1**	**Laminin subunit gamma-1**	38	14102	27411	22467	590410	673870	351950	TRUE
P11216	-1.405144859	0.03899092	TRUE	7011.8	20552.633	PYGB	Glycogen phosphorylase, brain form	25	23810	9257.9	28590	6161.9	6936.8	7936.7	FALSE
P12036-2	0.866027738	0.40978659	FALSE	23035	24456.3	NEFH	Neurofilament heavy polypeptide	15	18197	48104	7067.9	43173	25932	0	FALSE
P12277	-0.290092201	0.62906401	FALSE	545080	625000	CKB	Creatine kinase B-type	20	852430	503860	518710	771670	262960	600610	FALSE
P12814-3	-1.587049586	0.00526443	TRUE	22935.333	68646.667	ACTN1	Alpha-actinin-1	25	55684	72050	78206	28396	21082	19328	FALSE
P14136-2	-2.857850861	0.00396903	TRUE	34386	236616.667	GFAP	Glial fibrillary acidic protein	18	267980	303910	137960	39506	48147	15505	FALSE
P16152	-1.476048001	0.15863143	FALSE	19782.8	60767.333	CBR1	Carbonyl reductase [NADPH] 1	11	81408	38677	62217	50762	8586.4	0	FALSE

P17600	-1.043023822	0.13939595	FALSE	26643	57726	SYN1	Synapsin-1	17	47415	30165	95598	14860	35135	29934	FALSE
P17858	-0.168971642	0.73612986	FALSE	10884.8	8528.167	PFKL	ATP-dependent 6-phosphofructokinase, liver type	16	16958	0	8626.5	11200	12702	8752.4	FALSE
P18206-2	-3.185532101	0.002282	TRUE	1982.533	27622.333	VCL	Vinculin	34	17337	34925	30605	3592.8	0	2354.8	FALSE
P24844	1.515216462	0.0779743	FALSE	43720.667	8891	MYL9	Myosin regulatory light polypeptide 9	3	15320	0	11353	77132	32954	21076	FALSE
P35221	0.299400624	0.62906401	FALSE	6492.467	7848.067	CTNNA1	Catenin alpha-1	16	5353.4	7149.8	11041	6647.4	0	12830	FALSE
P35579	1.163998375	0.01837727	TRUE	699756.67	307740	MYH9	Myosin-9	73	303240	331540	288440	530310	771420	797540	FALSE
P35609	-1.735312296	0.02888155	TRUE	6183.633	33154.667	ACTN2	Alpha-actinin-2	2	17912	36337	45215	0	9053.7	9497.2	FALSE
P38405	0.262387622	0.70932101	FALSE	8302.733	4063.867	GNAL	Guanine nucleotide-binding protein G(olf) subunit alpha	9	6216	0	5975.6	3385.2	10123	11400	FALSE
P38606	-4.952763343	0.00021823	TRUE	1356.933	62044.667	ATP6V1A	V-type proton ATPase catalytic subunit A	26	55862	61455	68817	0	2433.2	1637.6	FALSE
P40123	-0.230935936	0.62906401	FALSE	6788.167	5344.2	CAP2	Adenylyl cyclase-associated protein 2;Adenylyl cyclase-associated protein	9	6740.4	0	9292.2	6173	6275.5	7916	FALSE
P45974-2	1.643337897	0.02711968	TRUE	58060	20767.533	USP5	Ubiquitin carboxyl-terminal hydrolase 5	18	21626	9307.6	31369	49652	62620	61908	FALSE
P46821	-1.985932957	0.00834604	TRUE	31966.333	127114.333	MAP1B	Microtubule-associated protein 1B;MAP1B heavy chain;MAP1 light chain LC1	64	81233	116760	183350	46437	27749	21713	FALSE
P50148	-3.122152507	0.00599241	TRUE	5229.567	33088.333	GNAQ	Guanine nucleotide-binding protein G(q) subunit alpha	7	34004	33422	31839	1886.6	11210	2592.1	FALSE
P55209	-0.323757719	0.76715474	FALSE	7397.467	8816	NAP1L1	Nucleosome assembly protein 1-like 1	5	11429	4499	10520	0	2110.4	20082	FALSE
P55268	5.6865980	4.82E-05	TRUE	786706.	15048.333	**LAMB2**	**Laminin subunit beta-**	44	12488	16475	16182	83013	958130	57186	TRUE

	48			67			**2**					0		0	
P60201-2	-3.941538546	0.00062298	TRUE	162456.67	2522300	PLP1	Myelin proteolipid protein	5	2605300	3447100	1514500	161260	220790	105320	FALSE
P63261	-0.925262995	0.03086724	FALSE	1005676.7	1902200	ACTG1	Actin, cytoplasmic 2;Actin, cytoplasmic 2, N-terminally processed;Actin, cytoplasmic 1;Actin, cytoplasmic 1, N-terminally processed	4	2050800	1879500	1776300	1159600	947980	909450	FALSE
P84077	-2.820362875	0.01089711	TRUE	8630.267	43561.333	ARF1	ADP-ribosylation factor 1;ADP-ribosylation factor 3	4	45259	39780	45645	4024.7	18781	3085.1	FALSE
Q01484	-2.95349594	0.00310493	TRUE	3131.8	37898.333	ANK2	Ankyrin-2	62	28084	28448	57163	5578.3	3817.1	0	FALSE
Q01813	0.237619049	0.68086845	FALSE	65601.333	58519.667	PFKP	ATP-dependent 6-phosphofructokinase, platelet type	20	32423	49779	93357	41716	87182	67906	FALSE
Q03252	-2.863098704	0.00066027	TRUE	25581.333	180743.333	LMNB2	Lamin-B2	25	180980	187500	173750	32991	25694	18059	FALSE
Q06830	-3.587871144	0.0045056	TRUE	7110.033	107550	PRDX1	Peroxiredoxin-1	8	120610	126810	75230	16813	0	4517.1	FALSE
Q09666	-2.021517258	0.03408418	TRUE	25055.5	83001.667	AHNAK	Neuroblast differentiation-associated protein AHNAK	81	62296	95057	91652	37420	30684	7062.5	FALSE
Q12860-2	-3.81959247	0.00144617	TRUE	2459.433	46736	CNTN1	Contactin-1	27	51190	40145	48873	5353.9	2024.4	0	FALSE
Q13148	2.890550684	0.00123776	TRUE	38266	4838.933	TARDBP	TAR DNA-binding protein 43	13	5228.9	4700.6	4587.3	21709	39582	53507	FALSE
Q13509	0.985005962	0.02898096	FALSE	178153.33	90041	TUBB3	Tubulin beta-3 chain	11	90137	77936	102050	154430	179610	200420	FALSE
Q13554-2	2.11879733	0.00599241	TRUE	417346.67	95066.667	CAMK2B	Calcium/calmodulin-dependent protein kinase type II subunit beta	10	109810	58230	117160	285830	346560	619650	FALSE

Q13885	0.9814713	0.044215	FALSE	3769233.3	1845733.333	TUBB2A	Tubulin beta-2A chain	3	1794000	1878300	1864900	2490300	4519200	4298200	FALSE
Q14576	-2.108992538	0.00144617	TRUE	6761.4	29478.333	ELAVL3	ELAV-like protein 3	7	28511	35954	23970	7245.6	7028.6	6010	FALSE
Q15019-2	-2.195844042	0.0045056	TRUE	1813.033	12714.267	SEPT2	Septin-2	7	15527	9227.8	13388	0	2516.7	2922.4	FALSE
Q16653	-3.145562066	0.03086724	TRUE	17588.367	140840	MOG	Myelin-oligodendrocyte glycoprotein	9	140080	173540	108900	0	47635	5130.1	FALSE
Q1KMD3	2.016392236	0.00292964	TRUE	123936.33	30411	HNRNPUL2	Heterogeneous nuclear ribonucleoprotein U-like protein 2	23	33645	23033	34555	91569	156510	123730	FALSE
Q5QPM1	0.817759876	0.10822682	FALSE	27844.667	10375	RALY	RNA-binding protein Raly	5	0	15937	15188	33497	21820	28217	FALSE
Q5VTE0	1.165868846	0.15438781	FALSE	63186.667	29504.333	EEF1A1P5	Putative elongation factor 1-alpha-like 3;Elongation factor 1-alpha 1;Elongation factor 1-alpha	7	42241	35004	11268	73002	86891	29667	FALSE
Q7L099	-0.087573521	0.86652685	FALSE	5509	8462.067	RUFY3	Protein RUFY3	10	7021.6	6681.6	11683	5269	11258	0	FALSE
Q92598-2	-1.521236	0.08818008	FALSE	6580.2	16012	HSPH1	Heat shock protein 105 kDa	20	16104	10363	21569	8284.9	9527.4	1928.3	FALSE
Q9H4G4	-1.231112642	0.11059615	FALSE	3709.867	8236	GLIPR2	Golgi-associated plant pathogenesis-related protein 1	5	0	14255	10453	0	7546.5	3583.1	FALSE
Q9NZN4	0.43574908	0.33986654	FALSE	10144.567	5074.633	EHD2	EH domain-containing protein 2	16	6272.6	0	8951.3	9570.7	10261	10602	FALSE
Q9UH99	0.492992205	0.44743592	FALSE	13331.333	14816.967	SUN2	SUN domain-containing protein 2	19	18941	8157.9	17352	0	15800	24194	FALSE
Q9UNW9	1.411990886	0.11102768	FALSE	9370.667	4003.267	NOVA2	RNA-binding protein Nova-2	8	3115.3	0	8894.5	0	12890	15222	FALSE
Q9Y490	-2.521461539	0.00144617	TRUE	9010.533	51415.333	TLN1	Talin-1	61	37567	53433	63246	10885	6186.7	9959.9	FALSE
A0A0A0MTC7	-	-	-	23367.333	0	**LAMA4**	**Laminin subunit alpha-4**	19	0	0	0	18394	33205	18503	TRUE

P07942	-	-	-	12741.6	0	**LAMB1**	**Laminin subunit beta-1**	12	0	0	0	4068.5	26218	7938.3	TRUE
O15230	-	-	-	253880	0	**LAMA5**	**Laminin subunit alpha-5**	51	0	0	0	261420	330580	169640	TRUE
P02452	-	-	-	21680.333	0	**COL1A1**	**Collagen alpha-1(I) chain**	3	0	0	0	56267	8774	0	TRUE
P02462-2	-	-	-	59615	0	**COL4A1**	**Collagen alpha-1(IV) chain;Arresten**	7	0	0	0	78956	53348	46541	TRUE
P02671	-	-	-	27617	0	FGA	Fibrinogen alpha chain;Fibrinopeptide A;Fibrinogen alpha chain	11	0	0	0	0	48293	34558	TRUE
P02751	-	-	-	146967.67	0	**FN1**	**Fibronectin;Anastellin;Ugl-Y1;Ugl-Y2;Ugl-Y3**	37	0	0	0	71933	268790	100180	TRUE
P08572	-	-	-	566533.33	0	**COL4A2**	**Collagen alpha-2(IV) chain;Canstatin**	11	0	0	0	642010	661940	395650	TRUE
P21810	-	-	-	14732.333	0	BGN	Biglycan	7	0	0	0	28863	15334	0	TRUE
P25391	-	-	-	11438.6	0	**LAMA1**	**Laminin subunit alpha-1**	22	0	0	0	4483.5	25620	4212.3	TRUE
P39060-2	-	-	-	55477.667	0	**COL18A1**	**Collagen alpha-1(XVIII) chain;Endostatin**	11	0	0	0	52379	81164	32890	TRUE
P98160	-	-	-	451150	0	**HSPG2**	**Basement membrane-specific heparan sulfate proteoglycan core protein;Endorepellin;LG3 peptide**	72	0	0	0	391100	647610	314740	TRUE
Q14112-2	-	-	-	184193.33	0	**NID2**	**Nidogen-2**	29	0	0	0	220450	202090	130040	TRUE
Q9H8L6	-	-	-	2776.667	0	MMRN2	Multimerin-2	5	0	0	0	3353.7	4976.3	0	TRUE
Q9Y6C2	-	-	-	9162	0	EMILIN1	EMILIN-1	11	0	0	0	14931	12555	0	TRUE
