# Supplementary material for: Global proteomic analysis of extracellular matrix in mouse and human brain highlights relevance to cerebrovascular disease
Source: J Cereb Blood Flow Metab. 2021 Mar 17;41(9):2423–38. doi: 10.1177/0271678X211004307 (PMC8392779; doi:10.1177/0271678X211004307)
Supplement: sj-pdf-8-jcb-10.1177_0271678X211004307 - Supplemental material for Global proteomic analysis of extracellular matrix in mouse and human brain highlights relevance to cerebrovascular disease [file sj-pdf-8-jcb-10.1177_0271678X211004307.pdf]

Full unedited Western Blots for Figure 1 A, B

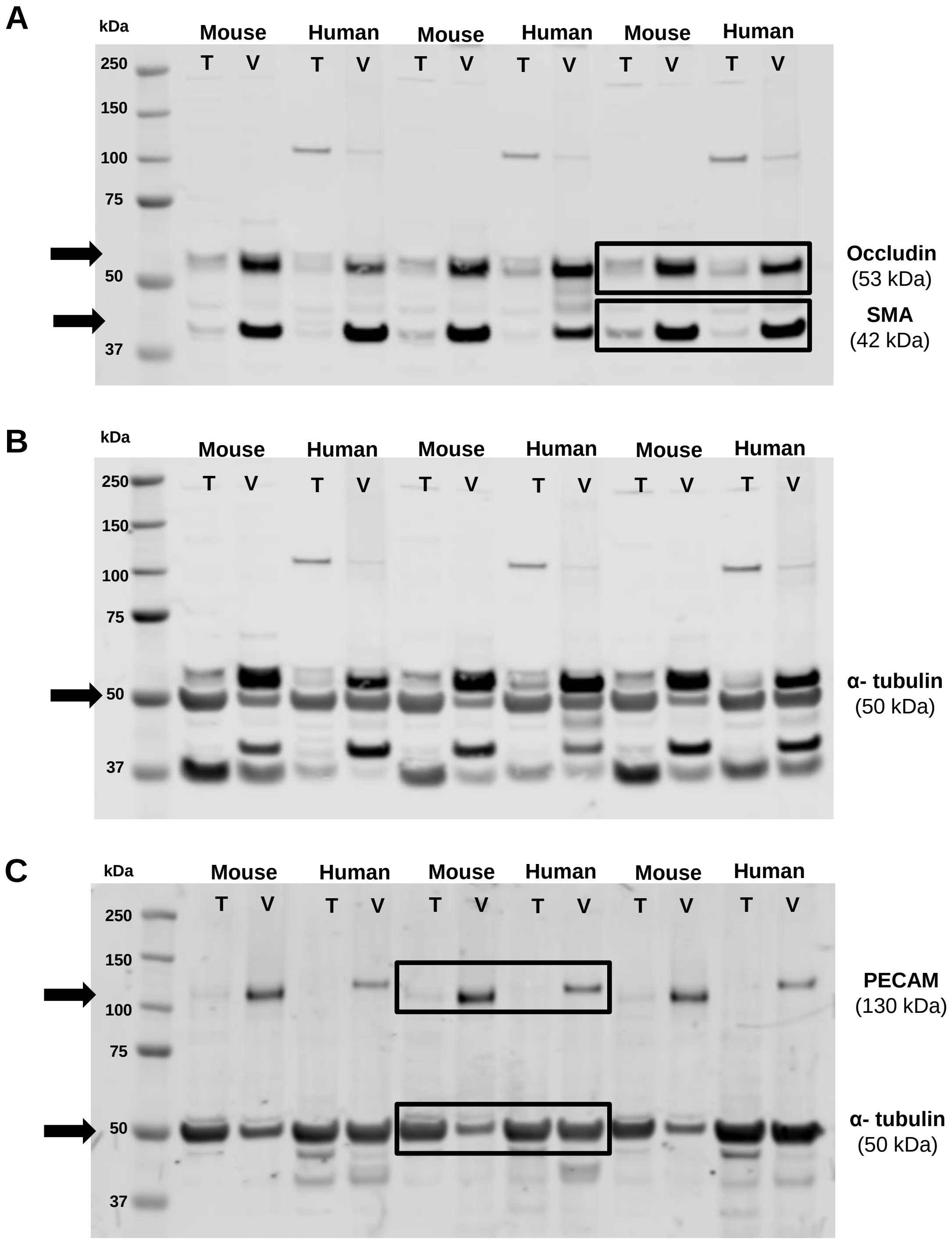

Figure S1. Full unedited Western blots of total and vessel enriched fractions in mouse and human samples in support of main Figure 1 A-B. All blots were re-probed with α-tubulin as a loading control to allow normalisation of proteins of interest (B,C). (A) Total and vessel enriched samples were probed for occludin and SMA proteins. (B) α-tubulin normalisation for Blot A. (C) Total and vessel enriched samples were probed for PECAM and normalised with α-tubulin. **Legend:** T = total, V = vessel enriched, ▭= bands used in Figure 1A,B.

Full unedited Western Blots for Figure 1 C,D

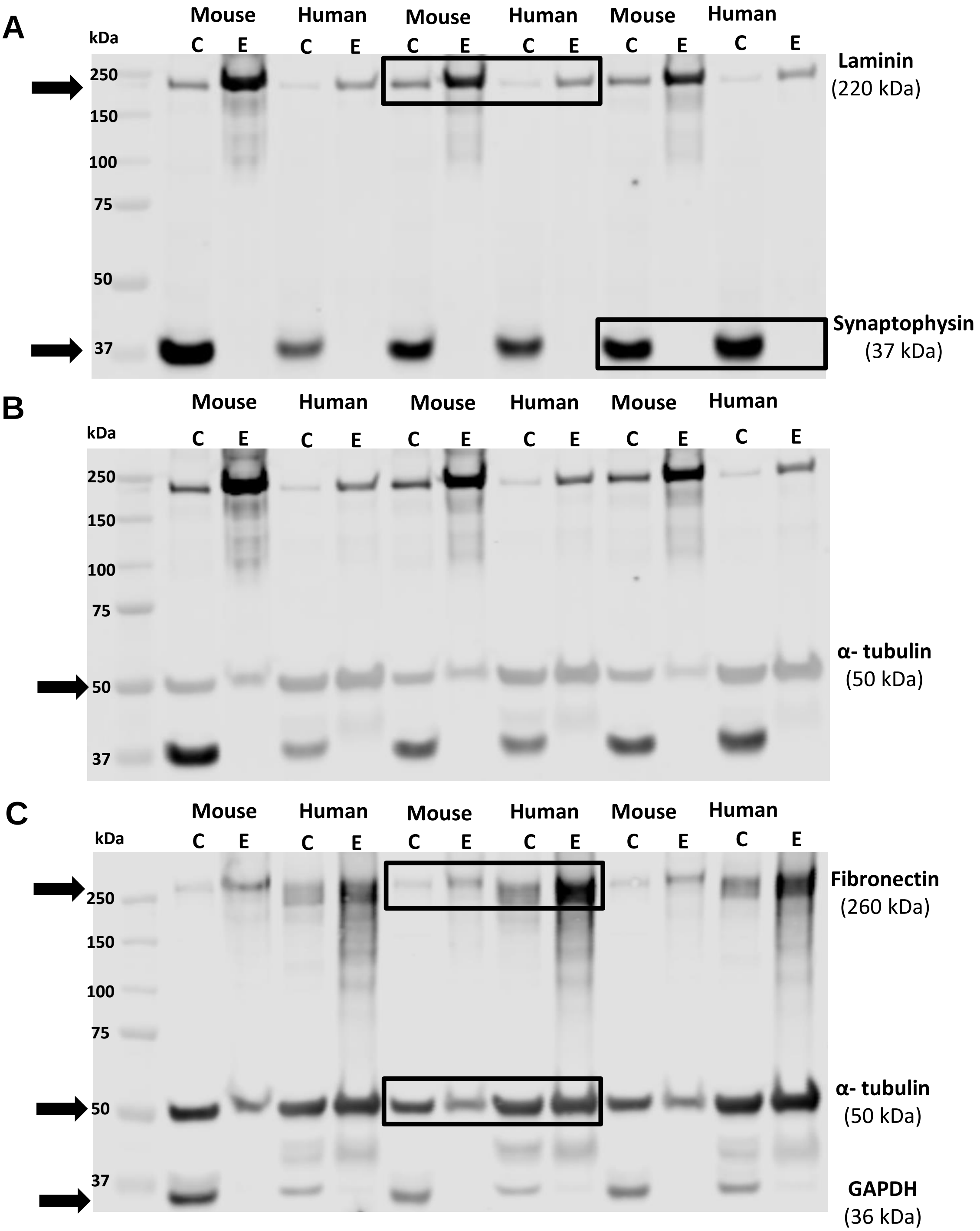

Figure S2. Full unedited Western blots of cellular and ECM enriched fractions in mouse and human samples in support of main Figure 1 C-D. All blots were re-probed with α-tubulin as a loading control to allow normalisation of proteins of interest (B,C). (A) Cellular and ECM enriched samples were probed for laminin and synaptophysin proteins. (B) α-tubulin normalisation for Blot A. (C) Cellular and ECM enriched samples were probed for fibronectin, GAPDH and normalised with α-tubulin. **Legend:** C = Cellular, E = ECM enriched, ▭= bands used in Figure 1 C,D.
